# Reactions of acryl thioamides with iminoiodinanes as a one-step synthesis of *N*-sulfonyl-2,3-dihydro-1,2-thiazoles

**DOI:** 10.3762/bjoc.21.104

**Published:** 2025-07-10

**Authors:** Vladimir G Ilkin, Pavel S Silaichev, Valeriy O Filimonov, Tetyana V Beryozkina, Margarita D Likhacheva, Pavel A Slepukhin, Wim Dehaen, Vasiliy A Bakulev

**Affiliations:** 1 TOS Department, Ural Federal University, 19 Mira str., Yekaterinburg 620002, Russia; 2 Department of Organic Chemistry, Perm State University, 15 Bukireva str., Perm 614990, Russia; 3 Department of Organic & Biomolecular Chemistry, Ural Federal University, 19 Mira str., Yekaterinburg 620002, Russia; 4 Postovsky Institute of Organic Synthesis, Ural Branch of Russian Academy of Sciences, 22/20 S. Kovalevskoy str., Yekaterinburg 620108, Russia; 5 Sustainable Chemistry for Metals and Molecules, Department of Chemistry, KU Leuven, Celestijnenlaan 200F, Leuven B-3001, Belgium

**Keywords:** iminoiodinanes, sulfonyl groups, synthesis, 1,2-thiazolines, thioamides

## Abstract

A one-step method has been developed for the preparation of 2,3-dihydro-2-sulfonyl-3,4,5-substituted 1,2-thiazoles by the reaction of acryl thioamides and iminoiodinanes. A library of 31 examples of tetrasubstituted 1,2-thiazoles was thus synthesized in high yields. The effectiveness of the synthesis method for these poorly studied 1,2-thiazoles was confirmed by scaling the reaction using gram amounts of the starting thioamide.

## Introduction

**Figure 1:**
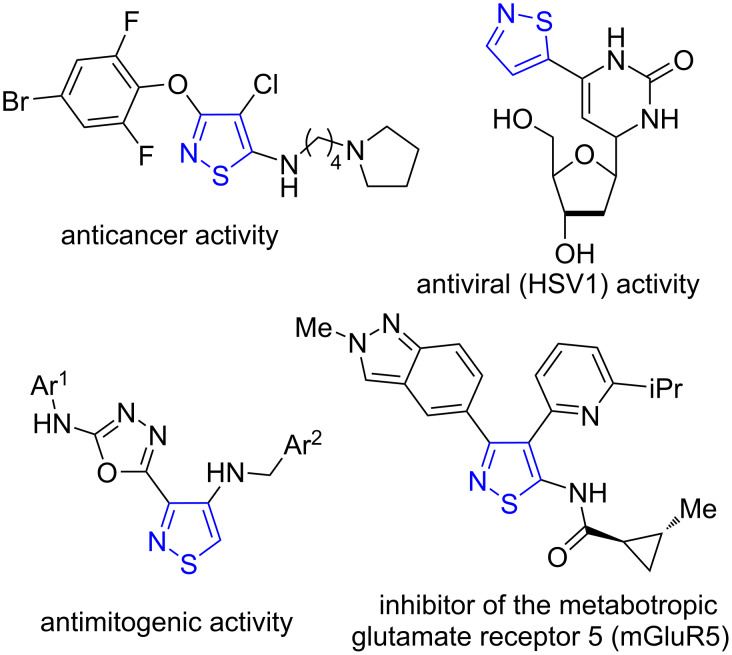
Representatives of biologically active 1,2-thiazoles.

1,2-Thiazoles (isothiazoles) exhibit a wide range of biological activity (Figure 1): antipoliovirus [1], anticancer [2–7], against Parkinson's disease [8], and diabetes [9–11], and are also used as microbiocides [12–13]. These data [1–5] inspired researchers to expand the chemistry of these compounds and to search for new synthetic methods and chemical transformations [14–15].

**Scheme 1:**
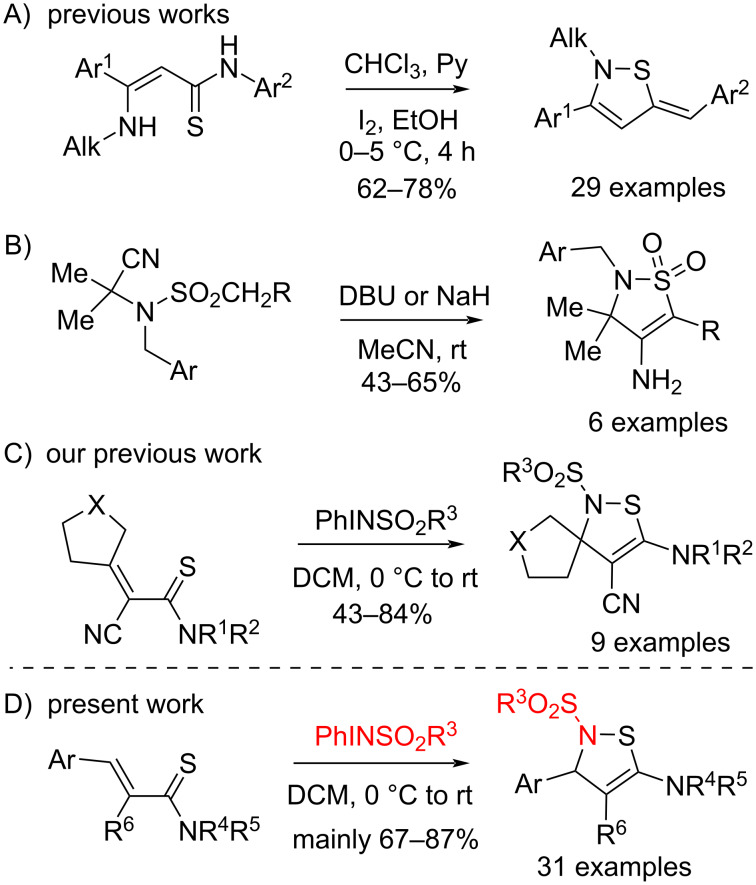
Synthesis of 2,5-dihydro-1,2-thiazoles.

Unlike the aromatic congeners, the chemistry of non-aromatic dihydro-1,2-thiazoles is less represented in the literature [16–18]. 2,5-Dihydro-1,2-thiazoles were synthesized by oxidative cyclization of *N*-arylamides of 3-(alkylamino)prop-2-enethiocarboxylic acids with iodine (Scheme 1А) [16].

2,3-Dihydro-1,2-thiazoles were first synthesized in 1997 by the reaction of 2,2-dimethyl-*N*-alkylsulfonyl-*N*-benzylaminoacetonitrile with strong bases in acetonitrile (Scheme 1B) [17]. Very recently, our group reported the synthesis of spirocyclic 2,3-dihydro-1,2-triazoles by the reaction of thioamides containing a cycloalkylidene fragment with *N*-tosyl and *N*-mesyliminoiodinanes (Scheme 1C) [18]. In the present work, an effective method for the synthesis of 2,3-dihydro-*N*-sulfonyl-1,2-thiazoles was developed based on the optimized reaction of acrylic acid thioamides containing aryl- and hetarylidene fragments with *N*-tosyl and *N*-mesyliminoiodinanes (Scheme 1D).

## Results and Discussion

The reaction of thioamide **1a** with PhINTs (**2a**) was chosen as a model for searching the optimal synthesis conditions (Table 1).

**Table 1:** Optimization of the reaction of thioamide **1a** with iodonium salts [I].

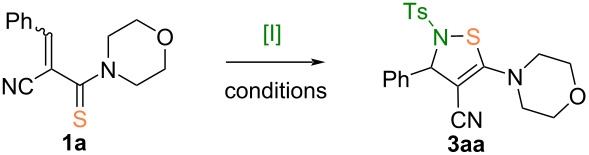					

Entry	Cat (mol %)	[I] (equiv)	Time	Solvent (*Т*, °С)	Yield, %

1	Rh_2_(Piv)_4_ (0.5)	PhINTs (1.5)	17 h	CHCl_3_ (50)	48
2	[Cu(MeCN)_4_]OTf (5)	PhINTs (1.5)	17 h	CHCl_3_ (50)	72
3	[Cu(MeCN)_4_]PF_6_ (5)	PhINTs (1.5)	10 min	DCM (0→24)	82
4	Cu(OAc)_2_ (5)	PhINTs (1.5)	10 min	DCM (0→24)	78
5	‒	PhINTs (1.1)	20 min	DCM (0→24)	56
6	‒	PhINTs (2.0)	10 min	DCM (0→24)	67
7	‒	PhINTs (1.5)	10 min	DCM (0→24)	78
8	‒	PhINTs (1.5)	10 min	EtOH (0→24)	‒^a^
9	‒	PhINTs (1.5)	10 min	DMSO (0→24)	‒^a^
10	‒	DMP^b^ (1.5)	10 min	DCM (0→24)	35^c^
11	‒	HTIB^d^ (1.5)	10 min	DCM (0→24)	‒^a^
12	‒	TsNH_2_ + PIDA^e^ (1.5)	10 min	DCM (0→24)	70

Conditions: thioamide **1a** (0.19 mmol), iodonium salt [I] (1.1–2.0 equiv), solvent (2.5 mL). ^a^The reaction does not occur. ^b^DMP = Dess–Martin periodinane. ^c^Yield of amide. ^d^HTIB = hydroxy(tosyloxy)iodobenzene. ^e^PIDA = PhI(OAc)_2_.

We found that when processing thioamide **1a** with PhINTs (**2a**, 1.5 equiv) in chloroform at 50 °C in the presence of Rh_2_(Piv)_4_ (0.5 equiv), 2,3-dihydro-*N*-sulfonyl-1,2-thiazole **3aa** is formed with a 48% yield (Table 1, entry 1). When using [Cu(MeCN)_4_]OTf instead of Rh_2_(Piv)_4_, the target product **3aa** was obtained in higher yield (72%, Table 1, entry 2) and with [Cu(MeCN)_4_]PF_6_, the yield of 1,2-thiazole **3aa** increased to 82%, while the reaction time decreased significantly (Table 1, entry 3). Using Cu(OAc)_2_ as a catalyst led to a slight decrease in the yield of the target product to 78% over the same time (Table 1, entry 4). When the reaction was carried out in the absence of metal catalysts (Table 1, entries 5 and 6), the yields of the target product **3aa** were generally lower (56–67%), and in some cases (Table 1, entries 8–11) the reaction did not occur. In the presence of metal catalyst PhINTs form a nitrenoid specie, containing electrophilic nitrogen. In metal-free conditions PhINTs participates in reactions as ylide with a nucleophilic nitrogen. We expected different reactivity of the two different forms of PhINTs. However, our expectations were not fulfilled.

**Scheme 2:**
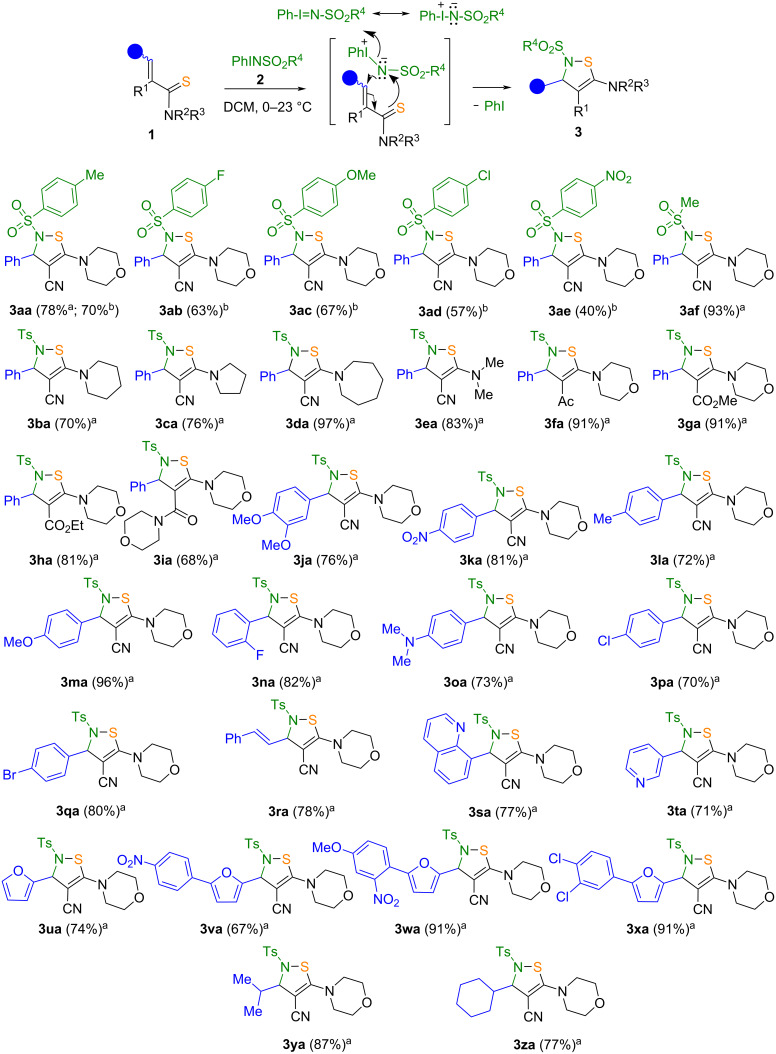
Synthesis of 2,3-dihydro-*N*-sulfonyl-1,2-thiazoles **3**. Conditions: ^a^Method A: thioamide **1** (1.0 equiv), PhINMs or PhINTs (**2a**, 1.5–2.0 equiv), 0→24 °C, 6‒60 min. ^b^Method B: thioamide **1** (1 equiv), aryl sulfonamide (1.2 equiv), PhI(OAc)_2_ (1.5 equiv), 0→24 °C, 10–30 min.

The exception is the data from entry 7 (Table 1), where the yield of compound **3aa** was 78%. Thus, the conditions described in entry 7 (absence of a catalyst, use of 1.5 equiv of PhINTs **2a** and dichloromethane (DCM) as a solvent at room temperature for 10 min) are optimal and were used for the synthesis of a number of 2,3-dihydro-*N*-sulfonyl-1,2-thiazoles **3** (Scheme 2, method A). The conditions described in entry 12 (Table 1; Scheme 2, method B) are also noteworthy, since they do not require the preliminary synthesis of iodonium salts **2**, which saves time and effort, however, the yields of 1,2-thiazoles **3aa**–**ae** are slightly lower (70 vs 78%) than by method A. We also investigated the effect of chiral catalysts or ligands in the reaction of thioamide **1a** with **2a**; however, we were unable to achieve high enantiomeric purity of product **3aa** (see Supporting Information File 1, Table S1).

Using the optimal reaction conditions (method A), twenty-seven 2,3-dihydro-*N*-sulfonyl-1,2-thiazoles **3** were synthesized in 69–96% yields (Scheme 2, method A). According to method B (Scheme 2), five compounds **3aa**–**ae** were obtained in moderate yields (40–70%). The formation of the final compounds starts by analogy with an aza-Michael reaction first with the addition of iminoiodinane accompanied with reorganization of double bonds including attack of the negatively charged sulfur atom on nitrogen with subsequent elimination of iodobenzene as good leaving group.

The diversity of the structure of the target compounds is provided by the variation of substituents in positions 2–5 of the 1,2-thiazole ring. Thus, arylsulfonyl groups containing a methyl, fluorine, chlorine, or nitro group at the aryl moiety, or a mesyl substituent were introduced into position 2 of the thiazole ring and various aryls, quinolinyl, pyridinyl, furyl, isopropyl, and cyclohexyl substituents were introduced into position 3; cyano and various carbonyl groups were added to position 4 and cyclic amine residues were added to position 5 which increase the solubility of the synthesized compounds **3** (Scheme 2). An analysis of the yields of compounds **3aa**–**af** allowed us to conclude that these decreased when electron-accepting groups (R^4^) were present in the iminoiodinane reactant. Thus, the replacement of the methyl group in **3af** with a tolyl group in **3aa** led to a 15% decrease of the yield of the target product. This effect can be explained by an increase of the electron density on the nitrogen atom of the iodonium salt **2** when the tosyl group is replaced by a less electron-deficient mesyl group. Using optimal reaction conditions (method A), twenty seven 2,3-dihydro-*N*-sulfonyl-1,2-thiazoles were synthesized in 69‒96% yields. We scaled the reaction of thioamide **1q** with iodonium salt **2a** and found that a 20-fold increase of thioamide loading only slightly (by 6%) reduced the yield of the target product **3qa** (see Supporting Information File 1). These data indicate the possibility of using the developed method for the synthesis of compounds **3** on a larger or even commercial scale.

**Figure 2:**
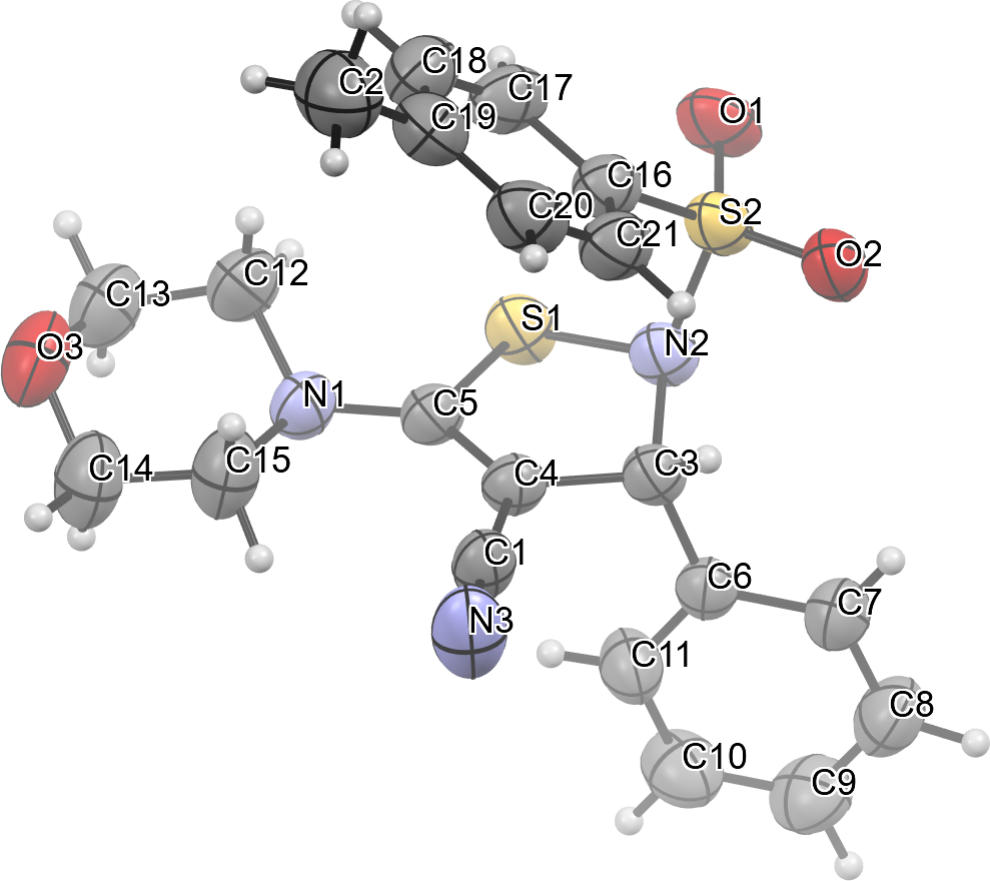
Compound **3aa** in thermal ellipsoids 50% probability.

The structures of compounds **3** were confirmed by ^1^H and ^13^C NMR spectroscopy and high-resolution mass spectrometry. The ^1^H NMR spectra are characterized by signals from protons of the aromatic rings in the range of 8.08–7.32 ppm, singlets for the proton at the C-3 position of the 2,3-dihydro-1,2-thiazole ring in the range of 6.44–6.06 ppm, and multiplets corresponding to the morpholine fragment in the range of 3.80–2.89 ppm or protons of residues of other amino groups in the range of 3.51–1.31 ppm. In the ^13^C NMR spectra, characteristic signals of the C-4 atom of the 2,3-dihydro-1,2-thiazole ring are in the range of 75.3–72.4 ppm and signals of the carbon atom of the cyano group in the range of 117.7–116.6 ppm. The structure of 2,3-dihydro-1,2-thiazole **3aa** was additionally confirmed by single crystal X-ray diffraction (Figure 2). According to the X-ray diffraction data, compound **3aa** crystallizes in the centrosymmetric spatial group of the monoclinic system.

The molecule has a tweezer-like conformation due to π–π interactions between the electron-donating tolyl and the electron-acceptor fragment NC‒C=C‒S. The heterocyclic fragment in the molecule is non-planar, the atom N(2) deviates from the RMS plane S(1)C(3)C(4)C(5) by 0.461 Å. The atom of the N(1) morpholine fragment has a planar configuration with significant asymmetry in the lengths of C–N bonds. Due to the electron-acceptor effects of substituents, the C(3)‒H bond exhibits significant polarity and is involved in the formation of a weak intermolecular hydrogen bond of the C‒H···N≡C type with distances H(3)···N(3) 2.53(3) Å, C(3)···N(3) 3.436(4) Å and angle C(3)H(3)N(3) 157(2)° (symmetry transformation [1−x, 1−y, 1−z]).

## Conclusion

Thus, we found that thioamides of acrylic acid easily interact with iodonium salts. The search for optimal conditions for the process has been carried out. The optimized reaction found to proceed in the absence of metal catalysts, using 1.5 equiv of the iodonium salt at room temperature in DCM. Using the optimized procedure, a library of 31 novel 2,3-dihydro-1,2-thiazoles was synthesized.

## Experimental

Thioamides **1a**,**f**,**g**,**l**,**r** [19], **1b**,**c**,**d**,**e**,**j**,**u**,**x** [20] and iodonium salts **2a** [21] and **2f** [22] were synthesized according to the previously described methods. The structures of all thioamides used in this study are provided in Supporting Information File 1.

**Preparation of thioamides 1h,i,k,n,o,s,t,v,w,y,z (general procedure).** A mixture of the corresponding thioacetamide (1.0 equiv), aldehyde (1.1–4.0 equiv) and DBU (0.1 equiv or 1.0 equiv for **1h**,**o**) in ethanol was stirred for 2–23 h at room temperature. For thioamide **1i**, the reaction time was 96 h at 80 °C. The formed precipitate was filtered off and washed with cold ethanol and diethyl ether.

**Preparation of 2-sulfonyl-2,3-dihydro-1,2-thiazoles 3 (general procedure).** Method A. The corresponding thioamide **1** (1.0 equiv) and DCM (1 mL) was added to an oven-dried standard microwave vial with a volume of 10 mL. The resulting solution was stirred for 10 min in an ice bath, then iodonium salt **2a** or **2f** (1.5–2.0 equiv) was added in one portion. The reaction vessel was removed from the ice bath and the reaction mass was stirred for 6–60 min, then transferred to a silica gel column and the corresponding 2-sulfonyl-2,3-dihydro-1,2-thiazole **3** was isolated.

Method B. The corresponding aryl sulfonamide (1.2 equiv), PhI(OAc)_2_ (1.5 equiv) and DCM (0.5 mL) was added to an oven-dried standard microwave vial with a volume of 10 mL. The resulting suspension was stirred for 10 min in an ice bath, then thioamide **1** (1.0 equiv) dissolved in DCM (1.5 mL) was added dropwise. The reaction vessel was removed from the ice bath and the reaction mass was stirred for 10–30 min, then transferred to a silica gel column and the corresponding 2-sulfonyl-1,3-thiazole **3** was isolated.

**X-ray structure determination of 3aa.** Crystal data for C_21_H_21_N_3_O_3_S_2_ (*M* = 427.53 g/mol): monoclinic, space group *P*2_1_/*n*, *a* = 14.8770(14) Å, *b* = 8.7007(6) Å, *c* = 17.5854(15) Å, β = 110.474(11), *V* = 2132.5(3) Å^3^, *Z* = 4, *T* = 295(2) K, μ(Mo Kα) = 0.277 mm^−1^, *D*_calc_ = 1.332 g/cm^3^, 14068 reflections measured (7.492° ≤ 2Θ ≤ 62.136°), 5660 unique (R_int_ = 0.0465, R_sigma_ = 0.0585). The final R_1_ = 0.0584, wR_2_ = 0.1508 (I > 2σ(I)) and R_1_ = 0.1081, wR_2_ = 0.2069 (all data). GooF = 1.027. Largest diff. peak/hole 0.31/−0.40 eÅ^−3^.

The experiment was performed on an automatic four-circle X-ray diffractometer "Xcalibur 3" with a CCD detector according to the standard procedure (Mo Kα irradiation, graphite monochromator, ω-scanning in 1° increments at *T* = 295(2) K). An empirical correction for absorption has been introduced. Using the Olex2 [23] software shell, the structure was solved using the SHELXT program and refined using the SHELXL [24–25] program with a full-matrix F^2^ MNC for non-hydrogen atoms. The H-atoms in C–H bonds are placed in the calculated positions and refined in the "rider" model in the isotropic approximation.

CCDC 2401684 (**3aa**) contains the supplementary crystallographic data for this paper. These data can be obtained free of charge via https://www.ccdc.cam.ac.uk (or from the CCDC, 12 Union Road, Cambridge CB2 1EZ, UK; Fax: +44 1223 336033; E-mail: deposit@ccdc.cam.ac.uk).

## Supporting Information

File 1 Full experimental details and characterization data of all new compounds.

File 2 Copies of NMR and HRMS spectra of all new compounds.

## Data Availability

All data that supports the findings of this study is available in the published article and/or the supporting information of this article.
