# Supplementary material for: Reactions of acryl thioamides with iminoiodinanes as a one-step synthesis of N-sulfonyl-2,3-dihydro-1,2-thiazoles
Source: Beilstein J Org Chem. 2025 Jul 10;21:1397–403. doi: 10.3762/bjoc.21.104 (PMC12256786; doi:10.3762/bjoc.21.104)
Supplement: File 1 — Full experimental details and characterization data of all new compounds. [file Beilstein_J_Org_Chem-21-1397-s001.pdf]

BEILSTEIN JOURNAL OF ORGANIC CHEMISTRY

Supporting Information

for

Reactions of acryl thioamides with iminoiodinanes as a one-step synthesis of *N*-sulfonyl-2,3-dihydro-1,2-thiazoles

Vladimir G. Ilkin, Pavel S. Silaichev, Valeriy O. Filimonov, Tetyana V. Beryozkina, Margarita D. Likhacheva, Pavel A. Slepukhin, Wim Dehaen and Vasiliy A. Bakulev

Beilstein J. Org. Chem. **2025,** *21,* 1397–1403. doi:10.3762/bjoc.21.104

Full experimental details and characterization data of all new compounds

License and Terms: This is a supporting information file under the terms of the Creative Commons Attribution License (https://creativecommons.org/licenses/by/4.0). Please note that the reuse, redistribution and reproduction in particular requires that the author(s) and source are credited and that individual graphics may be subject to special legal provisions.
The license is subject to the *Beilstein Journal of Organic Chemistry* terms and conditions: (https://www.beilstein-journals.org/bjoc/terms)

All reagents and solvents obtained from commercial sources were used without additional purification. The reaction progress and purity of the obtained compounds were controlled by TLC on Sorbfil UV-254 plates (Imid, Krasnodar, Russia) and visualization under UV light. Column chromatography was performed using silica gel with a particle size of 40–63 µm. Melting points were determined on a melting point apparatus Stuart SMP10 (Cole-Parmer Ltd, Staffordshire, UK) and are uncorrected. All NMR spectra were recorded with a Bruker Avance II spectrometer (Karlsruhe, Germany) at 400 MHz, (^{1}H NMR), 101 MHz (^{13}C NMR), 376 MHz (^{19}F NMR) in $CDCl_3$ and DMSO-d_6. The signal of the minor isomer in the NMR spectra of the initial thioamides is marked with an asterisk (*). High-resolution mass spectra (HRMS) were recorded using the ultrahigh resolution quadrupole time-of-flight mass spectrometer Bruker maXis impact HD (USA) with the electrospray ionization probe installed coupled with Agilent 1260 HPLC system. The X-ray diffraction experiment was carried out using the equipment of the CCP "CAOS" of the IOC of the Ural Branch of the Russian Academy of Sciences. The X-ray analysis was performed using an Xcalibur R Mo diffractometer (Agilent technologies, UK). An empirical correction for absorption has been introduced. Using the Olex2 software shell, the structure was solved using the SHELXT program and refined using the SHELXL program with a full-matrix F2 MNC for non-hydrogen atoms. The H-atoms in C–H bonds are placed in the calculated positions and refined in the "rider" model in the isotropic approximation.

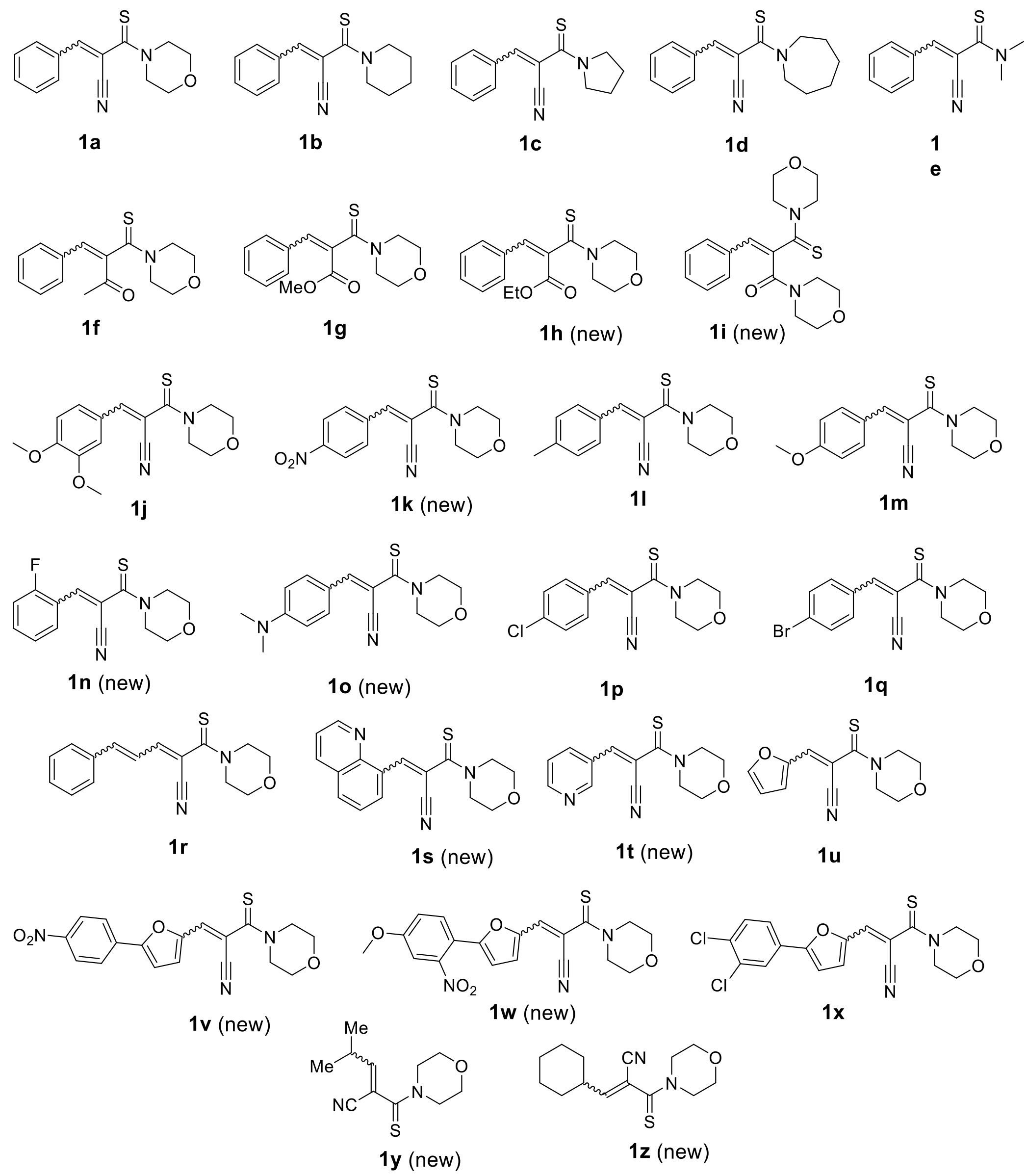

Scheme S1. Thioamides used in the research

Preparation of thioamides 1h,i,k,n,o,s,t,v,w,y,z (general procedure). A mixture of the corresponding thioacetamide (1.0 equiv), aldehyde (1.1–4.0 equiv) and DBU (0.1 equiv and 1.0 equiv for **1h**,**o**) in ethanol was stirred for 2–23 h at room temperature. For thioamide **1i**, the reaction time was 96 h at 80 °C. The formed precipitate was filtered off and washed with cold ethanol and diethyl ether.

Ethyl 2-(morpholine-4-carbonothioyl)-3-phenyl acrylate (1h) was obtained from ethyl 3-morpholine-3-thioxopropanoate (434 mg, 2.0 mmol), benzaldehyde (233 mg, 2.2 mmol) and DBU (298 mg, 2.0 mmol) in ethanol (4 mL), reaction time is 18 h. Yield 254 mg (42%);

pale yellow powder; mp 96–97 °C; ^{1}H NMR ($CDCl_3$) δ (*J*, Hz) 7.61–7.57 (2H, m, H Ar), 7.38–7.34 (4H, m, HC= + H Ar), 4.47–4.41 (1H, m, CH_2), 4.37–4.24 (3H, m, $CH_3C\underline{H}_2O$ + CH_2), 3.86–3.73 (2H, m, CH_2), 3.68–3.49 (3H, m, CH_2), 3.30–3.25 (1H, m, CH_2), 1.34 (3H, t, *J* 7.1, $\underline{CH}_3CH_2O$); ^{13}C NMR ($CDCl_3$) δ 194.6, 164.7, 135.1, 133.3, 131.8, 130.3, 129.0, 66.2, 66.1, 61.8, 51.5, 48.2, 14.3; HRMS–ESI-TOF (*m/z*): $[M + H]^+$ calcd. for $C_{16}H_{20}O_3NS^+$, 306.1164; found: 306.1160.

2-(Morpholino-4-carbonothioyl)-1-morpholino-3-phenylprop-2-en-1-one (1i) was obtained from 1,3-dimorpholino-3-thioxopropane-1-one (258 mg, 1.0 mmol), benzaldehyde (424.5 mg, 4.0 mmol) and DBU (15 mg, 0.1 mmol) in ethanol (10 mL), reaction time is 96 h at 80 °C. Yield 125 mg (36%); pale yellow powder; mp 156–157 °C; ^{1}H NMR ($CDCl_3$) δ (*J*, Hz) 7.43 (2H, d, *J* 8.8, H Ar), 7.38–7.28 (3H, d, H Ar), 6.17 (1H, s, HC=), 4.60–4.52 (1H, m, CH_2), 4.32–4.18 (1H, m, CH_2), 4.09–3.96 (3H, m, CH_2), 3.86–3.57 (8H, m, CH_2), 3.50–3.30 (2H, m, CH_2), 3.24–3.15 (1H, m, CH_2); ^{13}C NMR ($CDCl_3$) δ 193.4, 167.0, 133.9, 133.0, 129.2, 129.0, 128.8, 124.1, 67.6, 66.7, 66.5, 65.9, 52.0, 48.6, 48.1, 42.5; HRMS–ESI-TOF (*m/z*): $[M + H]^+$ calcd. for $C_{18}H_{23}N_2O_3S^+$, 347.1424; found: 347.1425.

2-(Morpholine-4-carbothioyl)-3-(4-nitrophenyl)prop-2-enenitrile (1k) was obtained from 3-morpholino-3-thoxopropionitrile (341 g, 2.0 mmol), 4-nitrobenzaldehyde (302 mg, 2.0 mmol), DBU (30 mg, 0.2 mmol) in ethanol (4 mL), reaction time is 23 h. Yield 516 mg (85%); yellow powder; mp 160–161 °C; ^{1}H NMR ($CDCl_3$) δ (*J*, Hz) 8.30 (2H, d, *J* 8.8, H Ar), 8.24* (2H*, d, *J* 8.7, H Ar), 7.99 (2H, d, *J* 8.8, H Ar), 7.67* (2H*, d, *J* 8.7, H Ar), 7.55 (1H, s, HC=), 7.07* (1H*, s, HC=), 4.51–3.30 (8H + 8H*, m, CH_2); ^{13}C NMR ($CDCl_3$) δ: 190.3, 187.4*, 149.2, 148.8*, 145.2*, 138.3*, 138.0, 137.4*, 130.40*, 130.38, 124.4, 116.4*, 116.1, 115.1, 66.4, 66.1*, 65.9*, 53.1*, 51.9, 50.4*, 48.6; HRMS–ESI-TOF (*m/z*): $[M + H]^+$ calcd. for $C_{14}H_{14}O_3N_3S^+$, 304.0756; found: 304.0753.

3-(2-Fluorophenyl)-2-(morpholine-4-carbothioyl)prop-2-enenitrile (1n) was obtained from 3-morpholino-3-thioxopropionitrile (680 mg, 4.0 mmol), 2-fluorobenzaldehyde (496 mg, 4.0 mmol), and DBU (60 mg, 0.4 mmol) in ethanol (12 mL), reaction time is 23 h. Yield 624 mg (57%); yellow powder; mp 138–139 °C; ^{1}H NMR ($CDCl_3$) δ (*J*, Hz) 8.21 (1H, dt, *J* 7.8, 1.2, H Ar), 7.72 (1H, s, HC=), 7.51–7.44 (1H, m, H Ar), 7.26 (1H, t, *J* 7.3, H Ar), 7.14 (1H, m, H Ar), 4.36–3.77 (8H, m, CH_2); ^{13}C NMR ($CDCl_3$) δ (*J*, Hz) 191.43, 162.2 (d, *J* 255.0), 139.7 (d, *J* 7.0), 133.7 (d, *J* 9.0), 128.5 (d, *J* 0.9), 124.9 (d, *J* 3.7), 120.7 (d, *J* 11.2), 116.2 (d, *J* 21.8), 115.6, 114.1 (d, *J* 2.2), 66.4, 53.0, 50.5; ^{19}F MNR ($CDCl_3$) δ -112.85*, -113.21; HRMS–ESI-TOF (*m/z*): $[M + H]^+$ calcd. for $C_{14}H_{14}FN_2OS^+$, 277.0811; found: 277.0809.

3-(4-(Dimethylamino)phenyl)-2-(morpholine-4-carbothioyl)prop-2-enenitrile (1o) was obtained from 3-morpholino-3-thioxopropionitrile (300 mg, 1.76 mmol), 4-(dimethylamino)benzaldehyde (368 mg, 2.47 mmol), and DBU (268 mg, 1.76 mmol) in ethanol (2 mL), reaction time is 23 h. Yield 246 mg (46%); pale yellow powder; mp 144–146 °C; ^{1}H NMR (DMSO-d_6) δ, (*J*, Hz) 7.32 (2H, d, *J* 8.8, H Ar), 7.16 (1H, c, HC=), 6.74 (2H, d, *J* 8.9, H Ar), 4.30–4.19 (2H, m, CH_2), 3.83–3.74 (3H, m, CH_2), 3.58–3.51 (2H, m, CH_2), 3.30–3.25 (1H, m, CH_2), 2.99 (6H, s, CH_3); ^{13}C NMR ($CDCl_3$) δ 190.8, 152.1, 141.7, 132.0, 120.0, 118.5, 111.7, 106.0, 66.1, 65.9, 51.5, 48.5, 40.0; HRMS–ESI-TOF (*m/z*): $[M + H]^+$ calcd. for $C_{16}H_{19}N_3OSNa^+$, 324.1141; found: 324.1139.

2-(Morpholine-4-carbothioyl)-3-(quinolin-8-yl)prop-2-enenitrile (1s) was obtained from 3-morpholino-3-thioxopropionitrile (300 mg, 1.76 mmol), quinoline-8-carbaldehyde (332 mg, 2.11 mmol) and DBU (27 mg, 0.18 mmol) in ethanol (3.5 mL), reaction time is 23 h. Yield 410 mg (75%); pale yellow powder; mp 192–194 °C; ^{1}H NMR (DMSO-d_6) δ (*J*, Hz) 9.00 (1H, d, *J* = 2.4, H Ar), 8.58 (1H, s, HC=), 8.50–8.47 (2H, m, H Ar), 8.41* (1H, s, H Ar), 8.19 (1H, d, *J* 8.0, H Ar), 8.15* (1H, d, *J* 8.0, H Ar), 8.18–8.14* (1H, m, H Ar), 7.93* (1H, d, *J* 6.6, H Ar), 7.79 (1H, t, *J* 7.7, H Ar), 7.72* (d, 1H, *J* 7.2, H Ar), 7.68–7.65

(1H, m, H Ar), 4.29 (2H, br. s, CH_2), 4.07 (2H, br. s, CH_2), 3.81 (2H, br. s, CH_2), 3.75 (2H, br. s, CH_2), 3.51–3.40* (4H, m, CH_2), 2.64–2.59* (4H, m, CH_2); ^{13}C NMR ($CDCl_3$) 189.3*, 187.1, 151.0*, 150.9, 145.4*, 145.0, 141.8, 137.5*, 136.9, 131.8*, 131.5, 130.1, 129.6*, 128.8*, 127.9, 126.4*, 126.3, 122.5*, 122.5, 116.2, 112.5, 66.0*, 65.5*, 65.0, 52.8*, 51.5, 49.6*, 48.3; HRMS–ESI-TOF (*m/z*): $[M + H]^+$ calcd. for $C_{17}H_{16}N_3OS^+$, 310.1008; found: 310.1009.

2-(Morpholine-4-carbothioyl)-3-(pyridin-3-yl)prop-2-enenitrile (1t) was obtained from 3-morpholino-3-thioxopropionitrile (340 mg, 2.0 mmol), nicotinic aldehyde (225 mg, 2.1 mmol) and DBU (30 mg, 0.2 mmol) in ethanol (4 mL), reaction time is 23 h. Yield 420 mg (81%); yellow powder; mp 87–89 °C; ^{1}H NMR ($CDCl_3$) δ (*J*, Hz) 8.82 (1H, d, *J* 2.4, H Ar), 8.69–8.66 (1H + 1H*, m, H Ar), 8.62* (1H, dd, *J* 4.8, 1.4, H Ar), 8.39 (1H, dt, *J* 8.1, 1.4, H Ar), 7.83* (1H, dt, *J* 8.0, 1.4, H Ar), 7.53 (1H, s, HC=), 7.41 (1H, dd, *J* 8.1, 4.8, H Ar) 7.33* (1H, dd, *J* 8.0, 4.8, H Ar), 7.02* (1H, s, HC=), 4.45–3.26 (8H + 8H*, m, CH_2); ^{13}C NMR ($CDCl_3$) δ 191.0, 187.9*, 152.4, 151.7, 150.8*, 145.1, 137.0*, 136.0*, 135.0, 128.6*, 128.4, 124.0, 123.8*, 116.4*, 115.4, 115.2*, 114.6, 66.4, 66.1*, 65.9*, 53.0*, 51.9, 50.4*, 48.5; HRMS–ESI-TOF (*m/z*): $[M + H]^+$ calcd. for $C_{13}H_{14}ON_3S^+$, 260.0858; found: 260.0854.

2-(Morpholine-4-carbothioyl)-3-(5-(4-nitrophenyl)furan-2-yl)prop-2-enenitrile (1v) was obtained from 3-morpholino-3-thioxopropionitrile (150 mg, 0.88 mmol), 5-(4-nitrophenyl)furan-2-carbaldehyde (229 mg, 1.06 mmol) and DBU (27 mg, 0.088 mmol) in ethanol (10 mL), reaction time is 4.5 h. Yield 281 mg (86%); orange powder; mp 212–215 °C; ^{1}H NMR (DMSO-*d*$_6$) δ (*J*, Hz) 8.33 (2H, d, *J* 8.7, H Ar), 8.07 (2H, d, *J* 8.7, H Ar), 7.57 (1H, d, *J* 3.6, H Fur), 7.49 (1H, s, HC=), 7.32 (1H, d, *J* 3.6, H Fur), 4.20 (2H, br. s, CH_2), 3.97 (2H, br. s, CH_2), 3.74 (4H, br. s, CH_2); ^{13}C NMR ($CDCl_3$) δ 189.1, 187.8*, 154.6*, 154.0, 149.3*, 149.2, 146.9, 134.3, 134.3*, 130.7, 126.3*, 125.7*, 125.0, 124.8*, 124.4*, 124.3, 121.9, 121.1*, 116.9*, 116.2, 112.7, 112.6*, 107.6*, 107.3, 65.5, 65.0*, 51.4, 48.3; HRMS–ESI-TOF (*m/z*): $[M + H]^+$ calcd. for $C_{18}H_{16}N_3O_4S^+$, 370.0856; found: 370.0859.

3-(5-(4-Methoxy-2-nitrophenyl)furan-2-yl)-2-(morpholine-4-carbothioyl)prop-2-enenitrile (1w) was obtained from 3-morpholino-3-thioxopropionitrile (200 mg, 1.17 mmol), 5-(2-methoxy-4-nitrophenyl)furan-2-carbaldehyde (348 mg, 1.41 mmol) and DBU (18 mg, 0.12 mmol) in ethanol (20 mL), reaction time is 2 h. Yield 435 mg (93%); bright yellow powder; mp 167–170 °C; ^{1}H NMR (DMSO-d_6) δ (*J*, Hz) 7.88 (1H, d, *J* 8.8, H Ar), 7.59 (1H, d, *J* 2.4, H Fur), 7.42–7.38 (2H, m, HC= + H Ar), 7.29 (1H, d, *J* 3.7, H Ar), 6.89 (1H, d, *J* 3.7, H Ar), 4.18 (2H, br. s, CH_2), 3.90 (5H, br. s, CH_2 + OMe), 3.73 (4H, br. s, CH_2); ^{13}C NMR ($CDCl_3$) δ 189.2, 160.2, 151.4, 148.4, 148.1, 131.0, 130.5, 121.5, 118.7, 116.1,114.1, 111.6, 109.8, 106.6, 65.6, 56.3, 52.7, 49.8; HRMS–ESI-TOF (*m/z*): $[M + H]^+$ calcd. for $C_{19}H_{17}N_3O_5S^+$, 400.0961; found: 400.0965.

4-Methyl-2-(morpholine-4-carbothioyl)pent-2-enenitrile (1y) was obtained from 3-morpholino-3-thioxopropanonitrile (341 mg, 2.0 mmol), isobutyraldehyde (160 mg, 1.1 2.20 mmol), DBU (30 mg, 0.20 mmol) in ethanol (4 mL), reaction time is 12 h. Yield 323 mg (72%); yellow oil; ^{1}H NMR ($CDCl_3$) δ (*J*, Hz) 6.70 (1H, d, *J* 10.1, HC=), 6.20* (1H, d, *J* 10.7, HC=), 4.34–3.70 (8H + 8H*, m, CH_2), 2.93 (1H, m, $(Me)_2C\underline{H}$), 2.70* (1H, m, $(Me)_2C\underline{H}$), 1.15 (3H, s, Me), 1.13 (3H, s, Me), 1.06* (3H, s, Me), 1.04* (3H, s, Me); ^{13}C NMR ($CDCl_3$) δ 191.2, 188.2*, 161.5, 154.9*, 115.9*, 115.4, 114.3, 113.8*, 66.5*, 66.4, 52.7*, 52.1, 50.4*, 48.8, 31.7, 29.7*, 21.6, 21.2*; HRMS–ESI-TOF (*m/z*): $[M + H]^+$ calcd. for $C_{11}H_{17}N_2OS^+$, 225.1056; found: 225.1058.

3-Cyclohexyl-2-(morpholine-4-carbothioyl)prop-2-enenitrile (1z) was obtained from 3-morpholino-3-thioxopropanonitrile (341 mg, 2.0 mmol), cyclohexylcarbaldehyde (224 mg, 2.1 mmol), DBU (30 mg, 0.20 mmol) in ethanol (4 mL), reaction time is 12 h. Yield 513 mg (97%); yellow oil; ^{1}H NMR ($CDCl_3$) δ (*J*, Hz) 6.73 (1H, d, *J* 10.1, HC=), 6.23* (1H*, d, *J* 10.7, HC=), 3.70–4.33 (8H + 8H*, m, CH_2 Morph), 2.63 (1H, m, CH), 2.42* (1H*, m, CH), 1.84–1.13 (10H + 10H*, m, CH_2 Cyclohex); ^{13}C NMR ($CDCl_3$) δ 191.4, 188.3*, 160.2, 153.6*, 116.1*, 115.5, 114.5, 114.1*, 66.5*, 66.3, 52.7*, 52.1, 50.4*, 48.8, 41.1, 39.2*, 31.6,

31.0*, 25.63*, 25.57, 25.1, 25.0*; HRMS–ESI-TOF (*m/z*): $[M + H]^+$ calcd. for $C_{14}H_{21}N_2OS^+$, 265.1369; found: 265.1370.

Preparation of 2-sulfonyl-2,3-dihydro-1,2-thiazoles 3 (general procedure)

Method A. The corresponding thioamide **1** (1.0 equiv) and DCM (1 mL) was added to an oven-dried standard microwave vial with a volume of 10 mL. The resulting solution was stirred for 10 min in an ice bath, then iodonium salt **2a** or **2f** (1.5–2.0 equiv) was added in one portion. The reaction vessel was removed from the ice bath and the reaction mass was stirred for 6–60 min, then transferred to a silica gel column and the corresponding 2-sulfonyl-2,3-dihydro-1,2-thiazole **3** was isolated.

Method B. The corresponding aryl sulfonamide (1.2 equiv), $PhI(OAc)_2$ (1.5 equiv) and DCM (0.5 mL) was added to an oven-dried standard microwave vial with a volume of 10 mL. The resulting suspension was stirred for 10 min in an ice bath, then thioamide **1** (1.0 equiv) dissolved in DCM (1.5 mL) was added dropwise. The reaction vessel was removed from the ice bath and the reaction mass was stirred for 10–30 min, then transferred to a silica gel column and the corresponding 2-sulfonyl-1,3-thiazole **3** was isolated.

5-Morpholino-3-phenyl-2-tosyl-2,3-dihydro-1,2-thiazole-4-carbonitrile (3aa) was obtained by method A from 2-(morpholine-4-carbonothioyl)-3-phenylacrylonitrile (**1a**, 50 mg, 0.19 mmol) and PhINTs (**2a**, 108 mg, 0.29 mmol) in DCM (2 mL), reaction time is 10 min. The purification of the crude product by column chromatography on SiO_2 (DCM/EtOAc, gradient 25:0 → 23.5:1.5) afforded **3aa** as a colorless powder (78%, 65 mg); mp 184–186 °C; ^1H NMR ($CDCl_3$) δ (*J*, Hz) 7.91 (2H, d, *J* 8.2, H Ar), 7.50–7.36 (7H, m, H Ar), 6.12 (1H, s, $CH_{thiazol}$), 3.58–3.56 (4H, m, CH_2), 3.33–3.24 (4H, m, CH_2), 2.51 (3H, s, Me); ^{13}C NMR ($CDCl_3$) δ 164.3, 145.7, 136.1, 131.2, 129.6, 129.3, 129.0, 128.8, 127.0, 116.6, 73.7, 68.9, 66.0, 51.2, 21.8; HRMS–ESI-TOF (*m/z*): $[M + H]^+$ calcd. for $C_{21}H_{22}N_3O_3S_2^+$, 428.1097; found: 428.1099.

2-Sulfonyl-1,2-thiazole **3aa** was obtained by method B from thioamide **1a** (40 mg, 0.15 mmol), 4-methylbenzenesulfonamide (32 mg, 0.18 mmol), $PhI(OAc)_2$ (75 mg, 0.22 mmol) in DCM (2 mL), reaction time is 30 min. The purification of the crude product by column chromatography on SiO_2 (DCM/EtOAc, gradient 25:0 → 23.5:1.5) afforded **3aa** as a colorless powder (70%, 46 mg); mp 184–186 °C.

X-ray structure determination of 3aa

Crystal data for $C_{21}H_{21}N_3O_3S_2$ (M = 427.53 g/mol): monoclinic, space group $P2_1/n$, a = 14.8770(14) Å, b = 8.7007(6) Å, c = 17.5854(15) Å, β = 110.474(11), V = 2132.5(3) Å^3, Z = 4, T = 295(2) K, μ(MoKα) = 0.277 mm^{-1}, D_{calc} = 1.332 g/cm^3, 14068 reflections measured (7.492° ≤ 2Θ ≤ 62.136°), 5660 unique (R_{int} = 0.0465, R_{sigma} = 0.0585). The final R_1 = 0.0584, wR_2 = 0.1508 (I > 2σ(I)) and R_1 = 0.1081, wR_2 = 0.2069 (all data). GooF = 1.027. Largest diff. peak/hole 0.31/-0.40 eÅ^{-3}.

The experiment was performed on an automatic four-circle X-ray diffractometer "Xcalibur 3" with a CCD detector according to the standard procedure (MoK_α irradiation, graphite monochromator, ω-scanning in 1° increments at T = 295(2) K). An empirical correction for absorption has been introduced. Using the Olex2 [12] software shell, the structure was solved using the SHELXT program and refined using the SHELXL [13] program with a full-matrix F^2 MNC for non-hydrogen atoms. The H-atoms in C–H bonds are placed in the calculated positions and refined in the "rider" model in the isotropic approximation.

CCDC 2401684 (**3aa**) contains the supplementary crystallographic data for this paper. These data can be obtained free of charge via http://www.ccdc.cam.ac.uk/conts/retrieving.html (or from the CCDC, 12 Union Road, Cambridge CB2 1EZ, UK; Fax: +44 1223 336033; E-mail: deposit@ccdc.cam.ac.uk).

2-((4-Fluorophenyl)sulfonyl)-5-morpholino-3-phenyl-2,3-dihydro-1,2-thiazole-4-carbonitrile (3ab) was obtained by method B from thioamide **1a** (50 mg, 0.19 mmol), 4-fluorobenzenesulfonamide (41 mg, 0.23 mmol), $PhI(OAc)_2$ (93 mg, 0.28 mmol) in DCM (2 mL), reaction time is 30 min. The crude product was chromatographed for SiO_2 (DCM/EtOAc, gradient 25:0 → 24.5:0.5). Yield 52 mg (63%); colorless powder; mp 163–165 °C; ^1H NMR ($CDCl_3$) δ (*J*, Hz) 8.07–8.04 (2H, m, H Ar), 7.48 (2H, d, *J* 7.0, H Ar), 7.42–7.32 (5H, m, H Ar), 6.12 (1H, s, $CH_{thiazol}$), 3.62–3.60 (4H, m, CH_2), 3.33–3.30 (4H, m, CH_2); ^{19}F NMR ($CDCl_3$) *δ* -101.49; ^{13}C NMR ($CDCl_3$) δ (*J*, Hz) 166.5 (d, *J* 258.4), 164.2, 135.8, 132.0 (d, *J* 9.5), 130.2 (d, *J* 3.2), 129.2, 128.9, 126.9, 116.4 (d, *J* 22.6), 116.4, 73.7, 68.8, 66.0, 51.2; HRMS–ESI-TOF (*m/z*): $[M + H]^+$ calcd. for $C_{20}H_{19}FN_3O_3S_2^+$, 432.0846; found: 432.0844.

2-((4-Methoxyphenyl)sulfonyl)-5-morpholino-3-phenyl-2,3-dihydro-1,2-thiazole-4-carbonitrile (**3ac**) was obtained by method B from thioamide **1a** (50 mg, 0.19 mmol), 4-methoxybenzenesulfonamide (43 mg, 0.23 mmol), $PhI(OAc)_2$ (93 mg, 0.22 mmol) in DCM (2 mL), reaction time is 30 min. The crude product was chromatographed for SiO_2 (DCM/EtOAc, gradient 25:0 → 24.3:0.7). Yield 58 mg (67%); colorless powder; mp 193–194 °C; ^1H NMR ($CDCl_3$) δ (*J*, Hz) 7.96 (2H, d, *J* 8.6, H Ar), 7.49 (2H, d, *J* 6.7, H Ar), 7.44–7.41 (3H, m, H Ar), 7.10 (2H, d, *J* 8.6, H Ar), 6.11 (1H, s, $CH_{thiazol}$), 3.93 (3H, s, OMe), 3.61–3.58 (4H, m, CH_2), 3.33–3.28 (4H, m, CH_2); ^{13}C NMR ($CDCl_3$) δ 164.6, 164.4, 136.2, 131.4, 129.0, 128.8, 127.0, 125.6, 116.7, 114.3, 73.7, 68.9, 66.1, 56.1, 51.2; HRMS–ESI-TOF (*m/z*): $[M + H]^+$ calcd. for $C_{21}H_{22}N_3O_4S_2^+$, 444.1046; found: 444.1050.

2-((4-Chlorophenyl)sulfonyl)-5-morpholino-3-phenyl-2,3-dihydro-1,2-thiazole-4-carbonitrile (3ad) was obtained by method B from thioamide **1a** (50 mg, 0.19 mmol), 4-chlorobenzenesulfonamide (44 mg, 0.23 mmol), $PhI(OAc)_2$ (93 mg, 0.22 mmol), DCM (2 mL), reaction time is 10 min. The crude product was chromatographed for SiO_2 (DCM). Yield 49 mg (57%); colorless powder; mp 185–187 °C; ^1H NMR ($CDCl_3$) δ (*J*, Hz) 7.97

(2H, d, *J* 8.4, H Ar), 7.63 (2H, d, *J* 8.4, H Ar), 7.48 (2H, d, *J* 6.5, H Ar), 7.42–7.35 (3H, m, H Ar), 6.12 (1H, s, $CH_{thiazol}$), 3.61–3.59 (4H, m, CH_2), 3.37–3.25 (4H, m, CH_2); ^{13}C NMR ($CDCl_3$) δ 164.0, 141.4, 135.7, 132.5, 130.6, 129.3, 129.2, 128.9, 126.9, 116.4, 73.7, 68.7, 66.0, 51.2; HRMS–ESI-TOF (*m/z*): $[M + H]^+$ calcd. for $C_{20}H_{18}ClN_3O_3S_2^+$, 448.0551; found: 448.0551.

5-Morpholino-2-((4-nitrophenyl)sulfonyl)-3-phenyl-2,3-dihydro-1,2-thiazole-4-carbonitrile (3ae) was obtained by method B from thioamide **1a** (50 mg, 0.19 mmol), 4-nitrobenzenesulfonamide (47 mg, 0.23 mmol), $PhI(OAc)_2$ (93 mg, 0.22 mmol), DCM (2 mL), reaction time is 15 min. The crude product was chromatographed for SiO_2 (DCM/EtOAc, gradient 25:0 → 24.6:0.4). Yield 36 mg (40%); pale yellow powder; mp 209–212 °C; ^{1}H NMR (DMSO-d_6) δ (*J*, Hz) 8.54 (2H, d, *J* 8.2, H Ar), 8.44 (2H, d, *J* 8.3, H Ar), 7.46 (5H, br. s, H Ar), 6.30 (1H, s, $CH_{thiazol}$), 3.55–3.44 (4H, m, CH_2), 3.30–3.25 (4H, m, CH_2); ^{13}C NMR (DMSO-d_6) δ 163.0, 151.1, 138.3, 136.0, 130.5, 129.0, 128.8, 126.9, 124.3, 115.9, 72.4, 66.7, 65.3, 50.7; HRMS–ESI-TOF (*m/z*): $[M + H]^+$ calcd. for $C_{20}H_{18}N_4O_5S_2^+$, 459.0791; found: 459.0794.

2-(Methylsulfonyl)-5-morpholino-3-phenyl-2,3-dihydro-1,2-thiazole-4-carbonitrile (3af) was obtained by method A from thioamide **1a** (70 mg, 0.27 mmol), PhINMs (**2f**, 121 mg, 0.41 mmol) in DCM (2 mL), reaction time is 10 min. The crude product was chromatographed for SiO_2 (DCM/EtOAc, gradient 25:0 → 22:3). Yield 88 mg (93%); colorless powder; mp 124–126 °C; ^{1}H NMR ($CDCl_3$) δ (*J*, Hz) 7.52–7.49 (2H, m, H Ar), 7.44–7.38 (3H, m, H Ar), 6.09 (1H, s, $CH_{thiazol}$), 3.80–3.78 (4H, m, CH_2), 3.64–3.62 (4H, m, CH_2), 3.05 (3H, m, Me); ^{13}C NMR ($CDCl_3$) δ 164.6, 136.3, 129.2, 129.0, 126.8, 116.7, 72.9, 68.2, 66.2, 51.3, 34.3; HRMS–ESI-TOF (*m/z*): $[M + H]^+$ calcd. for $C_{15}H_{17}N_3O_3S_2Na^+$, 374.0604; found: 374.0605.

3-Phenyl-5-(piperidine-1-yl)-2-tosyl-2,3-dihydro-1,2-thiazole-4-carbonitrile (3ba) was obtained by method A from 3-phenyl-2-(piperidin-1-carbonothioyl)acrylonitrile (**1b**, 26 mg, 0.1 mmol), PhINTs (**2a**, 56 mg, 0.15 mmol) in DCM (0.8 mL), reaction time is 20 min. The crude product was chromatographed for SiO_2 (PE/EtOAc, gradient 30:10 → 30:20). Yield 30 mg (70%); colorless powder; mp 152–153 °C; ^1H NMR ($CDCl_3$) δ (*J*, Hz) 7.92 (2H, d, *J* 8.2, H Ar), 7.52–7.50 (2H, m, H Ar), 7.43–7.32 (5H, m, H Ar), 6.11 (1H, s, $CH_{thiazol}$), 3.41–3.35 (2H, m, CH_2), 3.23–3.17 (2H, m, CH_2), 2.49 (3H, s, Me), 1.60–1.42 (6H, m, CH_2); ^{13}C NMR ($CDCl_3$) δ 163.9, 145.4, 136.9, 131.6, 129.6, 129.3, 128.9, 128.7, 127.1, 117.2, 73.8, 66.7, 53.0, 25.7, 23.8, 21.8; HRMS–ESI-TOF (*m/z*): $[M + H]^+$ calcd. for $C_{22}H_{24}N_3O_2S_2^+$, 426.1310; found: 426.1307.

3-Phenyl-5-(pyrrolidine-1-yl)-2-tosyl-2,3-dihydro-1,2-thiazole-4-carbonitrile (3ca) was obtained by method A from 3-phenyl-2-(pyrrolidin-1-carbonothioyl)acrylonitrile (**1c**, 73 mg, 0.3 mmol), PhINTs (**2a**, 168 mg, 0.45 mmol) in DCM (1.6 mL), reaction time is 20 min. The crude product was chromatographed for SiO_2 (PE/EtOAc, gradient 100:0 → 50:50). Yield 93 mg (76%); colorless powder; mp 166–167 °C; ^1H NMR ($CDCl_3$) δ (*J*, Hz) 7.92 (2H, d, *J* 8.4, H Ar), 7.54–7.52 (2H, m, H Ar), 7.41–7.32 (5H, m, H Ar), 6.06 (1H, s, $CH_{thiazol}$), 3.41–3.35 (2H, m, CH_2), 3.30–3.24 (2H, m, CH_2), 2.48 (3H, s, Me), 1.89–1.86 (4H, m, CH_2); ^{13}C NMR ($CDCl_3$) δ 160.7, 145.4, 136.8, 131.5, 129.4, 129.3, 128.8, 128.7, 127.1, 117.7, 73.4, 65.7, 51.6, 25.8, 21.8; HRMS–ESI-TOF (*m/z*): $[M + H]^+$ calcd. for $C_{21}H_{22}N_3O_2S_2^+$, 412.1153; found: 412.1148.

5-(Azepan-1-yl)-3-phenyl-2-tosyl-2,3-dihydro-1,2-thiazole-4-carbonitrile (3da) was obtained by method A from 2-(azepan-1-carbonothioyl)-3-phenylacrylonitrile (**1d**, 54 mg, 10.2 mmol), PhINTs (**2a**, 112 mg, 0.30 mmol) in DCM (1 mL), reaction time is 20 min. The crude product was chromatographed for SiO_2 (PE/DCM, gradient 20:10 → 10:15). Yield 85 mg (97%); colorless powder; mp 177–178 °C; ^1H NMR ($CDCl_3$) δ (*J*, Hz) 7.92 (2H, d, *J* 8.3, H Ar), 7.54–7.52 (2H, d, H Ar), 7.42–7.32 (5H, d, H Ar), 6.14 (1H, s, $CH_{thiazol}$),

3.51–3.45 (2H, m, CH_2), 3.25–3.18 (2H, m, CH_2), 2.47 (3H, s, Me), 1.77–1.63 (2H, m, CH_2), 1.59–1.44 (4H, m, CH_2), 1.41–1.31 (2H, m, CH_2); ^{13}C MNR ($CDCl_3$) δ 163.5, 145.3, 137.1, 131.8, 129.7, 129.3, 128.8, 128.7, 127.1, 117.7, 73.9, 64.4, 54.3, 28.9, 26.8, 21.8; HRMS–ESI-TOF (*m/z*): [M + H]$^+$ calcd. for $C_{23}H_{26}N_3O_2S_2^+$, 440.1466; found: 440.1463.

5-(Dimethylamino)-3-phenyl-2-tosyl-2,3-dihydro-1,2-thiazole-4-carbonitrile (3ea) was obtained by method A from 2-cyano-*N*,*N*-dimethyl-3-phenylprop-2-benzamide (**1e**, 22 mg, 0.1 mmol), PhINTs (**2a**, 56 mg, 0.15 mmol) in DCM (0.8 mL), reaction time is 20 min. The crude product was chromatographed for SiO_2 (PE/EtOAc, gradient 100:0 → 50:50). Yield 32 mg (83%); colorless powder; mp 185–186 °C; ^{1}H NMR ($CDCl_3$) δ (*J*, Hz) 7.92 (2H, d, *J* 8.3, H Ar), 7.53–7.50 (2H, m, H Ar), 7.44–7.34 (5H, m, H Ar), 6.08 (1H, s, $CH_{thiazol}$), 2.96 (6H, s, $N(Me)_2$), 2.50 (3H, s, $MeSO_2$); ^{13}C NMR ($CDCl_3$) δ 164.7, 145.6, 136.7, 131.5, 129.5, 129.2, 128.9, 128.8, 127.1, 117.2, 73.9, 67.4, 43.2, 21.9; HRMS–ESI-TOF (*m/z*): [M + H]$^+$ calcd. for $C_{19}H_{20}N_3O_2S_2^+$, 386.0997; found: 386.0995.

1-(5-Morpholino-3-phenyl-2-tosyl-2,3-dihydro-1,2-thiazole-4-yl)ethane-1-one (3fa) was obtained by method A from 3-(morpholino-4-carbonothioyl)-4-phenylbutyl-3-en-2-one (**1f**, 27.5 mg, 0.1 mmol), PhINTs (**2a**, 56 mg, 0.15 mmol) in DCM (0.8 mL), reaction time is 30 min. The crude product was chromatographed for SiO_2 (PE/EtOAc, 1:1). Yield 40 mg (91%); colorless powder; mp 125–126 °C; ^{1}H NMR ($CDCl_3$) δ (*J*, Hz): 7.81 (2H, d, *J* 8.3, H Ar), 7.49–7.45 (2H, m, H Ar), 7.39–7.34 (3H, m, H Ar), 7.29 (2H, d, *J* 8.3, H Ar), 6.36 (1H, s, HC=), 3.77–3.72 (2H, m, CH_2), 3.62–3.56 (2H, m, CH_2), 3.46–3.40 (2H, m, CH_2), 3.20–3.15 (2H, m, CH_2), 2.44 (3H, s, Me Tos), 1.84 (3H, s, <u>Me</u>CO); ^{13}C NMR ($CDCl_3$) δ 186.2, 167.0, 145.5, 137.4, 132.0, 129.4, 128.8, 128.7, 128.6, 127.5, 103.2, 74.9, 66.6, 53.6, 29.6, 21.8; HRMS–ESI-TOF (*m/z*): [M + H]$^+$ calcd. for $C_{22}H_{25}N_2O_4S_2^+$, 445.1256; found: 445.1253.

Methyl 5-morpholino-3-phenyl-2-tosyl-2,3-dihydro-1,2-thiazole-4-carboxylate (**3ga**) was obtained by method A from methyl 2-(morpholine-4-carbonothioyl)-3-phenylacrylate (**1g**, 29 mg, 0.1 mmol), PhINTs (**2a**, 61 mg, 0.15 mmol) in DCM (0.5 mL), reaction time is 30 min. The crude product was chromatographed for SiO_2 (PE/EtOAc, gradient 2:1 → 1:1). Yield 42 mg (91%); colorless powder; mp 175–176 °C; ^{1}H MNR ($CDCl_3$) δ (*J*, Hz) 7.81 (2H, d, *J* 8.0, H Ar), 7.46 (2H, d, *J* 6.9, H Ar), 7.38–7.30 (3H, m, H Ar), 7.27 (2H, d, *J* 8.0, H Ar), 6.44 (1H, s, HC=), 3.74–3.67 (2H, m, CH_2), 3.61–3.54 (2H, m, CH_2), 3.56 (3H, s, COOMe), 3.52–3.44 (2H, m, CH_2), 3.22–3.15 (2H, m, CH_2), 2.46 (3H, s, Me Tos); ^{13}C NMR ($CDCl_3$) δ 166.6, 161.4,145.2, 138.1, 131.9, 129.1, 128.7, 128.4, 128.4, 127.3, 91.9, 74.2, 66.8, 53.2, 51.0, 21.8; HRMS–ESI-TOF (*m/z*): $[M + H]^+$ calcd. for $C_{22}H_{25}N_2O_5S_2^+$, 461.1205; found: 461.1203.

Ethyl 5-morpholino-3-phenyl-2-tosyl-2,3-dihydro-1,2-thiazole-4-carboxylate (3ha) was obtained by method A from ethyl 2-(morpholine-4-carbonothioyl)-3-phenylacrylate (**1g**, 31 mg, 0.1 mmol), PhINTs (**2a**, 61 mg, 0.15 mmol) in DCM (0.5 mL), reaction time is 30 min. The crude product was chromatographed for SiO_2 (PE/EtOAc, gradient 2:1 → 1:1). Yield 38 mg (81%); colorless powder; mp 160–161 °C; ^{1}H NMR ($CDCl_3$) δ (*J*, Hz) 7.82 (2H, d, *J* 8.3, H Ar), 7.48–7.46 (2H, m, H Ar), 7.37–7.31 (3H, m, H Ar), 7.26 (2H, d, *J* 8.0, H Ar), 6.45 (1H, s, HC=), 4.10–3.92 (2H, m, CH_3CH_2O), 3.72–3.67 (2H, m, CH_2), 3.59–3.54 (2H, m, CH_2), 3.50–3.44 (2H, m, CH_2), 3.19–3.14 (2H, m, CH_2), 2.45 (3H, s, Me Tos), 1.21 (3H, t, *J* 7.1, CH_3CH_2O); ^{13}C NMR ($CDCl_3$) δ 166.2, 161.0, 145.1, 138.2, 132.0, 129.1, 128.8, 128.4, 128.3, 127.2, 92.7, 74.3, 66.8, 59.6, 53.1, 21.8, 14.8; HRMS–ESI-TOF (*m/z*): $[M + H]^+$ calcd. for $C_{23}H_{27}N_2O_5S_2^+$, 475.1361; found: 475.1363.

Morpholino(5-morpholino-3-phenyl-2-tosyl-2,3-dihydro-1,2-thiazole-4-yl)methanone (3ia) was obtained by method A from 2-(morpholine-4-carbonothioyl)-1-morpholino-3-phenylprop-2-ene-1-one (**1i**, 73 mg, 0.2 mmol), PhINTs (**2a**, 112 mg, 0.3 mmol) in DCM (1 mL), reaction time is 30 min. The crude product was chromatographed

for SiO_2 (PE/acetone, 35:10). Yield 70 mg (68%); colorless powder; mp 159–160 °C; ^{1}H NMR ($CDCl_3$) δ (*J*, Hz) 7.95 (2H, d, *J* 8.3, H Ar), 7.49–7.43 (2H, m, H Ar), 7.39–7.30 (5H, m, H Ar), 6.10 (1H, s, HC=), 3.66–3.57 (4H, m, CH_2), 3.48–3.34 (4H, m, CH_2), 3.33–3.15 (4H, m, CH_2), 3.03–2.87 (4H, m, CH_2), 2.45 (3H, s, Me); ^{13}C NMR ($CDCl_3$) δ 164.4, 152.5, 145.0, 137.7, 132.8, 129.6, 129.5, 128.7, 127.4, 100.6, 75.3, 66.9, 66.4, 51.4, 44.7, 21.8; HRMS–ESI-TOF (*m/z*): [M + H]$^+$ calcd. for $C_{25}H_{30}N_3O_5S_2^+$, 516.1627; found: 516.1623.

3-(3,4-Dimethoxyphenyl)-5-morpholino-2-tosyl-2,3-dihydro-1,2-thiazole-4-carbonitrile (3ja) was obtained by method A from 3-(3,4-dimethoxyphenyl)-2-(morpholine-4-carbonothioyl)acrylonitrile (**1j**, 50 mg, 0.16 mmol), PhINTs (**2a**, 88 mg, 0.23 mmol) in DCM (2.5 mL), reaction time is 20 min. The crude product was chromatographed for SiO_2 (DCM/EtOAc, gradient 24.5:0.5 → 24:1.0). Yield 58 mg (76%); colorless powder; mp 193–195 °C; ^{1}H NMR ($CDCl_3$) δ (*J*, Hz) 7.90 (2H, d, *J* 8.0, H Ar), 7.44 (2H, d, *J* 7.9, H Ar), 7.03 (1H, s, H Ar), 6.95 (1H, d, *J* 8.0, H Ar), 6.83 (1H, d, *J* 8.2 Hz, H Ar), 6.07 (1H, s, $CH_{thiazol}$), 3.91 (3H, s, OMe), 3.87 (3H, s, OMe), 3.58–3.56 (4H, m, CH_2), 3.33–3.25 (4H, m, CH_2), 2.51 (3H, s, Me); ^{13}C NMR ($CDCl_3$) δ 164.1, 149.8, 149.5, 145.7, 131.2, 129.6, 129.3, 128.3, 118.9, 116.6, 110.8, 110.6, 73.6, 69.1, 66.0, 56.1, 56.1, 51.2, 21.8; HRMS–ESI-TOF (*m/z*): [M + H]$^+$ calcd. for $C_{23}H_{26}N_3O_5S_2^+$, 488.1308; found: 488.1312.

5-Morpholino-3-(4-nitrophenyl)-2-tosyl-2,3-dihydro-1,2-thiazole-4-carbonitrile (3ka) was obtained by method A from 2-(morpholine-4-carbonothioyl)-3-(4-nitrophenyl)acrylonitrile (**1k**, 61 mg, 0.20 mmol), PhINTs (**2a**, 112 mg, 0.30 mmol) in DCM (1 mL), reaction time is 60 min. The crude product is chromatographed for SiO_2 (PE/EtOAc, 25:25). Yield 77 mg (81%); colorless powder; mp 168–169 °C. ^{1}H NMR (DMSO-d_6) δ (*J*, Hz) 8.30 (2H, d, *J* 8.7, H Ar), 8.01 (2H, d, *J* 8.2, H Ar), 7.73 (2H, d, *J* 8.7, H Ar), 7.58 (2H, d, *J* 8.2, H Ar), 6.37 (1H, s, $CH_{thiazol}$), 3.56–3.45 (4H, m, CH_2), 3.31–3.28 (4H, m, CH_2), 3.26 (3H, s, Me); ^{13}C NMR ($CDCl_3$) δ 164.5, 148.5, 146.2, 143.7, 131.0,

129.8, 129.3, 124.0, 116.3, 72.9, 67.9, 66.0, 51.4, 21.9. HRMS–ESI-TOF (*m/z*): $[M + H]^+$ calcd. for $C_{21}H_{21}N_4O_5S_2^+$, 473.0953; found: 473.0952.

5-Morpholino-3-(*p*-tolyl)-2-tosyl-2,3-dihydro-1,2-thiazole-4-carbonitrile (3la) was obtained by method A from 2-(morpholine-4-carbonothioyl)-3-(*p*-tolyl)acrylonitrile (**1l**, 50 mg, 0.18 mmol), PhINTs (**2a**, 103 mg, 0.27 mmol) in DCM (2 mL), reaction time is 10 min. The crude product was chromatographed for SiO_2 (PE/EtOAc, gradient 35:15 → 25:25). Yield 58 mg (72%); colorless powder; mp 193–195 °C; ^1H NMR ($CDCl_3$) δ (*J*, Hz) 7.91 (2H, d, *J* 8.0, H Ar), 7.44 (2H, d, *J* 8.0, H Ar), 7.37 (2H, d, *J* 7.9, H Ar), 7.19 (2H, d, *J* 7.8, H Ar), 6.10 (1H, s, $CH_{thiazol}$), 3.58–3.56 (4H, m, CH_2), 3.33–3.24 (4H, m, CH_2), 2.51 (3H, s, <u>Me</u>$C_6H_4SO_2$), 2.36 (3H, s, Me); ^{13}C NMR ($CDCl_3$) δ 164.2, 145.7, 139.0, 133.0, 131.3, 129.6, 129.5, 129.3, 126.9, 116.6, 73.6, 69.2, 66.0, 51.2, 21.8, 21.3; HRMS–ESI-TOF (*m/z*): $[M + H]^+$ calcd. for $C_{22}H_{24}N_3O_3S_2^+$, 442.1253; found: 442.1257.

3-(4-Methoxyphenyl)-5-morpholino-2-tosyl-2,3-dihydro-1,2-thiazole-4-carbonitrile (3ma) was obtained by method A from 3-(4-methoxyphenyl)-2-(morpholine-4-carbonothioyl)acrylonitrile (**1m**, 58 mg, 0.20 mmol), PhINTs (**2a**) (112 mg, 0.30 mmol) in DCM (4 mL), reaction time is 30 min. The crude product was chromatographed for SiO_2 (PE/DCM, 10:20; DCM/EtOAc 20:1). Yield 95 mg (96%); colorless powder; mp 214–215°C; ^1H NMR ($CDCl_3$) δ (*J*, Hz) 7.90 (2H, d, *J* 8.2, H Ar), 7.41 (2H, d, *J* 8.2, H Ar), 7.39 (2H, d, *J* 8.8, H Ar), 6.91 (2H, d, *J* 8.8, H Ar), 6.08 (1H, s, $CH_{thiazol}$), 3.81 (3H, s, OMe), 3.59–3.56 (4H, m, CH_2), 3.34–3.24 (4H, m, CH_2), 2.51 (3H, s, Me); ^{13}C NMR ($CDCl_3$) δ 164.1, 160.4, 145.6, 131.5, 129.6, 129.3, 128.4, 128.0, 116.5, 114.3, 73.6, 69.5, 66.1, 55.5, 51.3, 21.8; HRMS–ESI-TOF (*m/z*): $[M + H]^+$ calcd. for $C_{22}H_{24}N_3O_4S_2^+$, 458.1208; found: 458.1206.

3-(2-Fluorophenyl)-5-morpholino-2-tosyl-2,3-dihydro-1,2-thiazole-4-carbonitrile (3na) was obtained by method A from 3-(2-fluorophenyl)-2-(morpholine-4-

carbonothioyl)acrylonitrile (**1n**, 50 mg, 0.18 mmol), PhINTs (**2a**, 101 mg, 0.27 mmol) in DCM (2 mL), reaction time is 10 min. The crude product was chromatographed for SiO_2 (PE/EtOAc, gradient 35:15 → 25:25). Yield 66 mg (82%); colorless powder; mp 205–207 °C; ^1H NMR ($CDCl_3$) δ (*J*, Hz) 7.92 (2H, d, *J* 8.0, H Ar), 7.44 (2H, d, *J* 8.0, H Ar), 7.38–7.33 (2H, m, H Ar), 7.13 (2H, t, *J* 8.2, H Ar), 6.42 (1H, s, $CH_{thiazol}$), 3.58–3.56 (4H, m, CH_2), 3.33–3.23 (4H, m, CH_2), 2.50 (3H, s, Me); ^{19}F NMR ($CDCl_3$) *δ* -114.22; ^{13}C NMR (DMSO-d_6) δ (*J*, Hz) 163.7, 160.1 (d, *J* 249.5), 145.6, 131.4 (d, *J* 8.2), 129.9, 129.7, 129.1, 128.6 (d, *J* 2.8), 124.5 (d, *J* 3.5), 122.9 (d, *J* 13.3), 116.0 (d, *J* 20.8), 115.8, 67.6 (d, *J* 2.3), 65.6, 65.3, 50.7, 21.1; HRMS–ESI-TOF (*m/z*): $[M + H]^+$ calcd. for $C_{21}H_{21}FN_3O_3S_2^+$, 446.1003; found: 446.1007.

3-(4-(Dimethylamino)phenyl)-5-morpholino-2-tosyl-2,3-dihydro-1,2-thiazole-4-carbonitrile (3oa) was obtained by method A from 3-(4-(dimethylamino)phenyl)-2-(morpholine-4-carbonothioyl)acrylonitrile (**1o**, 50 mg, 0.16 mmol), PhINTs (**2a**, 103 mg, 0.25 mmol) in DCM (2 mL), reaction time is 6 min. The crude product was chromatographed for SiO_2 (DCM/EtOAc, gradient 25:0 → 24.2:0.8). Yield 57 mg (73%); pale yellow powder; mp 184–185 °C; ^1H NMR ($CDCl_3$) δ (*J*, Hz) 7.90 (2H, d, *J* 7.9, H Ar), 7.43 (2H, d, *J* 7.8, H Ar), 7.31 (2H, d, *J* 8.4, H Ar), 6.71 (2H, d, *J* 8.4, H Ar), 6.08 (1H, s, $CH_{thiazol}$), 3.58–3.56 (4H, m, CH_2), 3.33–3.23 (4H, m, CH_2), 2.96 (6H, s, N(<u>Me</u>)$_2$), 2.50 (3H, s, Me); ^{13}C NMR ($CDCl_3$) δ 164.0, 151.1, 145.5, 131.4, 129.5, 129.3, 128.1, 122.7, 116.6, 112.3, 73.9, 69.6, 66.1, 51.2, 40.5, 21.8; HRMS–ESI-TOF (*m/z*): $[M + H]^+$ calcd. for $C_{23}H_{27}N_4O_3S_2^+$, 471.1519; found: 471.1519.

3-(4-Chlorophenyl)-5-morpholino-2-tosyl-2,3-dihydro-1,2-thiazole-4-carbonitrile (3pa) was obtained by method A from 3-(4-chlorophenyl)-2-(morpholine-4-carbonothioyl)acrylonitrile (**1p**, 50 mg, 0.17 mmol), PhINTs (**2a**, 96 mg, 0.26 mmol) in DCM (2 mL), reaction time is 10 min. The crude product was chromatographed for SiO_2 (PE/EtOAc, gradient 15:35 → 25:25). Yield 55 mg (70%); colorless powder; mp 223–224

°C; ^{1}H NMR ($CDCl_3$) δ (*J*, Hz) 7.89 (2H, d, *J* 6.4, H Ar), 7.43–7.40 (4H, m, H Ar), 7.36–7.34 (2H, m, H Ar), 6.07 (1H, s, $CH_{thiazol}$), 3.57 (4H, br. s, CH_2), 3.33–3.24 (4H, m, CH_2), 2.51 (3H, s, Me); ^{13}C NMR ($CDCl_3$) δ 164.3, 145.9, 135.0, 134.7, 131.0, 129.7, 129.3, 129.0, 128.4, 116.4, 73.0, 68.4, 66.0, 51.2, 21.8; HRMS–ESI-TOF (*m/z*): $[M + H]^+$ calcd. for $C_{21}H_{21}ClN_3O_3S_2^+$, 462.0707; found: 462.0712.

3-(4-Bromophenyl)-5-morpholino-2-tosyl-2,3-dihydro-1,2-thiazole-4-carbonitrile (3qa) was obtained by method A from 3-(4-bromophenyl)-2-(morpholine-4-carbonothioyl)acrylonitrile (**1q**, 68 mg, 0.20 mmol), PhINTs (**2a**, 112 mg, 0.30 mmol) in DCM (1 mL), reaction time is 10 min. The crude product was chromatographed for SiO_2 (PE/DCM, gradient 20:10 → 10:20). Yield 81 mg (80%); colorless powder; mp 220–221 °C; ^{1}H NMR ($CDCl_3$) δ (*J*, Hz) 7.90 (2H, d, *J* 8.3, H Ar), 7.52 (2H, d, *J* 8.5, H Ar), 7.44 (2H, d, *J* 8.3, H Ar), 7.36 (2H, d, *J* 8.5, H Ar), 6.05 (1H, s, $CH_{thiazol}$), 3.63–3.55 (4H, m, CH_2), 3.34–3.24 (4H, m, CH_2), 2.51 (3H, s, Me); ^{13}C NMR ($CDCl_3$) δ 164.4, 145.9, 135.4, 132.0, 131.2, 129.7, 129.3, 128.7, 123.2, 116.4, 73.2, 68.6, 66.0, 51.3, 21.8; HRMS–ESI-TOF (*m/z*): $[M + H]^+$ calcd. for $C_{21}H_{21}BrN_3O_3S_2^+$, 506.0208; found: 506.0201.

Scaling up the synthesis of 2,3-dihydro-1,2-thiazole-4-carbonitrile 3qa. To the suspension of PhINTs (**2a**, 1660 mg, 4.448 mmol) in DCM (22 mL) at 0 ° C, a solution of thioamide **1q** (1000 mg, 2.965 mmol) in DCM (15 mL) was added dropwise over 10 min and stirred at 0 °C for 30 min, then the temperature of the bath was brought to room temperature and stirred for 18 h more. The resulting solution was evaporated in vacuum and product **3qa** was isolated on a column with SiO_2 (gradient PE/DCM 1:1 → DCM). The fractions containing the product were combined and the solvent was evaporated to dryness. The yield is 1270 mg (85%), colorless powder.

5-Morpholino-3-styryl-2-tosyl-2,3-dihydro-1,2-thiazole-4-carbonitrile (3ra) was obtained by method A from 2-(morpholine-4-carbonothioyl)-5-phenylpent-2,4-dienenitrile

(**1r**, 202 mg, 0.71 mmol), PhINTs (**2a**, 398 mg, 1.06 mmol) in DCM (6.5 mL), reaction time is 10 min. The crude product was chromatographed for SiO_2 (DCM/EtOAc, gradient 25:0 → 24.8:0.2). Yield 252 mg (78%); colorless powder; mp 178–180 °C; ^{1}H NMR ($CDCl_3$) δ (*J*, Hz) 7.88 (2H, d, *J* = 8.1 Hz, H Ar), 7.43–7.40 (4H, m, H Ar), 7.34–7.29 (3H, m, H Ar), 6.72 (1H, d, *J* = 15.8 Hz, PhC<u>H</u>=CH), 6.20 (1H, dd, *J* = 15.8, 5.6 Hz, PhCH=C<u>H</u>), 5.65 (1H, d, *J* = 5.5 Hz, $CH_{thiazol}$), 3.57–3.55 (4H, m, CH_2), 3.26–3.23 (4H, m, CH_2), 2.50 (3H, s, Me); ^{13}C NMR ($CDCl_3$) δ 163.2, 145.7, 135.8, 132.9, 131.2, 129.6, 129.3, 128.8, 128.5, 127.1, 122.6, 116.3, 73.0, 69.0, 66.0, 51.1, 21.9; HRMS–ESI-TOF (*m/z*): [M + H]$^+$ calcd. for $C_{23}H_{24}N_3O_3S_2^+$, 454.1253; found: 454.1255.

5-Morpholino-3-(quinoline-8-yl)-2-tosyl-2,3-dihydro-1,2-thiazole-4-carbonitrile (3sa) was obtained by method A from 2-(morpholine-4-carbonothioyl)-3-(quinoline-8-yl)acrylonitrile (**1s**, 50 mg, 0.16 mmol), PhINTs (**2a**, 121 mg, 0.32 mmol) in DCM (2.5 mL), reaction time is 34 min. The crude product was chromatographed for SiO_2 (PE/EtOAc, gradient 25:0 → 20:30). Yield 40 mg (77%); colorless powder; mp 165–166 °C; ^{1}H NMR (DMSO-*d*$_6$) δ (*J*, Hz): 9.03–9.01 (1H, m, H Ar), 8.44 (1H, d, *J* = 8.1 Hz, H Ar), 8.05 (1H, d, *J* = 8.1 Hz, H Ar), 7.99 (3H, d, *J* = 7.4 Hz, H Ar), 7.68–7.61 (4H, m, H Ar), 7.51 (1H, s, $CH_{thiazol}$), 3.54–3.45 (4H, m, CH_2), 3.25 (4H, br. s, CH_2), 2.50 (3H, s, Me); ^{13}C NMR ($CDCl_3$) δ 162.8, 150.2, 145.5, 144.3, 136.4, 134.6, 130.1, 129.7, 129.1, 129.0, 128.00, 127.1, 126.4, 121.9, 116.1, 69.3, 67.8, 65.3, 50.7, 21.2; HRMS–ESI-TOF (*m/z*): [M + H]$^+$ calcd. for $C_{24}H_{23}N_4O_3S_2^+$, 479.1206; found: 479.1205.

5-Morpholino-3-(pyridine-3-yl)-2-tosyl-2,3-dihydro-1,2-thiazole-4-carbonitrile (3ta) was obtained by method A from 2-(morpholine-4-carbonothioyl)-3-(pyridine-3-yl)acrylonitrile (**1t**, 52 mg, 0.20 mmol), PhINTs (**2a**, 112 mg, 0.30 mmol) in DCM (1 mL), reaction time is 35 min. The crude product was chromatographed for SiO_2 (EtOAc). Yield 61 mg (71%); colorless powder mp 192–193 °C; ^{1}H NMR ($CDCl_3$) (*J*, Hz) 8.70 (1H, d, *J* 1.8, H Ar), 8.61 (1H, d, *J* 4.7, H Ar), 7.91 (2H, d, *J* 8.1, H Ar), 7.82 (1H, d, *J* 8.4, H Ar),

7.45 (2H, d, *J* 8.1, H Ar), 7.32 (1H, dd, *J* 8.0, 4.8, H Ar), 6.12 (1H, s, $CH_{thiazol}$), 3.59–3.57 (4H, m, CH_2), 3.35–3.25 (4H, m, CH_2), 2.52 (3H, s, Me); ^{13}C NMR ($CDCl_3$) δ 164.5, 150.4, 148.3, 146.0, 135.0, 132.0, 131.2, 129.8, 129.3, 123.6, 116.1, 72.0, 67.9, 66.0, 51.4, 21.9; HRMS–ESI-TOF (*m/z*): $[M + H]^+$ calcd. for $C_{20}H_{21}N_4O_3S_2^+$, 429.1055; found: 429.1051.

3-(Furan-2-yl)-5-morpholino-2-tosyl-2,3-dihydro-1,2-thiazole-4-carbonitrile (3ua) was obtained by method A from 3-(furan-2-yl)-2-(morpholine-4-carbonothioyl)acrylonitrile (**1u**, 50 mg, 0.20 mmol), PhINTs (**2a**, 112 mg, 0.30 mmol) in DCM (1 mL), reaction time is 35 min. The crude product was chromatographed for SiO_2 (PE/DCM, gradient 20:10 → 10:20). Yield 64 mg (74%); colorless powder; mp 140–141 °C; ^{1}H NMR ($CDCl_3$) δ (*J*, Hz) 7.88 (2H, d, *J* 8.2, H Ar), 7.46 (1H, d, J 8.0, H Ar), 7.42 (2H, d, *J* 8.2, H Ar), 6.39 (1H, d, *J* 3.3, H Fur), 6.36–6.34 (1H, m, H Fur), 6.13 (1H, s, $CH_{thiazol}$), 3.58–3.56 (4H, m, CH_2), 3.33–3.23 (4H, m, CH_2), 2.50 (3H, s, Me); ^{13}C NMR ($CDCl_3$) δ 164.4, 148.6, 145.8, 144.1, 131.3, 129.6, 129.4, 115.9, 110.6, 109.6, 68.4, 67.5, 66.0, 51.2, 21.8; HRMS–ESI-TOF (*m/z*): $[M + H]^+$ calcd. for $C_{19}H_{20}N_3O_4S_2^+$, 418.0895; found: 418.0892.

5-Morpholino-3-(5-(4-nitrophenyl)furan-2-yl)-2-tosyl-2,3-dihydro-1,2-thiazole-4-carbonitrile (3va) was obtained by method A from 2-(morpholine-4-carbonothioyl)-3-(5-(4-nitrophenyl)furan-2-yl)acrylonitrile (**1v**, 100 mg, 0.27 mmol), PhINTs (**2a**, 151 mg, 0.41 mmol), $[Cu(MeCN)_4]PF_6$ (5 mol%, 7.6 mg) in DCM (4 mL), reaction time is 15 min. The crude product was chromatographed for SiO_2 (PE/EtOAc, gradient 25:0 → 20:30). Yield 98 mg (67%); yellow powder; mp 179–180 °C; ^{1}H NMR ($CDCl_3$) δ (*J*, Hz) 8.23 (2H, d, *J* 8.7, H Ar), 7.90 (2H, d, *J* 8.1, H Ar), 7.78 (2H, d, *J* 8.7, H Ar), 7.45 (2H, d, *J* 7.9, H Ar), 6.82 (1H, d, *J* 3.2, H Fur), 6.54 (1H, d, *J* 3.0, H Fur), 6.20 (1H, s, $CH_{thiazol}$), 3.59–3.57 (4H, s, CH_2), 3.35–3.25 (4H, m, CH_2), 2.52 (3H, s, Me); ^{13}C NMR ($CDCl_3$) δ 164.4, 153.0, 150.3, 146.8, 146.0, 135.9, 130.9, 129.7, 129.4, 124.4, 124.3, 115.9, 111.9, 109.6, 68.3, 66.69, 65.9, 51.2, 21.9; HRMS–ESI-TOF (*m/z*): $[M + H]^+$ calcd. for $C_{25}H_{23}N_4O_6S_2^+$, 539.1053; found: 539.1061.

3-(5-(4-Methoxy-2-nitrophenyl)furan-2-yl)-5-morpholino-2-tosyl-2,3-dihydro-1,2-thiazole-4-carbonitrile (3wa) was obtained by method A from 3-(5-(4-methoxy-2-nitrophenyl)furan-2-yl)-2-(morpholine-4-carbonothioyl)acrylonitrile (**1w**, 200 mg, 0.50 mmol), PhINTs (**2a**) (280 mg, 0.75 mmol) in DCM (5 mL), reaction time is 33 min. The crude product was chromatographed for SiO_2 (DCM/EtOAc, gradient 25:0 → 23.5:1.5). Yield 232 mg (91%); yellow powder; mp 184–185 °C; ^{1}H MNR (DMSO-d_6) δ (*J*, Hz) 7.95 (2H, d, *J* 8.3, H Ar), 7.73 (1H, d, *J* 8.7, H Ar), 7.54 (2H, d, *J* 8.1, H Ar), 7.49 (1H, d, *J* 2.6, H Ar), 7.32 (1H, dd, *J* 8.8, 2.6, H Ar), 6.75 (1H, d, *J* 3.4, H Fur), 6.67 (1H, d, *J* 3.4, H Fur), 6.21 (1H, s, $CH_{thiazol}$), 3.87 (3H, s, OMe), 3.57–3.47 (4H, m, CH_2), 3.29–3.26 (4H, m, CH_2), 2.47 (3H, s, Me); ^{13}C NMR (DMSO-d_6) δ 163.2, 159.5, 149.7, 149.1, 147.9, 145.6, 130.0, 129.9, 129.6, 129.0, 118.5, 115.6, 114.7, 111.6, 109.0, 109.0, 66.9, 65.2, 65.1, 56.2, 50.7, 21.1; HRMS–ESI-TOF (*m/z*): [M + H]$^+$ calcd. for $C_{26}H_{25}N_4O_7S_2^+$, 569.1159; found: 569.1164.

3-(5-(3,4-Dichlorophenyl)furan-2-yl)-5-morpholino-2-tosyl-2,3-dihydro-1,2-thiazole-4-carbonitrile (3xa) was obtained by method A from 3-(5-(3,4-dichlorophenyl)furan-2-yl)-2-(morpholine-4-carbonothioyl)acrylonitrile (**1x**, 200 mg, 0.51 mmol), PhINTs (**2a**, 284 mg, 0.76 mmol) in DCM (5 mL), reaction time is 18 min. The crude product is chromatographed for SiO_2 (PE/EtOAc, gradient 25:0 → 20:30). Yield 232 mg (91%); pale yellow powder; mp 178–179 °C; ^{1}H NMR ($CDCl_3$) δ (*J*, Hz) 7.90 (2H, d, *J* 8.1, H Ar), 7.71 (1H, s, H Ar), 7.50–7.43 (4H, m, H Ar), 6.62 (1H, d, *J* 3.3, H Fur), 6.48 (1H, d, *J* 3.3, H Fur), 6.17 (1H, s, $CH_{thiazol}$), 3.60–3.58 (4H, m, CH_2), 3.36–3.25 (4H, m, CH_2), 2.52 (3H, s, Me); ^{13}C NMR (DMSO-d_6) δ 164.5, 153.1, 148.8, 146.0, 133.1, 131.7, 131.1, 130.9, 130.3, 129.7, 129.4, 125.7, 123.3, 116.0, 111.8, 107.2, 68.4, 67.0, 66.0, 51.3, 21.9; HRMS–ESI-TOF (*m/z*): [M + H]$^+$ calcd. for $C_{25}H_{22}Cl_2N_3O_4S_2^+$, 562.0423; found: 562.0425.

3-Isopropyl-5-morpholino-2-tosyl-2,3-dihydro-1,2-thiazole-4-carbonitrile (3ya) was obtained from 4-methyl-2-(morpholine-4-carbonothioyl)pent-2-enonitrile (**1y**, 34 mg, 0.15

mmol), TsNIPh (**2a**, 84 mg, 0.225 mmol) in DCM (1.5 mL), reaction time is 1 h. The crude product was chromatographed for SiO_2 (PE/EtOAc, gradient 2:1 → 1:1). Yield 51 mg (87%); colorless powder; mp 91–92 °C; ^{1}H NMR ($CDCl_3$) δ (*J*, Hz) 7.82 (2H, d, *J* 8.3, H Ar), 7.39 (2H, d, *J* 8.3, H Ar), 4.58 (1H, d, *J* 8.4, $CH_{thiazol}$), 3.55–3.53 (4H, m, CH_2), 3.28–3.15 (4H, m, CH_2), 2.48 (3H, s, Me-Tos), 2.03 (1H, m, $(Me)_2C\underline{H}$), 1.11 (3H, d, *J* 6.7, $\underline{(Me)}_2CH$), 1.07 (3H, d, *J* 6.7, $\underline{(Me)}_2CH$); ^{13}C NMR (DMSO-d_6) δ 163.8, 145.4, 131.4, 129.5, 129.2, 117.0, 78.4, 69.8, 66.1, 51.2, 32.5, 21.8, 19.6, 18.9; HRMS−ESI-TOF (*m/z*): $[M + H]^+$ calcd. for $C_{18}H_{24}N_3O_3S_2^+$, 394.1254; found: 394.1253.

3-Cyclohexyl-5-morpholino-2-tosyl-2,3-dihydro-1,2-thiazole-4-carbonitrile (3za) was obtained from 3-cyclohexyl-2-(morpholine-4-carbonothioyl)acrylonitrile (**1z**, 58 mg, 0.219 mmol), TsNIPh (**2a**, 123 mg, 0.329 mmol) in DCM (1.7 mL), reaction time is 15 min. The crude product was chromatographed for SiO_2 (PE/EtOAc, gradient 5:1 → 4:1). Yield 73 mg (77%); colorless powder; mp 157–158 ºC; ^{1}H NMR ($CDCl_3$) δ (*J*, Hz) 7.81 (2H, d, *J* 8.2, H Ar), 7.38 (2H, d, *J* 8.2, H Ar), 4.65 (1H, d, *J* 8.4 Hz, $CH_{thiazol}$), 3.56–3.51 (4H, m, CH_2), 3.28–3.15 (4H, m, CH_2), 2.47 (3H, s, Me), 2.07–2.01 (1H, m, CH Cyclohex), 1.85–1.62 (5H, m, CH_2 Cyclohex), 1.28–1.12 (5H, m, CH_2 Cyclohex); ^{13}C NMR ($CDCl_3$) δ 163.7, 145.4, 131.4, 129.4, 129.2, 117.0, 77.4, 69.4, 66.1, 51.2, 41.3, 29.9, 29.4, 26.3, 26.0, 25.7, 21.8; HRMS–ESI-TOF (*m/z*): $[M + H]^+$ calcd. for $C_{21}H_{28}N_3O_3S_2^+$, 434.1567; found: 434.1568.

Optimization study for the reaction of thioamide 1a with PhINTs (2a) using chiral catalysts or ligands

Table S1. Optimization of the synthesis of sulfonylisothiazolines **3aa** using chiral catalysts or ligands

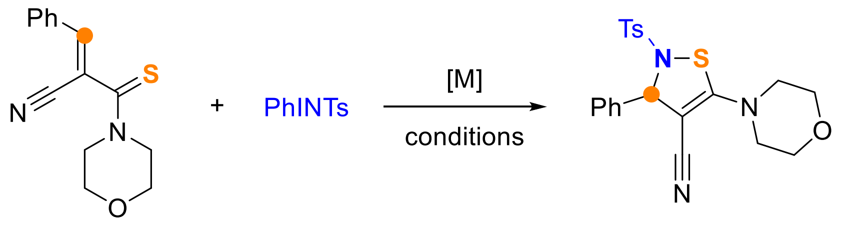

1a **2a** **3aa**

№	[M] (mol %)	T, °C	Equiv. of **2a**	Solvent (mL)	Time (h)	Yield, %	Ee, %
1	$[Cu(MeCN)_4]OTf$ (5.0) + (*R*)-DM-BINAP (10)	50	1.3	$CHCl_3$ (1.5)	16	73	1.4
2	$[Cu(MeCN)_4]OTf$ (5.0) + (*R*)-SEGPHOS (10)	50	1.3	$CHCl_3$ (1.5)	16	86	2.9
3	$[Cu(MeCN)_4]OTf$ (5.0) + (*R*)-DTBM-SEGPHOS (10)	50	1.3	$CHCl_3$ (1.5)	16	91	2.9
4	Rh_2(*S*-BSP)$_4$ (1.0)	50	1.3	$CHCl_3$ (1.5)	16	73	6.9
5	Rh_2(*S*-PTTL)$_4$ (1.0)	50	1.3	$CHCl_3$ (1.5)	16	65	5.2
6	Rh_2(*S*-BSP)$_4$ (1.0)	0 to rt	1.3	DCM (2.5)	50 min	60	racemic
7	Rh_2(*S*-PTTL)$_4$ (1.0)	0 to rt	1.3	DCM (2.5)	50 min	76	racemic
8	Rh_2(*S*-PTTL)$_4$ (1.0)	-35	1.3	DCM (2.5)	180 min	80	racemic
9	Rh_2(*S*-NTTL)$_4$ (1.0)	0 to rt	1.3	DCM (2.5)	50 min	78	racemic
10	Rh_2(*S*-PTAD)$_4$ (1.0)	0 to rt	1.3	DCM (2.5)	50 min	82	racemic
11	Rh_2(*R*-p-Ph-TPCP)$_4$ (1.0)	0 to rt	1.3	DCM (2.5)	50 min	78	racemic
12	$[Cu(MeCN)_4]OTf$ (5.0) + (*R*)-PyBOX (10)	0 to rt	1.3	DCM (2.5)	50 min	88	racemic

Conditions: thioamide **1a** (0.12 mmol), PhINTs (**2a**) (0.17 mmol). Isolated yields.
