# Supplementary material for: Reactions of acryl thioamides with iminoiodinanes as a one-step synthesis of N-sulfonyl-2,3-dihydro-1,2-thiazoles
Source: Beilstein J Org Chem. 2025 Jul 10;21:1397–403. doi: 10.3762/bjoc.21.104 (PMC12256786; doi:10.3762/bjoc.21.104)
Supplement: File 2 — Copies of NMR and HRMS spectra of all new compounds. [file Beilstein_J_Org_Chem-21-1397-s002.pdf]

BEILSTEIN JOURNAL OF ORGANIC CHEMISTRY

Supporting Information

for

Reactions of acryl thioamides with iminoiodinanes as a one-step synthesis of *N*-sulfonyl-2,3-dihydro-1,2-thiazoles

Vladimir G. Ilkin, Pavel S. Silaichev, Valeriy O. Filimonov, Tetyana V. Beryozkina, Margarita D. Likhacheva, Pavel A. Slepukhin, Wim Dehaen and Vasiliy A. Bakulev

Beilstein J. Org. Chem. **2025,** *21,* 1397–1403. doi:10.3762/bjoc.21.104

Copies of NMR and HRMS spectra of all new compounds

License and Terms: This is a supporting information file under the terms of the Creative Commons Attribution License (https://creativecommons.org/licenses/by/4.0). Please note that the reuse, redistribution and reproduction in particular requires that the author(s) and source are credited and that individual graphics may be subject to special legal provisions.
The license is subject to the *Beilstein Journal of Organic Chemistry* terms and conditions: (https://www.beilstein-journals.org/bjoc/terms)

[1] H NMR (CDCl$_3$) spectrum of **1h**

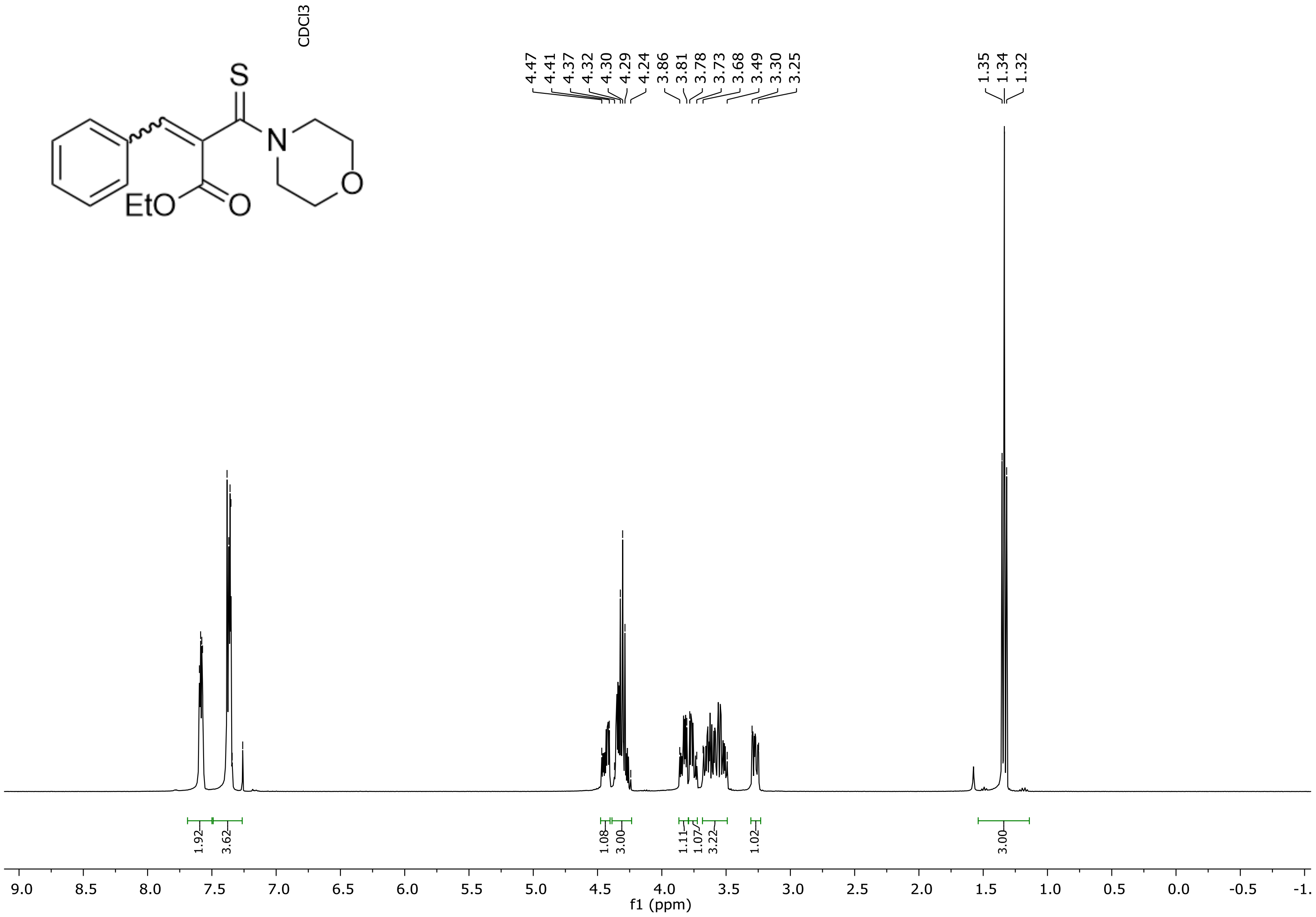

[13] C NMR ($CDCl_3$) spectrum of **1h**

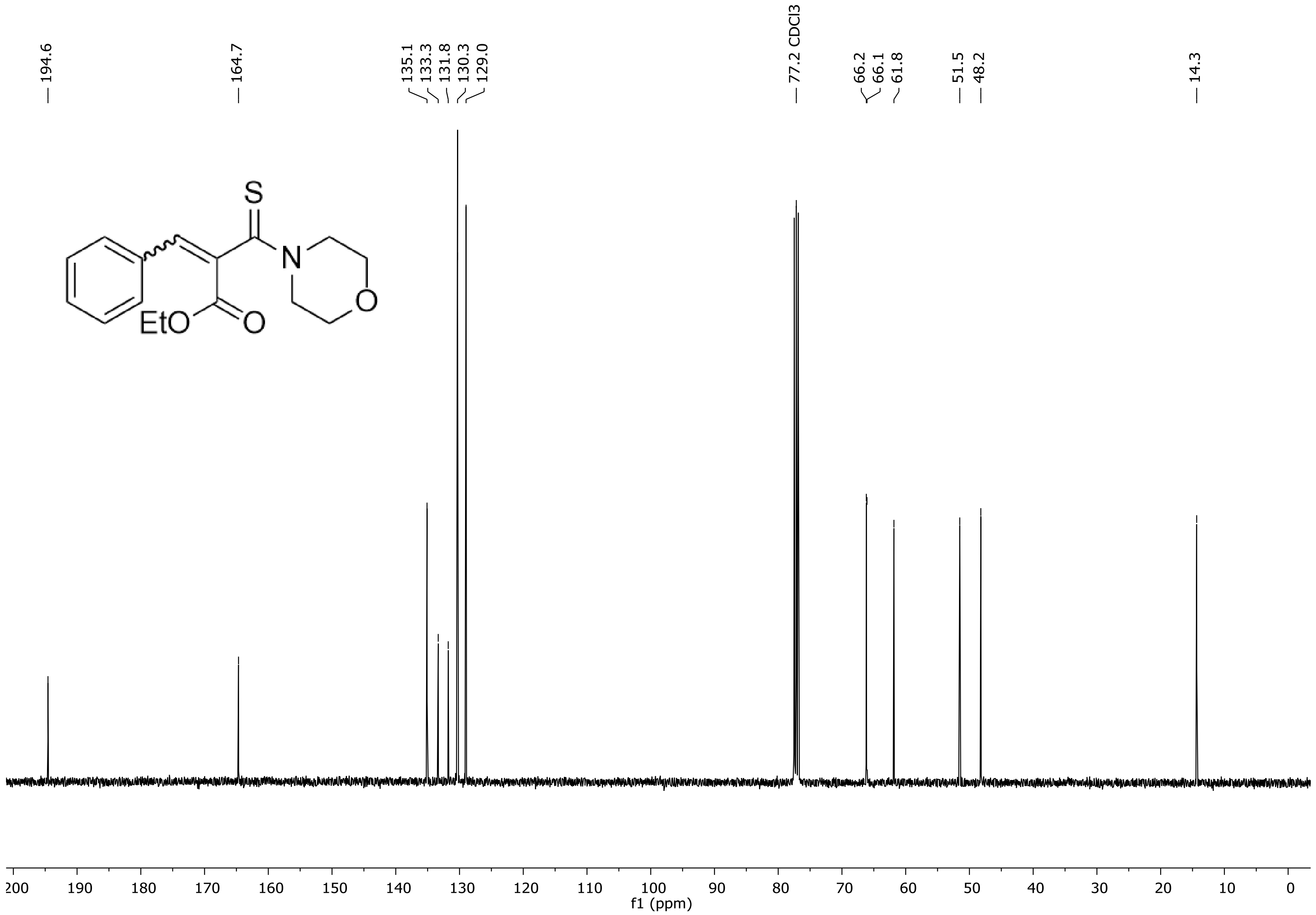

HRMS of **1h**

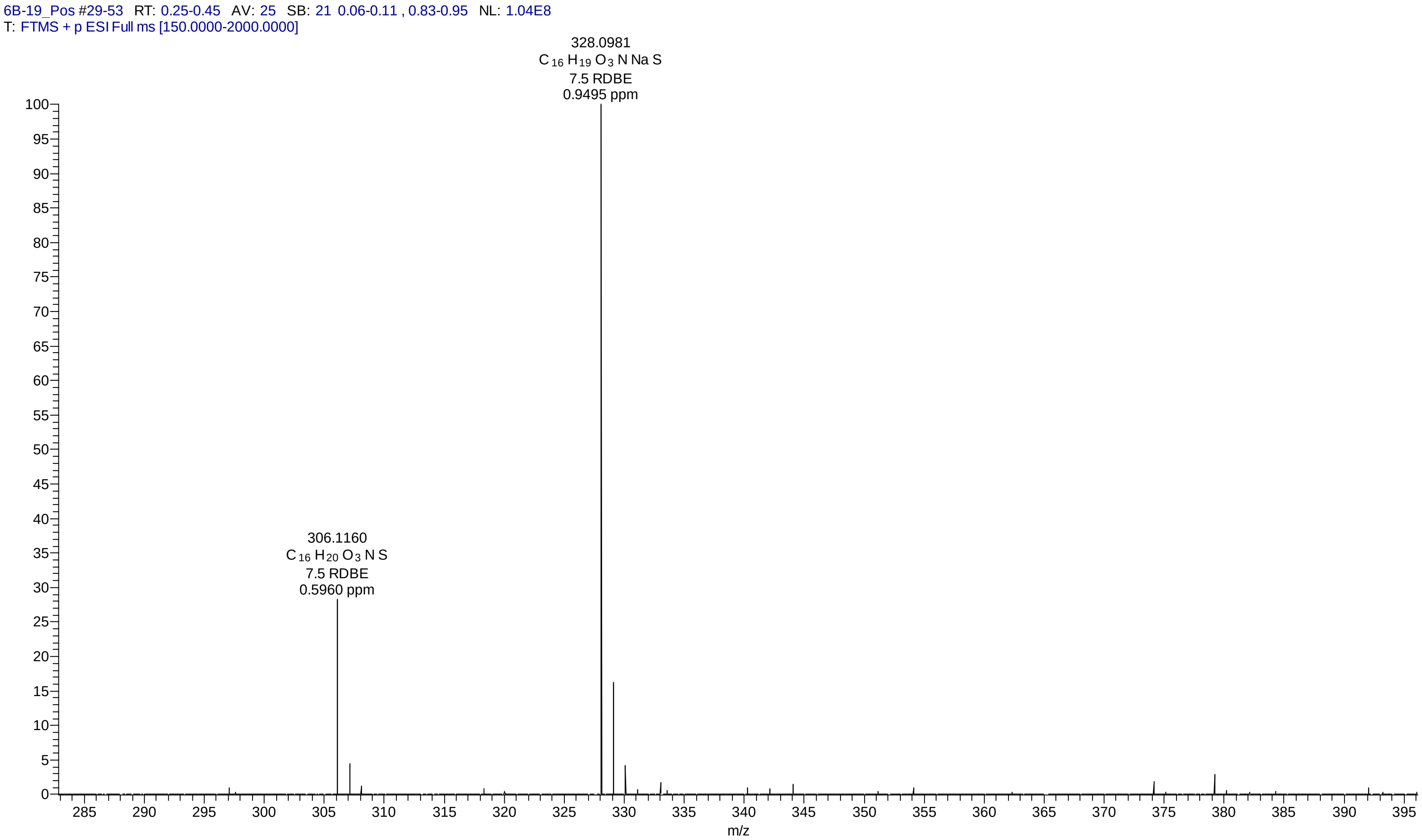

6B-19_Pos #29-53 RT: 0.25-0.45 AV: 25 SB: 21 0.06-0.11 , 0.83-0.95 NL: 1.04E8
T: FTMS + p ESI Full ms [150.0000-2000.0000]
328.0981
C 16 H19 O3 N Na S
7.5 RDBE
0.9495 ppm
306.1160
C 16 H20 O3 N S
7.5 RDBE
0.5960 ppm
m/z

[1] H NMR ($CDCl_3$) spectrum of **1i**

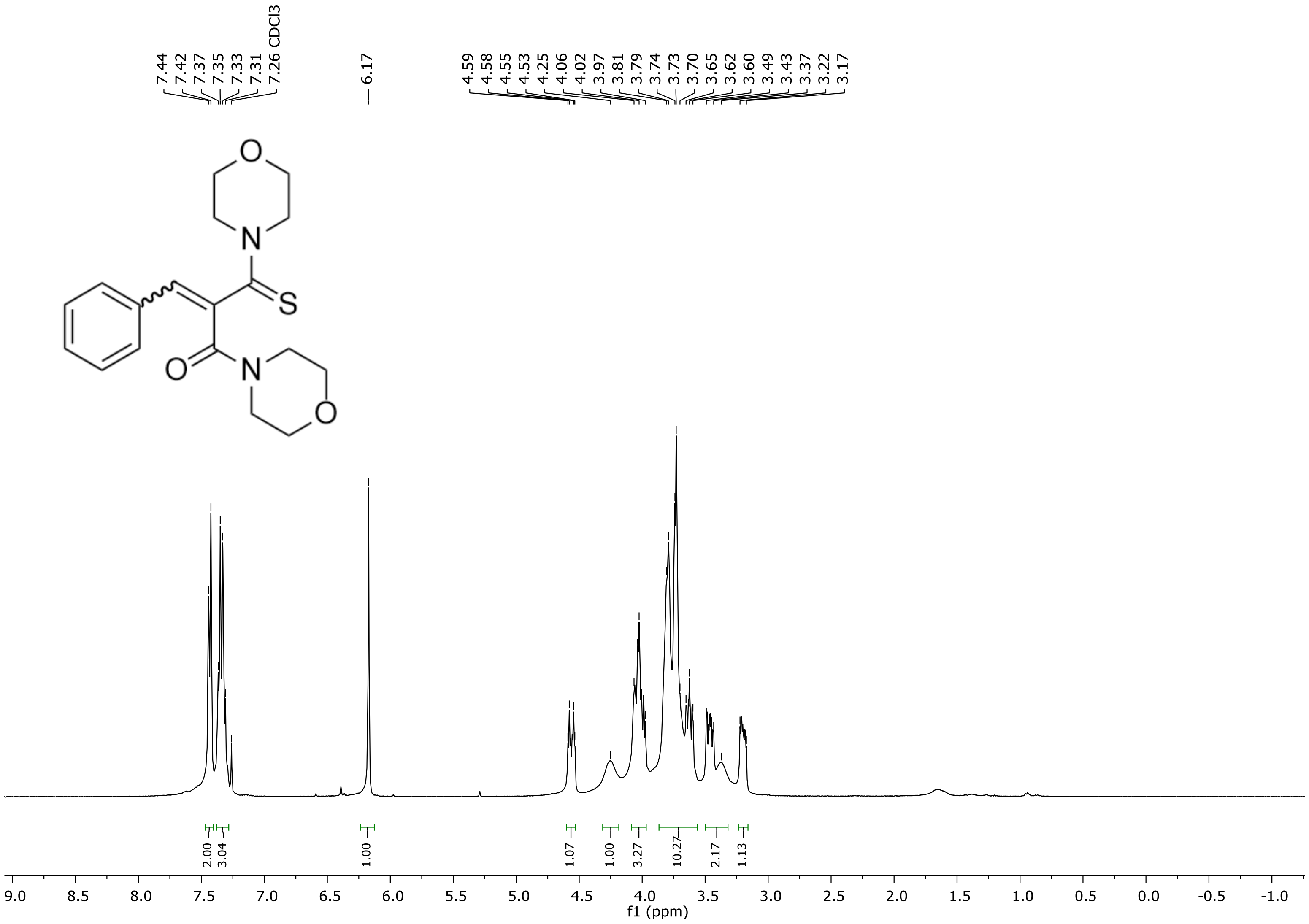

^{13}C NMR ($CDCl_3$) spectrum of **1i**

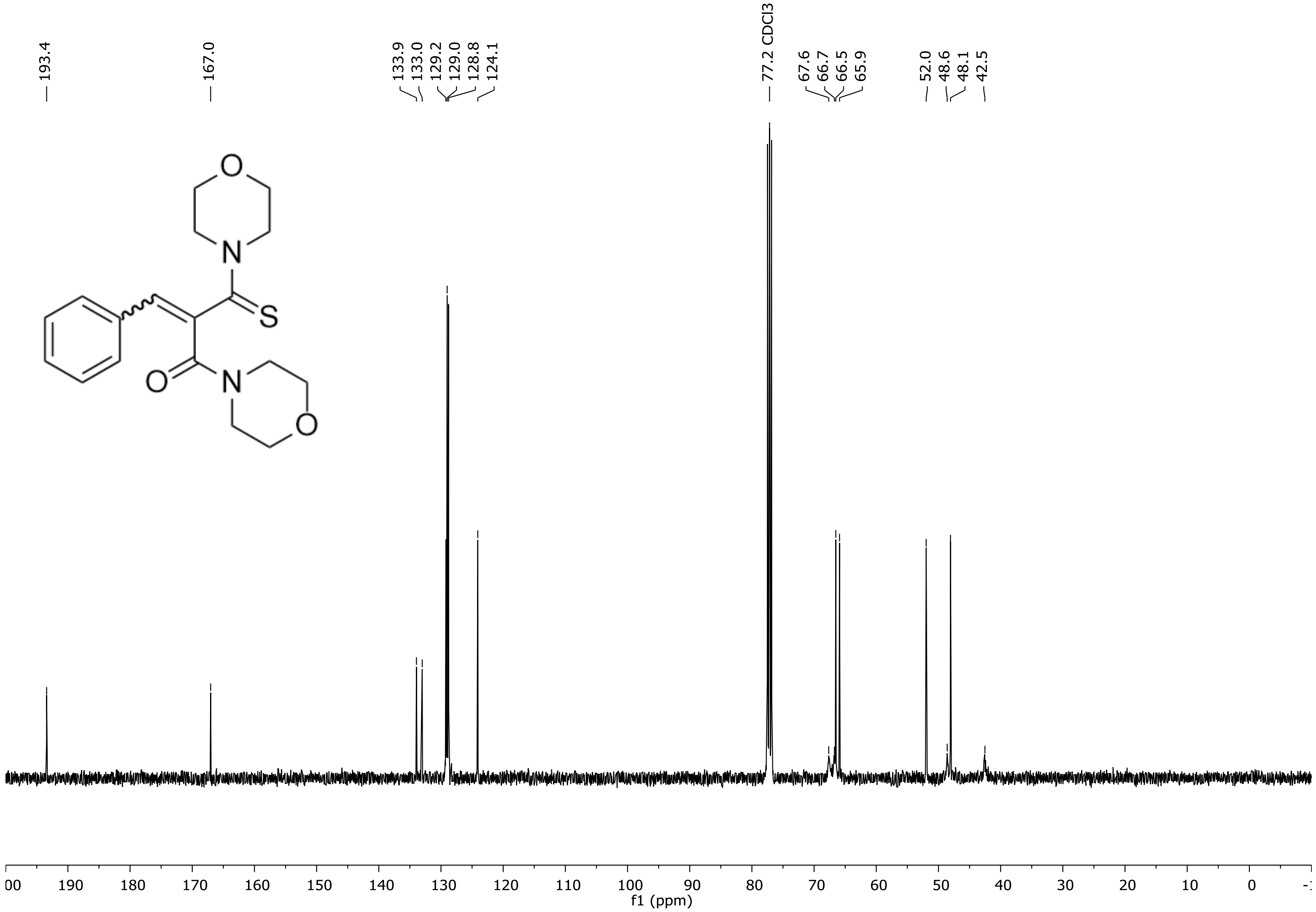

HRMS of **1i**

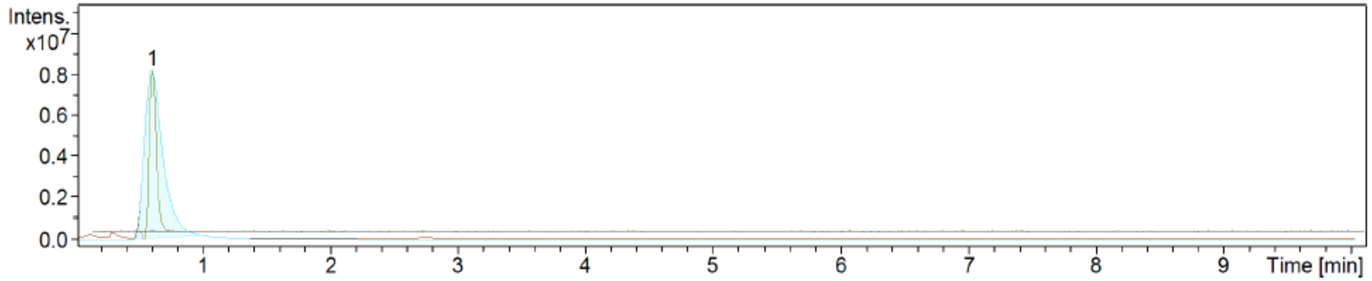

#	RT [min]	Area	Int. Type	I	S/N	Chromatogram	Max. m/z	FWHM [min]
1	0.6	80415208	Manual	8167088	20407.5	EIC 369.1246±0.02 +All MS	369.1246	

Cmpd 1, 0.6 min

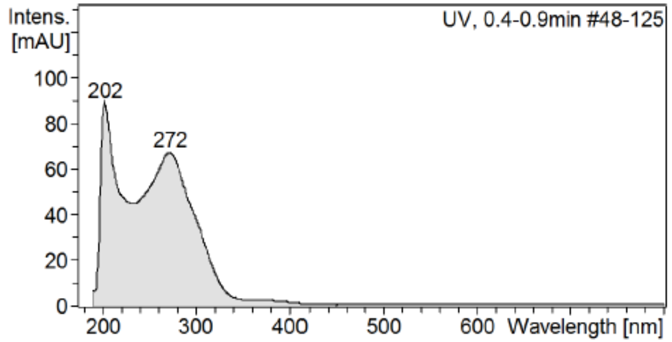

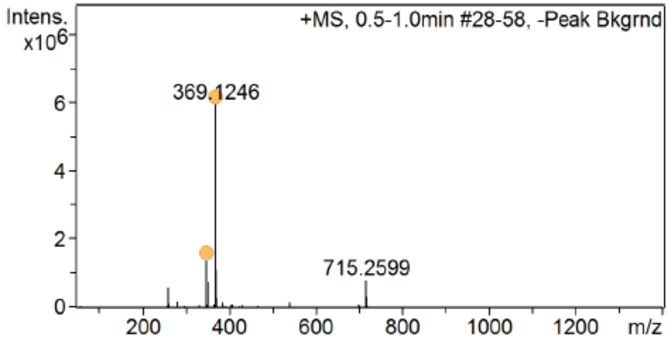

#	Wavelength	Intensity
0	202	88.5
1	272	67.2

#	m/z	Res.	S/N	I	I %	FWHM
1	260.0741	25358	180831264.0	566339	9.6	0.0103
2	280.1306	21792	48452780.0	151748	2.6	0.0129
3	347.1425	30459	432144384.0	1353419	22.9	0.0114
4	348.1453	22287	86193560.0	269947	4.6	0.0156
5	353.1474	32516	235000608.0	735991	12.4	0.0109
6	369.1246	39171	1890813184.0	5921775	100.0	0.0094
7	370.1274	30763	345250080.0	1081277	18.3	0.0120
8	371.1235	19195	86247744.0	270116	4.6	0.0193
9	715.2599	38524	245509232.0	768902	13.0	0.0186
10	716.2626	29051	93640208.0	293269	5.0	0.0247

^{1}H NMR ($CDCl_3$) spectrum of **1k**

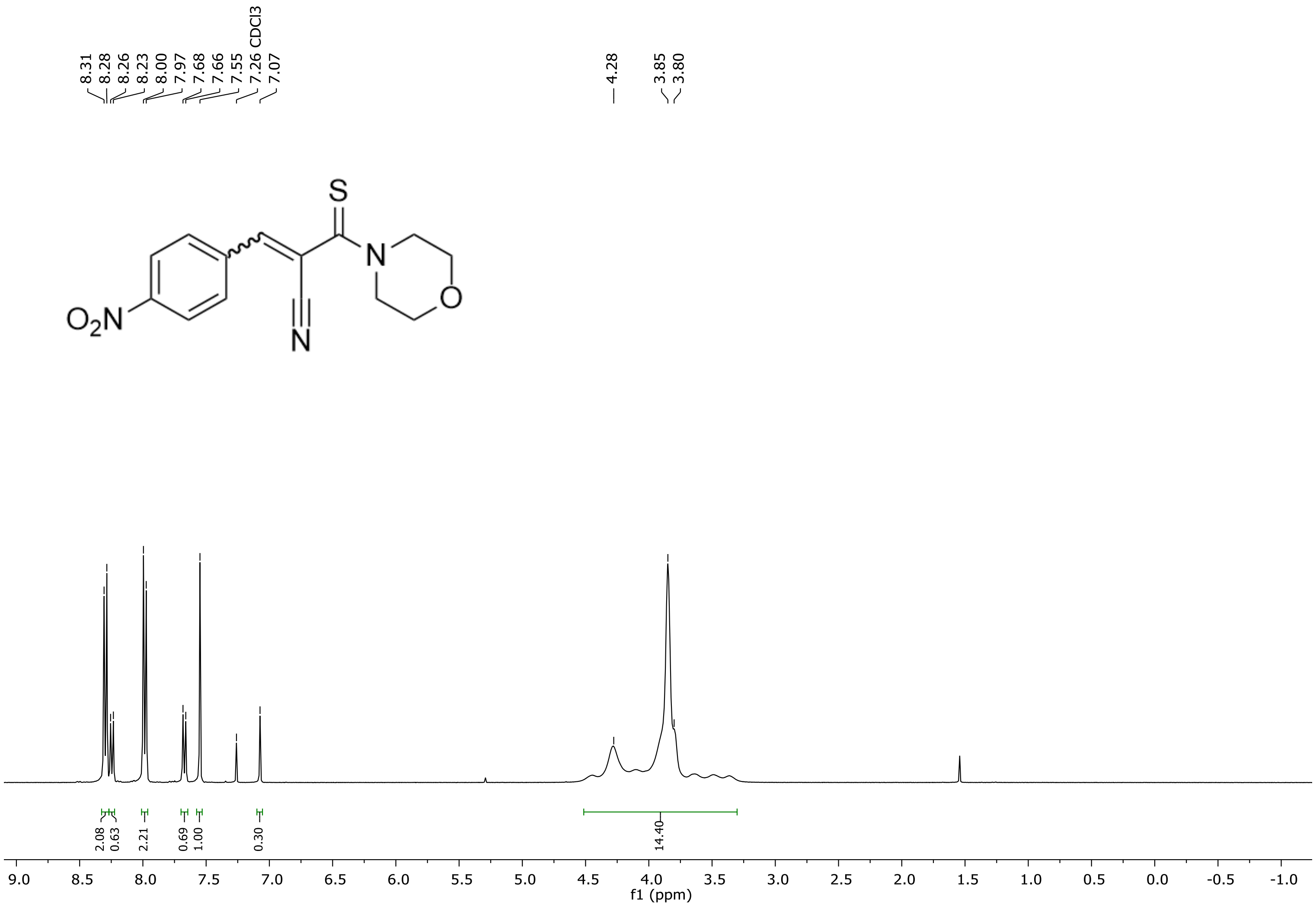

^{13}C NMR ($CDCl_3$) spectrum of **1k**

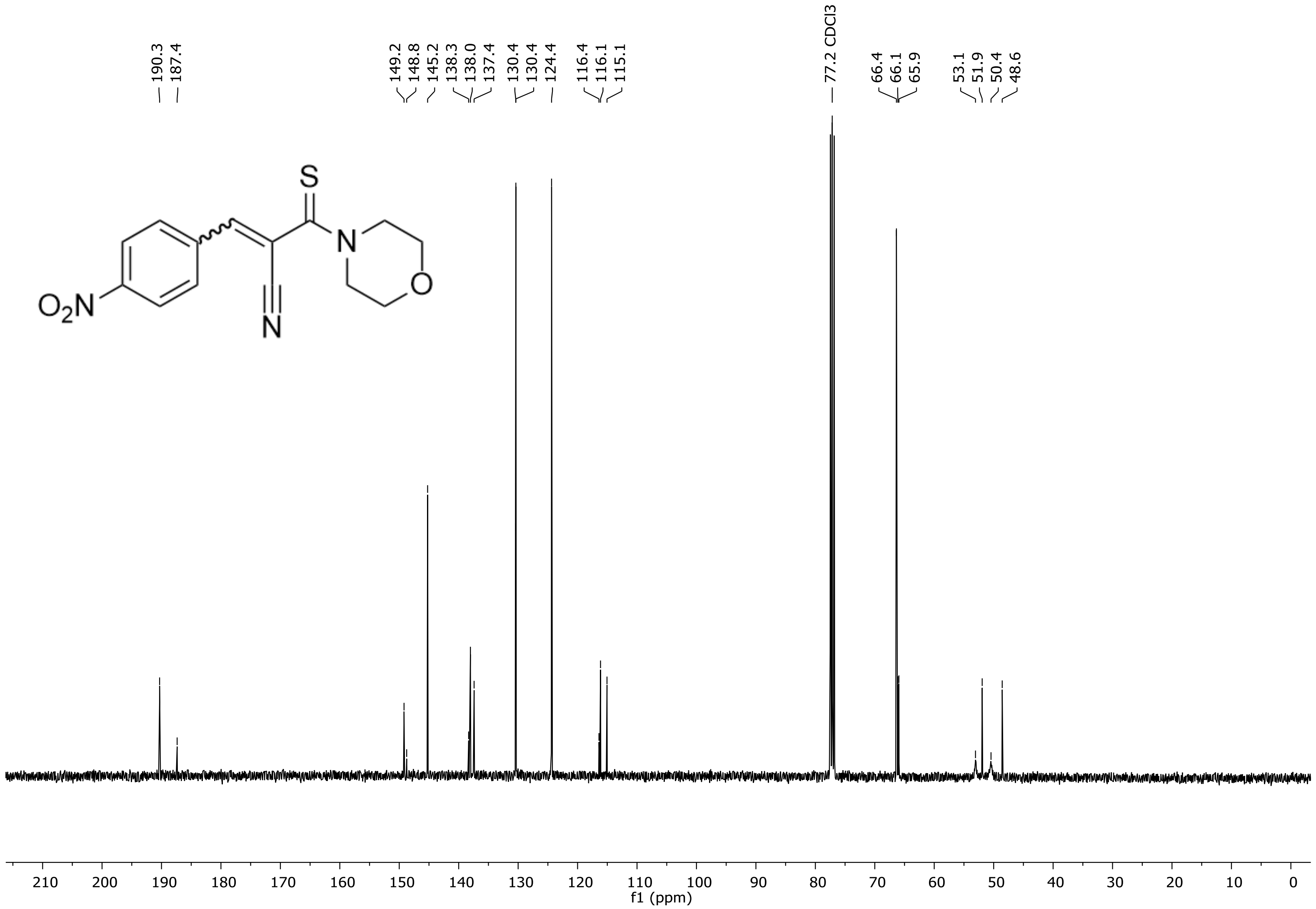

HRMS of **1k**

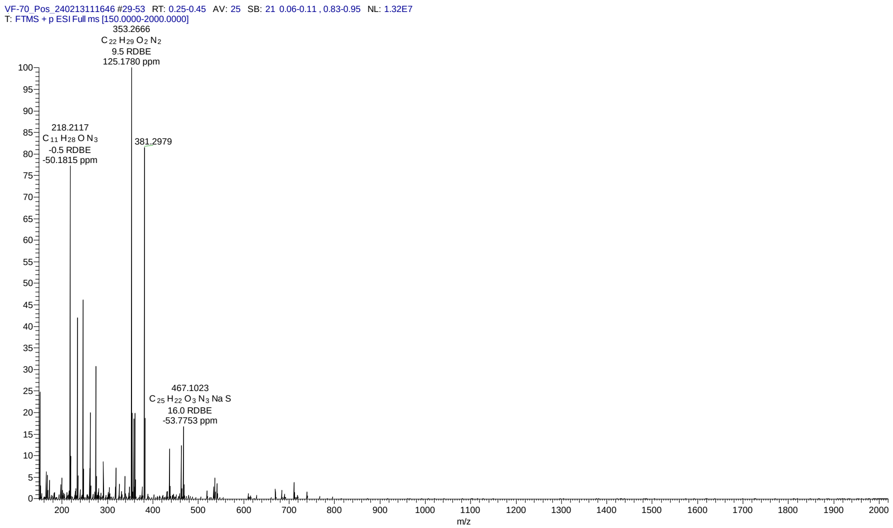
VF-70_Pos_240213111646 #29-53 RT: 0.25-0.45 AV: 25 SB: 21 0.06-0.11 , 0.83-0.95 NL: 1.32E7
T: FTMS + p ESI Full ms [150.0000-2000.0000]
353.2666
C 22 H29 O2 N2
9.5 RDBE
125.1780 ppm
218.2117
C 11 H28 O N3
-0.5 RDBE
-50.1815 ppm
381.2979
467.1023
C 25 H22 O3 N3 Na S
16.0 RDBE
-53.7753 ppm
m/z

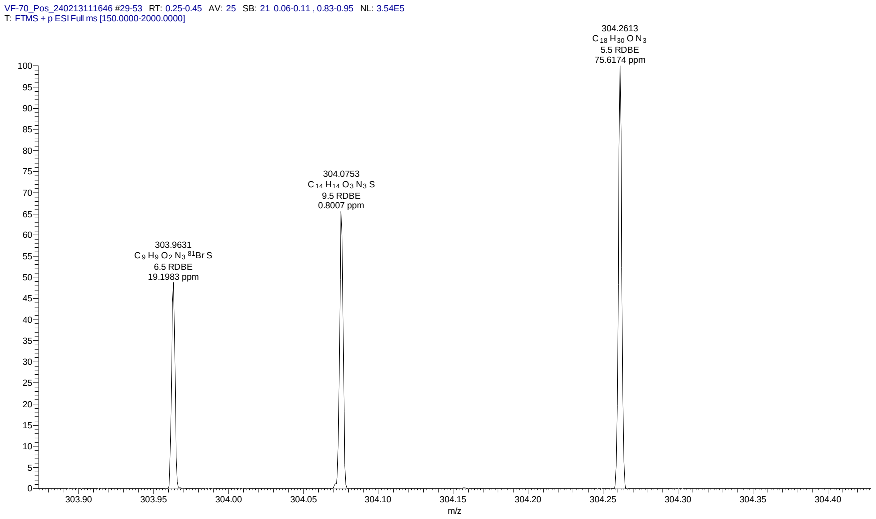
VF-70_Pos_240213111646 #29-53 RT: 0.25-0.45 AV: 25 SB: 21 0.06-0.11 , 0.83-0.95 NL: 3.54E5
T: FTMS + p ESI Full ms [150.0000-2000.0000]
304.2613
C 18 H30 O N3
5.5 RDBE
75.6174 ppm
304.0753
C 14 H14 O3 N3 S
9.5 RDBE
0.8007 ppm
303.9631
C 9 H9 O2 N3 81Br S
6.5 RDBE
19.1983 ppm
m/z

^{1}H NMR ($CDCl_3$) spectrum of **1n**

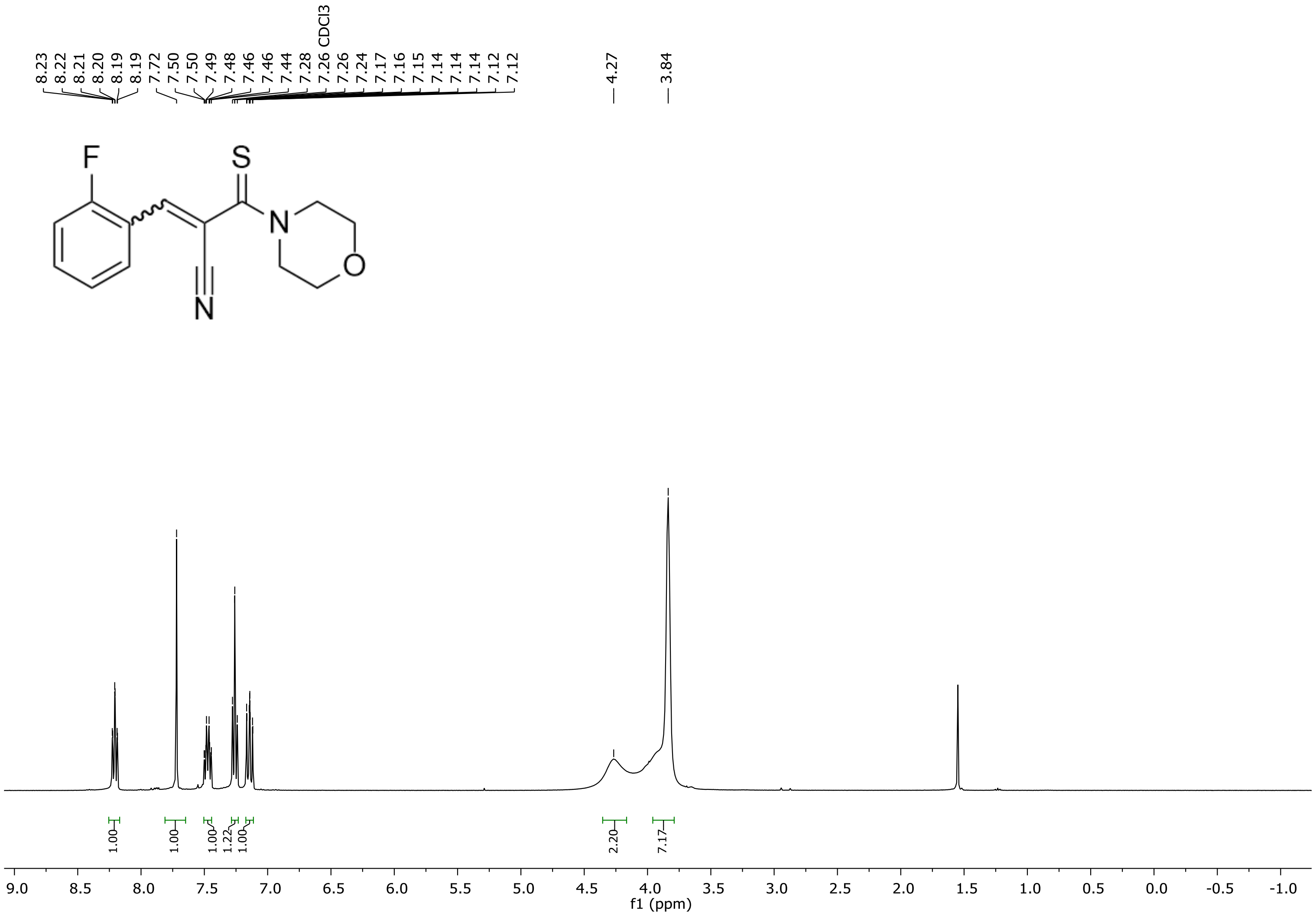

^{13}C NMR ($CDCl_3$) spectrum of **1n**

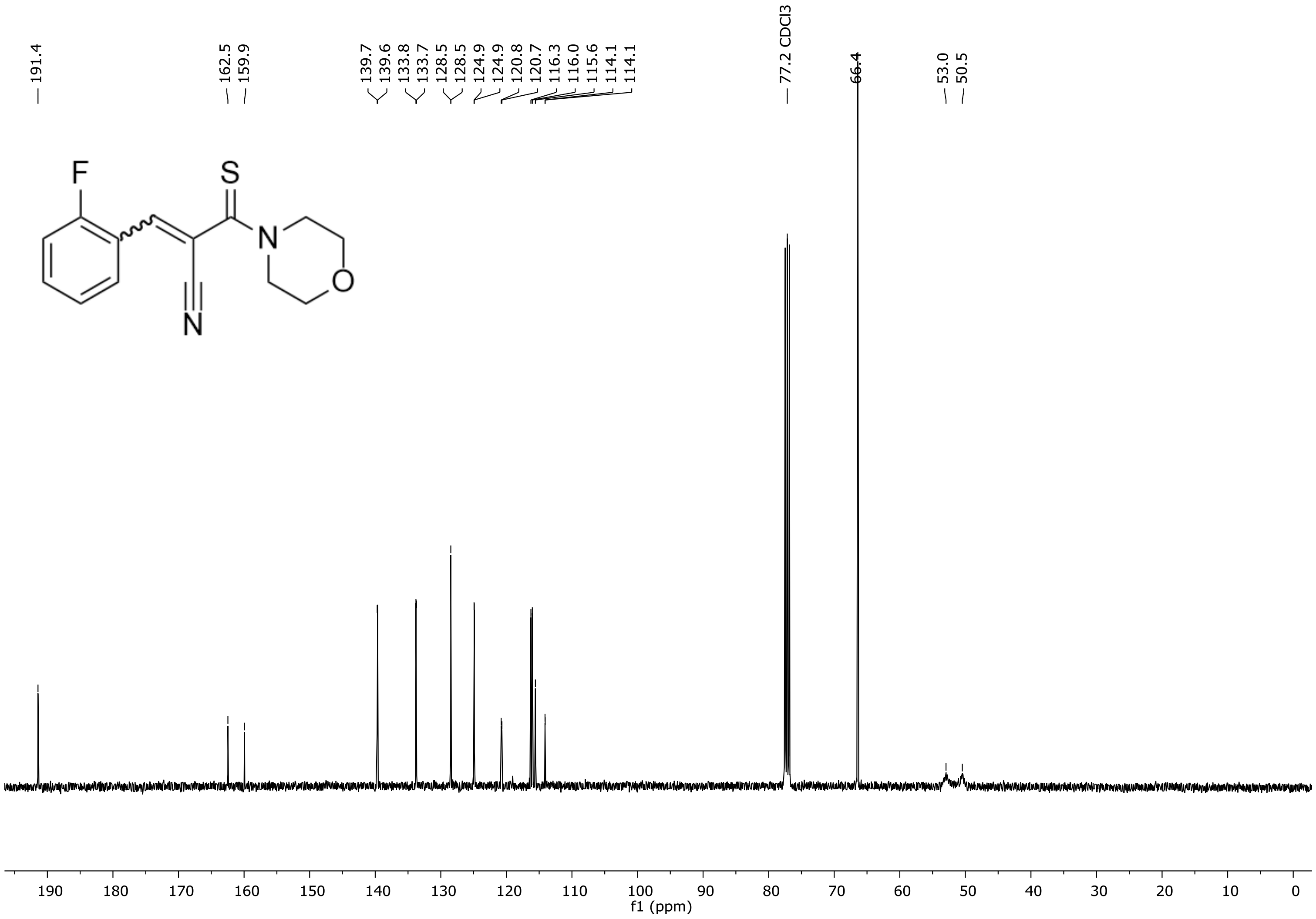

HRMS **1n**

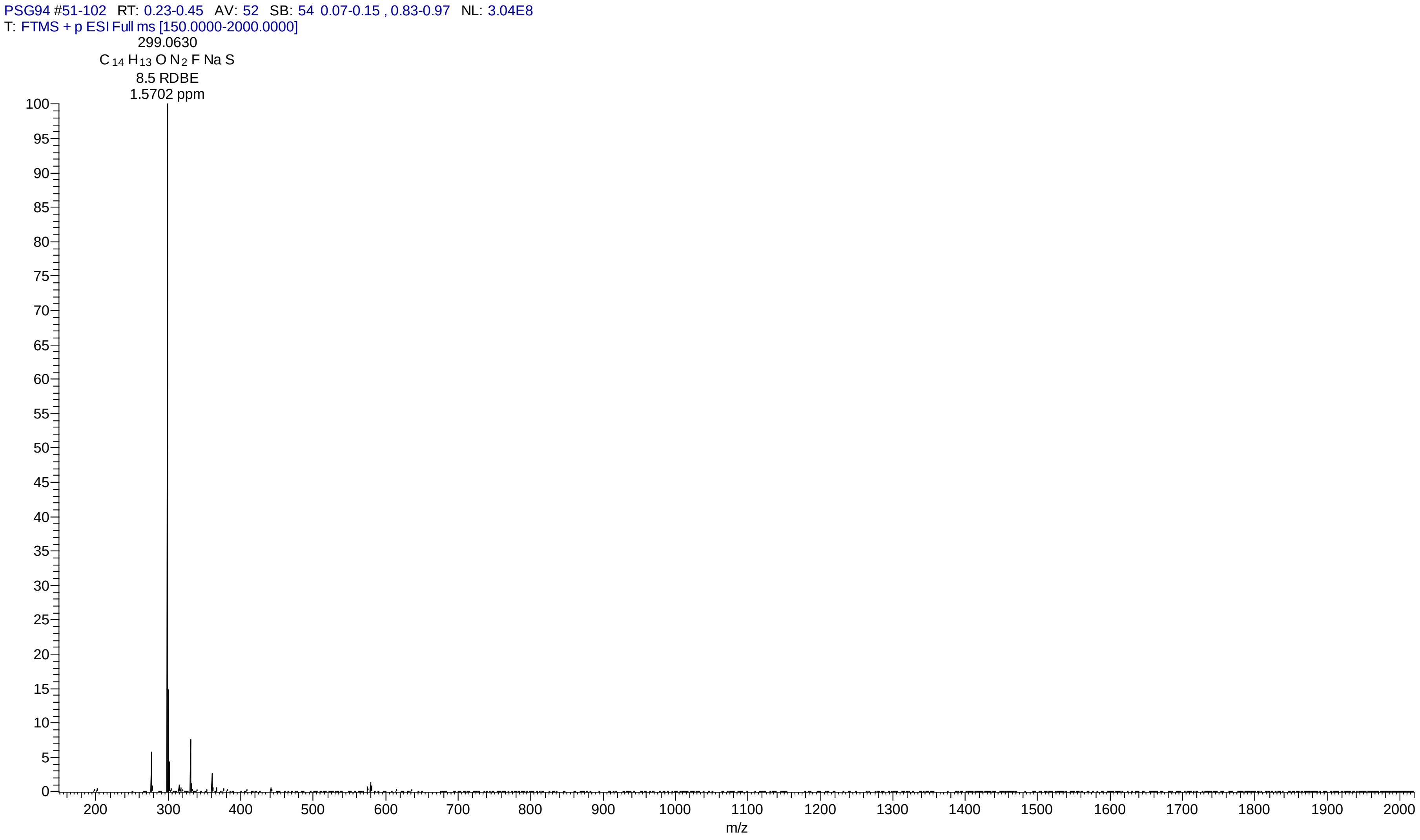

PSG94 #51-102 RT: 0.23-0.45 AV: 52 SB: 54 0.07-0.15 , 0.83-0.97 NL: 3.04E8
T: FTMS + p ESI Full ms [150.0000-2000.0000]
299.0630
C 14 H 13 O N 2 F Na S
8.5 RDBE
1.5702 ppm
m/z

^{1}H NMR ($CDCl_3$) spectrum of **1o**

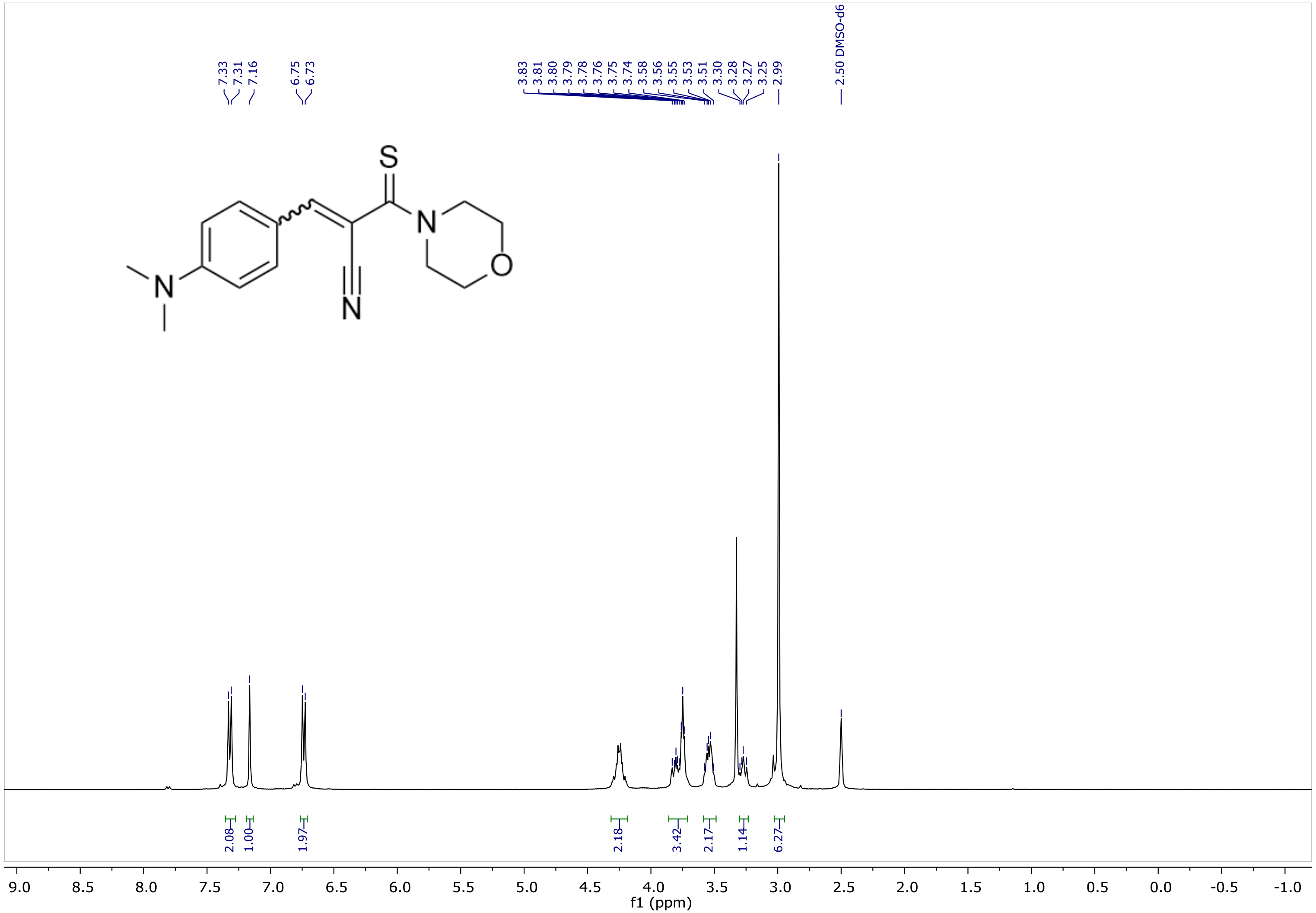

^{13}C NMR ($CDCl_3$) spectrum of **1o**

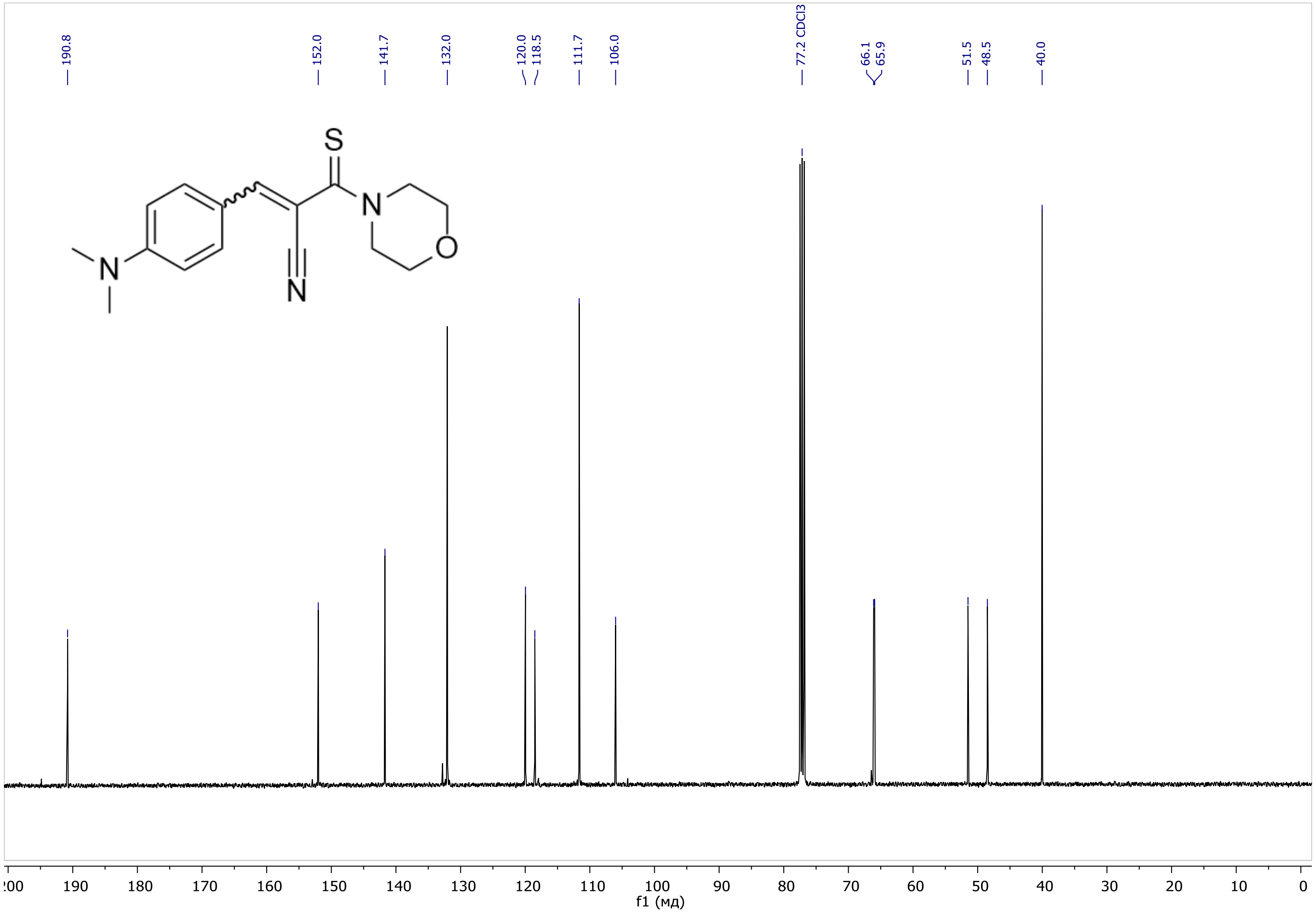

HRMS of **1o**

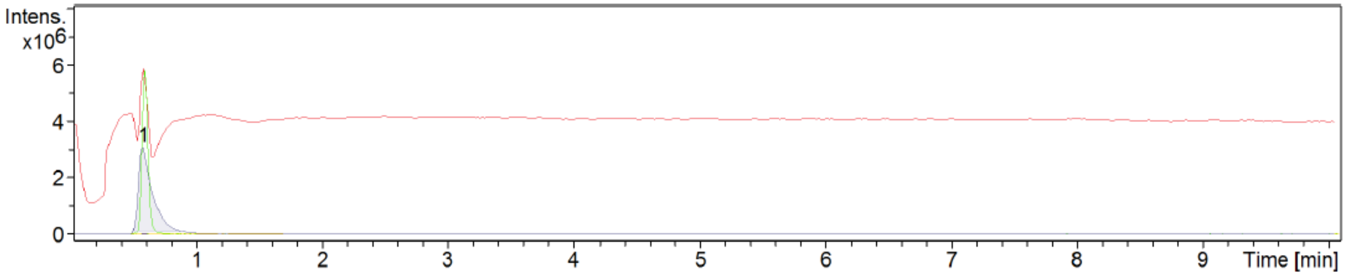

#	RT [min]	Area	Int. Type	I	S/N	Chromatogram	Max. m/z	FWHM [min]
1	0.6	22423718	Manual	3086967	1362.7	EIC 302.1321 +All MS	324.1139	

Cmpd 1, 0.6 min

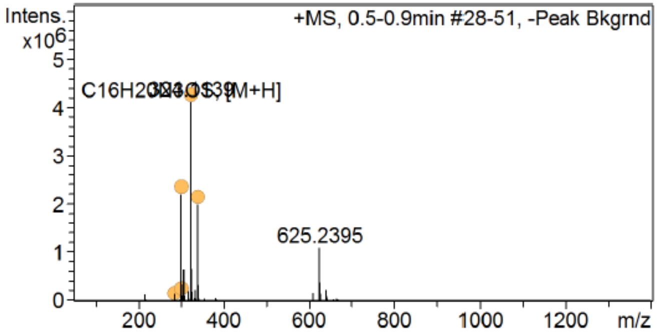

#	m/z	Res.	S/N	I	I %	FWHM
1	302.1321	31816	433271232.0	2194917	53.5	0.0095
2	303.1347	21914	66431592.0	336537	8.2	0.0138
3	308.1364	25284	125473632.0	635639	15.5	0.0122
4	324.1139	34370	809106240.0	4098866	100.0	0.0094
5	325.1166	26225	129311816.0	655083	16.0	0.0124
6	340.0884	30338	393768992.0	1994801	48.7	0.0112
7	341.0909	21635	65457388.0	331602	8.1	0.0158
8	342.0859	19429	44935800.0	227641	5.6	0.0176
9	625.2395	34626	216216320.0	1095334	26.7	0.0181
10	626.2421	26293	74780872.0	378834	9.2	0.0238

^{1}H NMR ($CDCl_3$) spectrum of **1s**

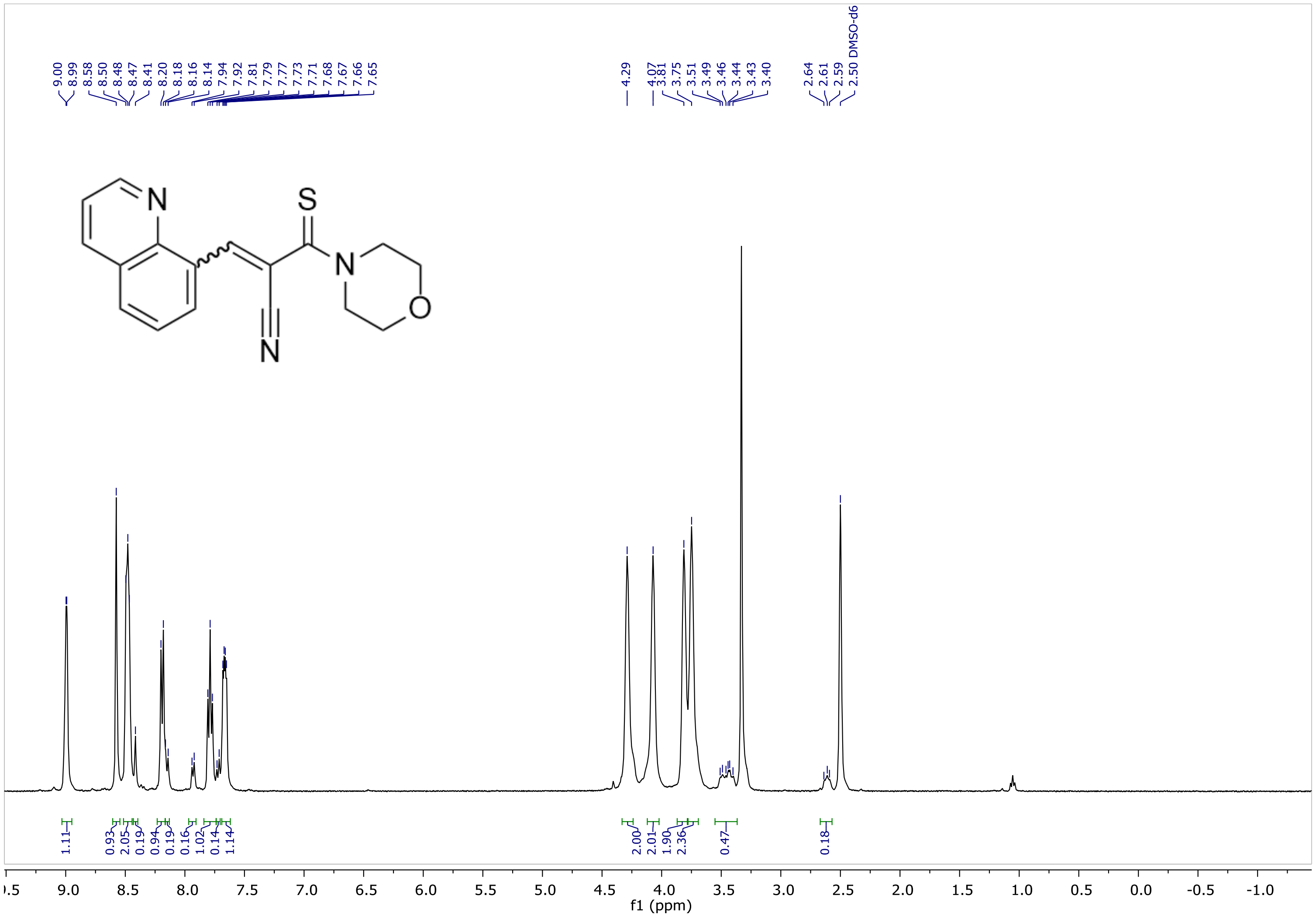

^{13}C NMR ($CDCl_3$) spectrum of **1s**

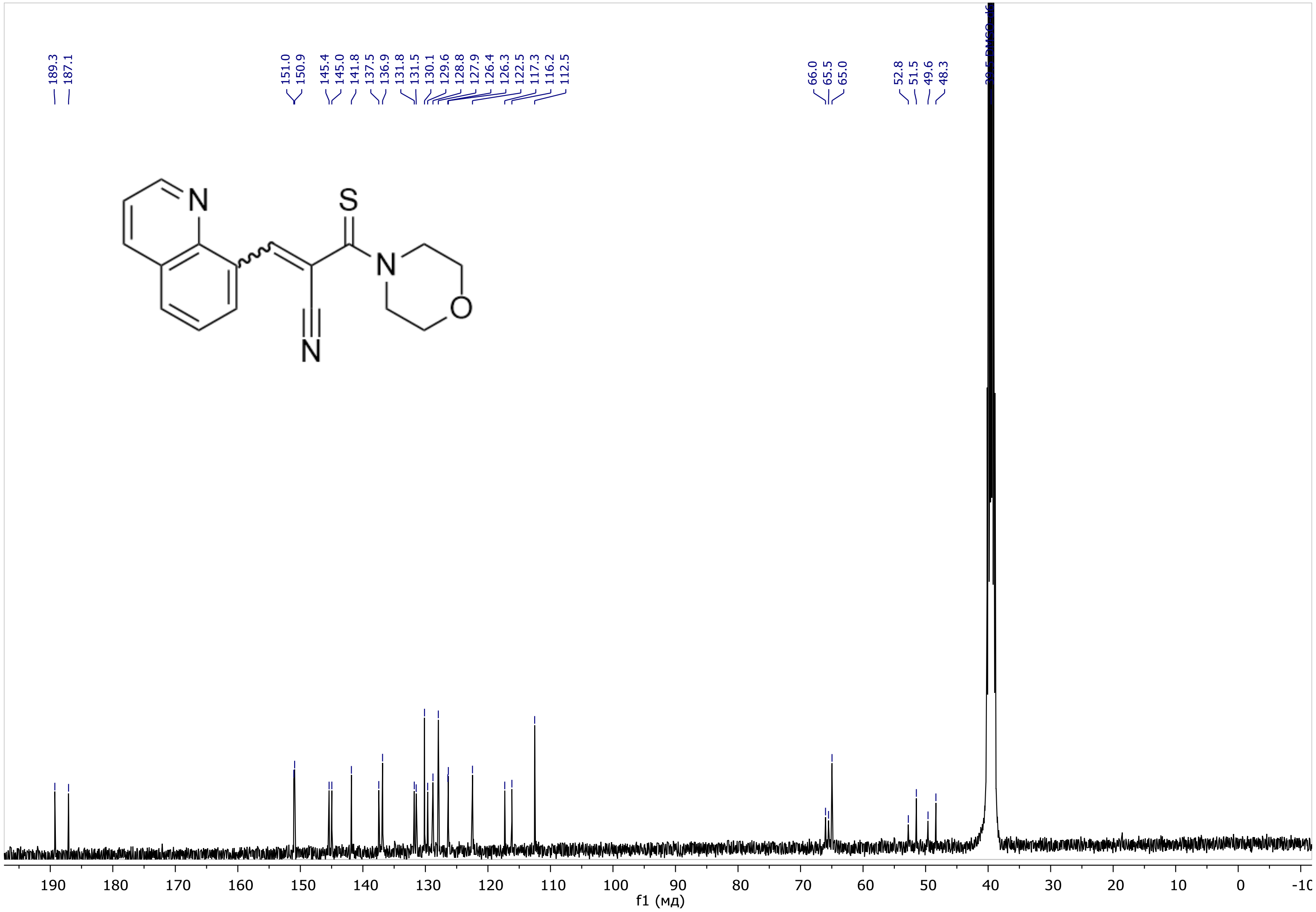

HRMS of **1s**

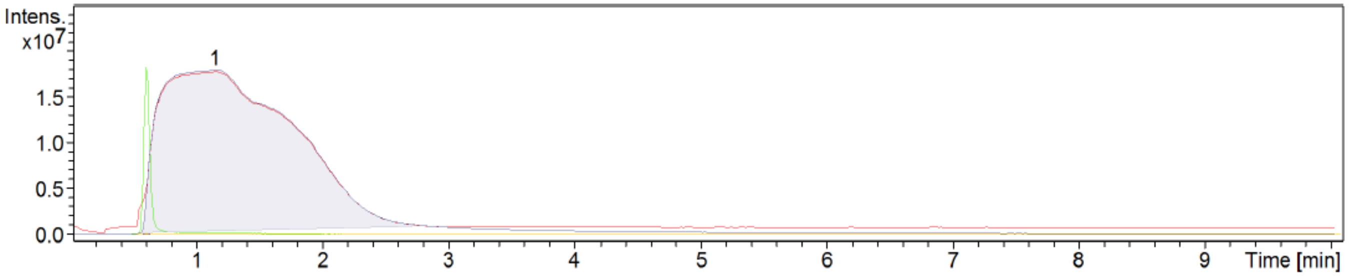

#	RT [min]	Area	Int. Type	I	S/N	Chromatogram	Max. m/z	FWHM [min]
1	1.1	1292521728	Manual	17865116	2900.5	EIC 328.1115 +All MS	328.1115	

Cmpd 1, 1.1 min

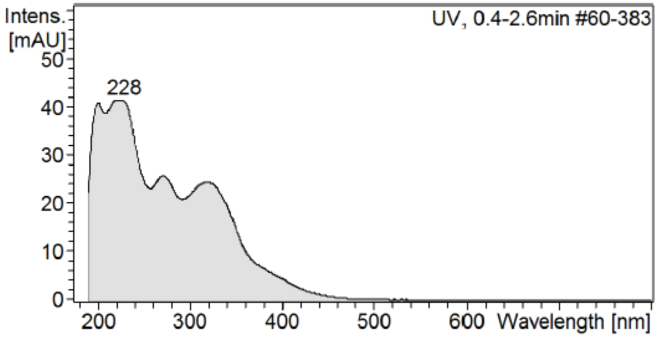

#	Wavelength	Intensity
0	228	41.2

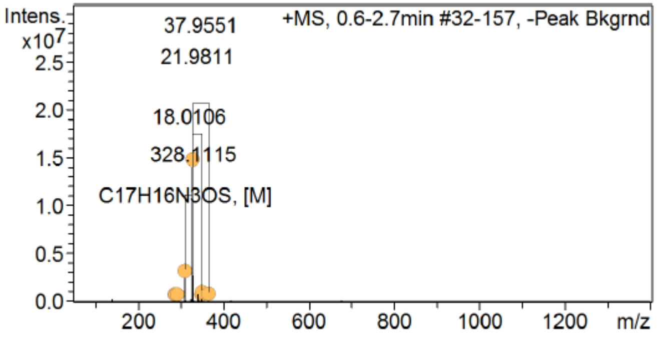

#	m/z	Res.	S/N	I	I %	FWHM
1	139.1226	18943	101209912.0	259449	1.9	0.0073
2	310.1009	32536	947761536.0	2429565	17.4	0.0095
3	311.1035	24608	168408576.0	431711	3.1	0.0126
4	326.0951	21429	101091328.0	259145	1.9	0.0152
5	328.1115	25741	5457144320.0	13989262	100.0	0.0127
6	329.1142	31533	1050522944.0	2692991	19.3	0.0104
7	330.1100	19140	249405584.0	639345	4.6	0.0172
8	342.1267	25349	319576032.0	819226	5.9	0.0135
9	343.1292	20143	64647076.0	165721	1.2	0.0170
10	350.0926	21255	92324624.0	236672	1.7	0.0165

^{1}H NMR ($CDCl_3$) spectrum of **1t**

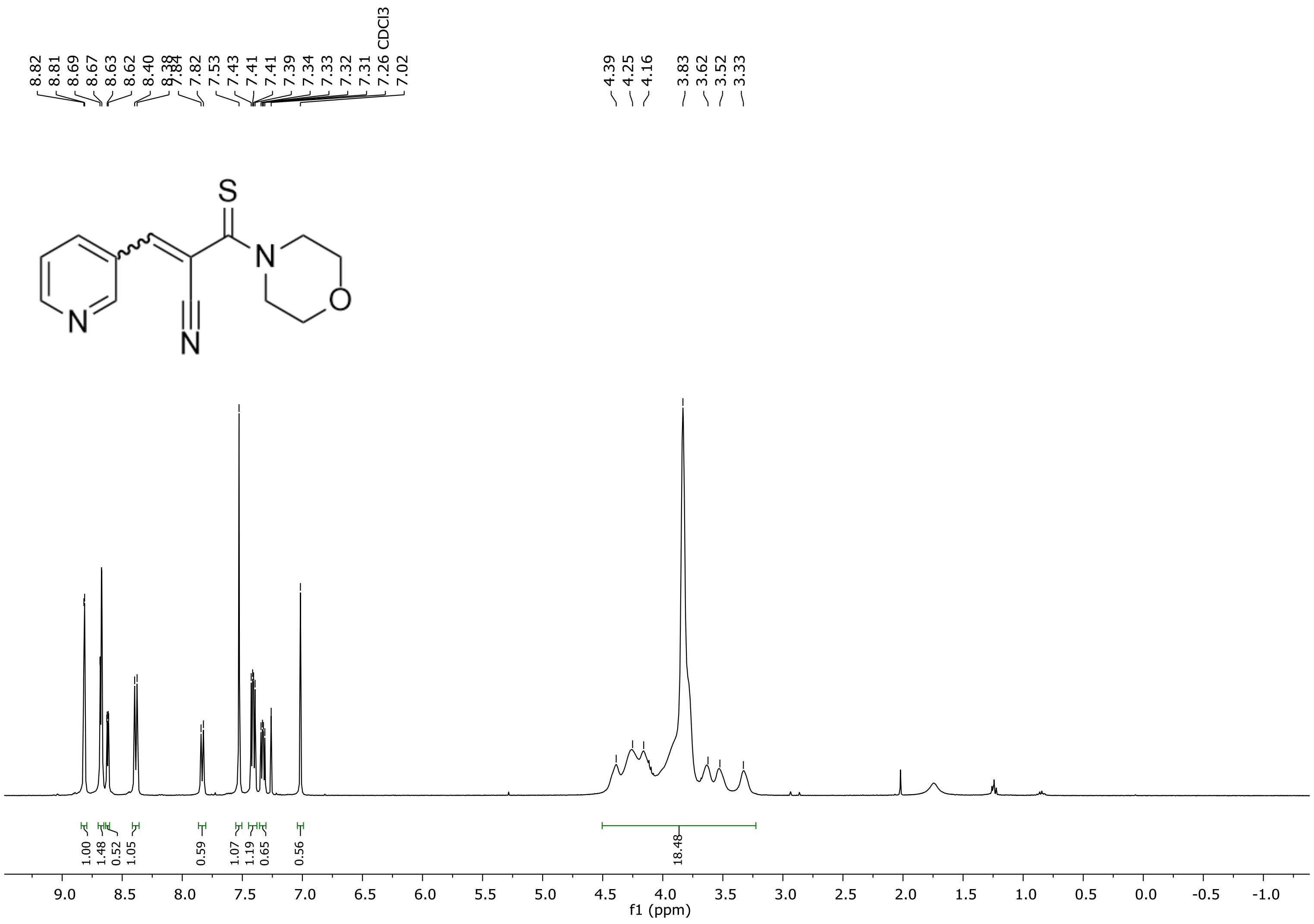

^{13}C NMR ($CDCl_3$) spectrum of **1t**

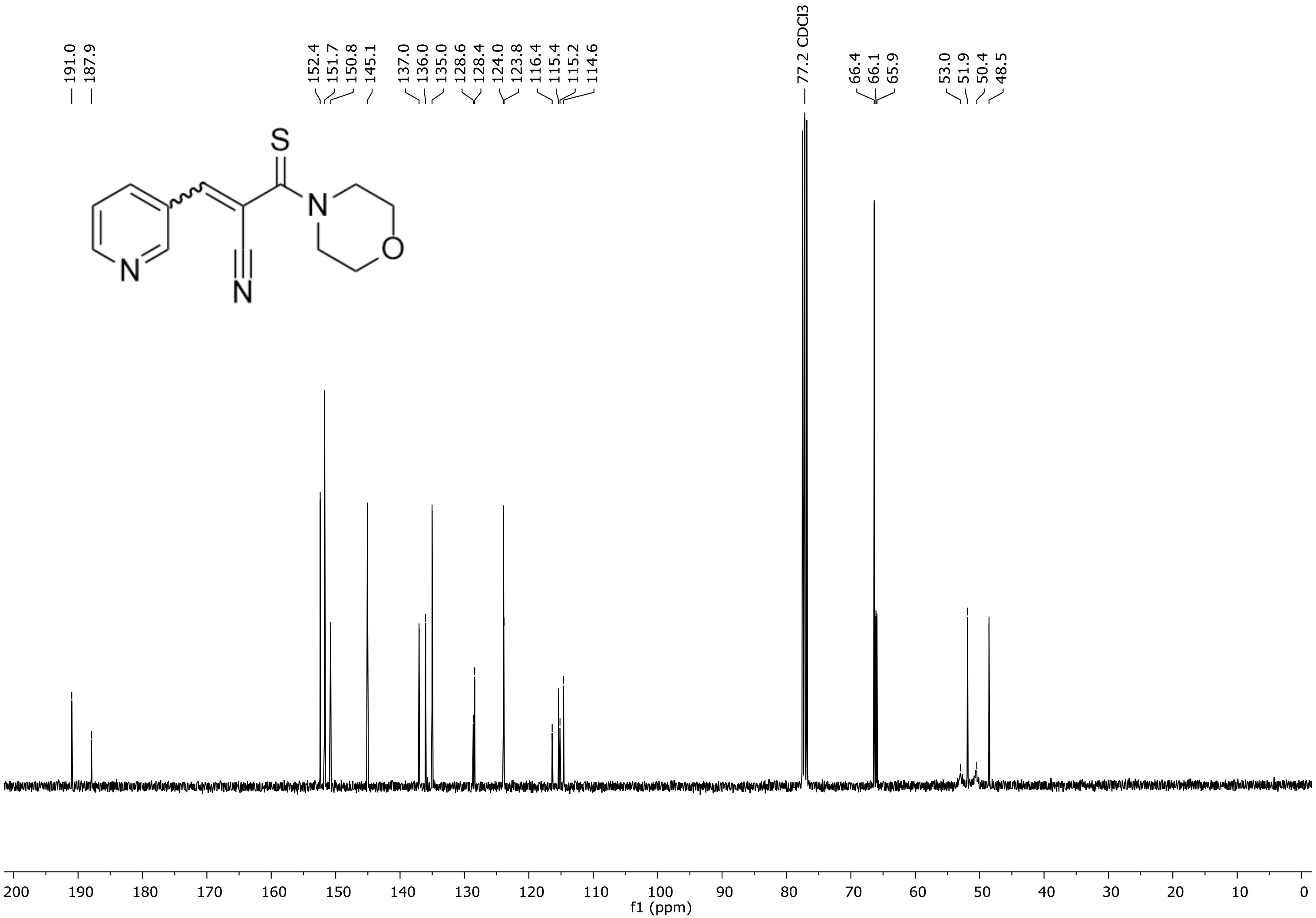

HRMS of **1t**

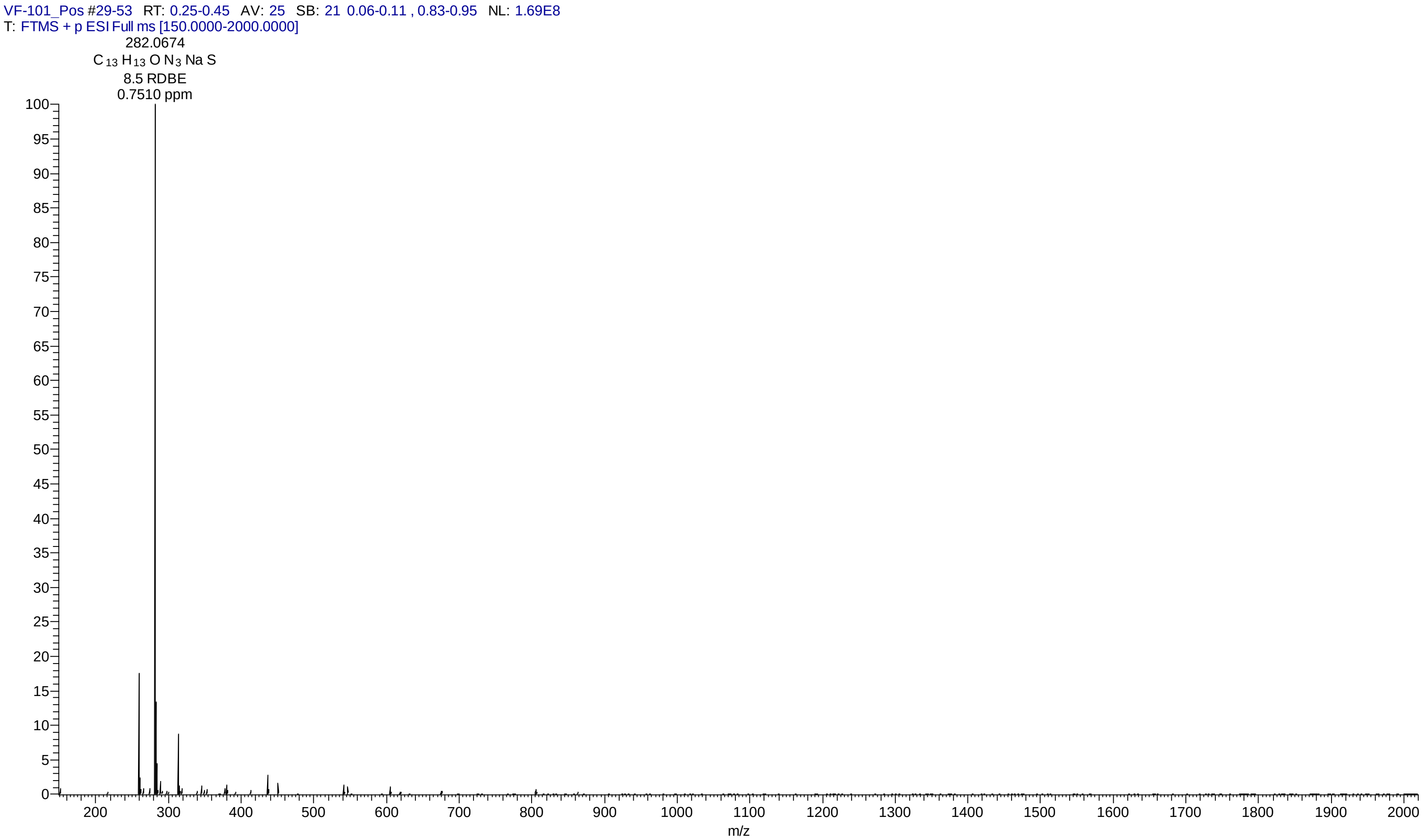

VF-101_Pos #29-53 RT: 0.25-0.45 AV: 25 SB: 21 0.06-0.11 , 0.83-0.95 NL: 1.69E8
T: FTMS + p ESI Full ms [150.0000-2000.0000]
282.0674
C 13 H 13 O N 3 Na S
8.5 RDBE
0.7510 ppm
m/z

^{1}H NMR (DMSO-d$_6$) spectrum of **1v**

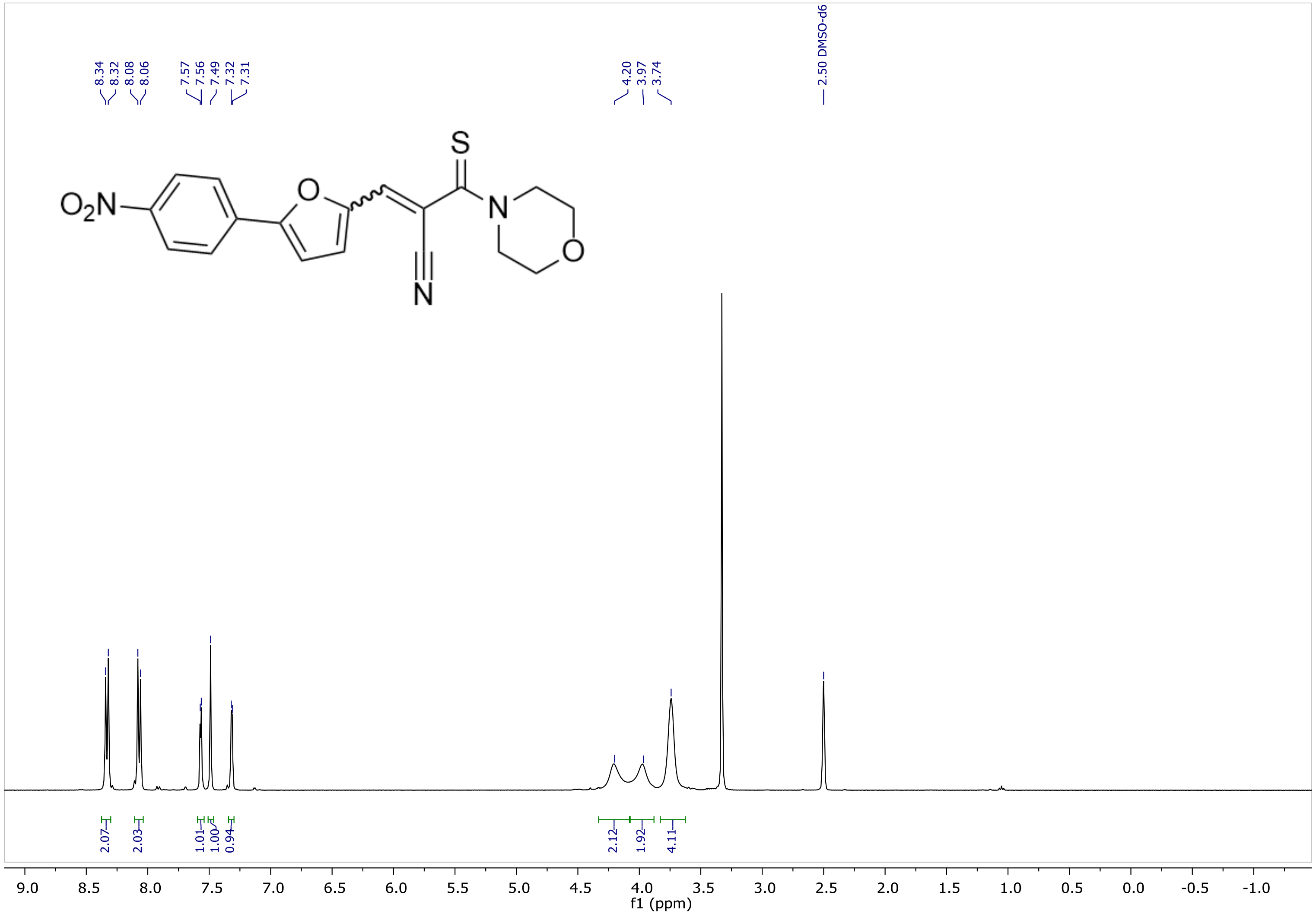

^{13}C NMR (DMSO-d$_6$) spectrum of **1v**

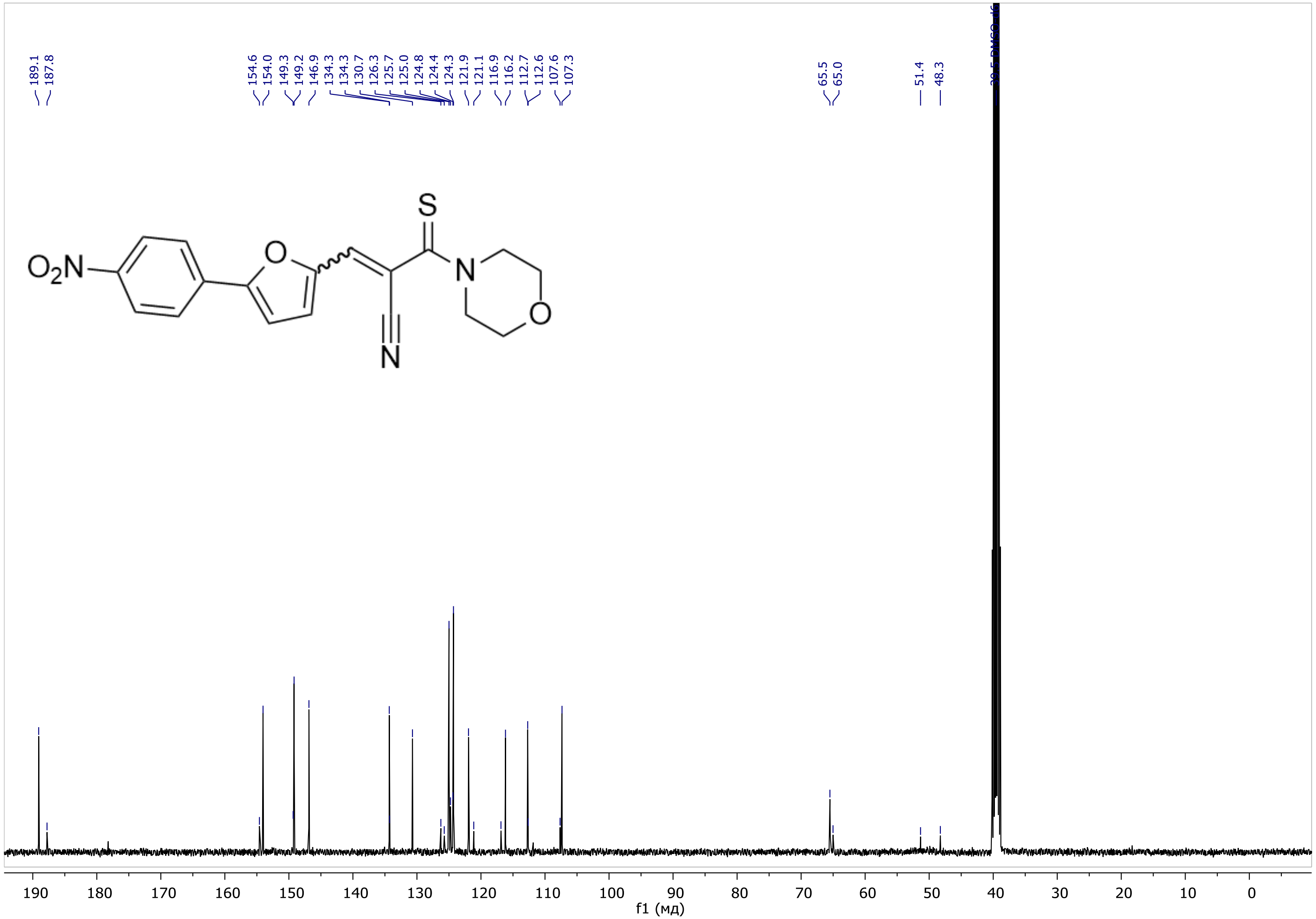

HRMS of **1v**

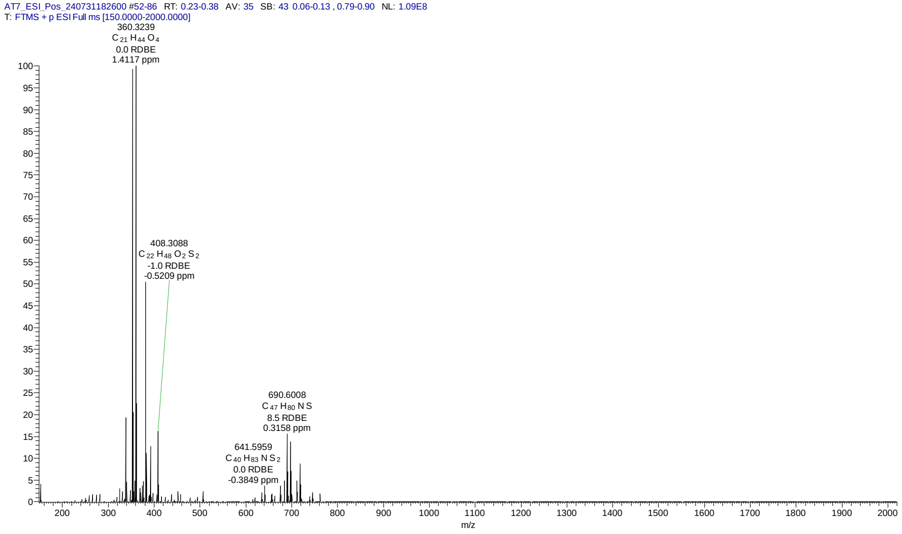
AT7_ESI_Pos_240731182600 #52-86 RT: 0.23-0.38 AV: 35 SB: 43 0.06-0.13 , 0.79-0.90 NL: 1.09E8
T: FTMS + p ESI Full ms [150.0000-2000.0000]
360.3239
C 21 H 44 O 4
0.0 RDBE
1.4117 ppm
408.3088
C 22 H 48 O 2 S 2
-1.0 RDBE
-0.5209 ppm
690.6008
C 47 H 80 N S
8.5 RDBE
0.3158 ppm
641.5959
C 40 H 83 N S 2
0.0 RDBE
-0.3849 ppm
m/z

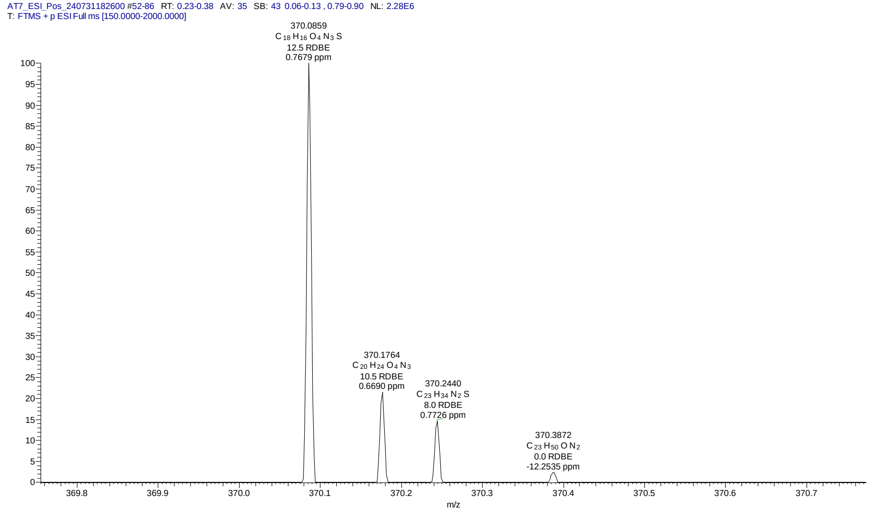
AT7_ESI_Pos_240731182600 #52-86 RT: 0.23-0.38 AV: 35 SB: 43 0.06-0.13 , 0.79-0.90 NL: 2.28E6
T: FTMS + p ESI Full ms [150.0000-2000.0000]
370.0859
C 18 H 16 O 4 N 3 S
12.5 RDBE
0.7679 ppm
370.1764
C 20 H 24 O 4 N 3
10.5 RDBE
0.6690 ppm
370.2440
C 23 H 34 N 2 S
8.0 RDBE
0.7726 ppm
370.3872
C 23 H 50 O N 2
0.0 RDBE
-12.2535 ppm
m/z

^{1}H NMR (DMSO-d$_6$) spectrum of **1w**

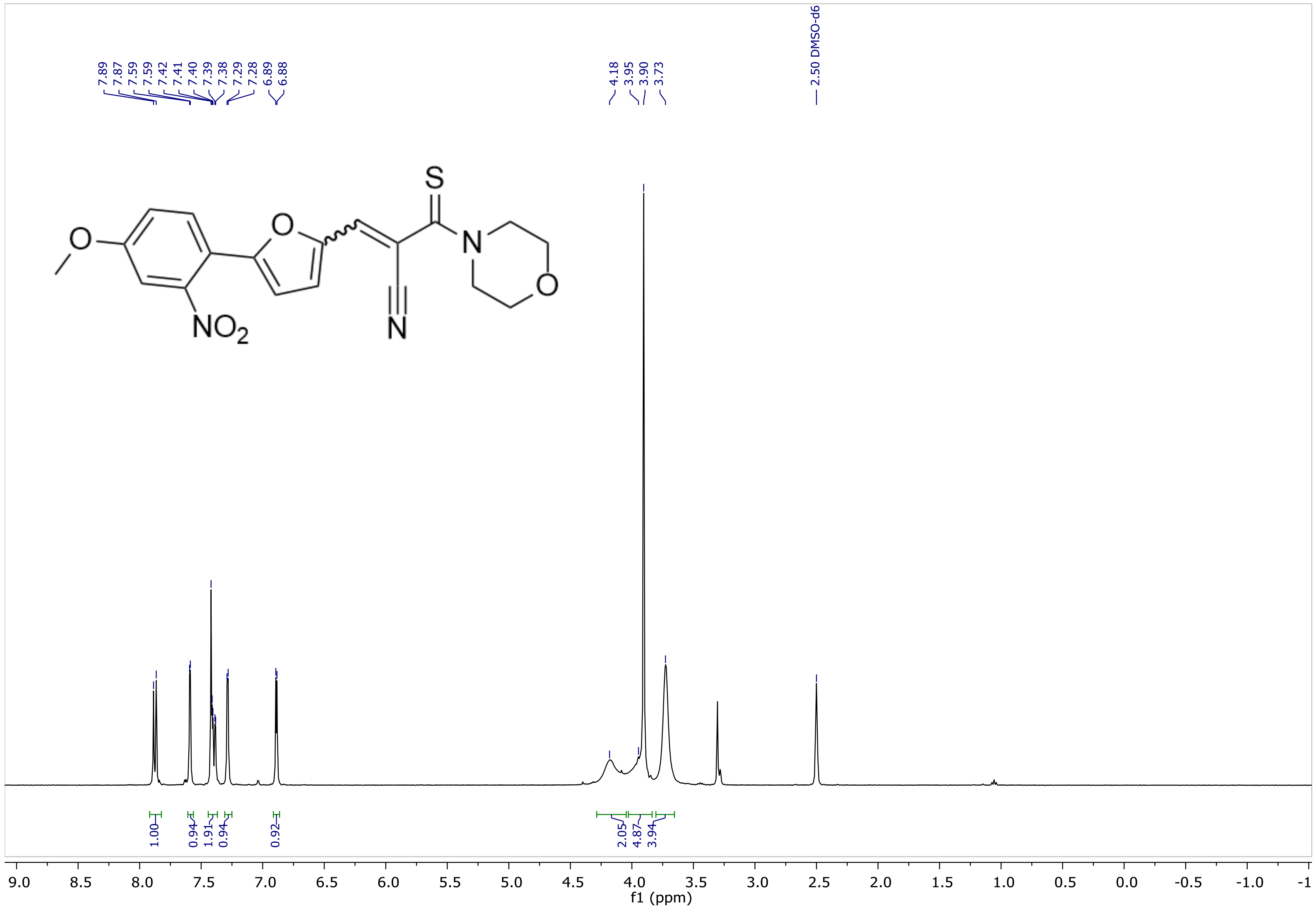

^{13}C NMR (DMSO-d_6) spectrum of **1w**

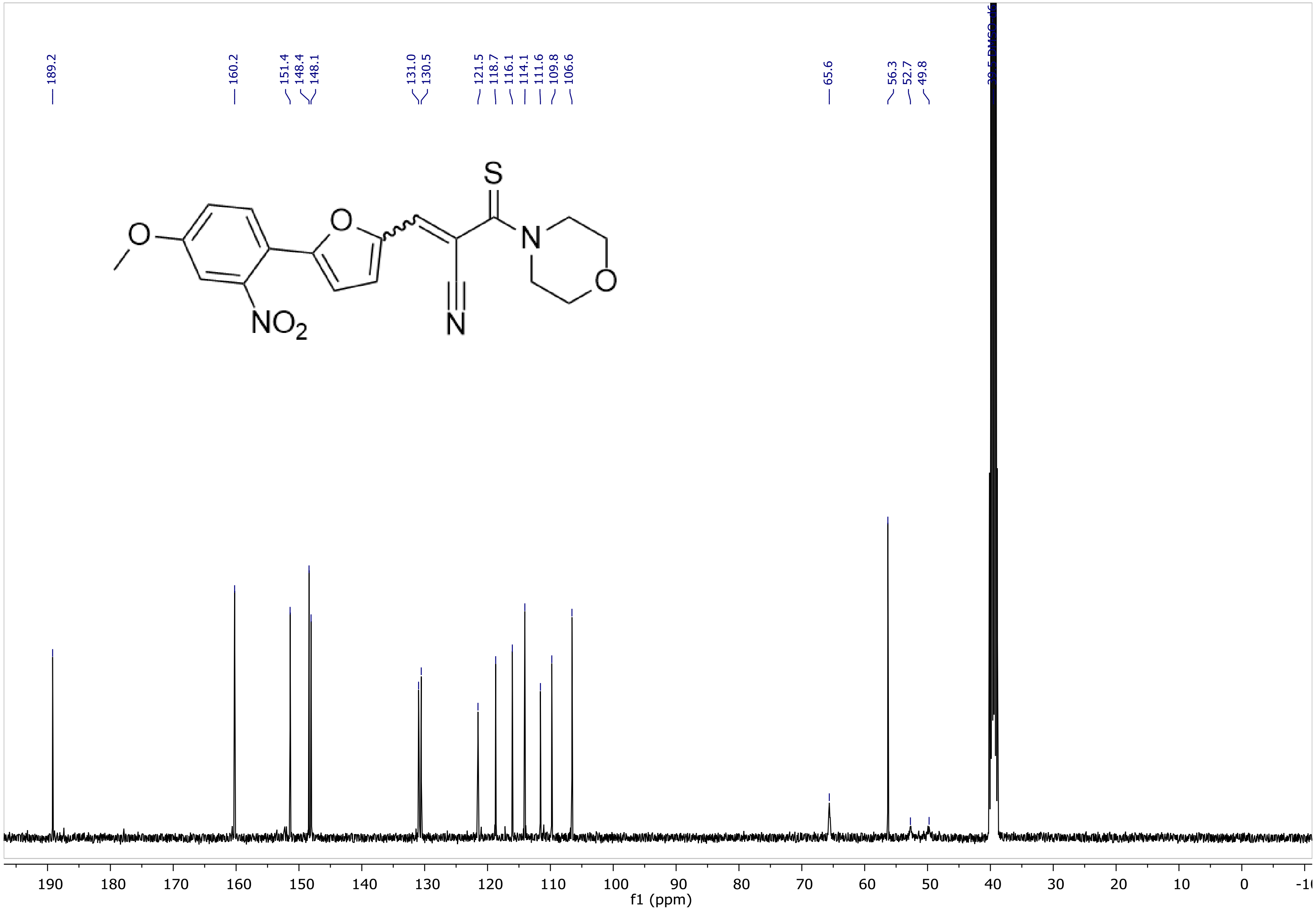

HRMS of **1w**

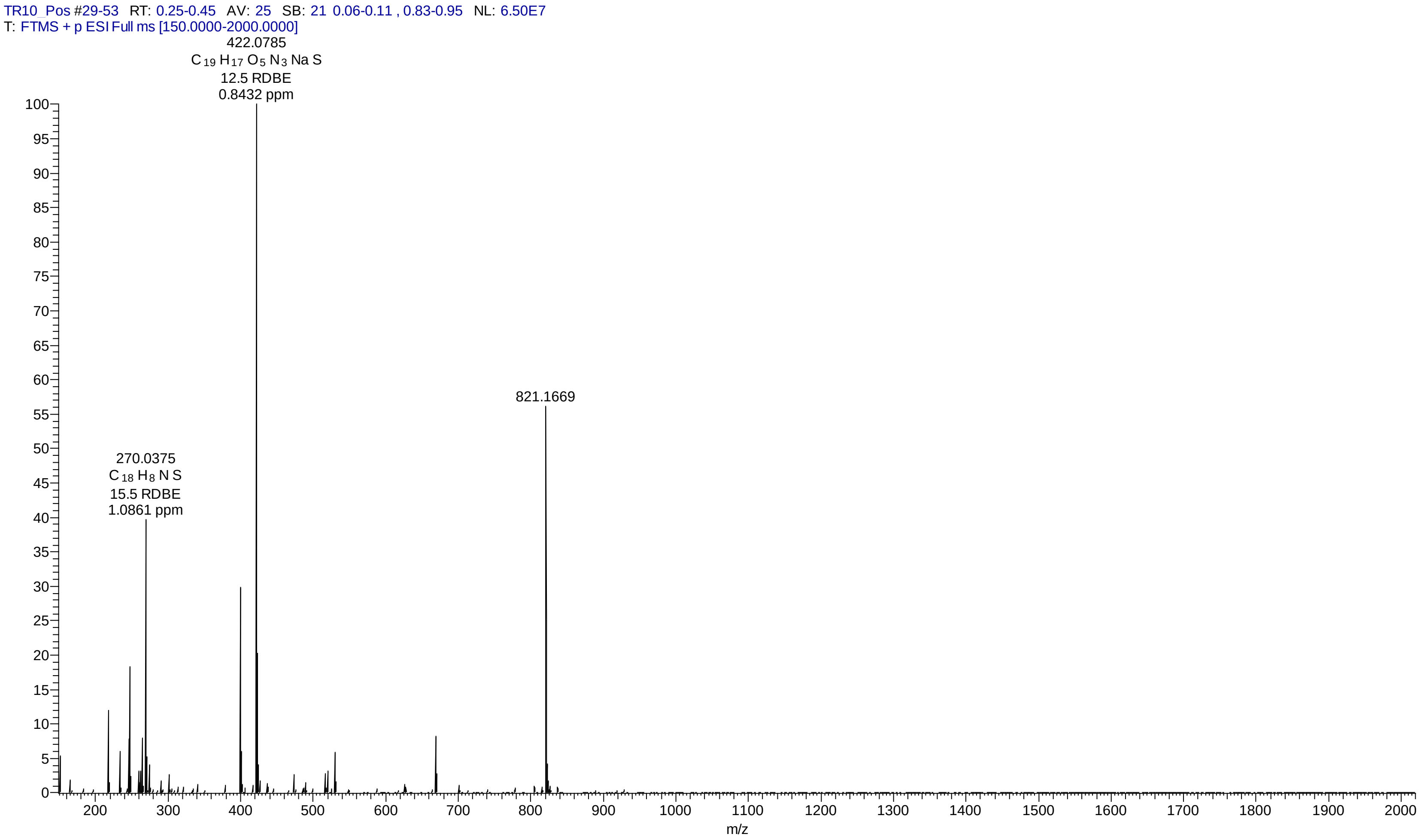

TR10_Pos #29-53 RT: 0.25-0.45 AV: 25 SB: 21 0.06-0.11 , 0.83-0.95 NL: 6.50E7
T: FTMS + p ESI Full ms [150.0000-2000.0000]
422.0785
C 19 H 17 O 5 N 3 Na S
12.5 RDBE
0.8432 ppm
821.1669
270.0375
C 18 H 8 N S
15.5 RDBE
1.0861 ppm
m/z

^{1}H NMR ($CDCl_3$) spectrum of **1y**

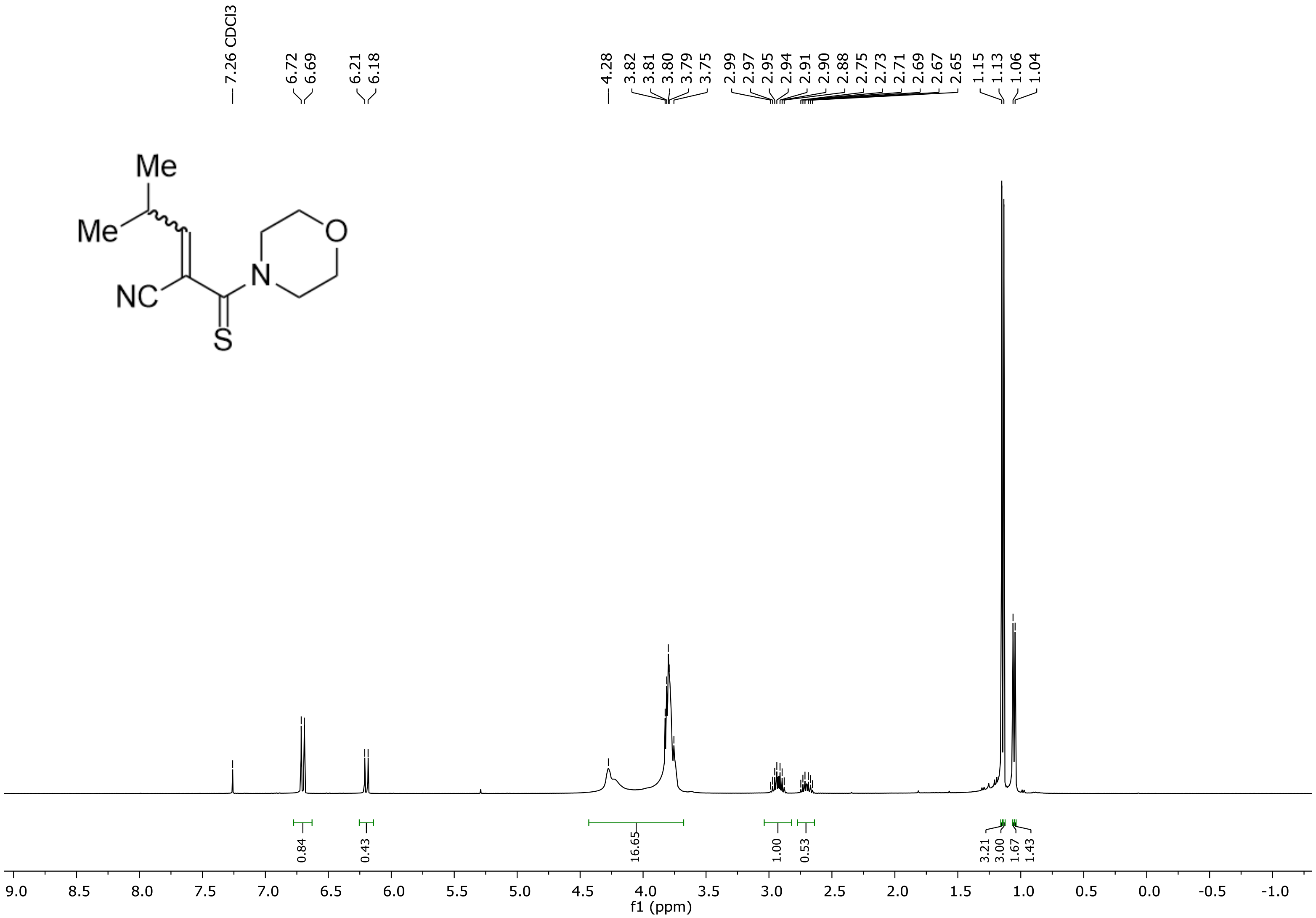

^{13}C NMR ($CDCl_3$) spectrum of **1y**

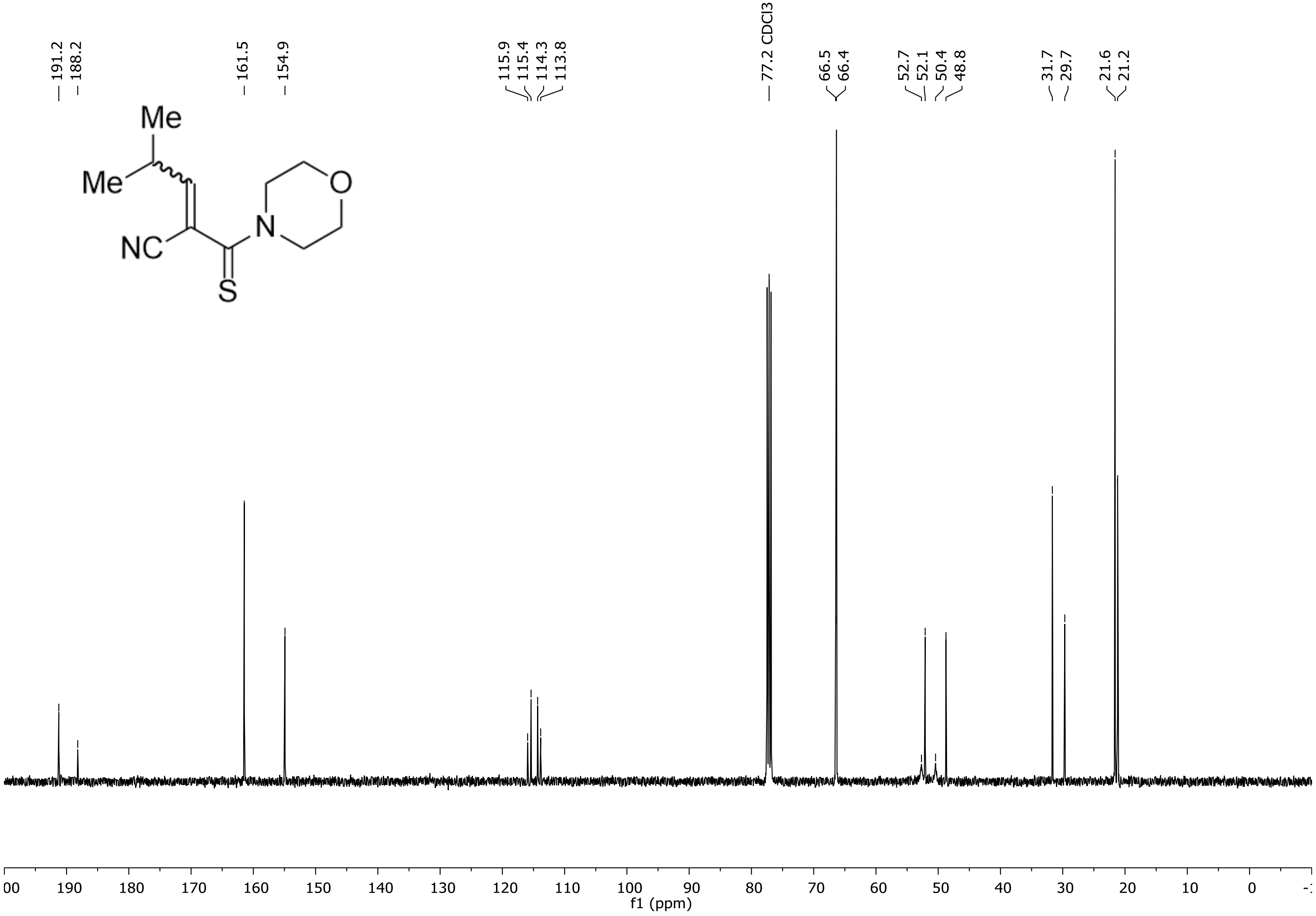

HRMS of **1y**

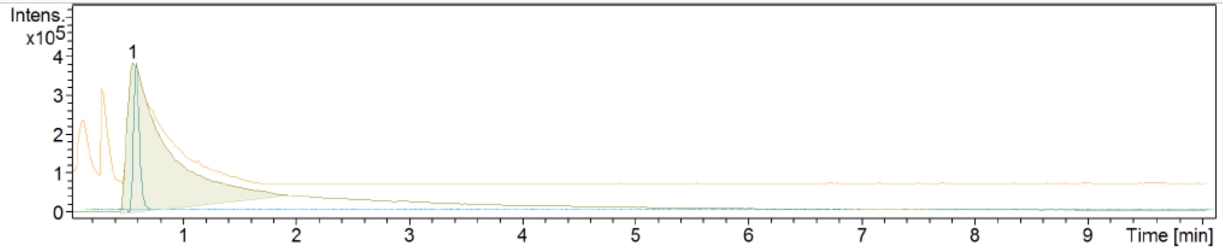

#	RT [min]	Area	Int. Type	I	S/N	Chromatogram	Max. m/z	FWHM [min]
1	0.6	9414062	Manual	380794	711.7	EIC 247.0876±0.02 +All MS	247.0876	

Cmpd 1, 0.6 min

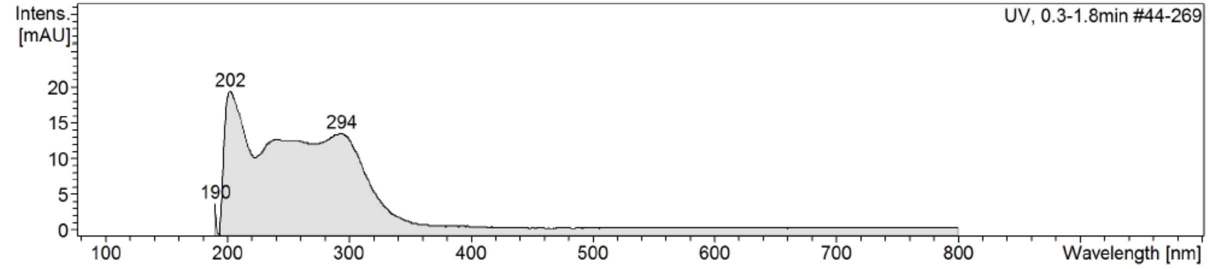

Cmpd 1, 0.6 min

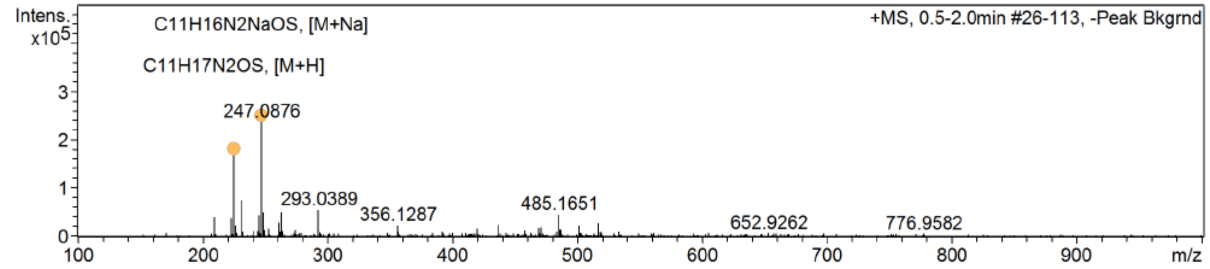

Meas. m/z	#	Ion Formula	m/z	err [ppm]	mSigma	# mSigma	Score	rdb	e⁻ Conf	N-Rule
225.1058	1	$C_{11}H_{17}N_2OS$	225.1056	-0.7	5.6	1	100.00	4.5	even	ok
247.0876	1	$C_{11}H_{16}N_2NaOS$	247.0876	-0.2	38.9	1	100.00	4.5	even	ok

^1H NMR ($CDCl_3$) spectrum of **1z**

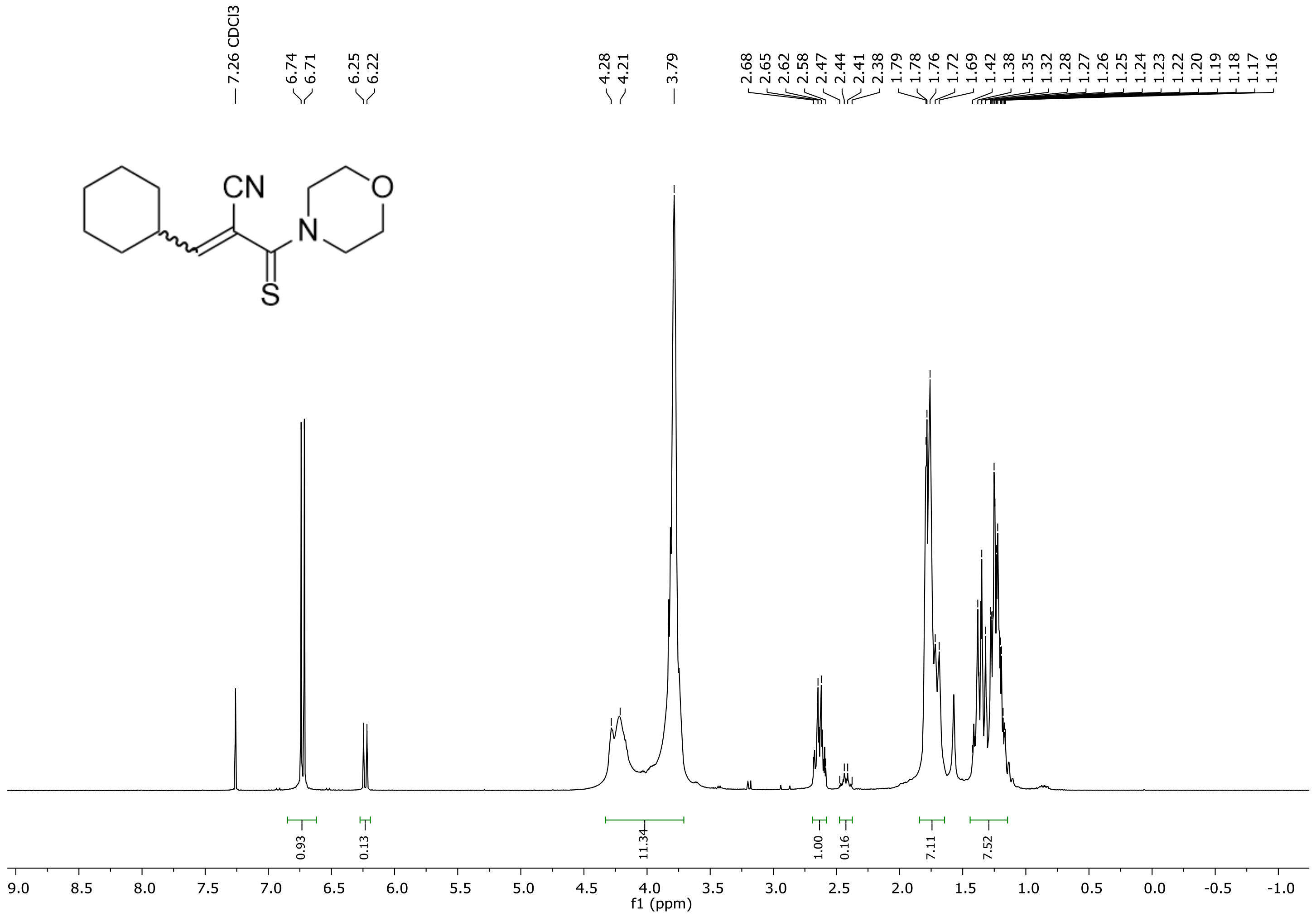

^{13}C NMR ($CDCl_3$) spectrum of **1z**

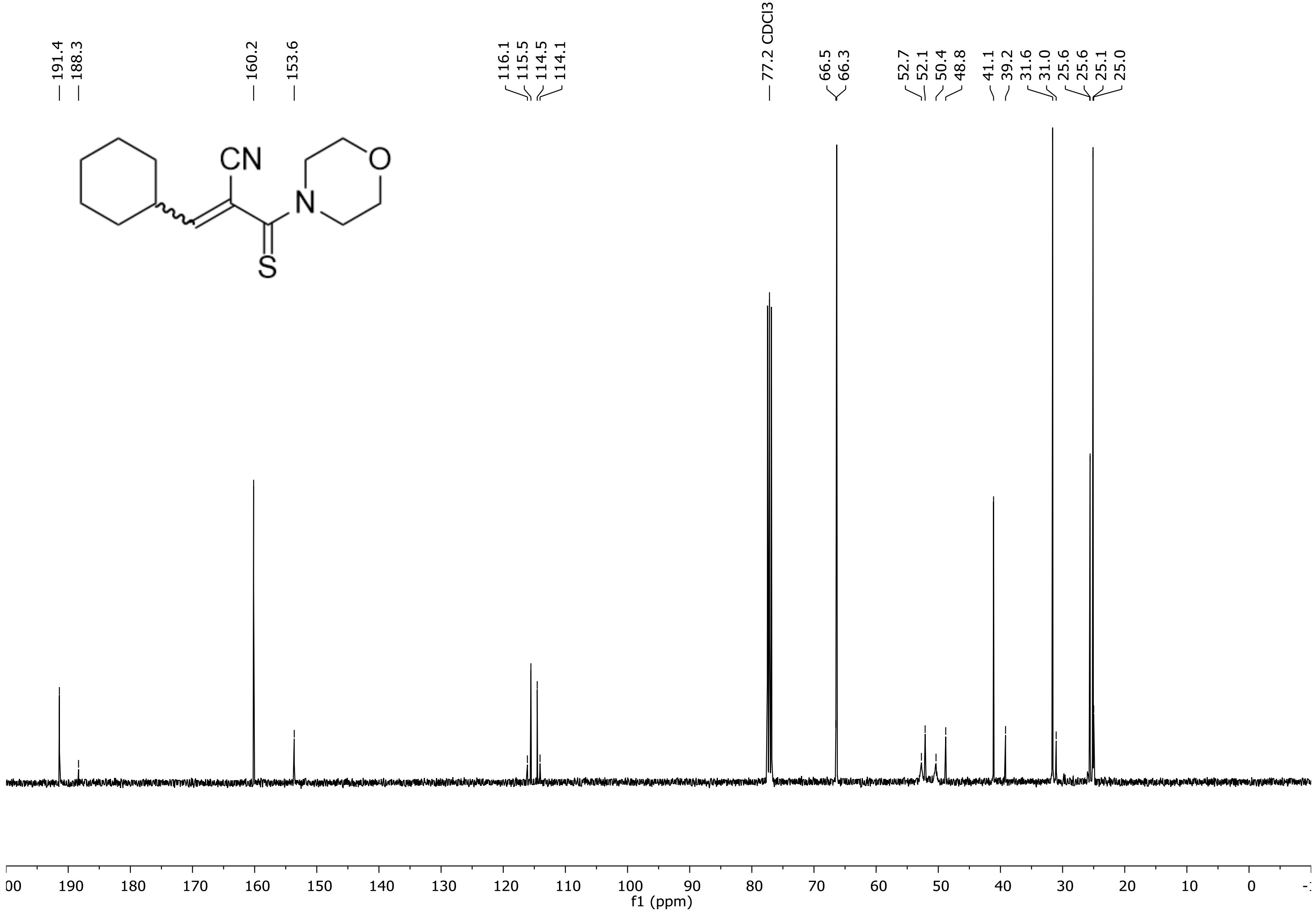

HRMS of **1y**

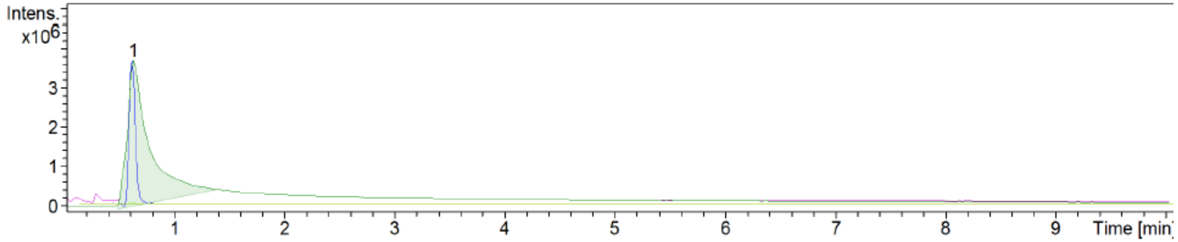

#	RT [min]	Area	Int. Type	I	S/N	Chromatogram	Max. m/z	FWHM [min]
1	0.6	52031964	Manual	3677293	1694.7	EIC 287.1190±0.02 +All MS	287.1190	

Cmpd 1, 0.6 min

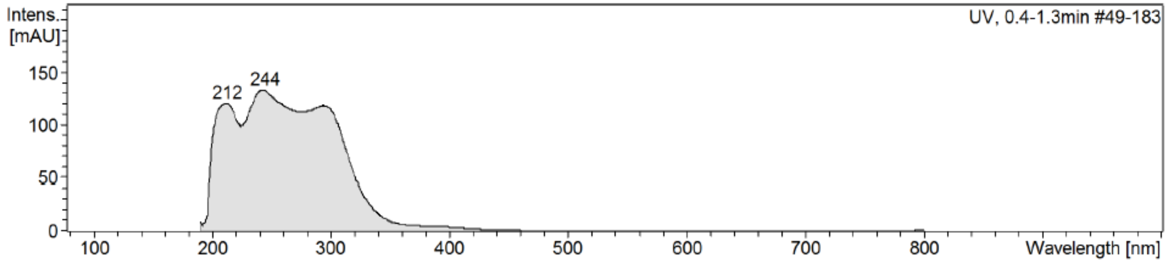

Cmpd 1, 0.6 min

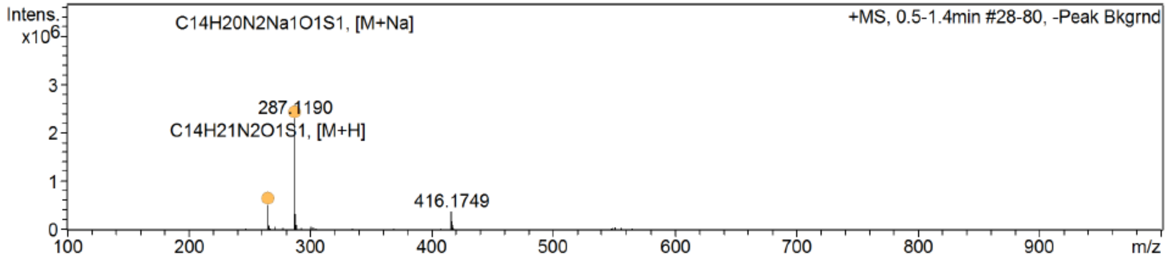

Meas. m/z	#	Ion Formula	m/z	err [ppm]	mSigma	# mSigma	Score	rdb	e⁻ Conf	N-Rule
265.1370	1	C14H21N2OS	265.1369	-0.3	13.9	1	100.00	5.5	even	ok
287.1190	1	C14H20N2NaOS	287.1189	-0.4	14.7	1	100.00	5.5	even	ok

^{1}H NMR ($CDCl_3$) spectrum of **3aa**

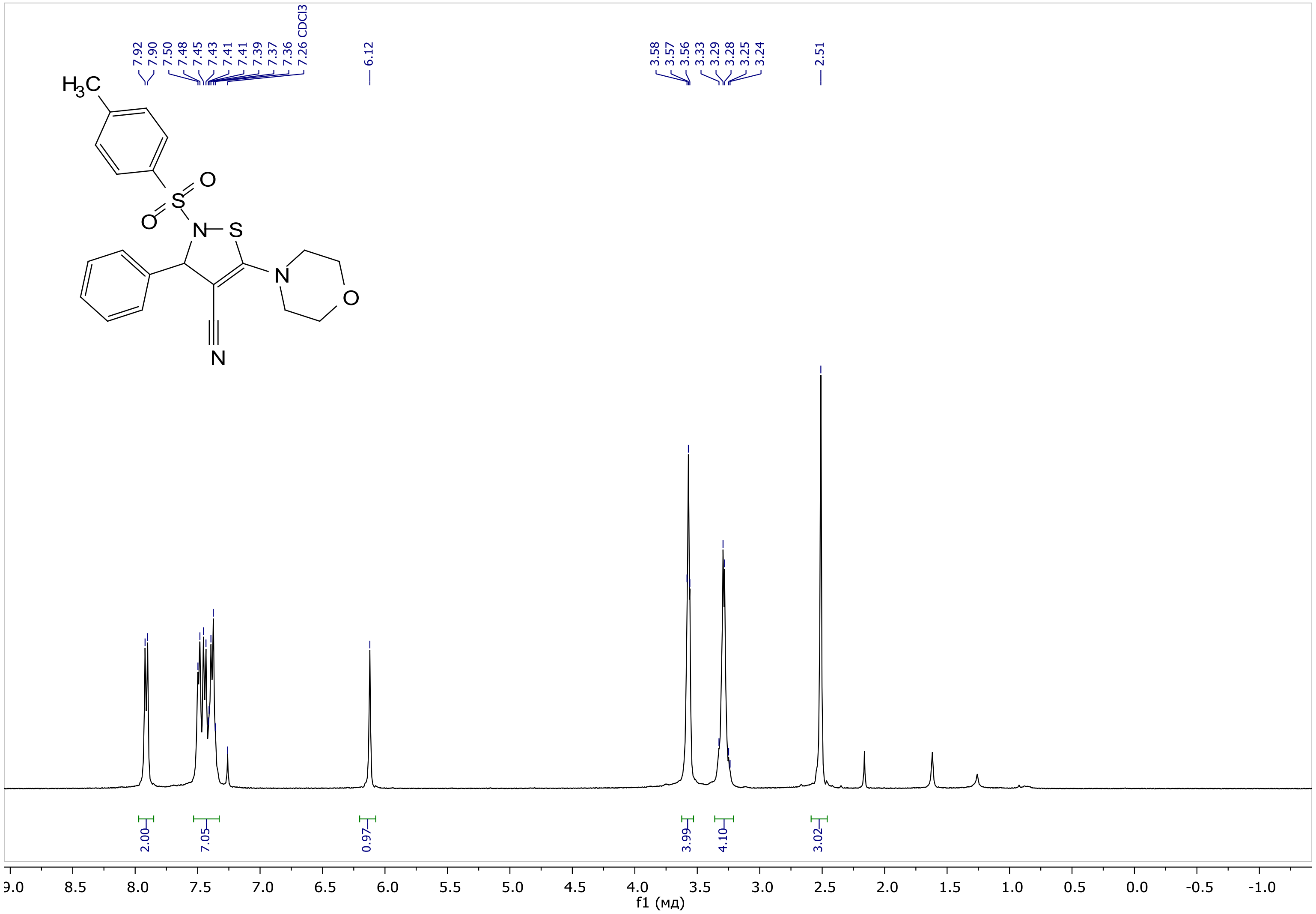

^{13}C NMR ($CDCl_3$) spectrum of **3aa**

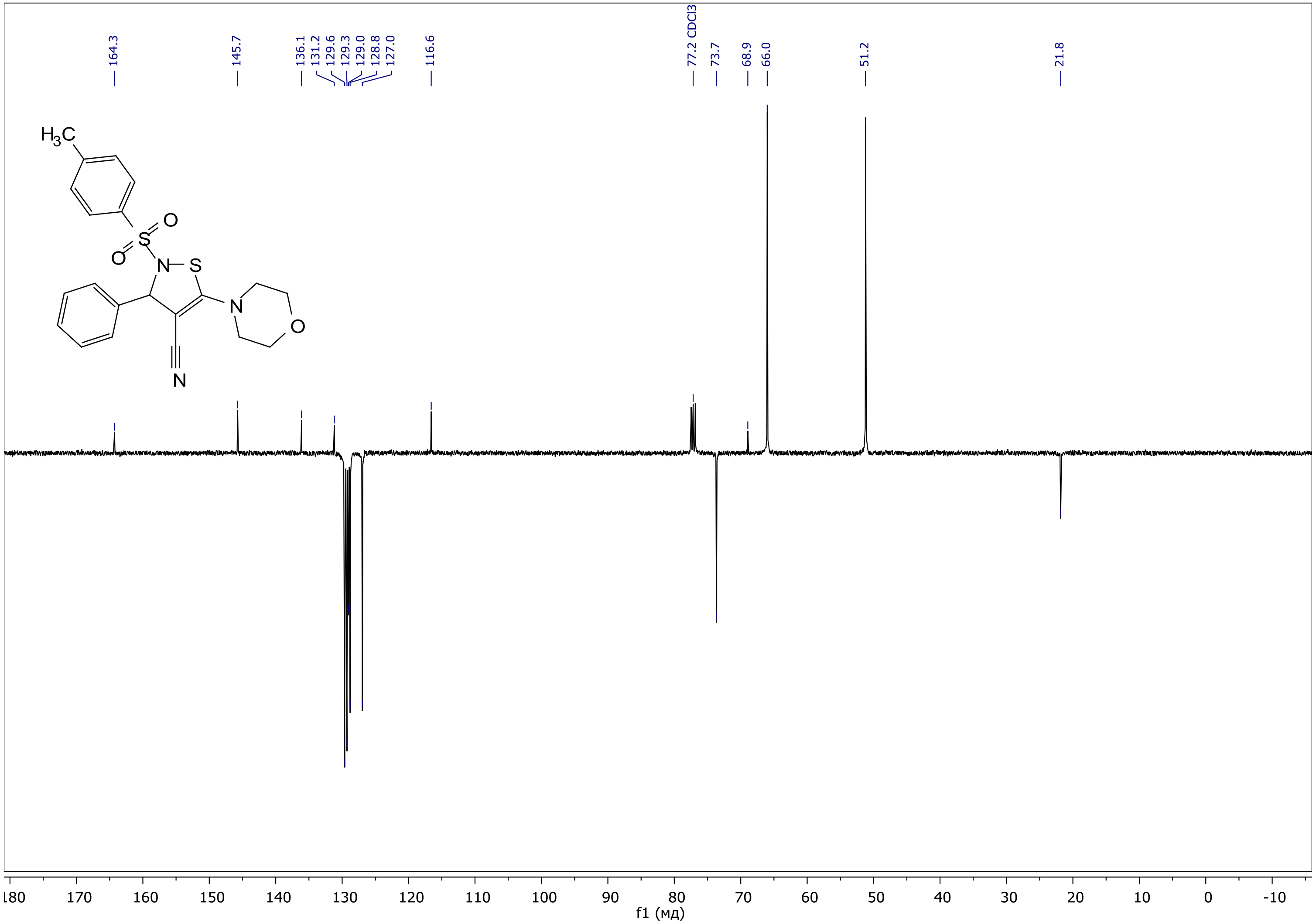

HRMS of **3aa**

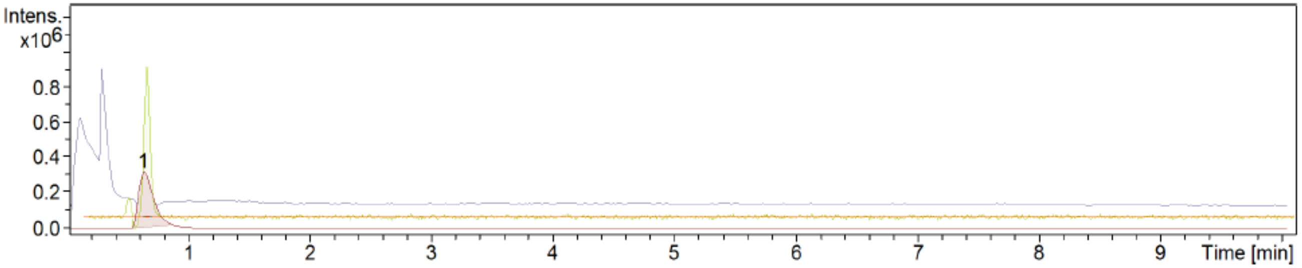

#	RT [min]	Area	Int. Type	I	S/N	Chromatogram	Max. m/z	FWHM [min]
1	0.6	2565338	Manual	309358	1792.3	EIC 428.1100±0.02 +All MS	428.1099	

Cmpd 1, 0.6 min

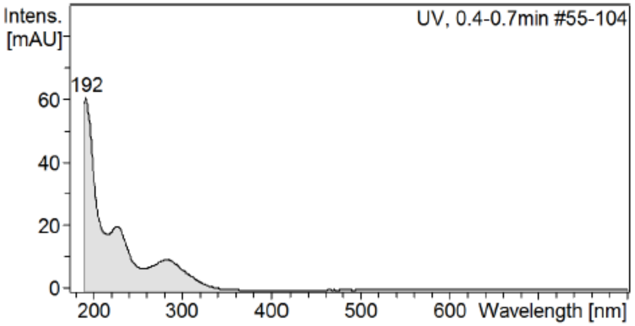

#	Wavelength	Intensity
0	192	60.4

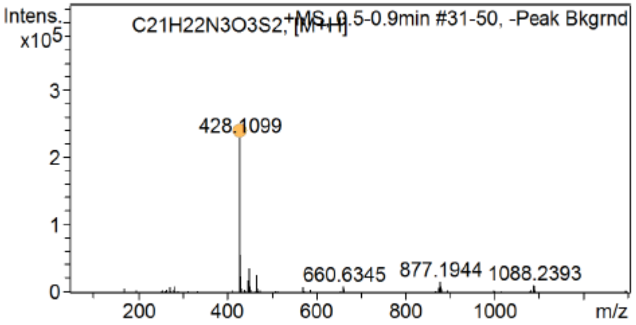

#	m/z	Res.	S/N	I	I %	FWHM
1	428.1099	23016	128879464.0	228119	100.0	0.0186
2	429.1125	19976	30610454.0	54181	23.8	0.0215
3	430.1079	17574	13124928.0	23231	10.2	0.0245
4	447.0837	20913	9885801.0	17498	7.7	0.0214
5	447.5843	20398	4946705.5	8756	3.8	0.0219
6	450.0914	20401	19882364.0	35192	15.4	0.0221
7	466.0654	20919	14484337.0	25638	11.2	0.0223
8	877.1944	22742	8865959.0	15693	6.9	0.0386
9	1088.2393	25762	5895925.0	10436	4.6	0.0422
10	1088.7391	24706	5371830.0	9508	4.2	0.0441

^{1}H NMR ($CDCl_3$) spectrum of **3ab**

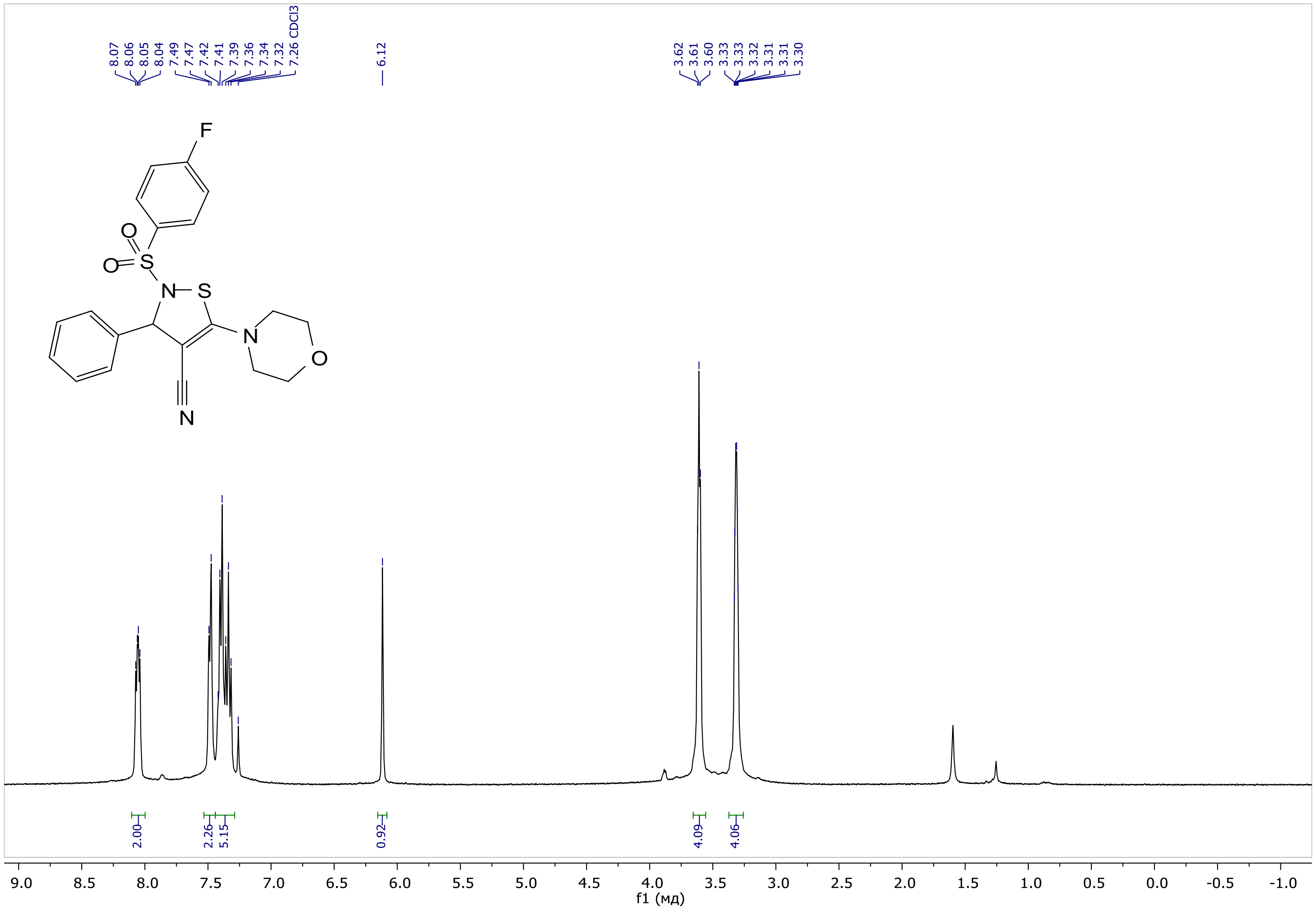

^{19}F NMR ($CDCl_3$) spectrum of **3ab**

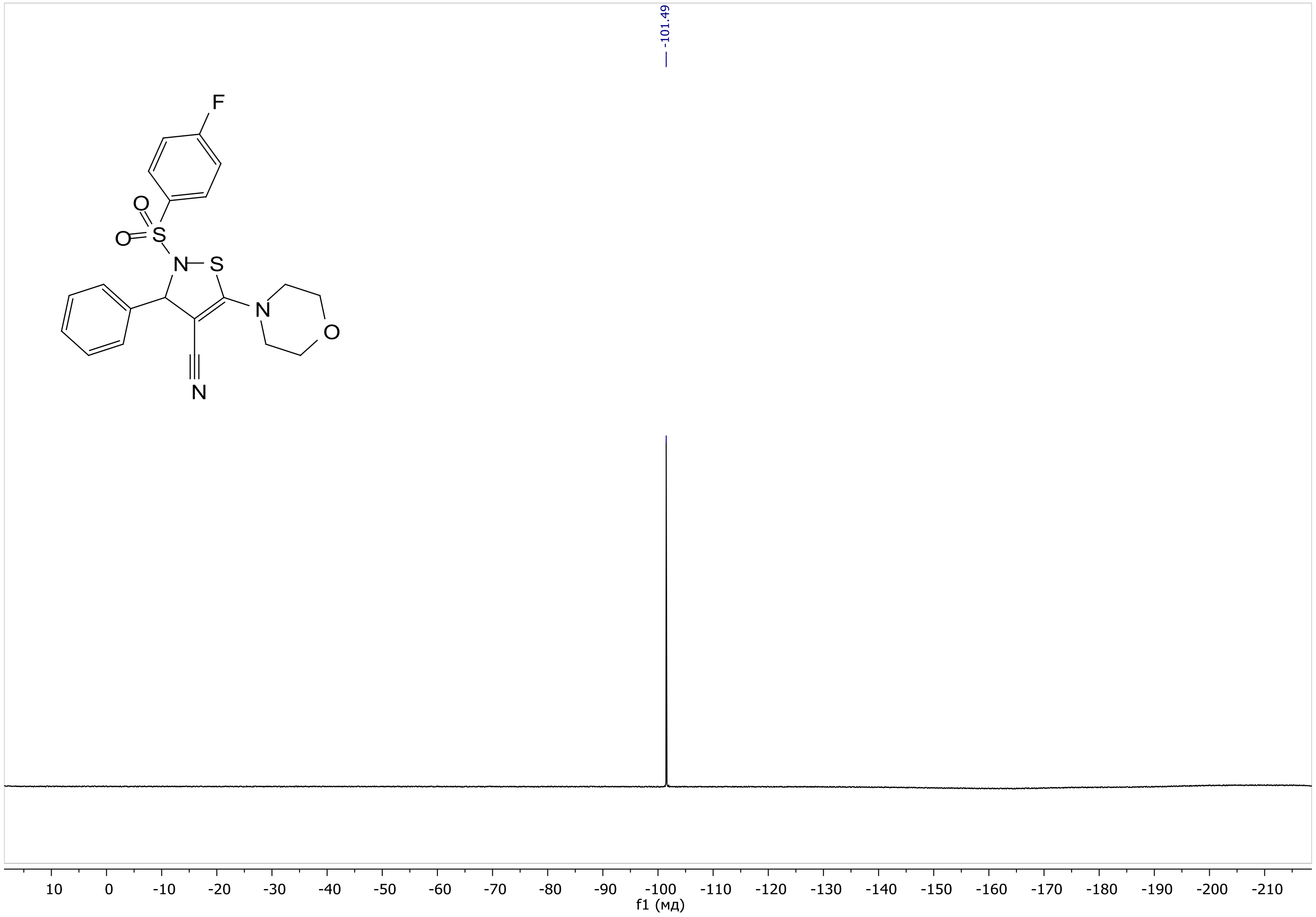

^{13}C NMR (CDCl$_3$) spectrum of **3ab**

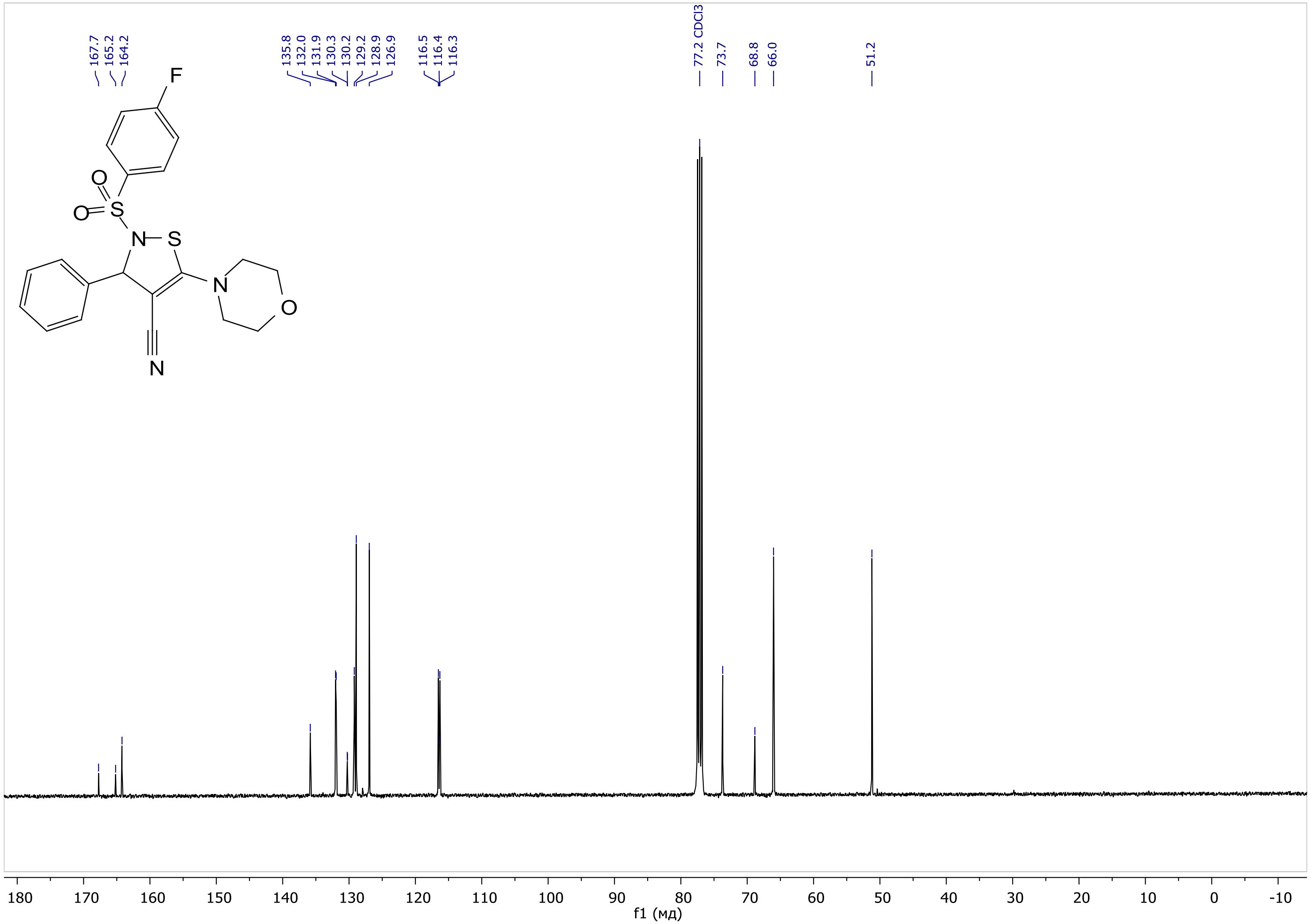

HRMS of **3ab**

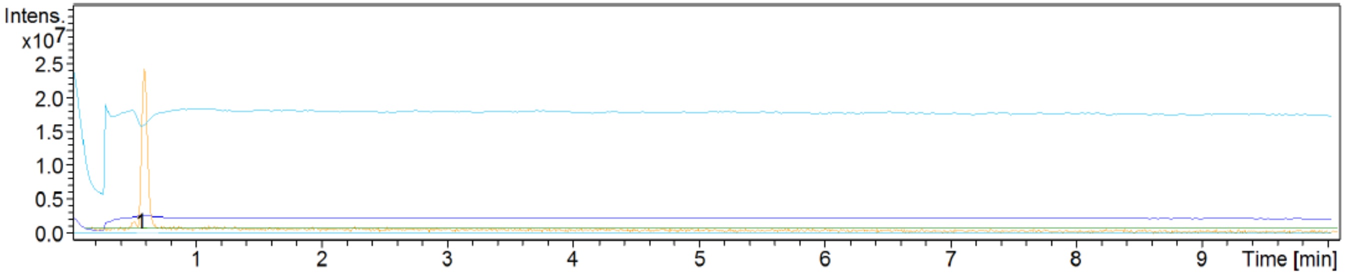

#	RT [min]	Area	Int. Type	I	S/N	Chromatogram	Max. m/z	FWHM [min]
1	0.6	203380	Manual	77368	12.4	EIC C20H18FN3O3S2 [M+H, M+NH4]+ 432.0846; 449.1112 All MS	291.1300	

Cmpd 1, 0.6 min

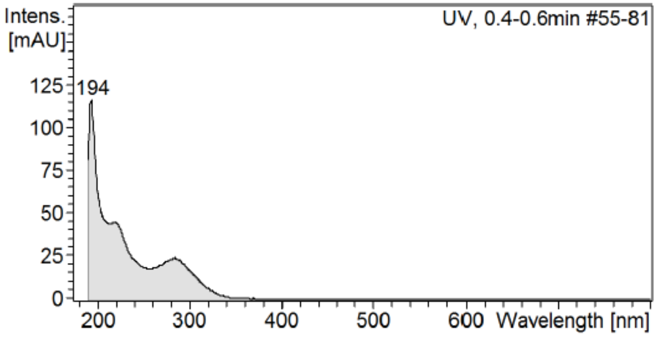

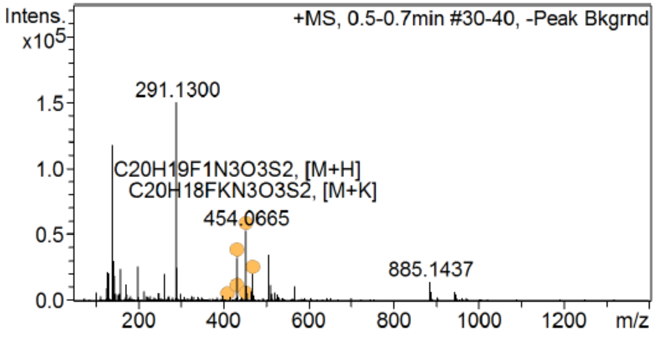

#	Wavelength	Intensity
0	194	115.5

#	m/z	Res.	S/N	I	I %	FWHM
1	128.1434	17366	1741176.0	21421	14.3	0.0074
2	139.1231	20703	9553919.0	117536	78.5	0.0067
3	142.1589	18244	2498196.8	30734	20.5	0.0078
4	158.1537	18186	1960475.1	24119	16.1	0.0087
5	199.1439	19525	2093463.8	25755	17.2	0.0102
6	291.1300	29033	12170005.0	149720	100.0	0.0100
7	292.1329	21848	2008376.1	24708	16.5	0.0134
8	432.0844	20308	2672344.0	32876	22.0	0.0213
9	454.0665	20988	4325999.5	53220	35.5	0.0216
10	507.3282	19672	2838541.3	34921	23.3	0.0258

^{1}H NMR ($CDCl_3$) spectrum of **3ac**

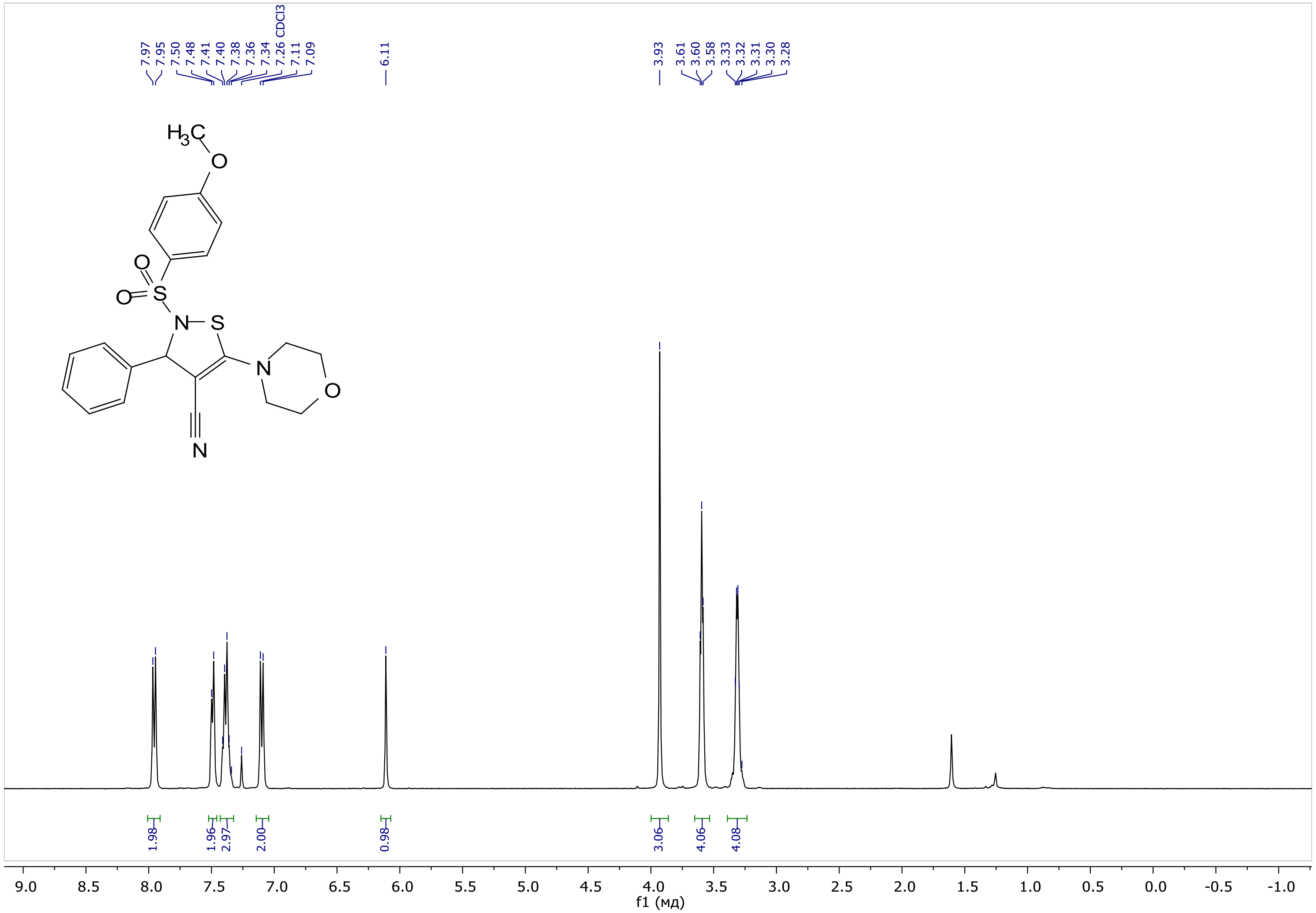

^{13}C NMR ($CDCl_3$) spectrum of **3ac**

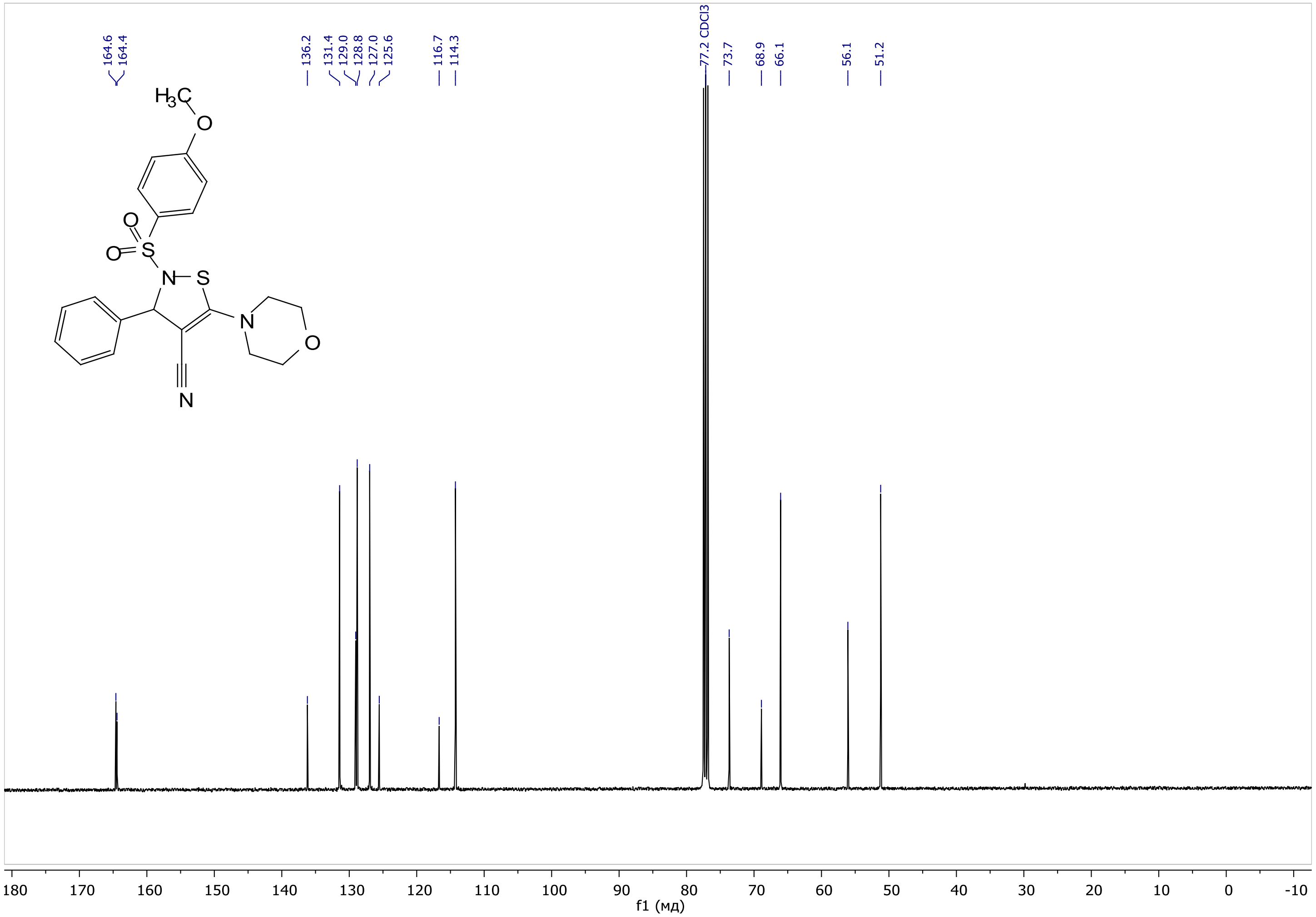

HRMS of **3ac**

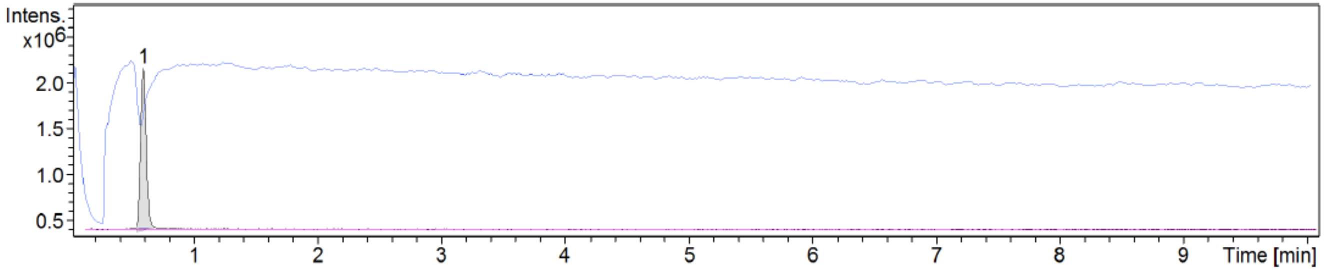

#	RT [min]	Area	Int. Type	I	S/N	Chromatogram	Max. m/z	FWHM [min]
1	0.6	403660	Manual	121236	282.9	UV Chromatogram, 190-800 nm	444.1050	

Cmpd 1, 0.6 min

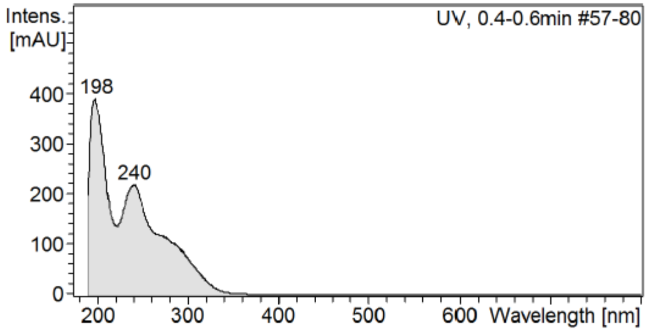

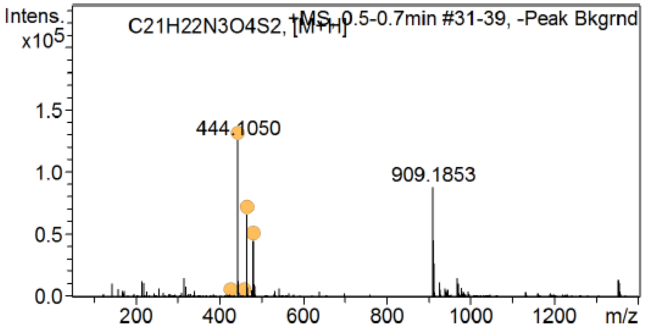

#	Wavelength	Intensity
0	198	387.5
1	240	217.0

#	m/z	Res.	S/N	I	I %	FWHM
1	316.3203	20538	1227602.0	14843	11.9	0.0154
2	444.1050	22934	10334461.0	124951	100.0	0.0194
3	445.1076	20293	2541050.0	30723	24.6	0.0219
4	466.0864	21282	5452649.0	65926	52.8	0.0219
5	467.0891	19810	1410503.0	17054	13.6	0.0236
6	482.0603	20799	3710549.0	44863	35.9	0.0232
7	909.1853	25666	7262980.0	87814	70.3	0.0354
8	910.1874	23292	3765613.3	45529	36.4	0.0391
9	911.1845	21354	2179008.3	26346	21.1	0.0427
10	967.1430	21640	1259315.4	15226	12.2	0.0447

^{1}H NMR ($CDCl_3$) spectrum of **3ad**

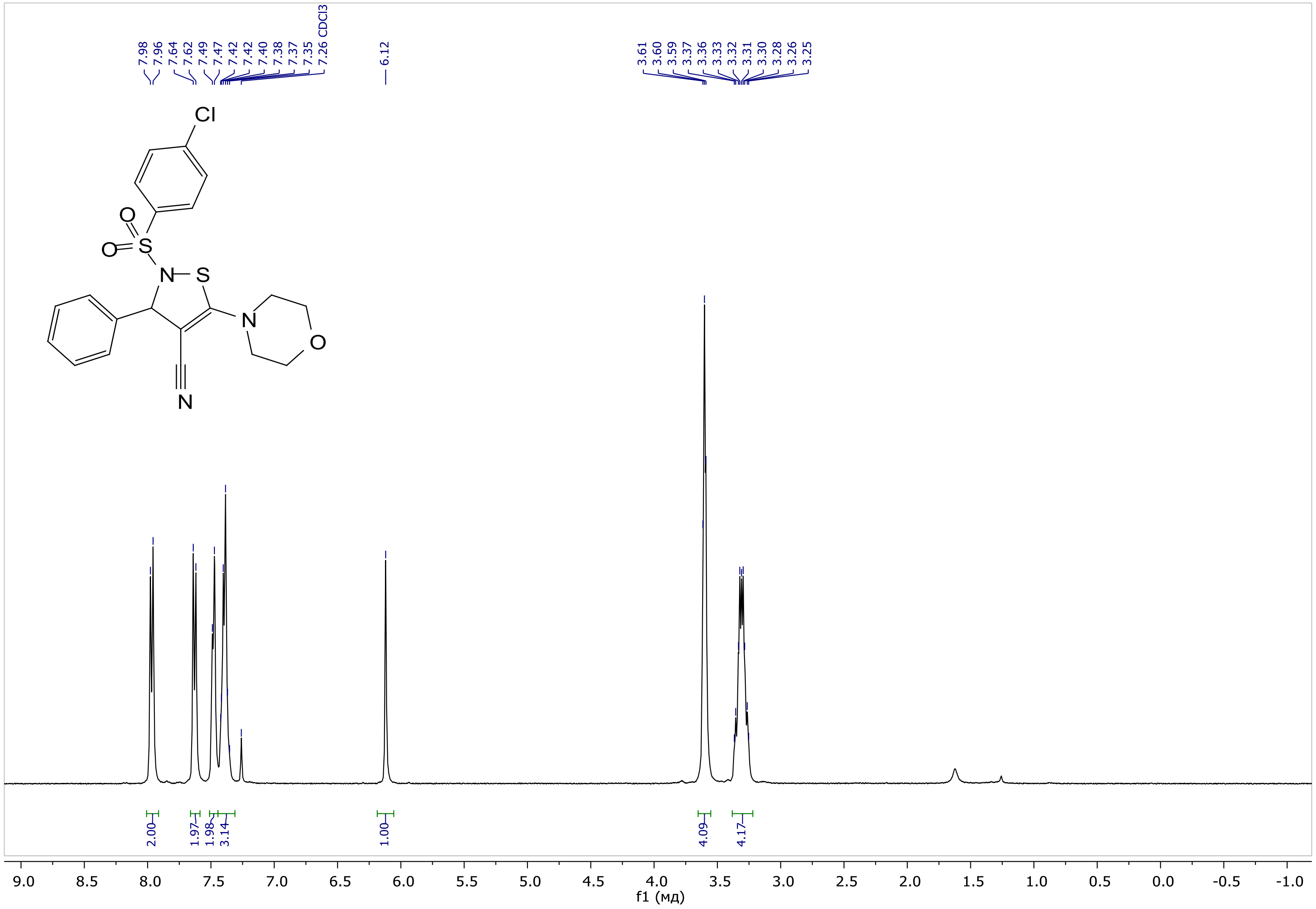

^{13}C NMR ($CDCl_3$) spectrum of **3ad**

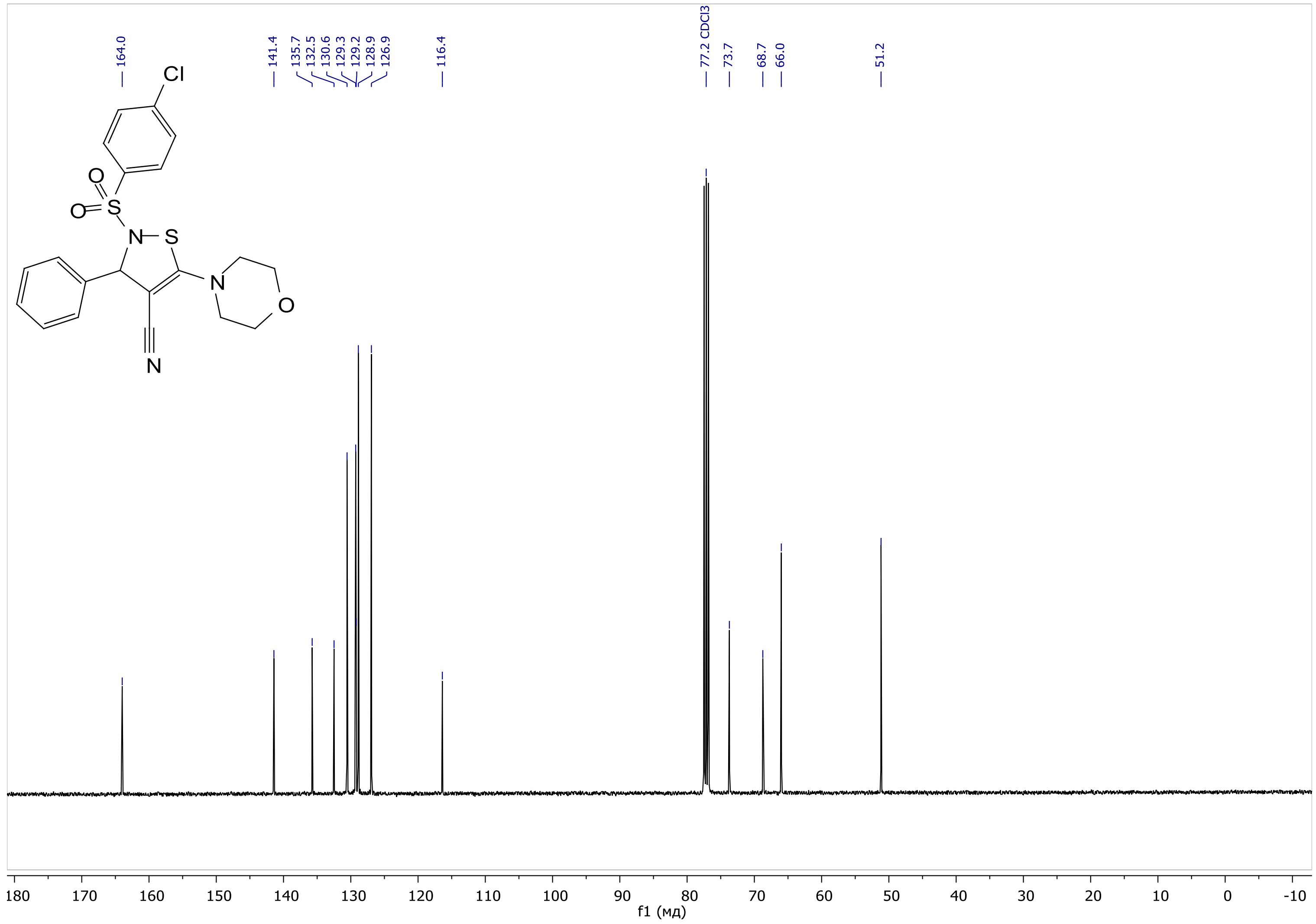

HRMS of **3ad**

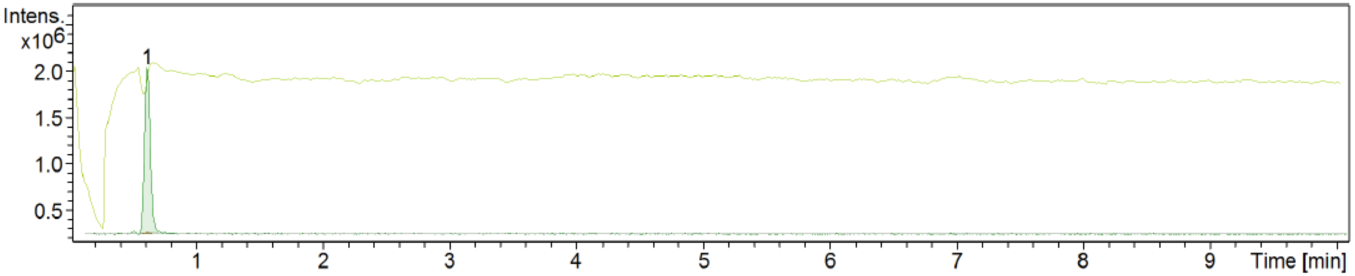

#	RT [min]	Area	Int. Type	I	S/N	Chromatogram	Max. m/z	FWHM [min]
1	0.6	257070	Manual	78451	184.4	UV Chromatogram, 190-800 nm	139.1232	

Cmpd 1, 0.6 min

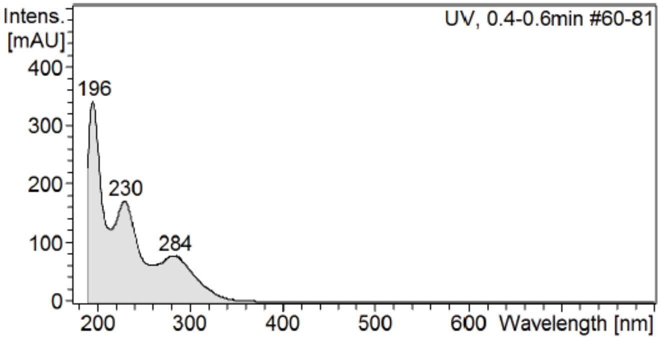

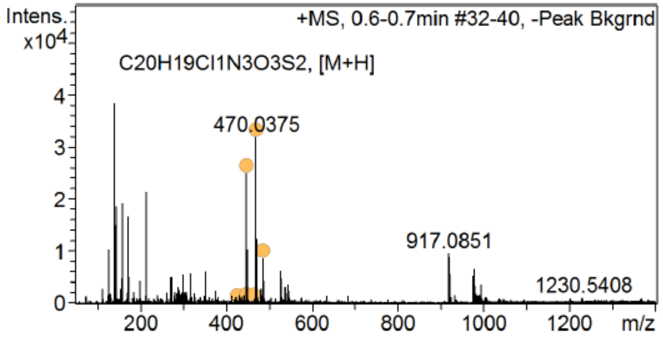

#	Wavelength	Intensity
0	196	340.3
1	230	171.5
2	284	78.1

#	m/z	Res.	S/N	I	I %	FWHM
1	139.1232	19729	5283883.0	38297	100.0	0.0071
2	142.1590	18635	2081020.3	15083	39.4	0.0076
3	143.1181	16569	2571527.0	18638	48.7	0.0086
4	158.1538	18410	2645341.3	19173	50.1	0.0086
5	172.1331	17904	2317819.8	16799	43.9	0.0096
6	213.1227	17851	2956544.0	21429	56.0	0.0119
7	448.0551	20718	3446639.5	24981	65.2	0.0216
8	450.0528	19281	1437743.1	10421	27.2	0.0233
9	470.0375	21134	4386319.0	31792	83.0	0.0222
10	472.0346	19605	1726835.1	12516	32.7	0.0241

^{1}H NMR (DMSO-d_6) spectrum of **3ae**

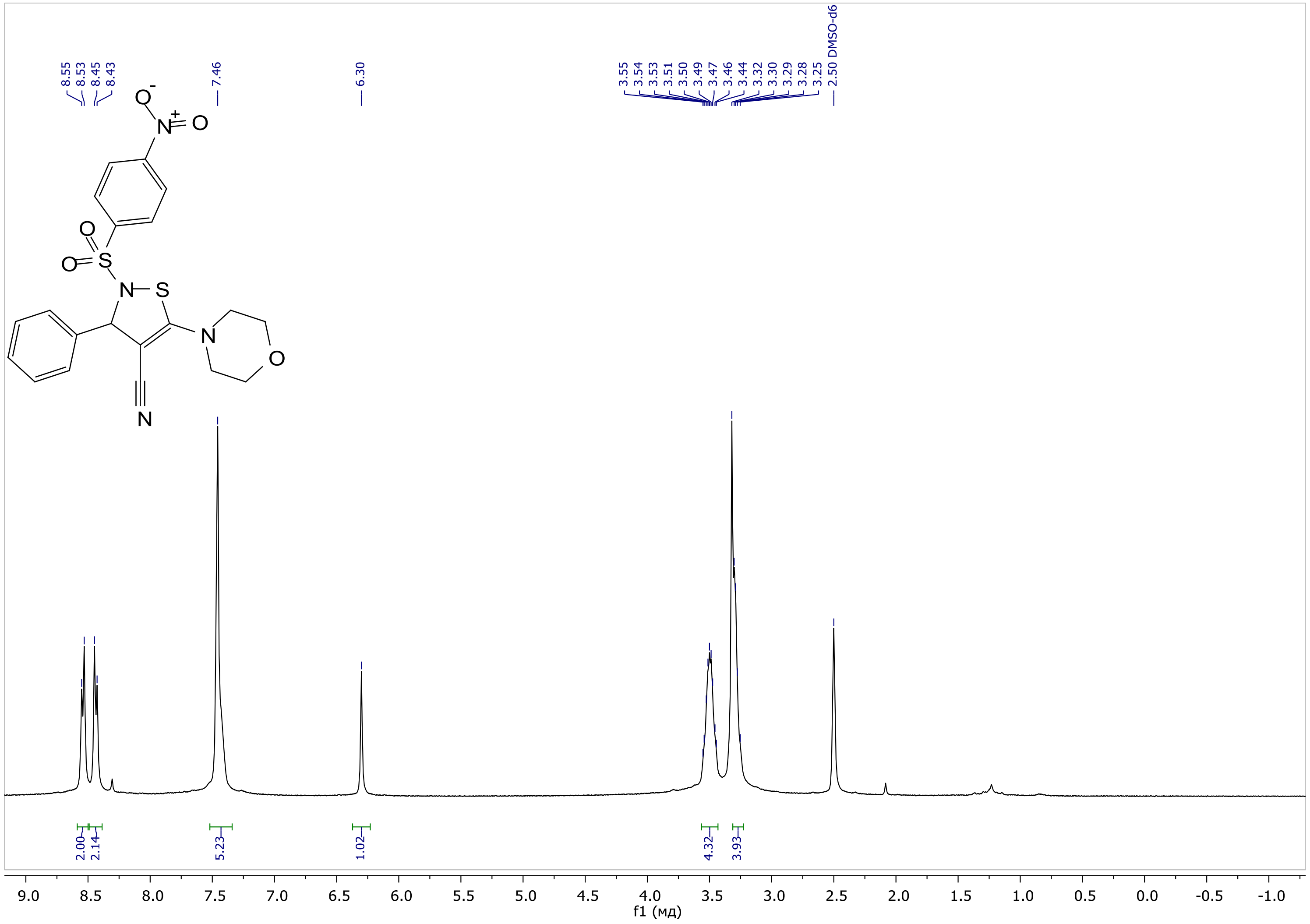

^{13}C NMR (DMSO-d_6) spectrum of **3ae**

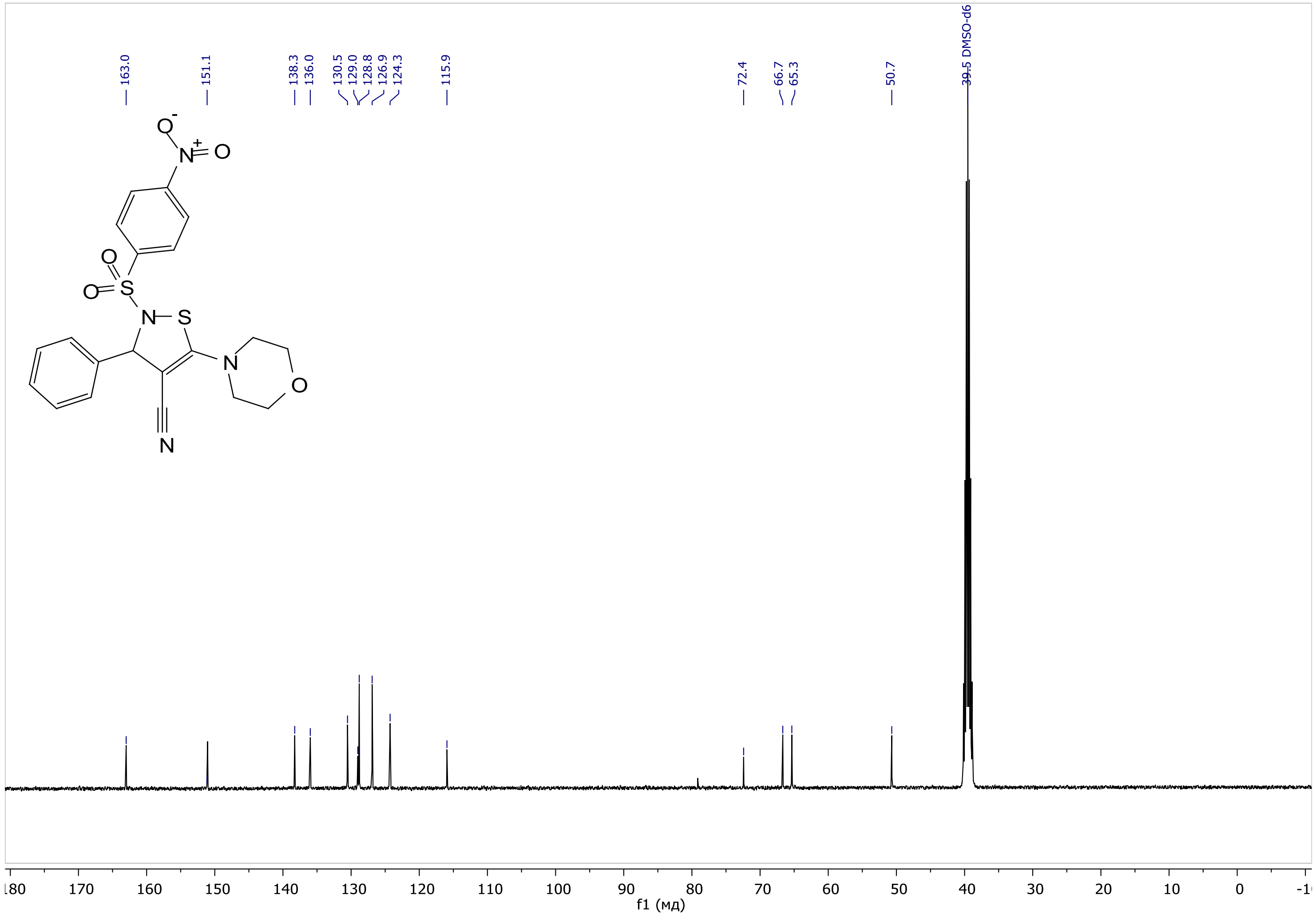

HRMS of **3ae**

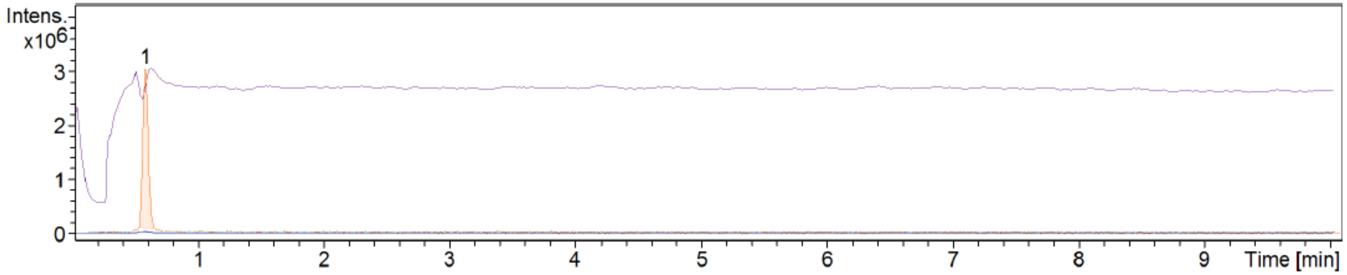

#	RT [min]	Area	Int. Type	I	S/N	Chromatogram	Max. m/z	FWHM [min]
1	0.6	172609	Manual	56511	122.5	UV Chromatogram, 190-800 nm	139.1230	

Cmpd 1, 0.6 min

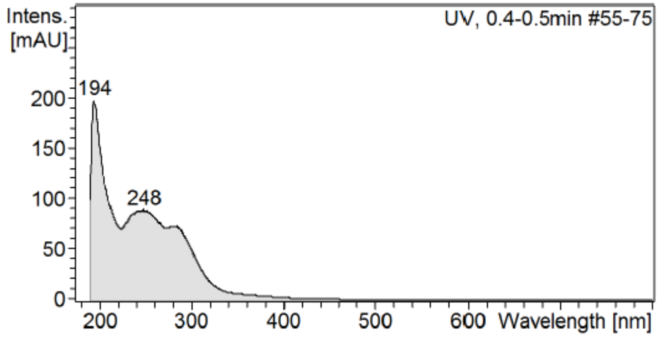

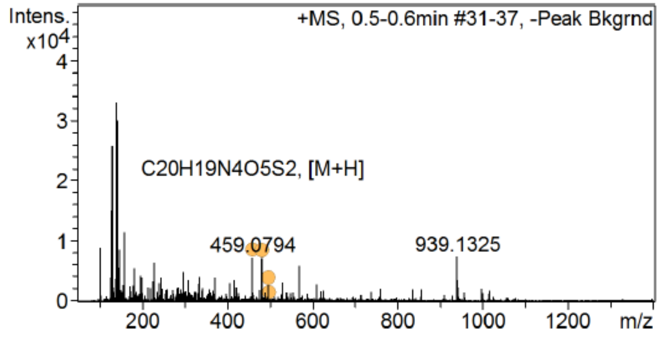

#	Wavelength	Intensity
0	194	196.8
1	248	88.1

#	m/z	Res.	S/N	I	I %	FWHM
1	102.1275	17196	3022350.3	8918	27.1	0.0059
2	128.1434	18031	5108408.5	15073	45.8	0.0071
3	130.1591	18518	8719433.0	25728	78.1	0.0070
4	139.1230	19362	11164568.0	32943	100.0	0.0072
5	142.1590	19005	10164604.0	29992	91.0	0.0075
6	146.1536	17303	2929789.0	8645	26.2	0.0084
7	158.1537	17964	3890437.5	11479	34.8	0.0088
8	459.0794	20841	2510372.0	7407	22.5	0.0220
9	481.0612	20302	2467577.3	7281	22.1	0.0237
10	939.1325	22608	2525650.8	7452	22.6	0.0415

^{1}H NMR ($CDCl_3$) spectrum of **3af**

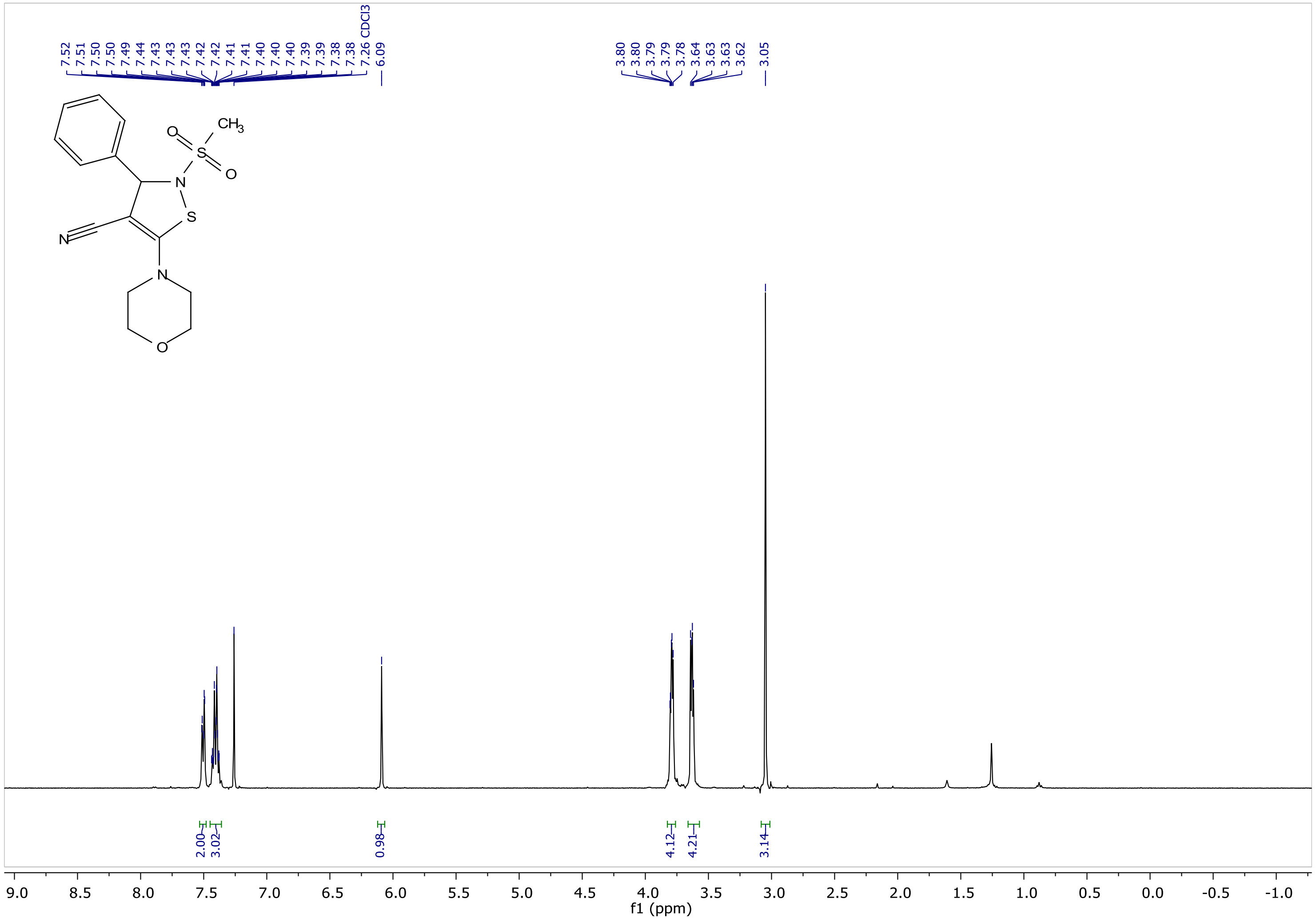

^{13}C NMR ($CDCl_3$) spectrum of **3af**

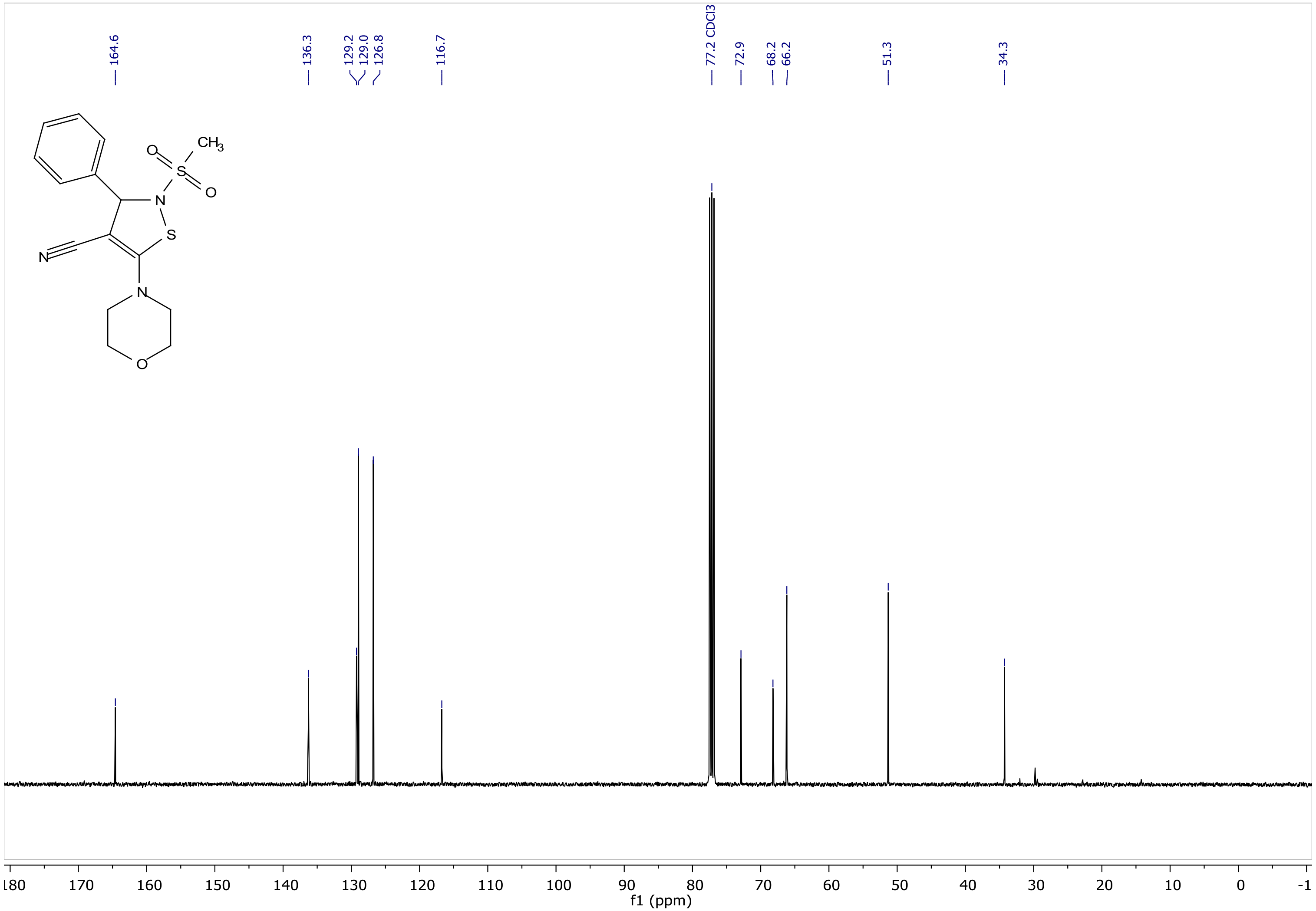

HRMS of **3af**

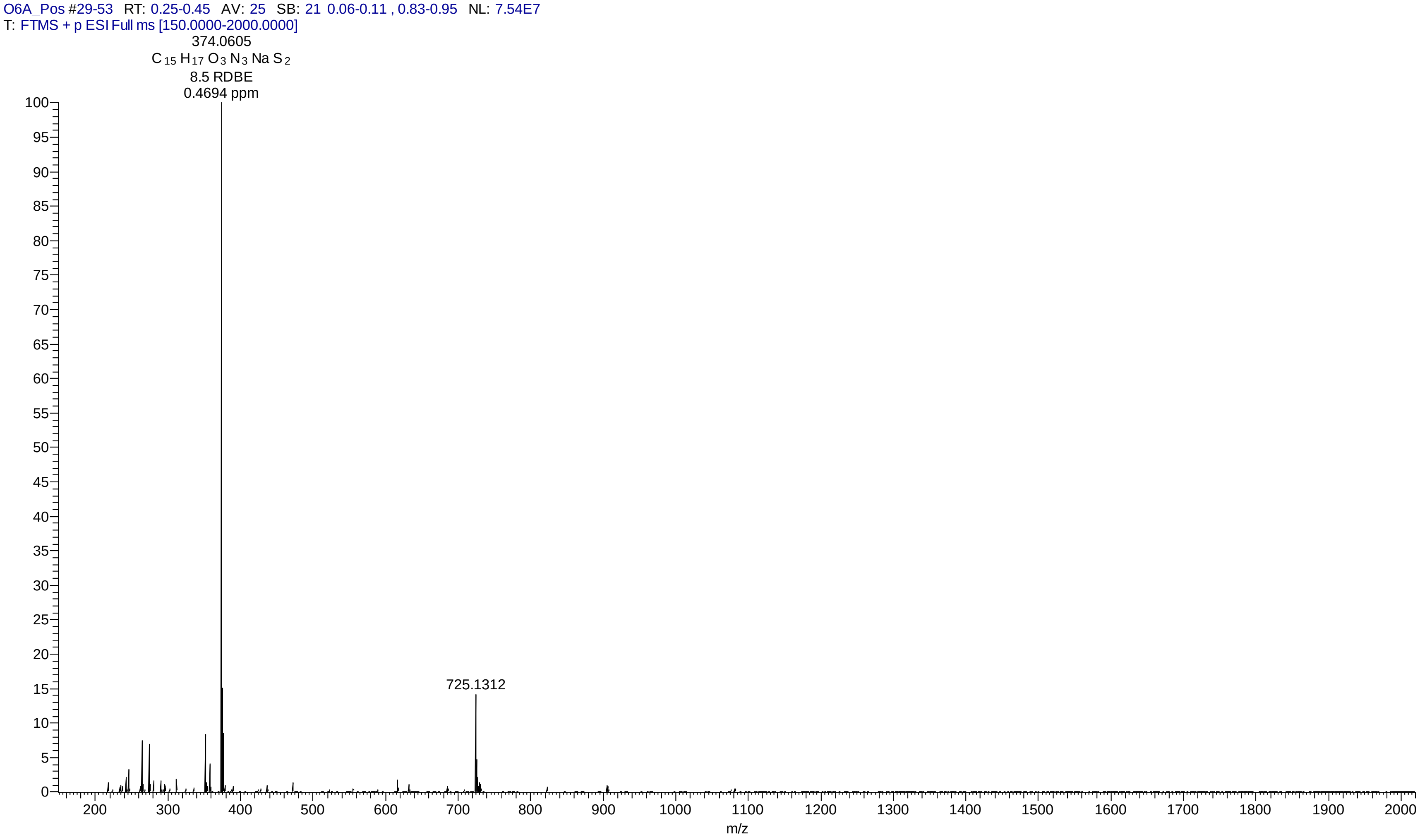

O6A_Pos #29-53 RT: 0.25-0.45 AV: 25 SB: 21 0.06-0.11 , 0.83-0.95 NL: 7.54E7
T: FTMS + p ESI Full ms [150.0000-2000.0000]
374.0605
C 15 H 17 O 3 N 3 Na S 2
8.5 RDBE
0.4694 ppm
725.1312
m/z

^{1}H NMR ($CDCl_3$) spectrum of **3ba**

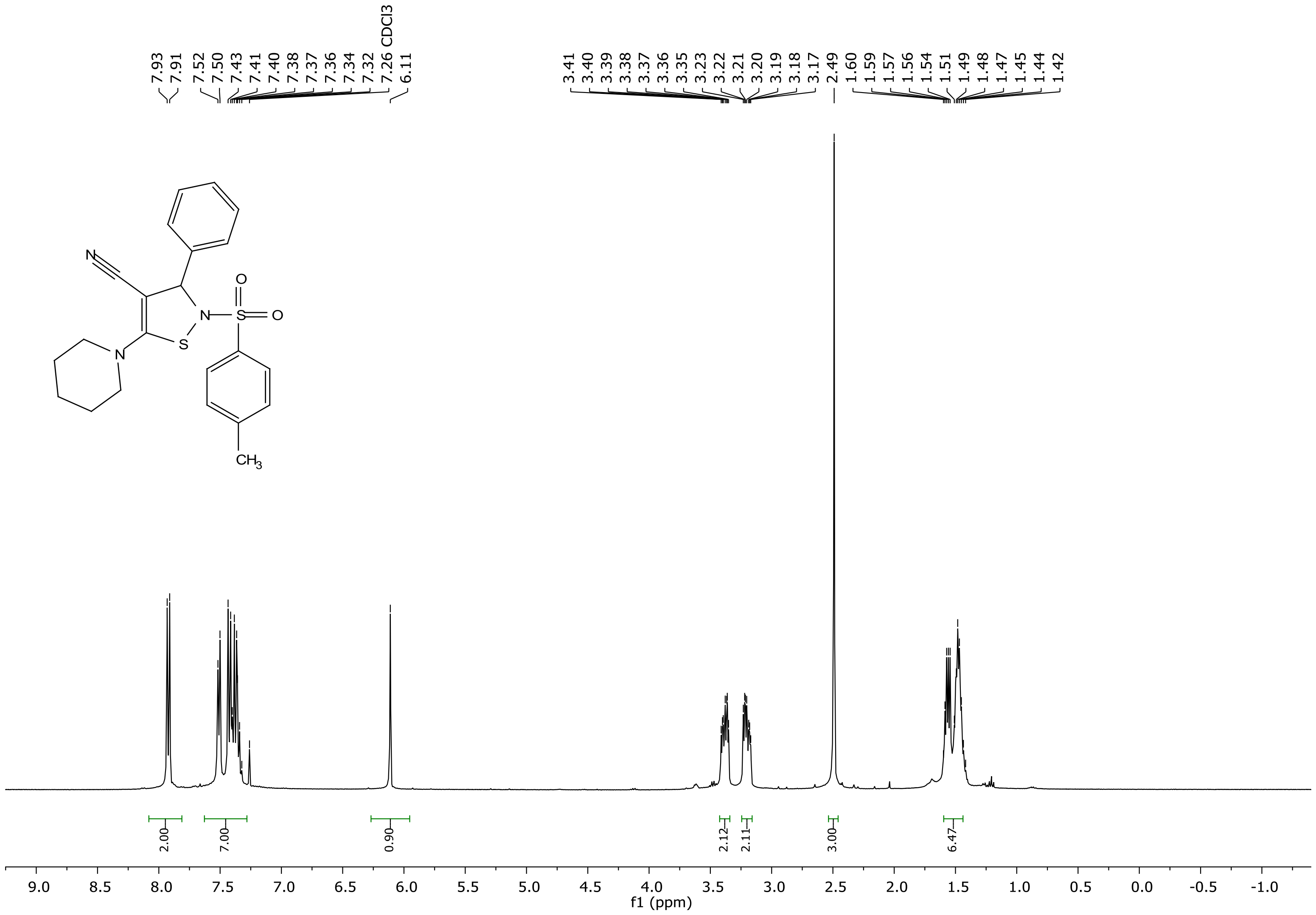

^{13}C NMR (CDCl$_3$) spectrum of **3ba**

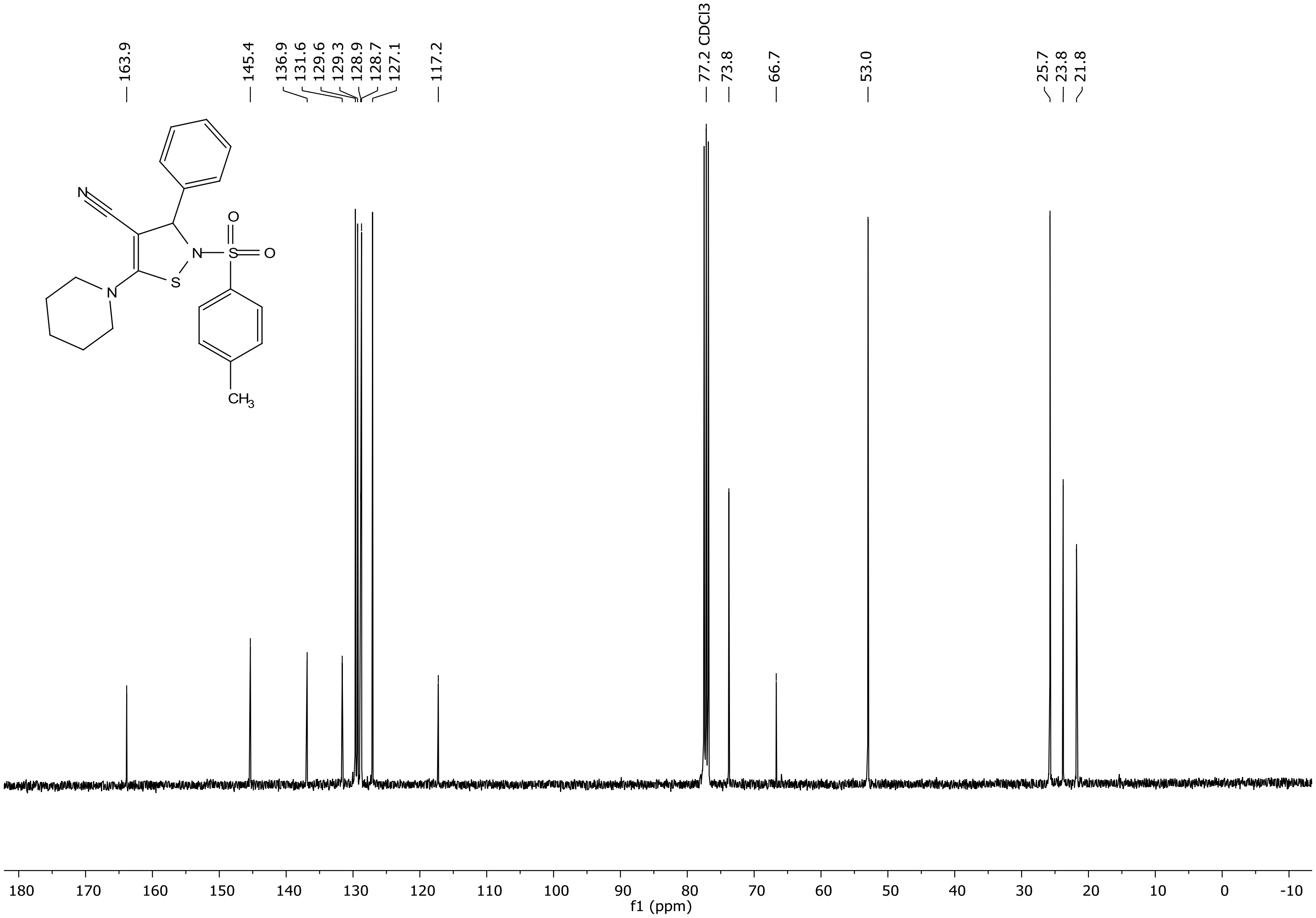

HRMS of **3ba**

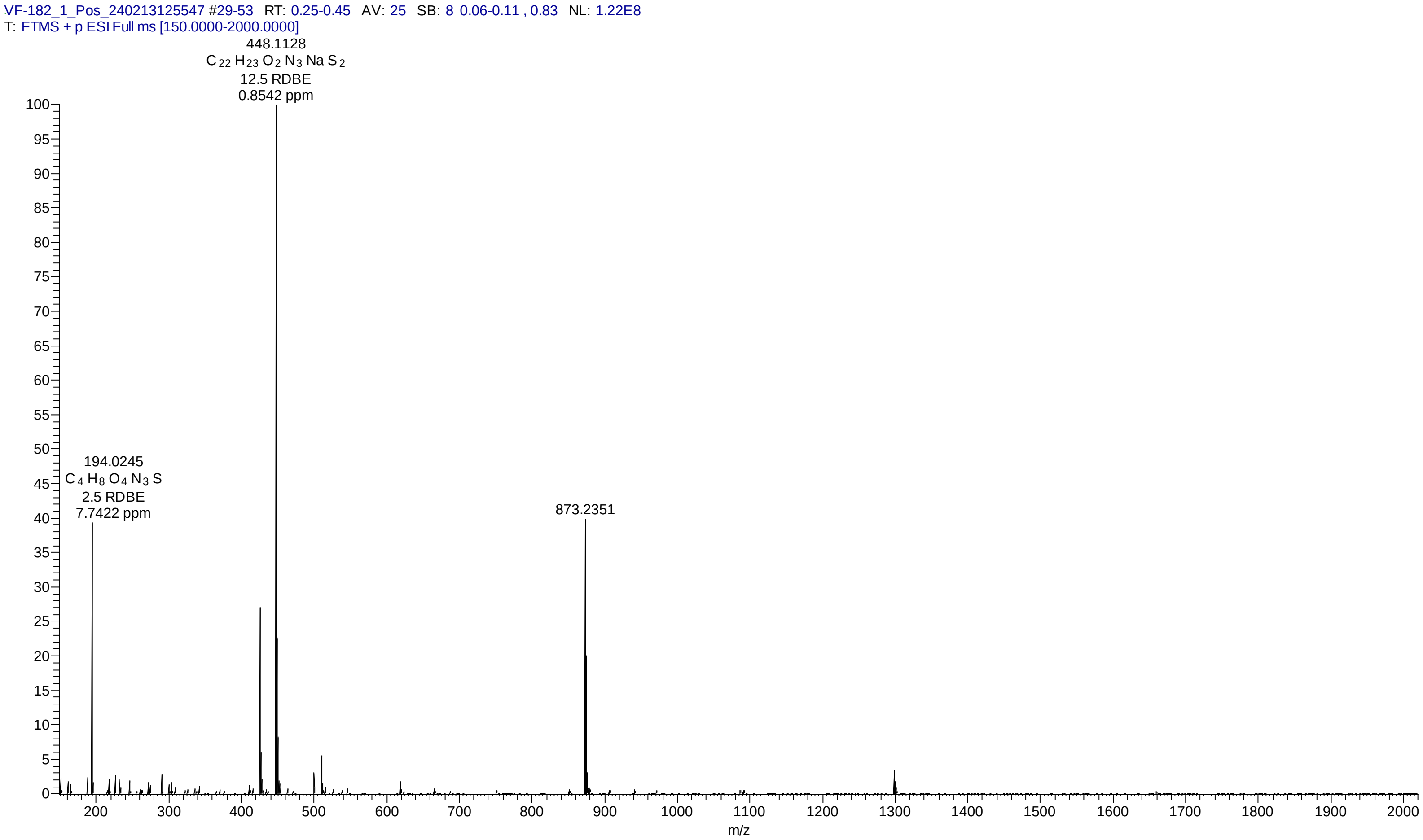

VF-182_1_Pos_240213125547 #29-53 RT: 0.25-0.45 AV: 25 SB: 8 0.06-0.11 , 0.83 NL: 1.22E8
T: FTMS + p ESI Full ms [150.0000-2000.0000]
448.1128
C 22 H 23 O 2 N 3 Na S 2
12.5 RDBE
0.8542 ppm
194.0245
C 4 H 8 O 4 N 3 S
2.5 RDBE
7.7422 ppm
873.2351
m/z

^{1}H NMR ($CDCl_3$) spectrum of **3ca**

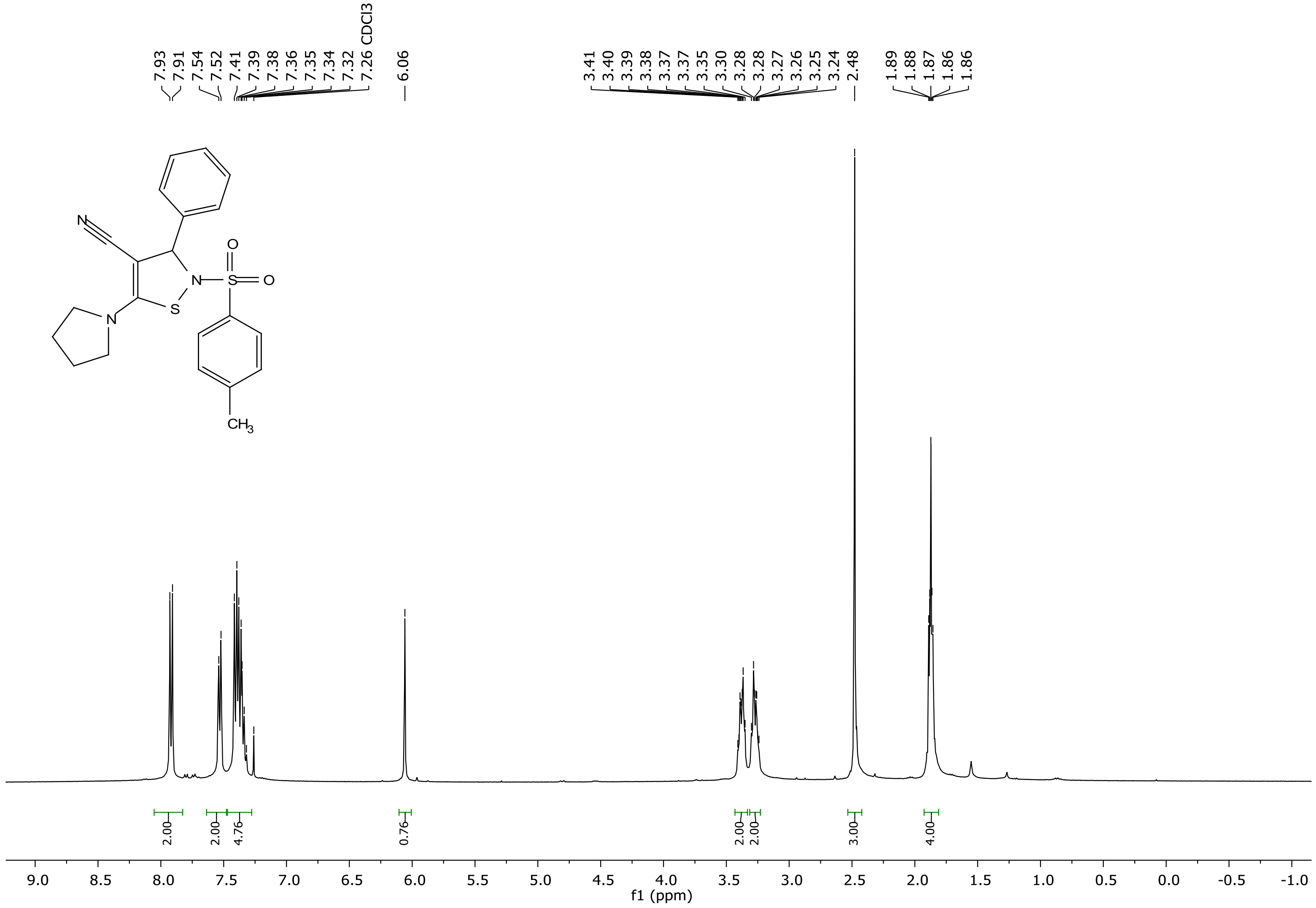

^{13}C NMR (CDCl$_3$) spectrum of **3ca**

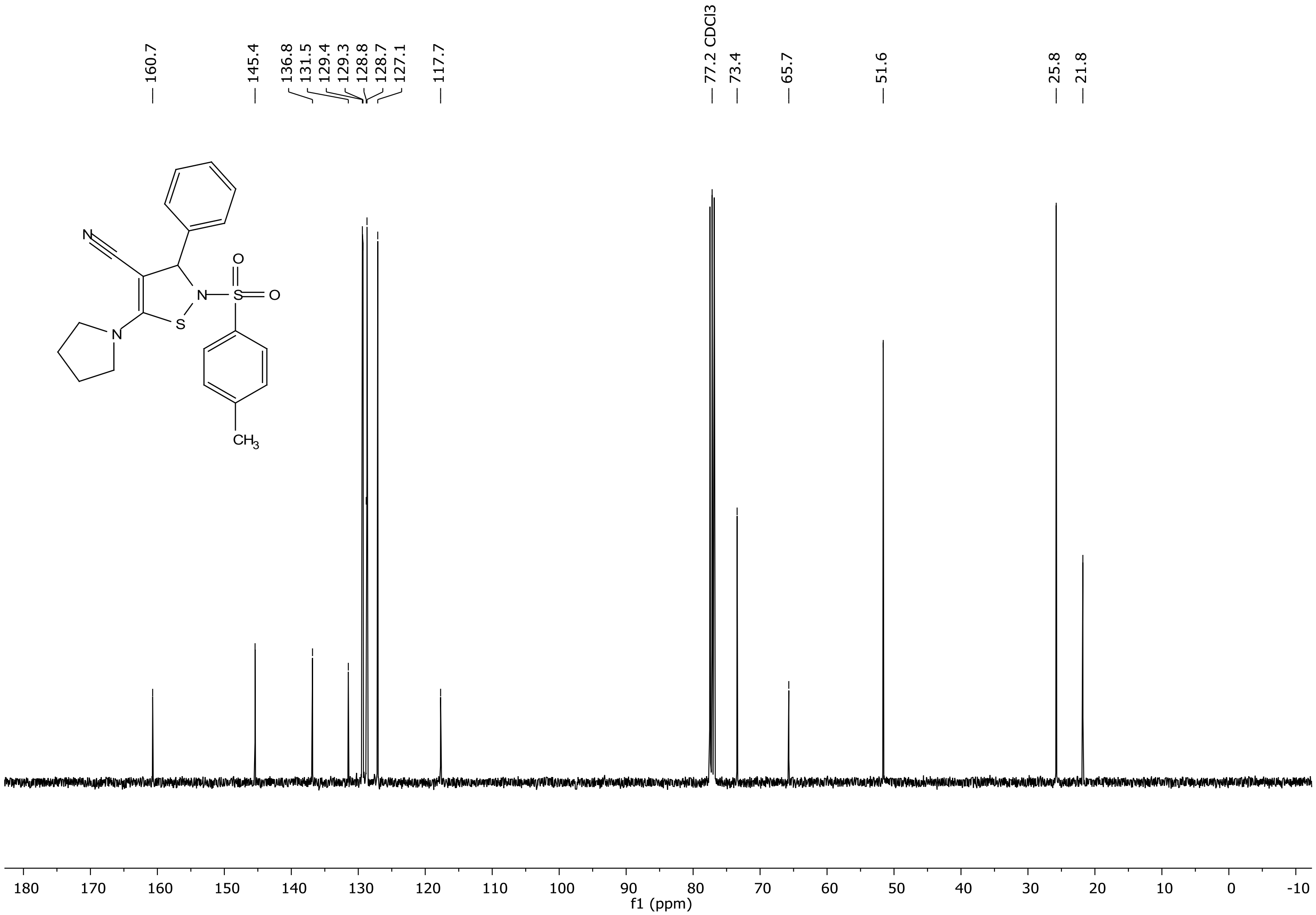

HRMS of **3ca**

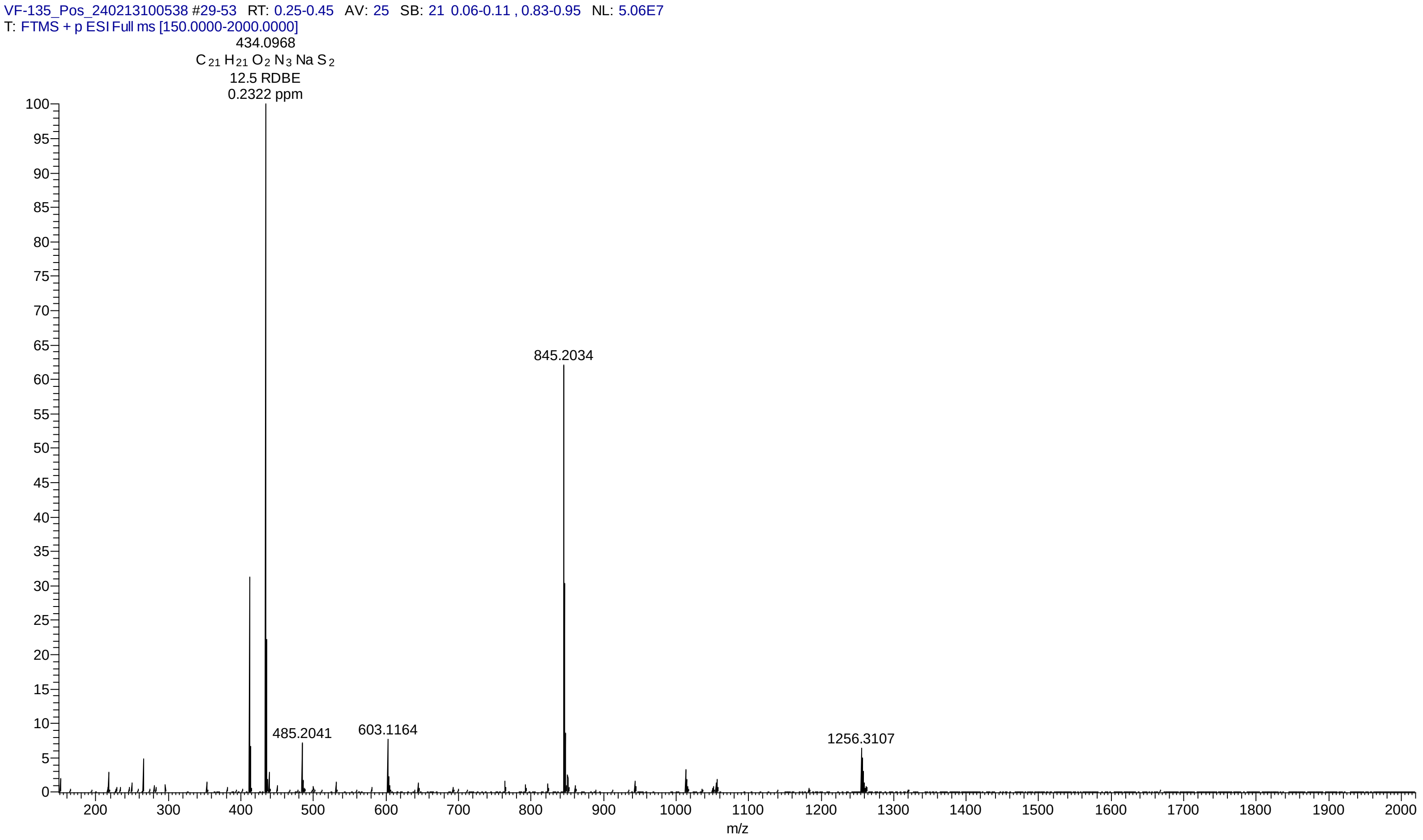

VF-135_Pos_240213100538 #29-53 RT: 0.25-0.45 AV: 25 SB: 21 0.06-0.11 , 0.83-0.95 NL: 5.06E7
T: FTMS + p ESI Full ms [150.0000-2000.0000]
434.0968
C 21 H 21 O 2 N 3 Na S 2
12.5 RDBE
0.2322 ppm
845.2034
485.2041
603.1164
1256.3107
m/z

^{1}H NMR ($CDCl_3$) spectrum of **3da**

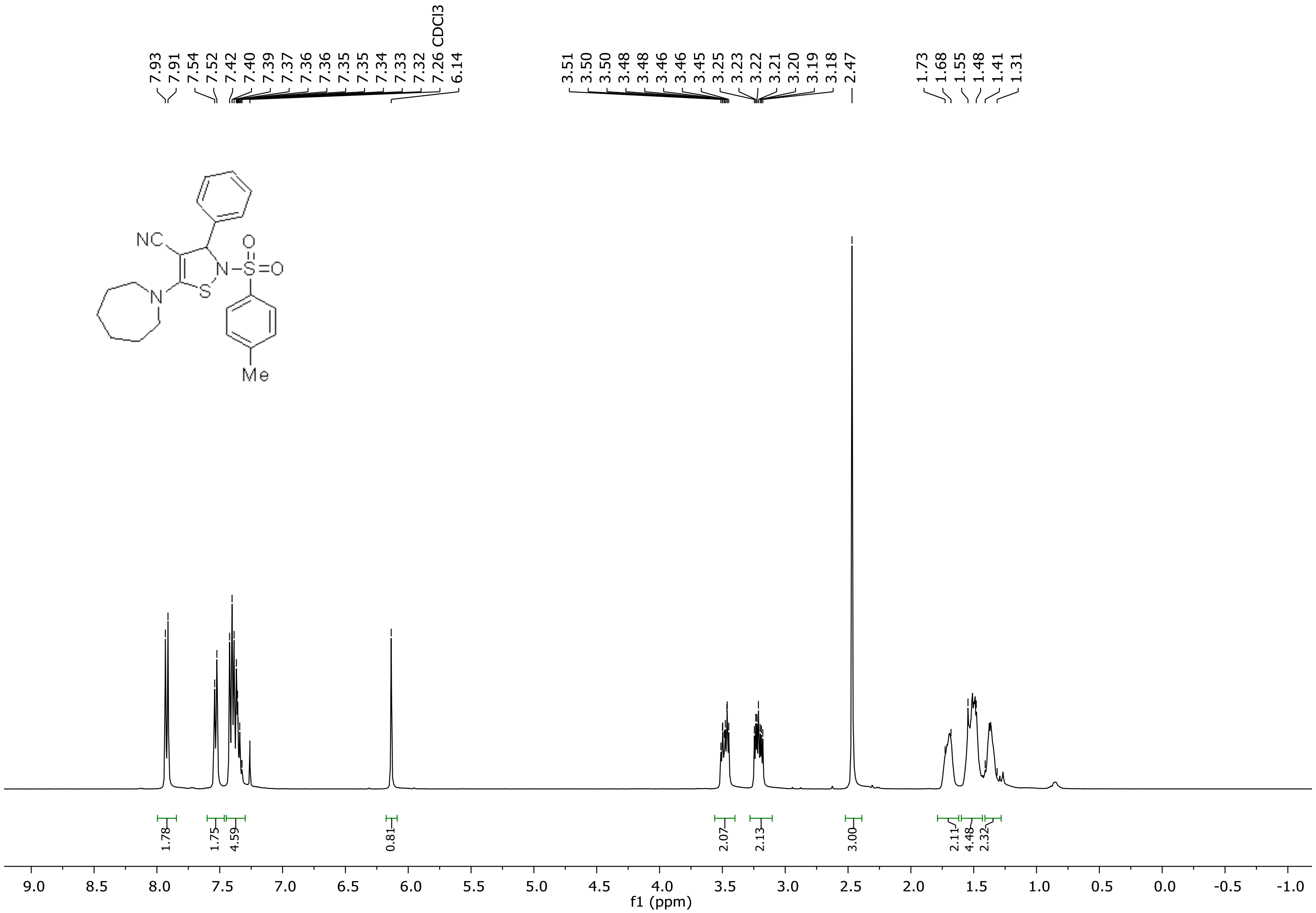

^{13}C NMR ($CDCl_3$) spectrum of **3da**

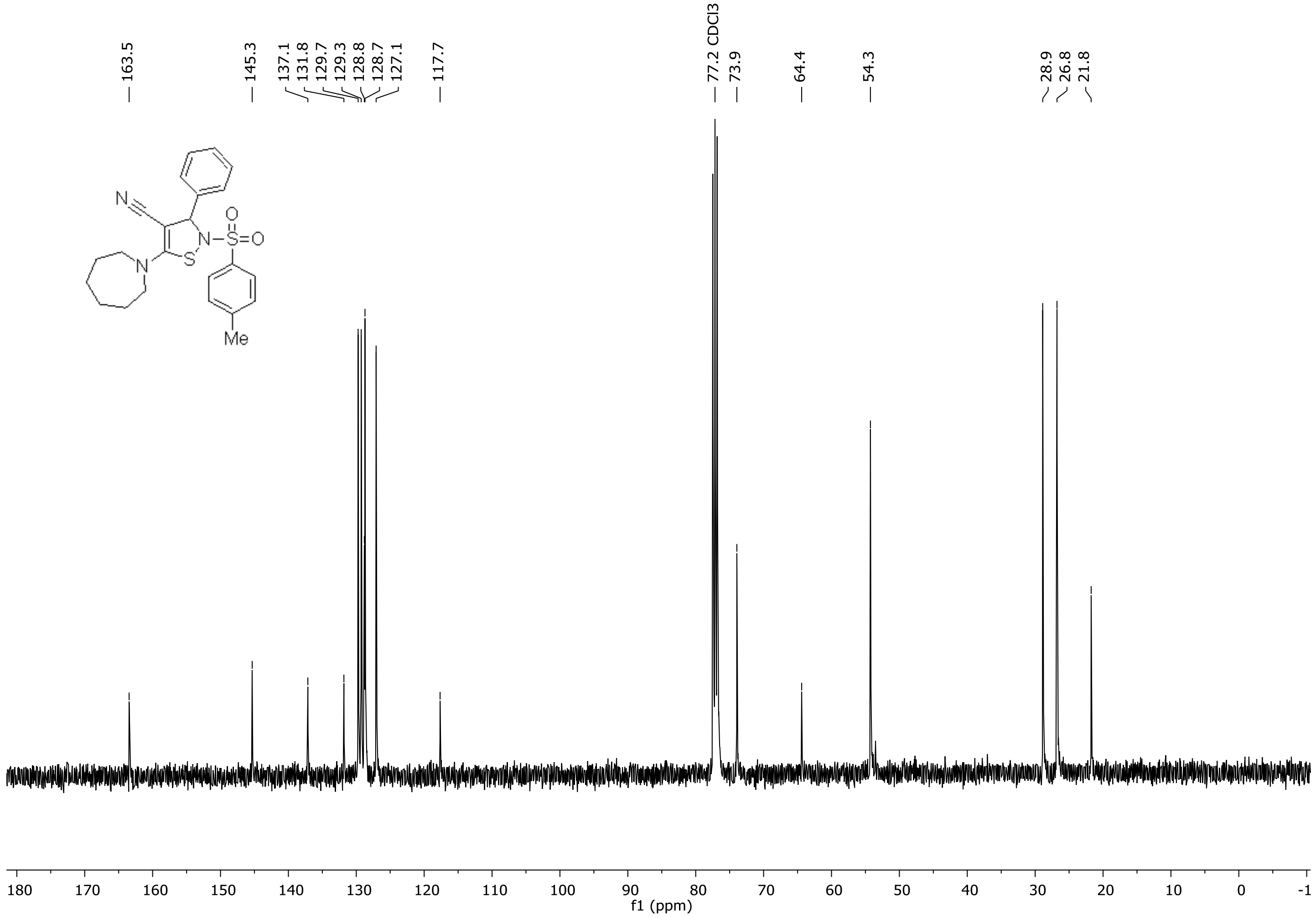

HRMS of **3da**

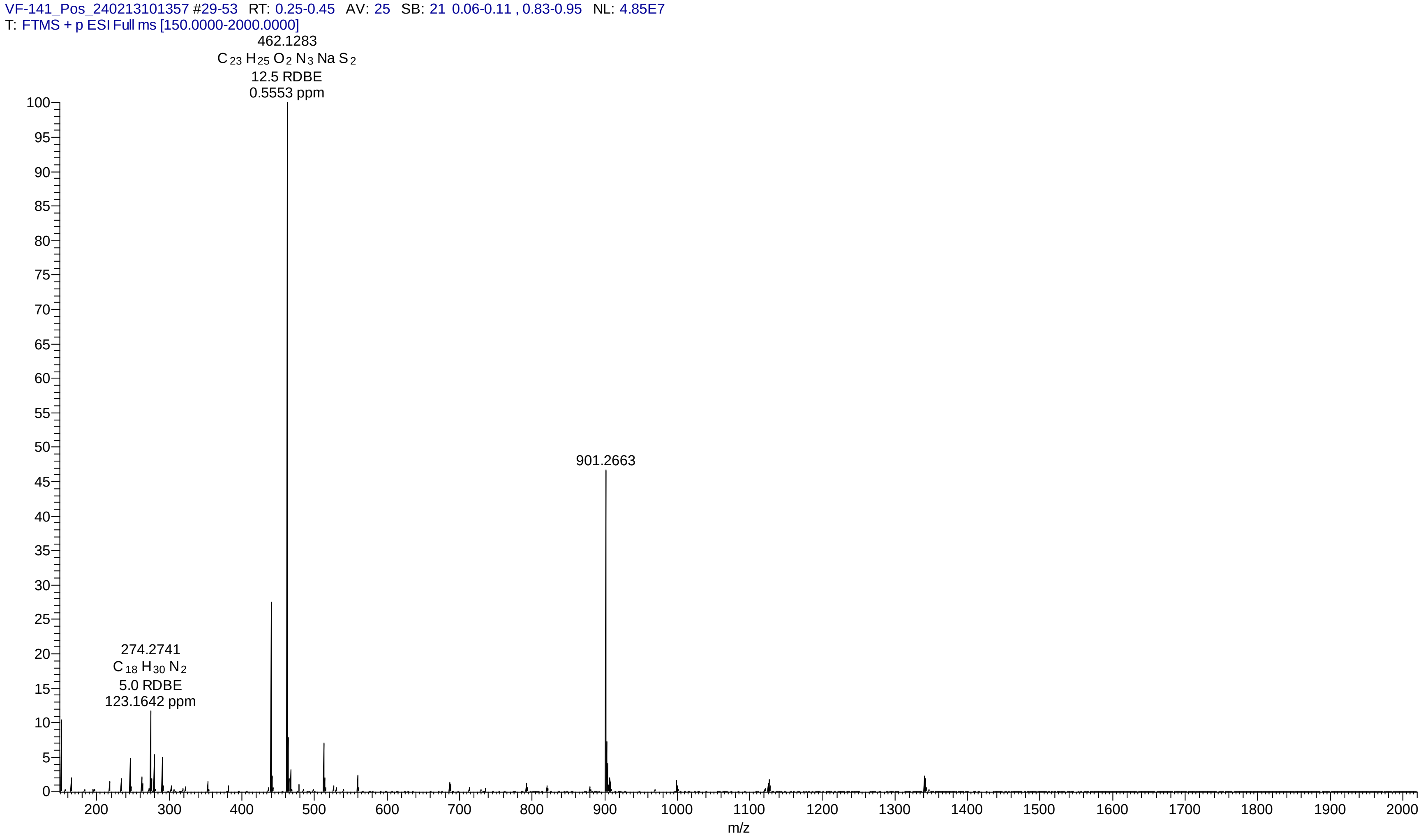

VF-141_Pos_240213101357 #29-53 RT: 0.25-0.45 AV: 25 SB: 21 0.06-0.11 , 0.83-0.95 NL: 4.85E7
T: FTMS + p ESI Full ms [150.0000-2000.0000]
462.1283
C 23 H 25 O 2 N 3 Na S 2
12.5 RDBE
0.5553 ppm
901.2663
274.2741
C 18 H 30 N 2
5.0 RDBE
123.1642 ppm
m/z

^{1}H NMR ($CDCl_3$) spectrum of **3ea**

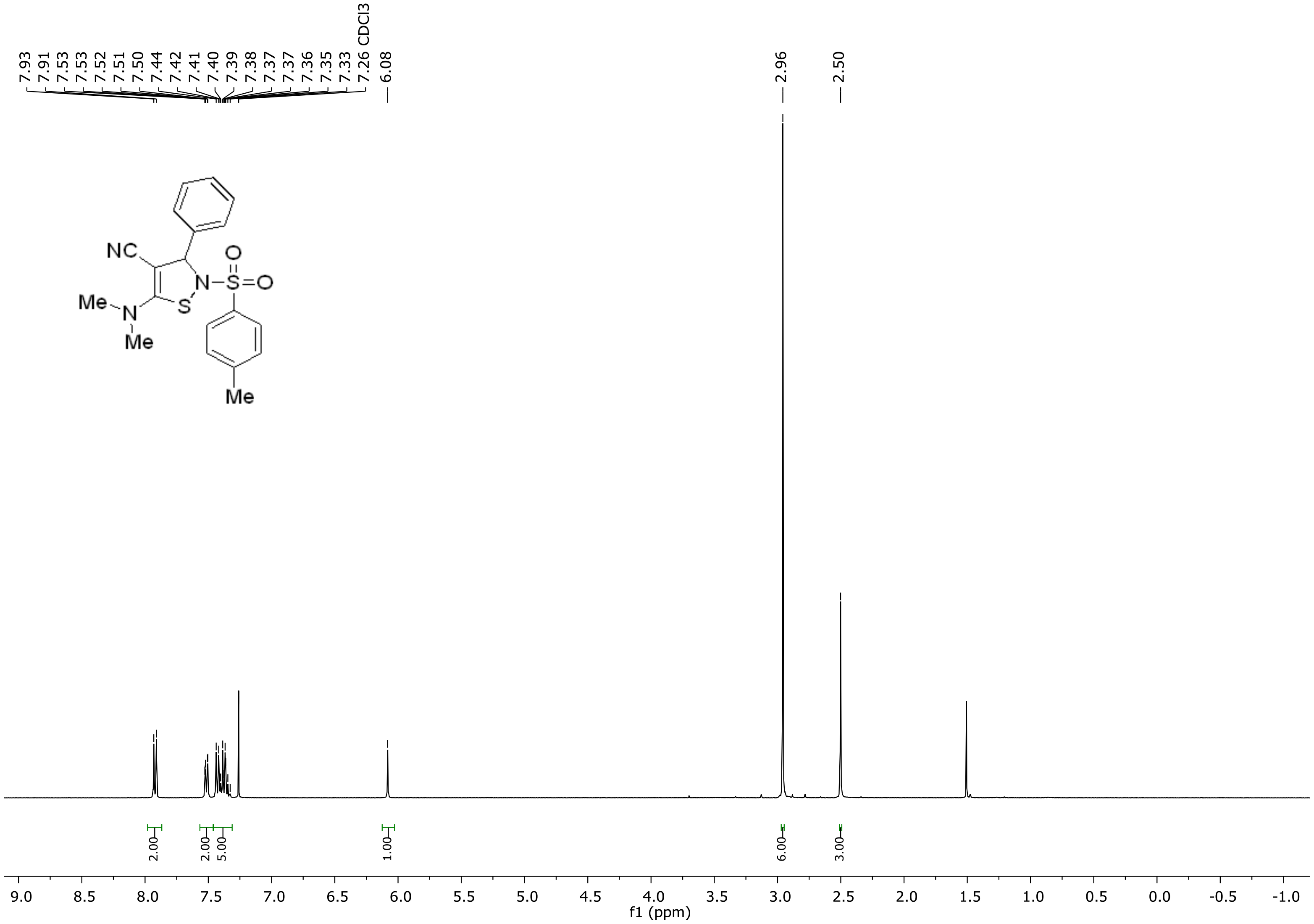

^{13}C NMR ($CDCl_3$) spectrum of **3ea**

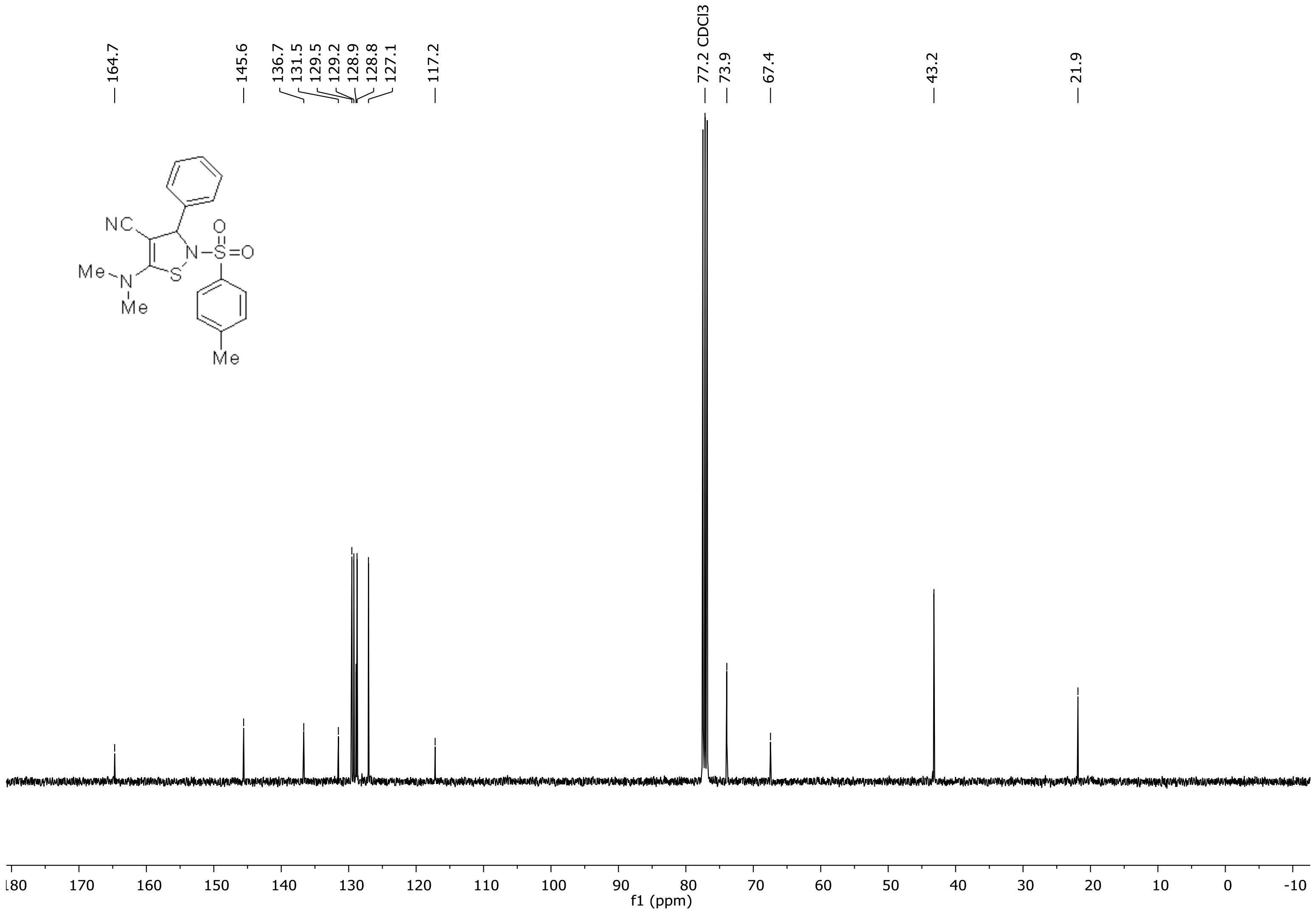

HRMS of **3ea**

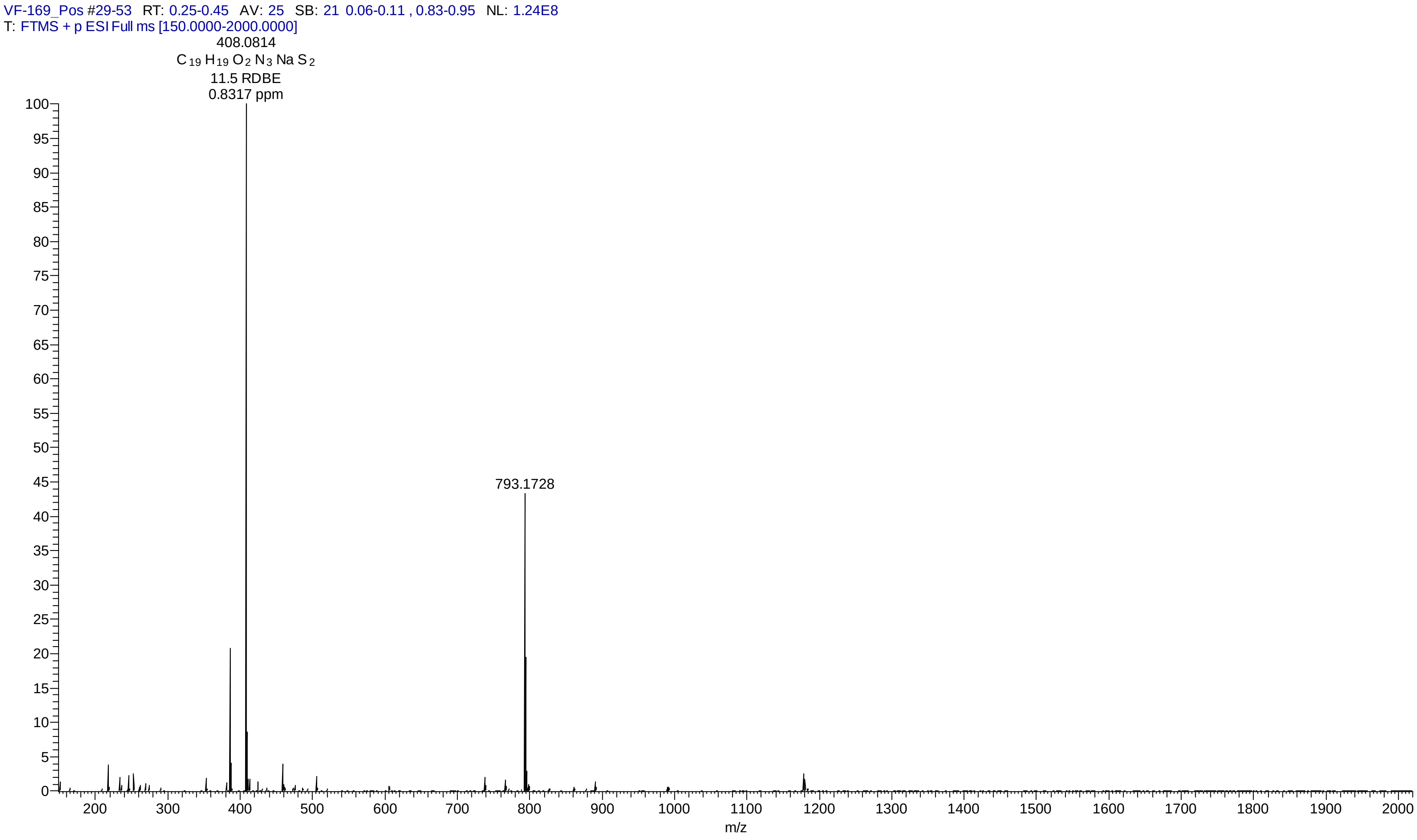

VF-169_Pos #29-53 RT: 0.25-0.45 AV: 25 SB: 21 0.06-0.11 , 0.83-0.95 NL: 1.24E8
T: FTMS + p ESI Full ms [150.0000-2000.0000]
408.0814
C 19 H 19 O 2 N 3 Na S 2
11.5 RDBE
0.8317 ppm
793.1728
m/z

^{1}H NMR ($CDCl_3$) spectrum of **3fa**

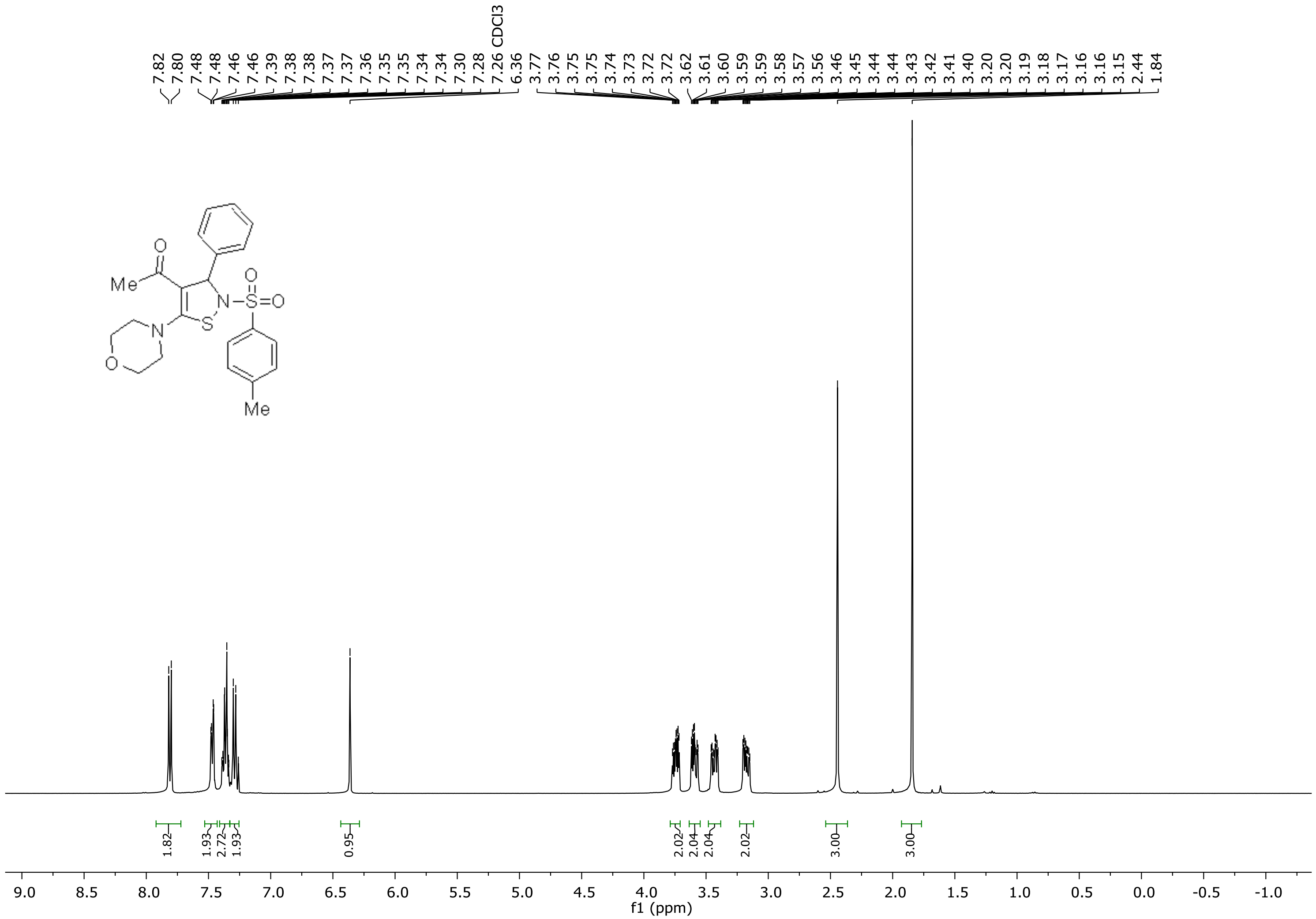

^{13}C NMR ($CDCl_3$) spectrum of **3fa**

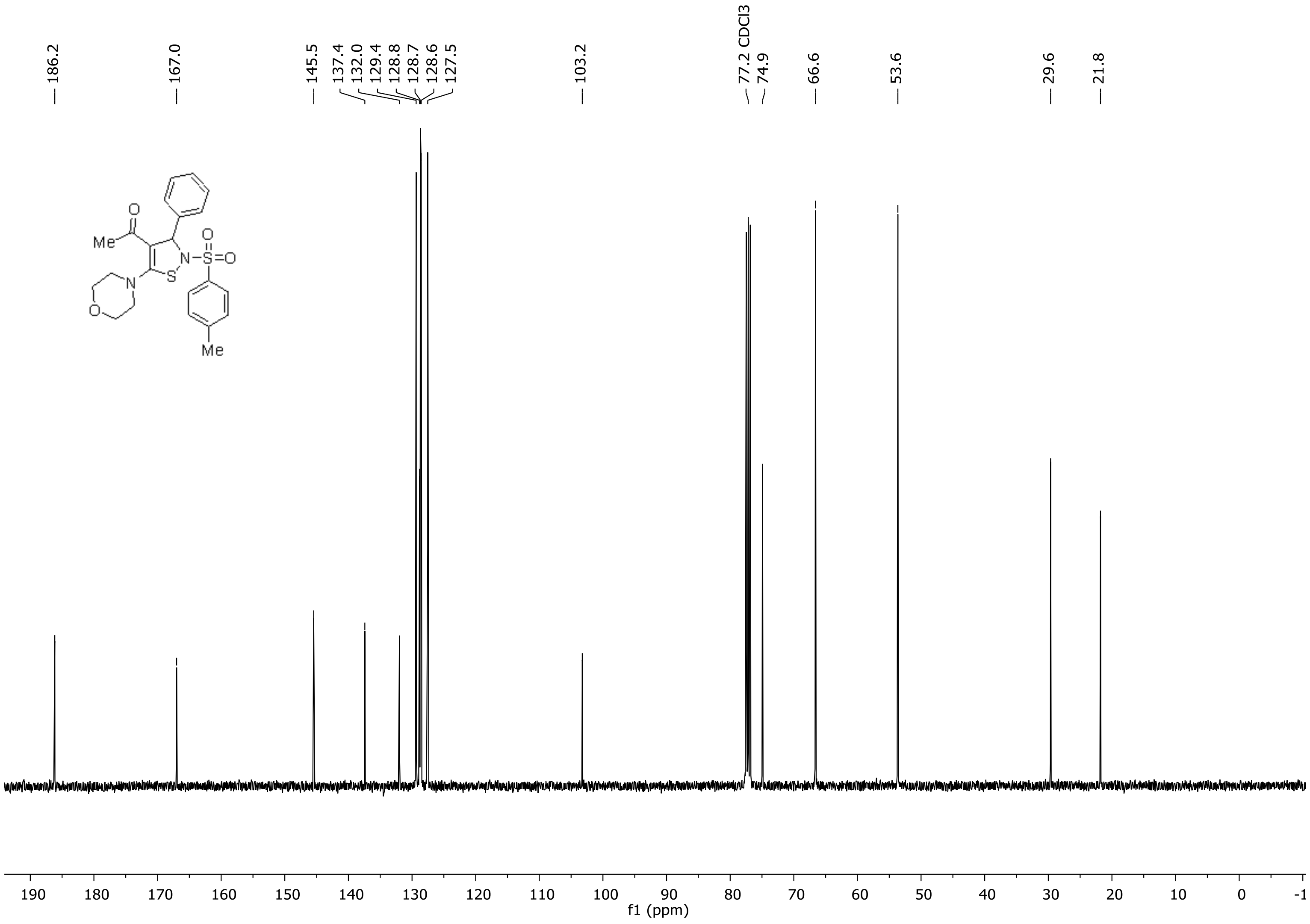

HRMS of **3fa**

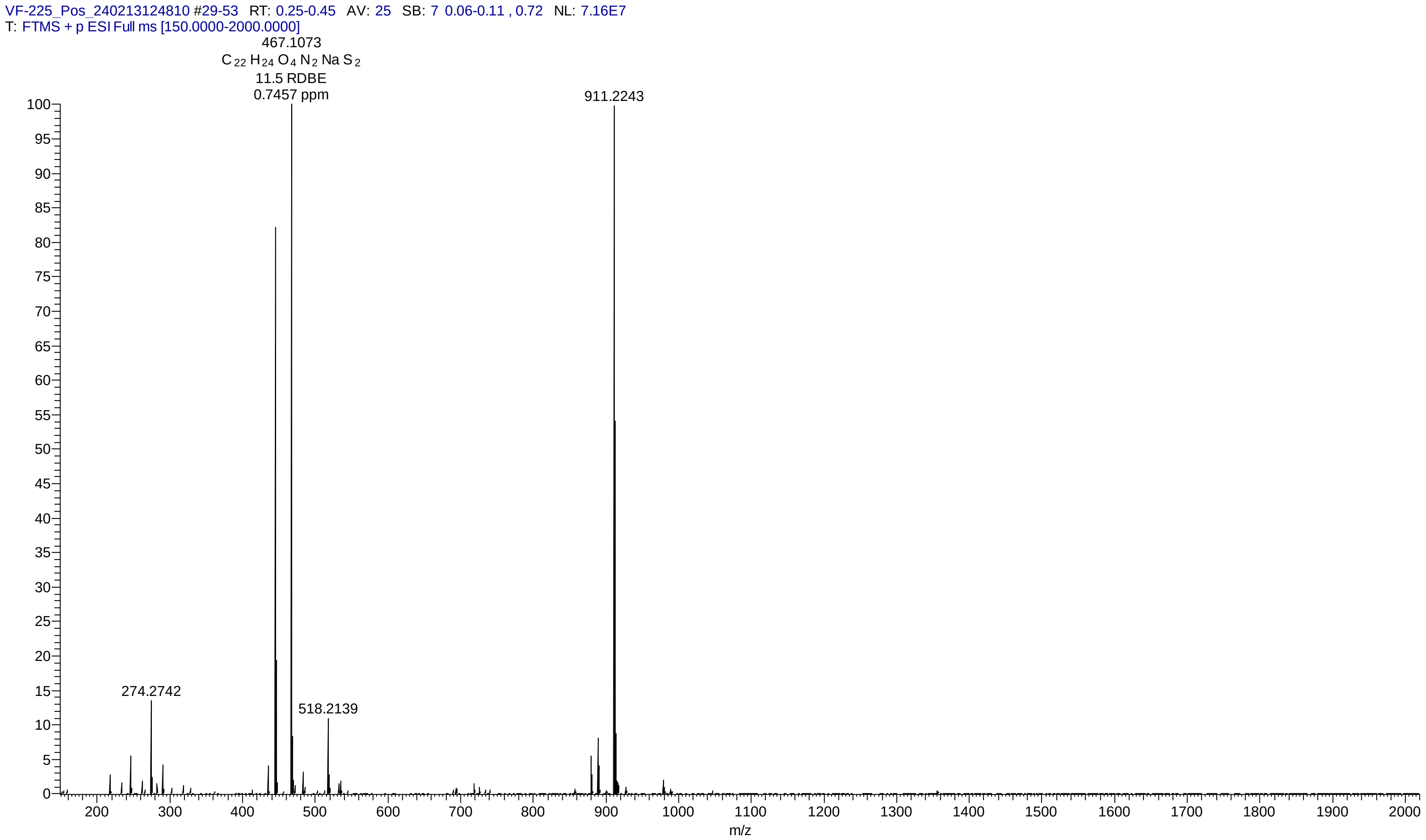

VF-225_Pos_240213124810 #29-53 RT: 0.25-0.45 AV: 25 SB: 7 0.06-0.11 , 0.72 NL: 7.16E7
T: FTMS + p ESI Full ms [150.0000-2000.0000]
467.1073
C 22 H 24 O 4 N 2 Na S 2
11.5 RDBE
0.7457 ppm
911.2243
274.2742
518.2139
m/z

^{1}H NMR (CDCl$_3$) spectrum of **3ga**

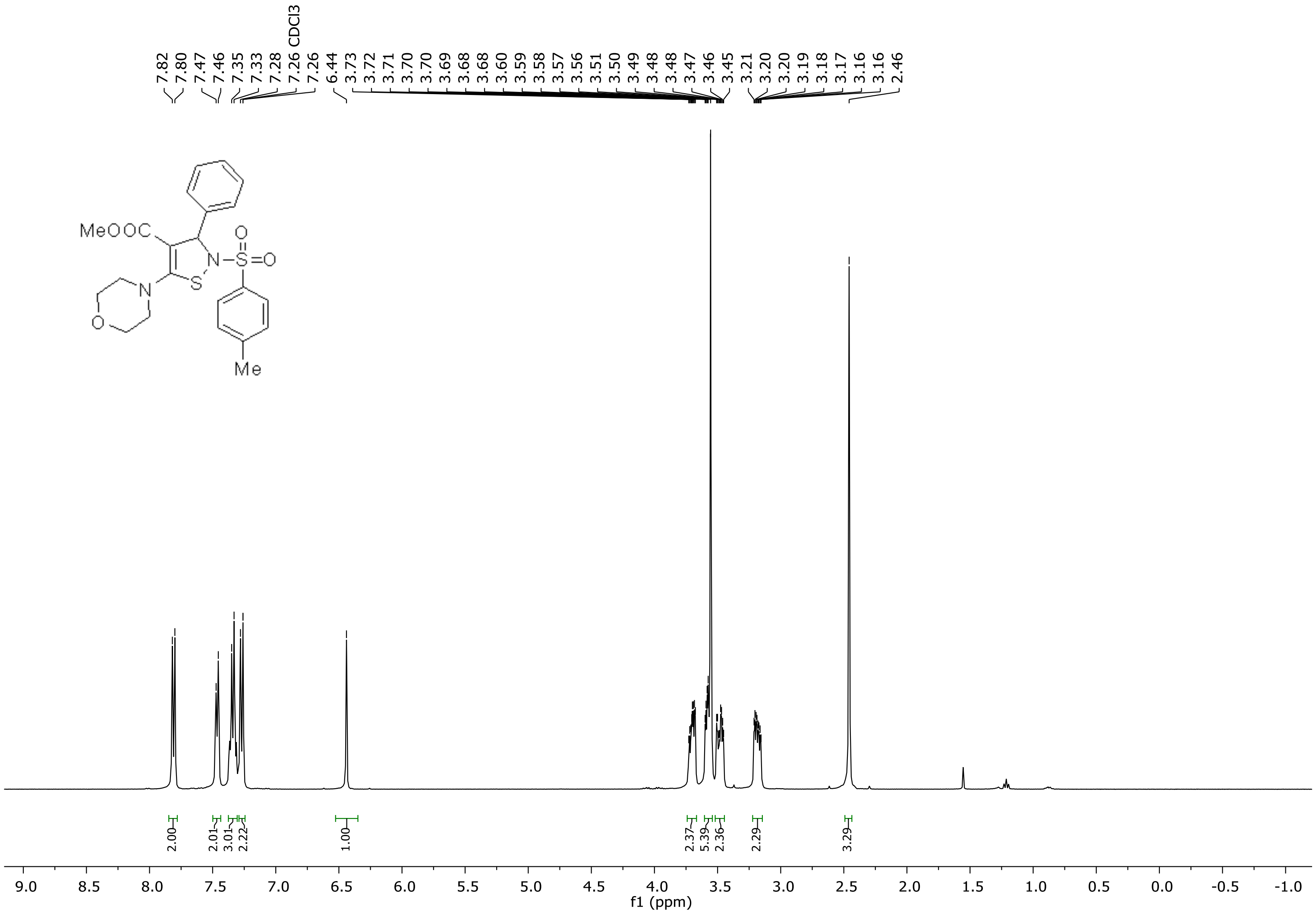

^{13}C NMR ($CDCl_3$) spectrum of **3ga**

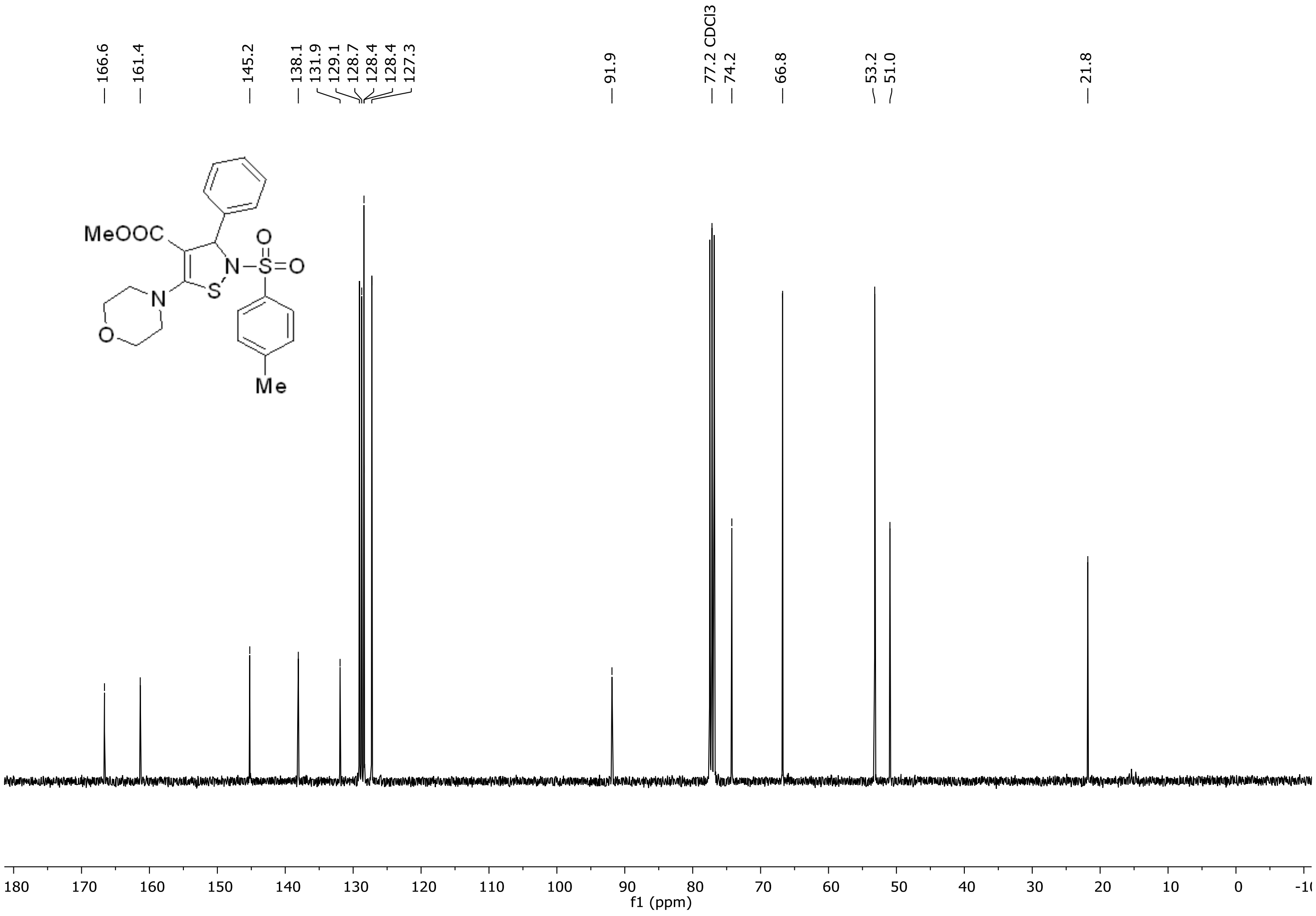

HRMS of **3ga**

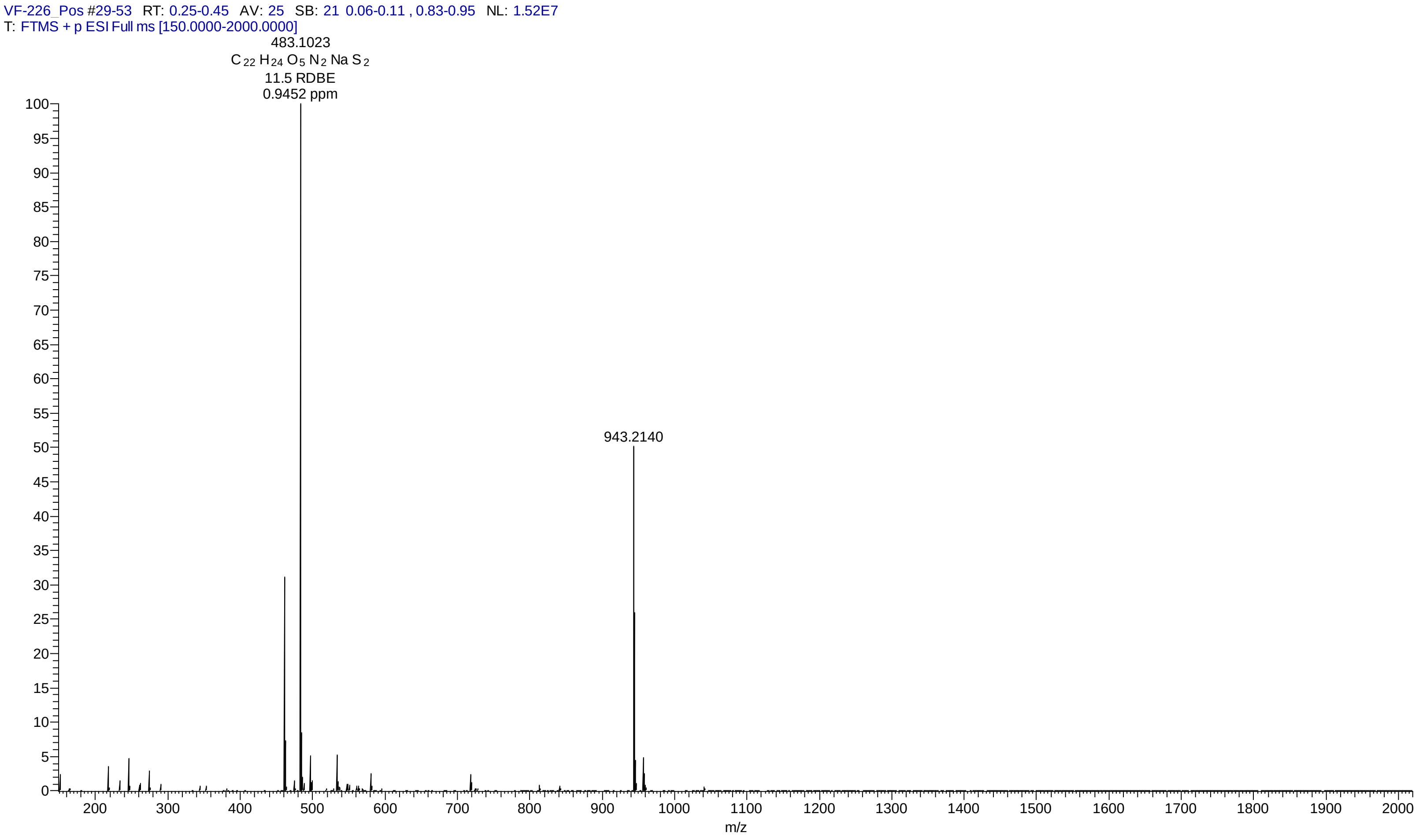

VF-226_Pos #29-53 RT: 0.25-0.45 AV: 25 SB: 21 0.06-0.11 , 0.83-0.95 NL: 1.52E7
T: FTMS + p ESI Full ms [150.0000-2000.0000]
483.1023
C 22 H 24 O 5 N 2 Na S 2
11.5 RDBE
0.9452 ppm
943.2140
m/z

^{1}H NMR ($CDCl_3$) spectrum of **3ha**

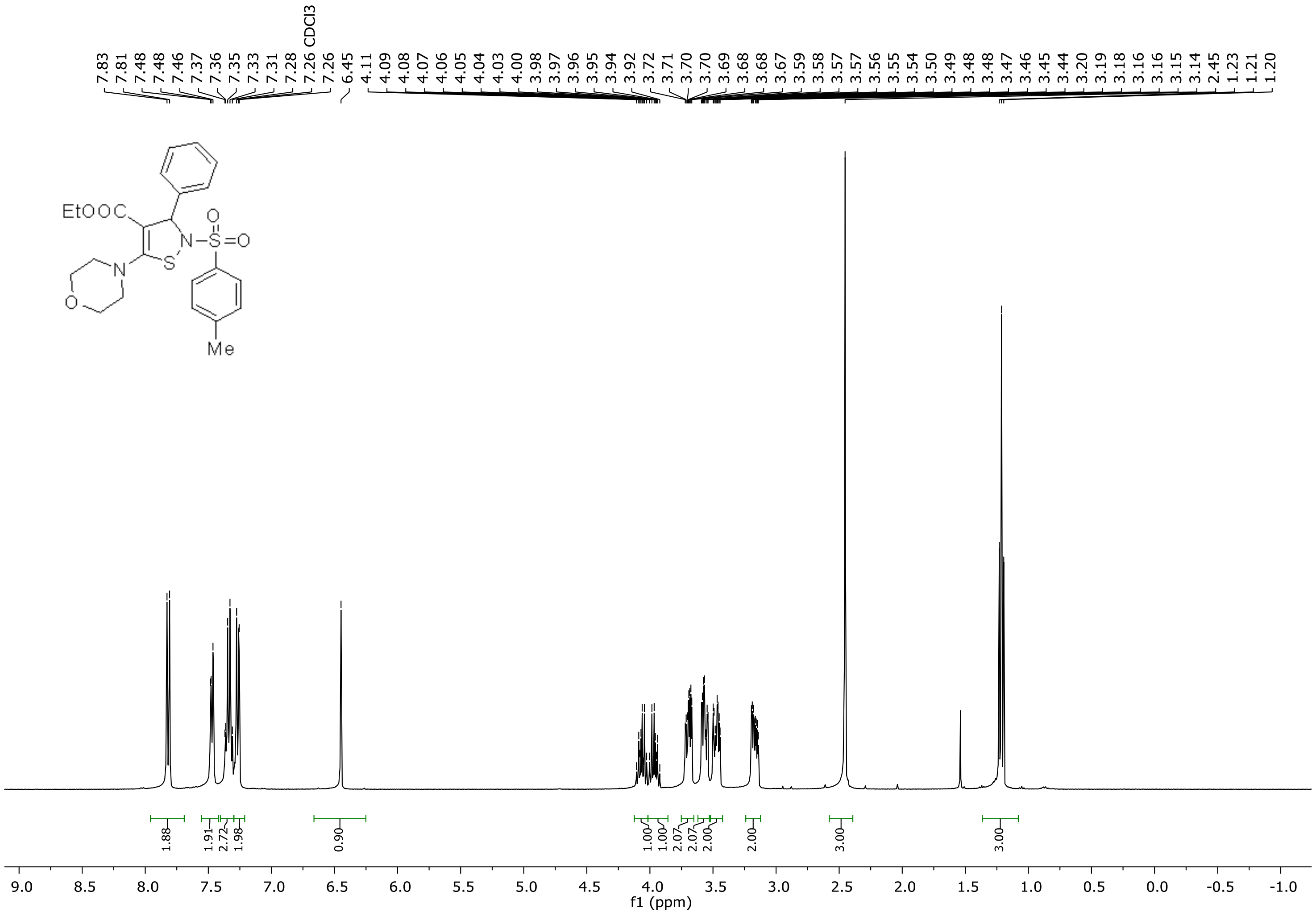

^{13}C NMR (CDCl$_3$) spectrum of **3ha**

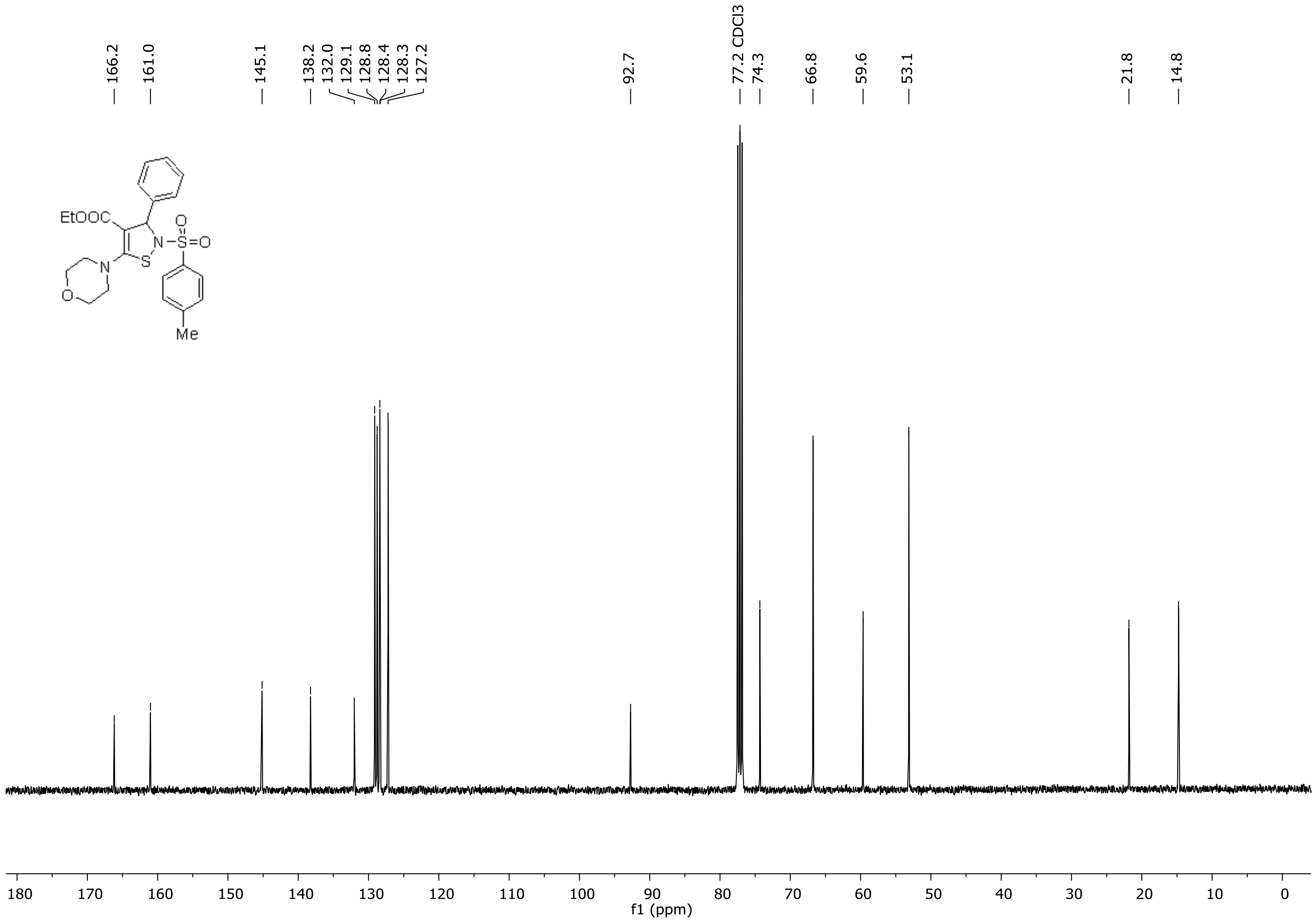

HRMS of **3ha**

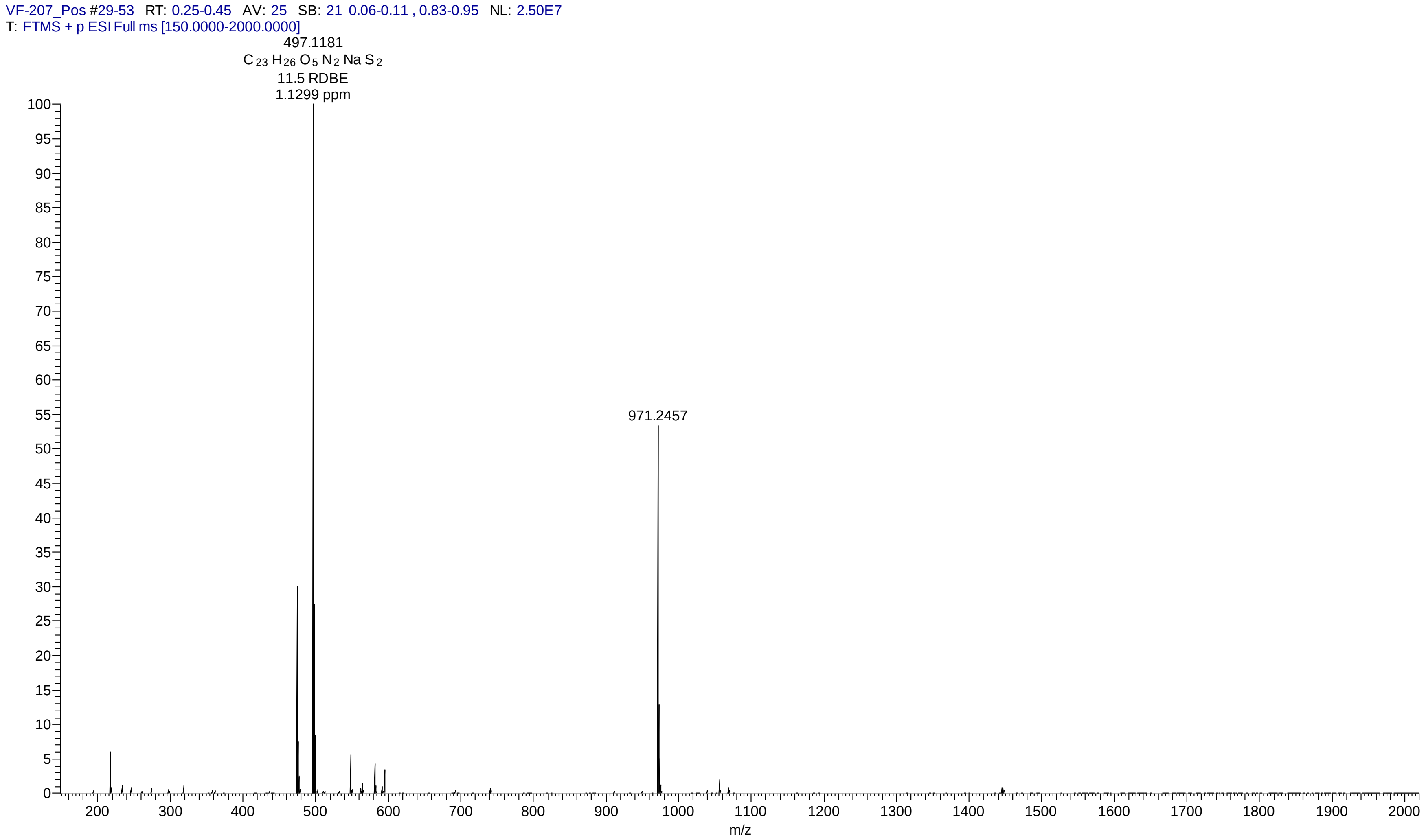

VF-207_Pos #29-53 RT: 0.25-0.45 AV: 25 SB: 21 0.06-0.11 , 0.83-0.95 NL: 2.50E7
T: FTMS + p ESI Full ms [150.0000-2000.0000]
497.1181
C 23 H 26 O 5 N 2 Na S 2
11.5 RDBE
1.1299 ppm
971.2457
m/z

^{1}H NMR ($CDCl_3$) spectrum of **3ia**

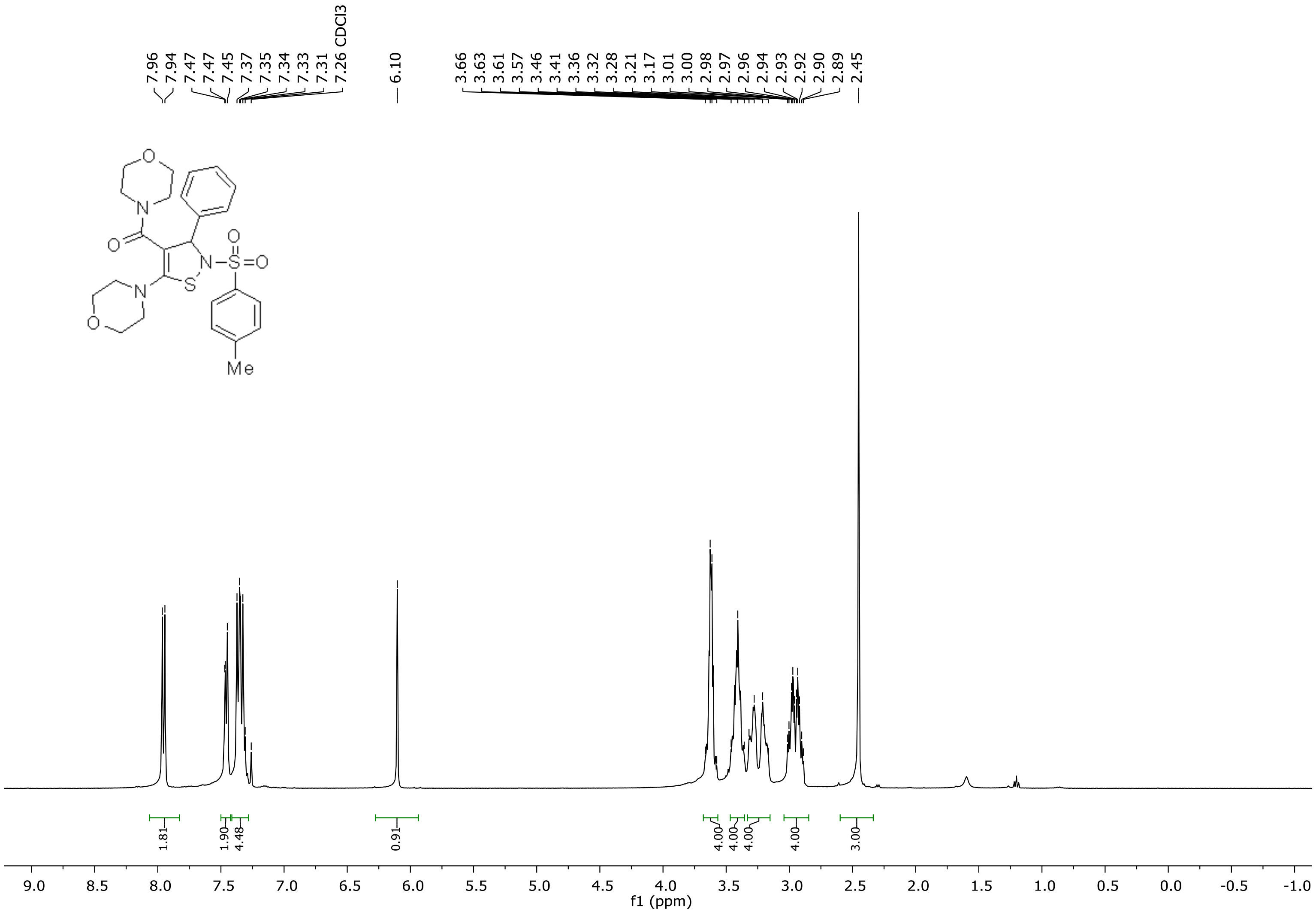

^{13}C NMR ($CDCl_3$) spectrum of **3ia**

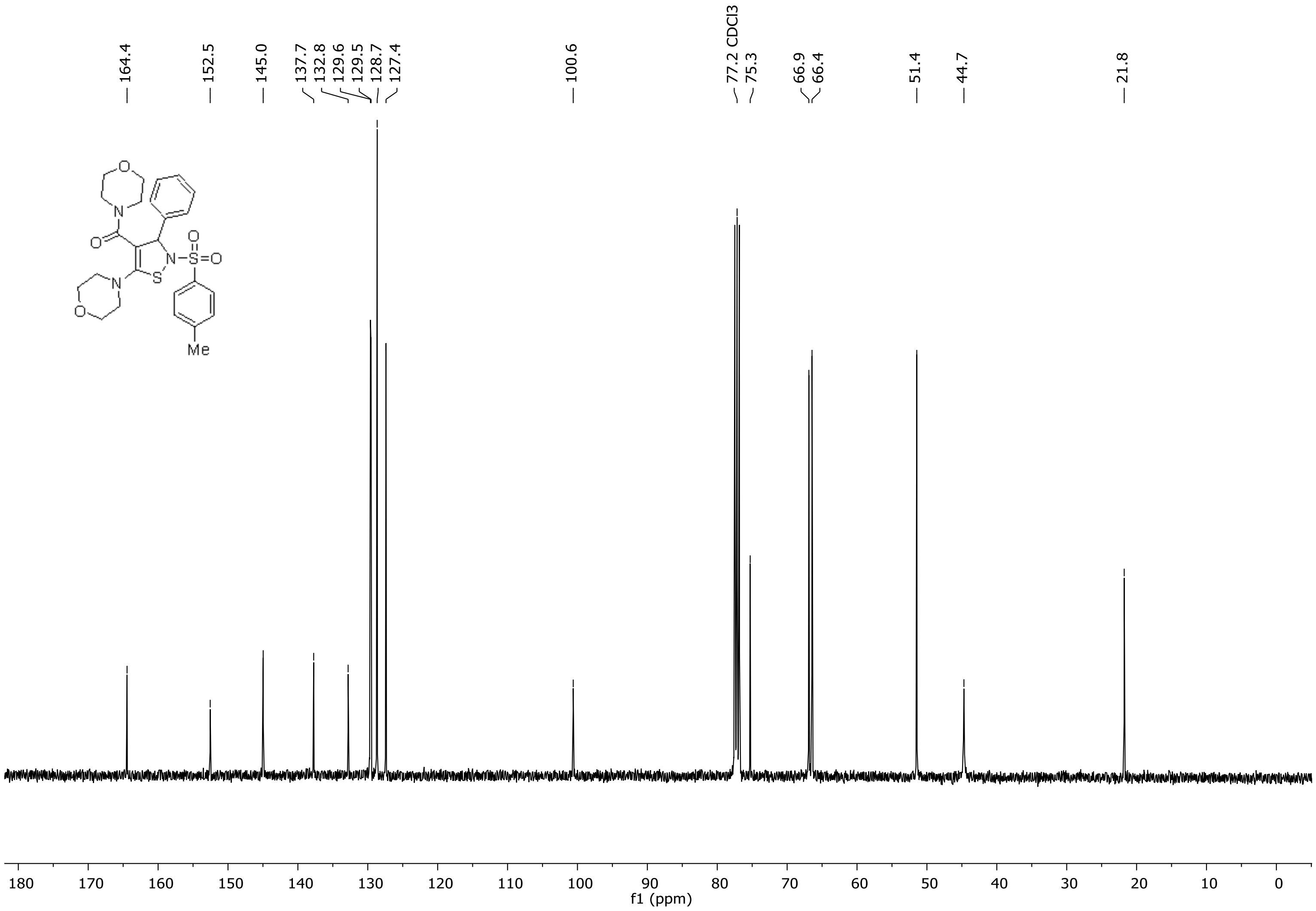

HRMS of **3ia**

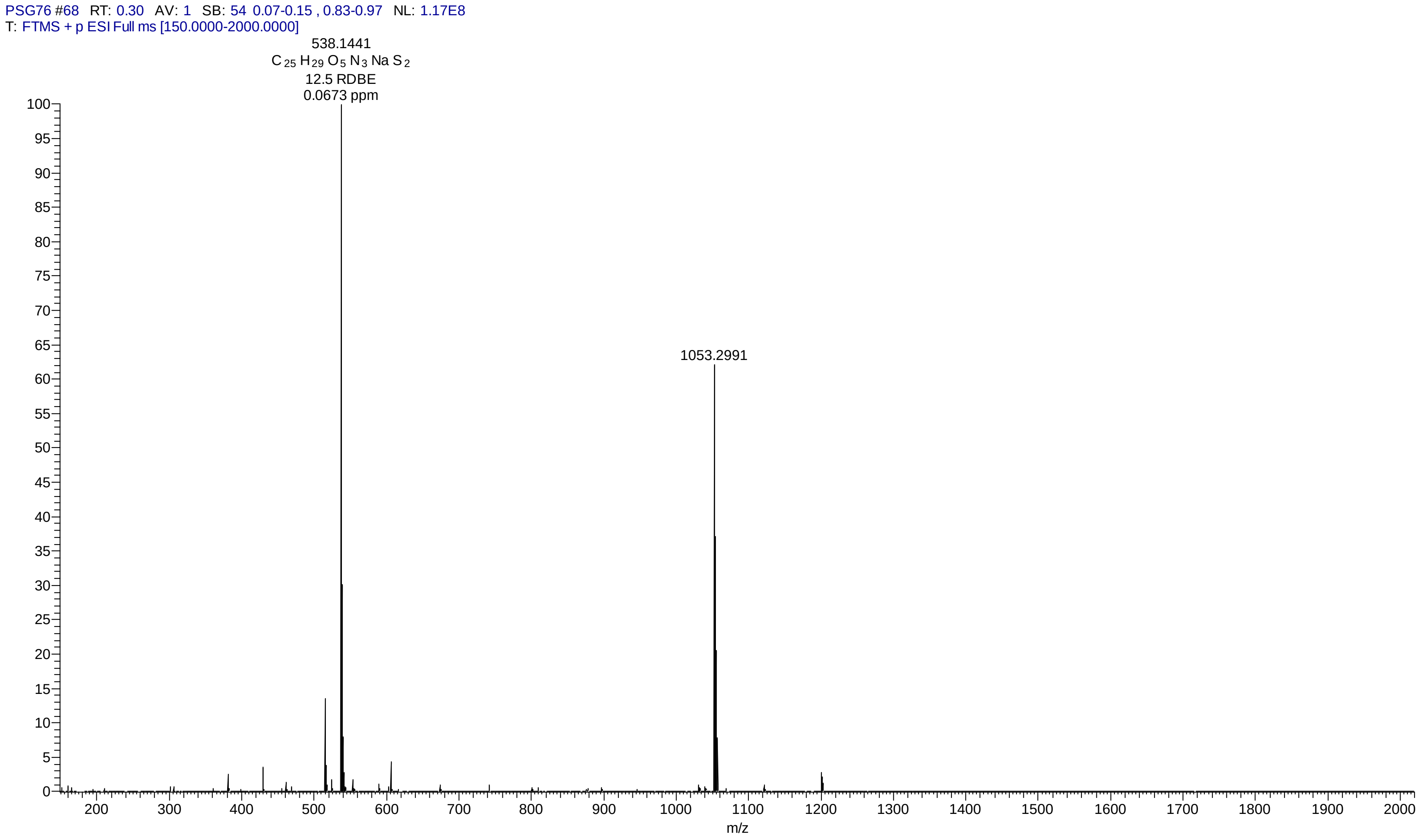

PSG76 #68 RT: 0.30 AV: 1 SB: 54 0.07-0.15 , 0.83-0.97 NL: 1.17E8
T: FTMS + p ESI Full ms [150.0000-2000.0000]
538.1441
C 25 H 29 O 5 N 3 Na S 2
12.5 RDBE
0.0673 ppm
1053.2991
m/z

^1H NMR ($CDCl_3$) spectrum of **3ja**

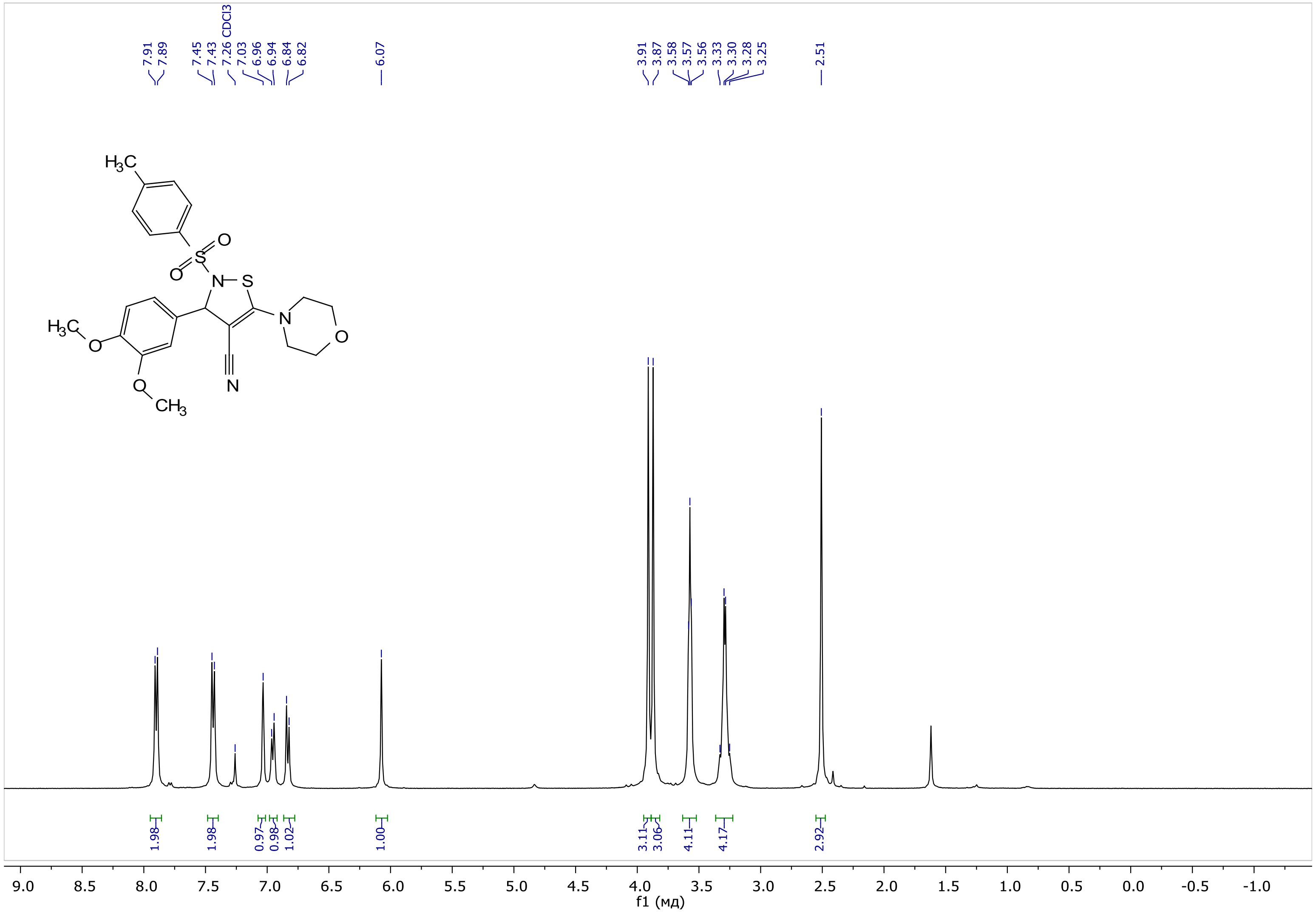

^{13}C NMR ($CDCl_3$) spectrum of **3ja**

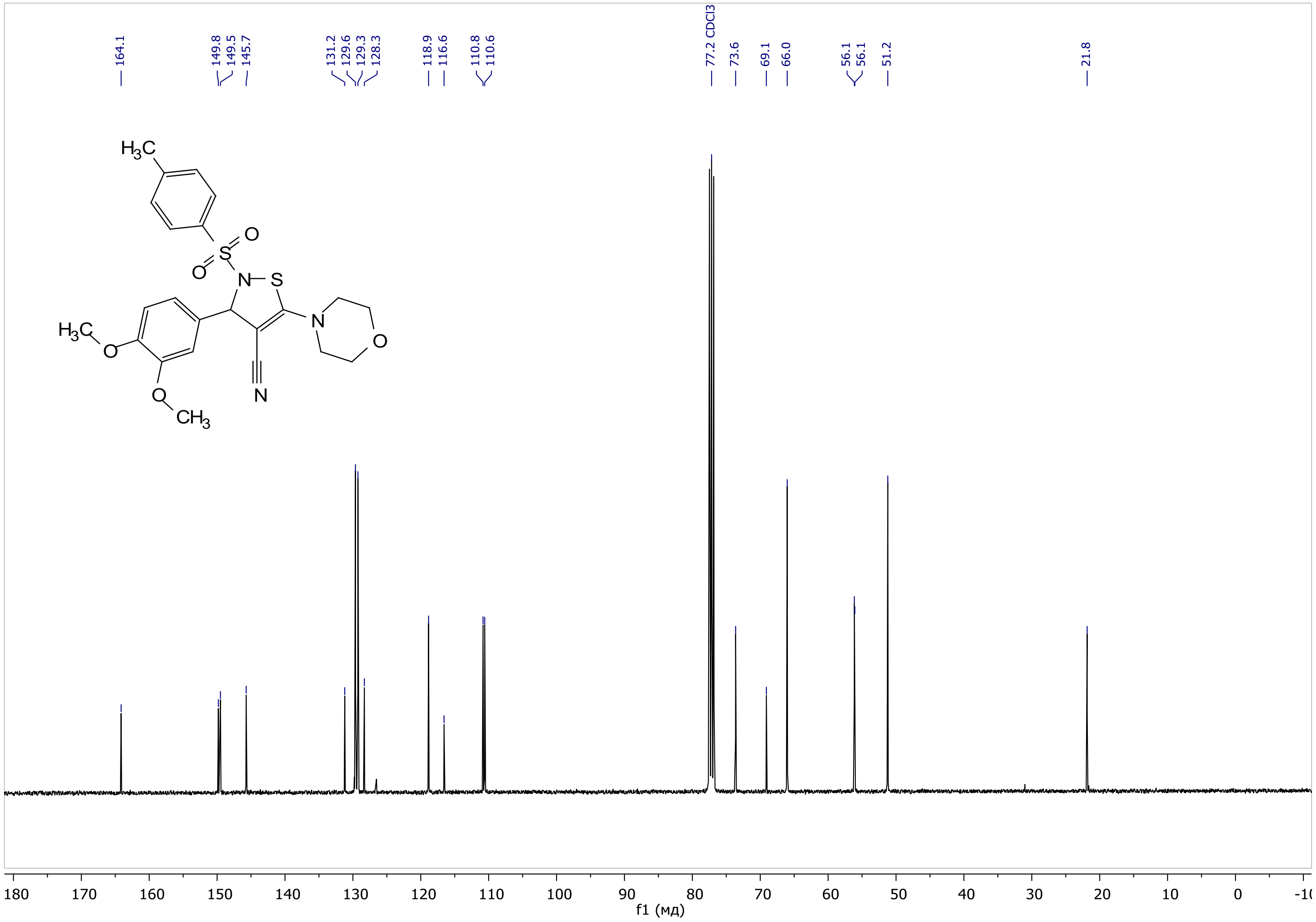

HRMS of **3ja**

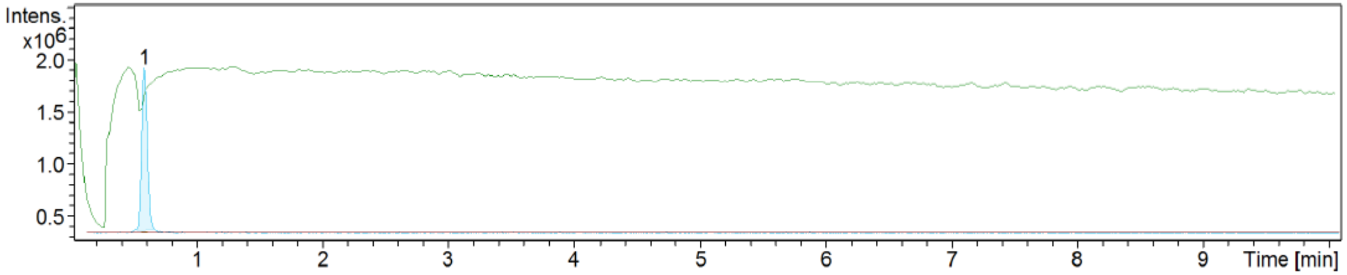

#	RT [min]	Area	Int. Type	I	S/N	Chromatogram	Max. m/z	FWHM [min]
1	0.6	257715	Manual	80426	193.0	UV Chromatogram, 190-800 nm	488.1312	

Cmpd 1, 0.6 min

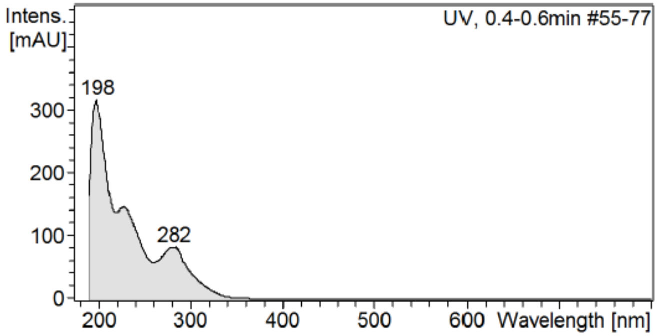

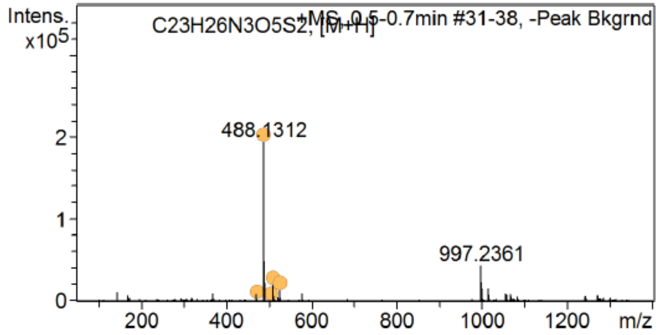

#	Wavelength	Intensity
0	198	314.3
1	282	82.4

#	m/z	Res.	S/N	I	I %	FWHM
1	143.1180	16781	3880511.5	11032	5.7	0.0085
2	488.1312	25807	68036504.0	193421	100.0	0.0189
3	489.1337	21515	17491142.0	49726	25.7	0.0227
4	490.1294	19151	7984089.0	22698	11.7	0.0256
5	510.1122	20146	6707280.5	19068	9.9	0.0253
6	526.0862	20197	4741428.5	13479	7.0	0.0260
7	997.2361	24204	15259904.0	43382	22.4	0.0412
8	998.2388	22076	8146493.5	23160	12.0	0.0452
9	999.2365	22085	5331550.5	15157	7.8	0.0452
10	1013.2098	22014	5355012.5	15224	7.9	0.0460

^{1}H NMR (DMSO-d_6) spectrum of **3ka**

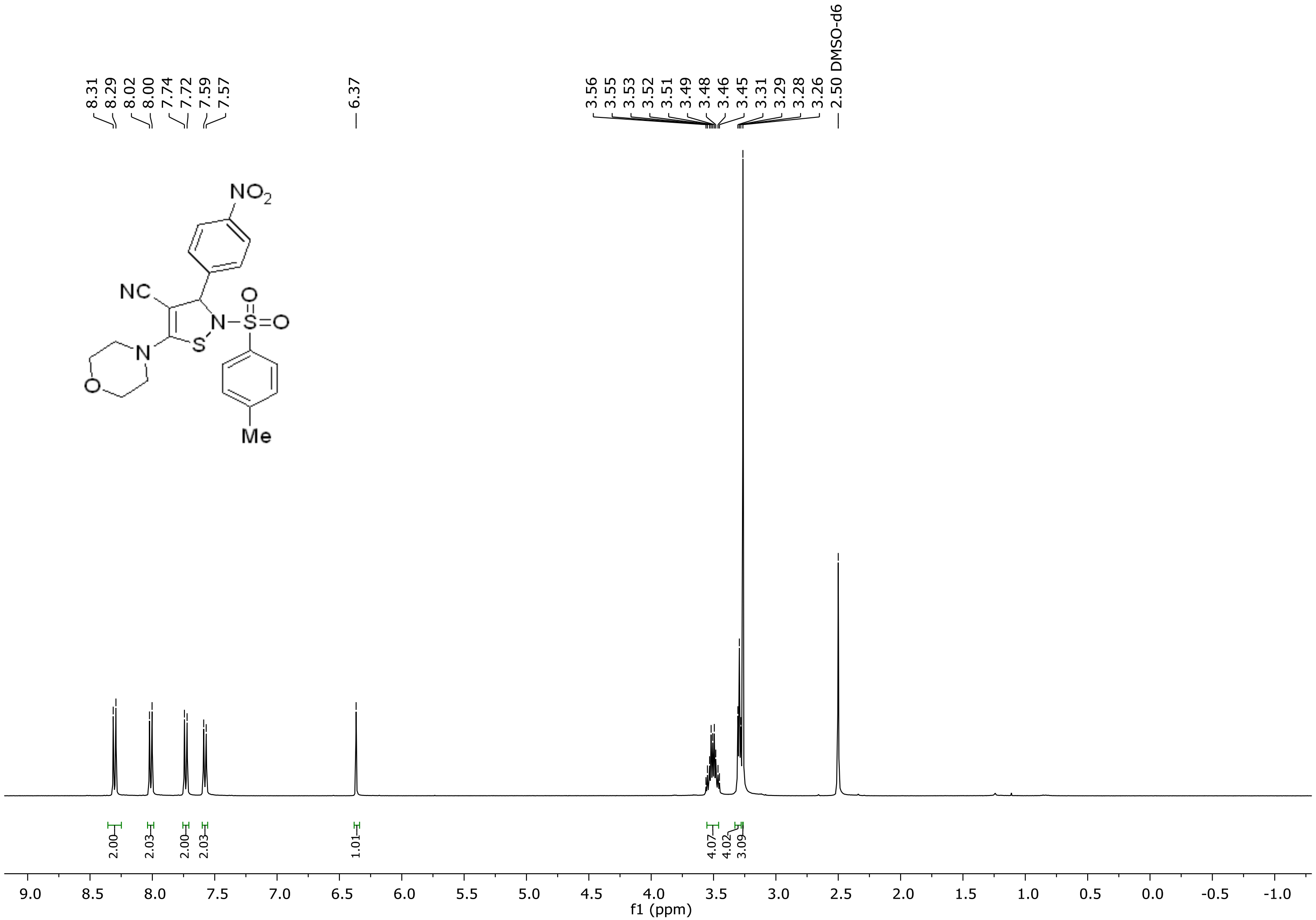

^{13}C NMR ($CDCl_3$) spectrum of **3ka**

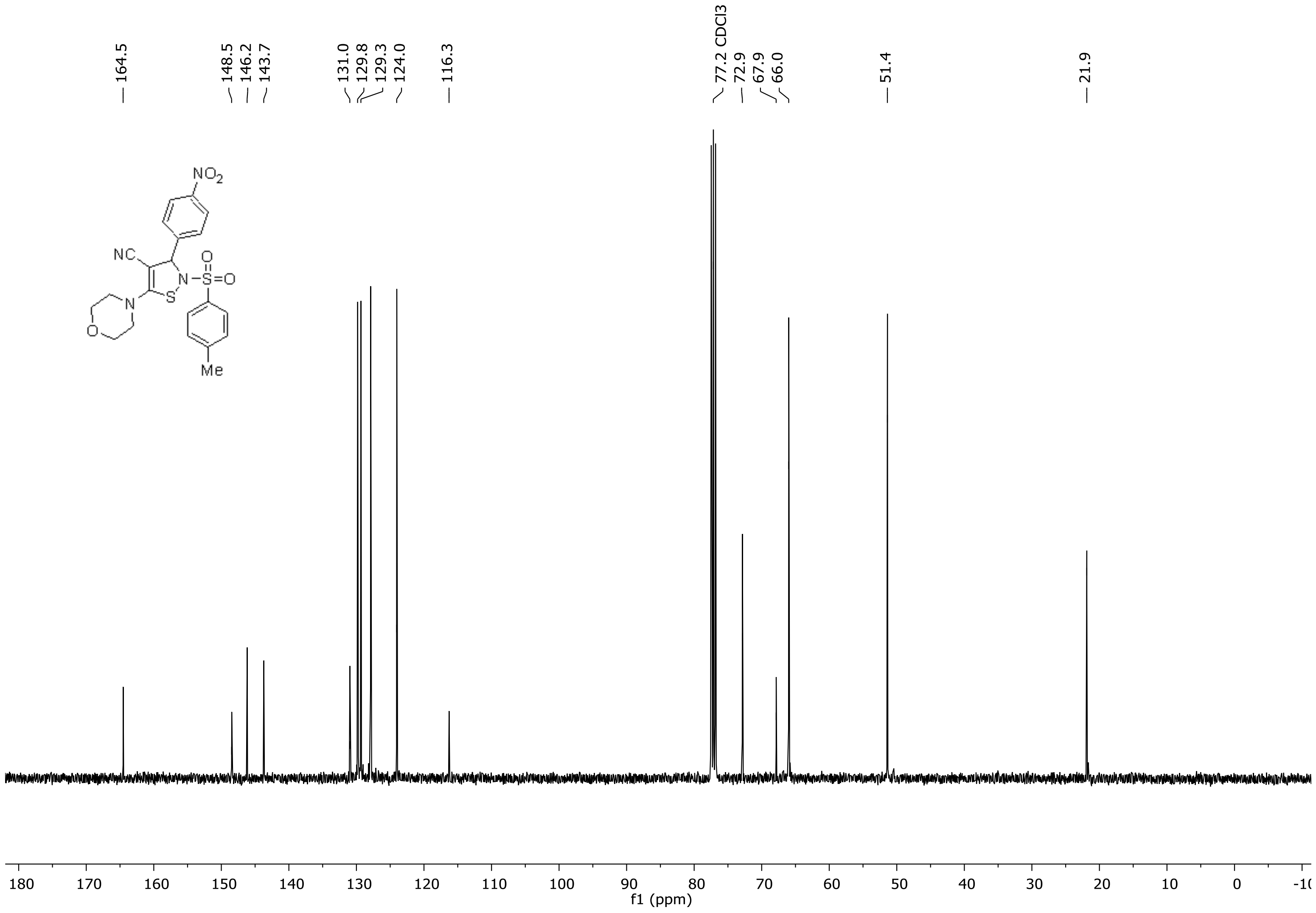

HRMS of **3ka**

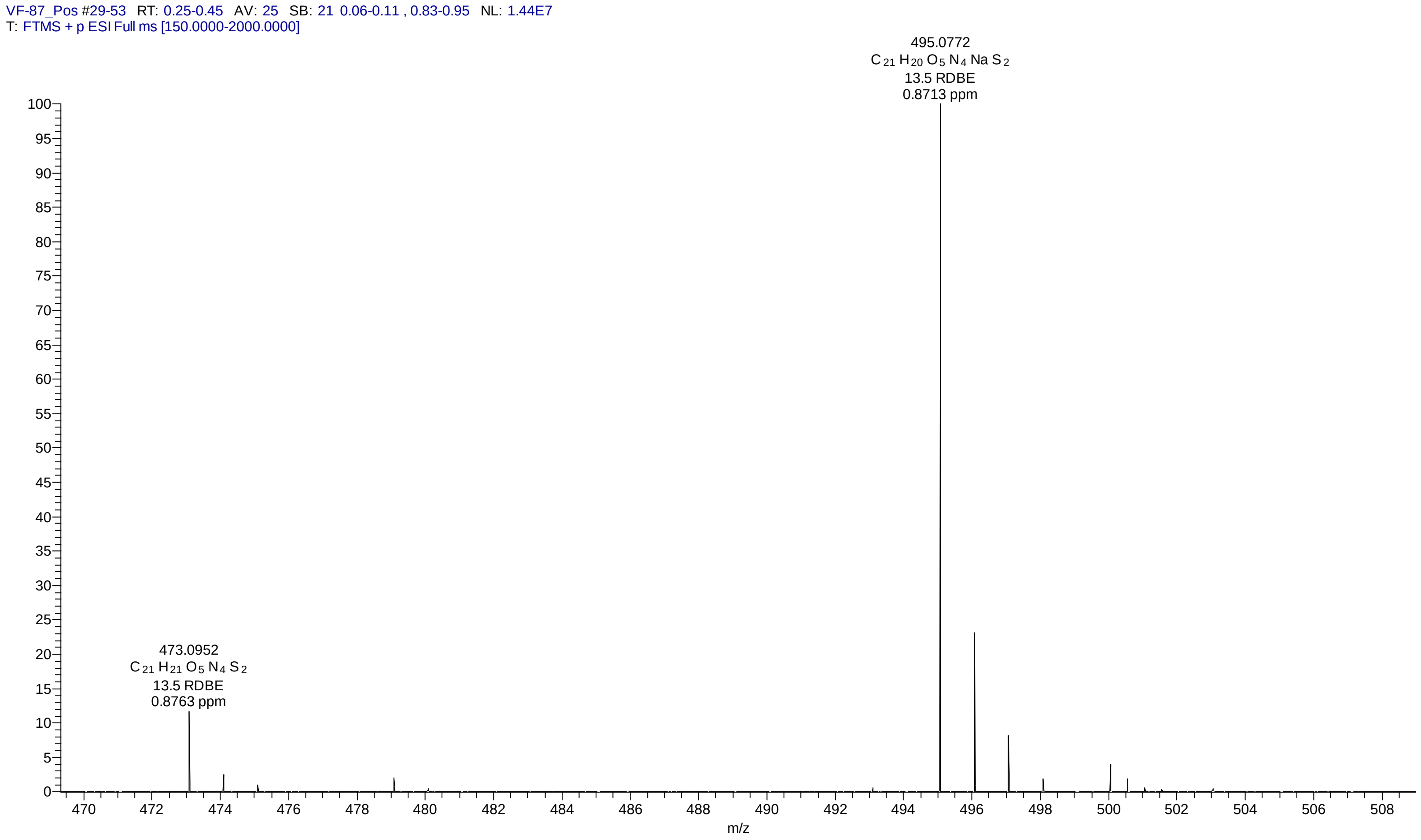

VF-87_Pos #29-53 RT: 0.25-0.45 AV: 25 SB: 21 0.06-0.11 , 0.83-0.95 NL: 1.44E7
T: FTMS + p ESI Full ms [150.0000-2000.0000]
495.0772
C 21 H 20 O 5 N 4 Na S 2
13.5 RDBE
0.8713 ppm
473.0952
C 21 H 21 O 5 N 4 S 2
13.5 RDBE
0.8763 ppm
m/z

^{1}H NMR ($CDCl_3$) spectrum of **3la**

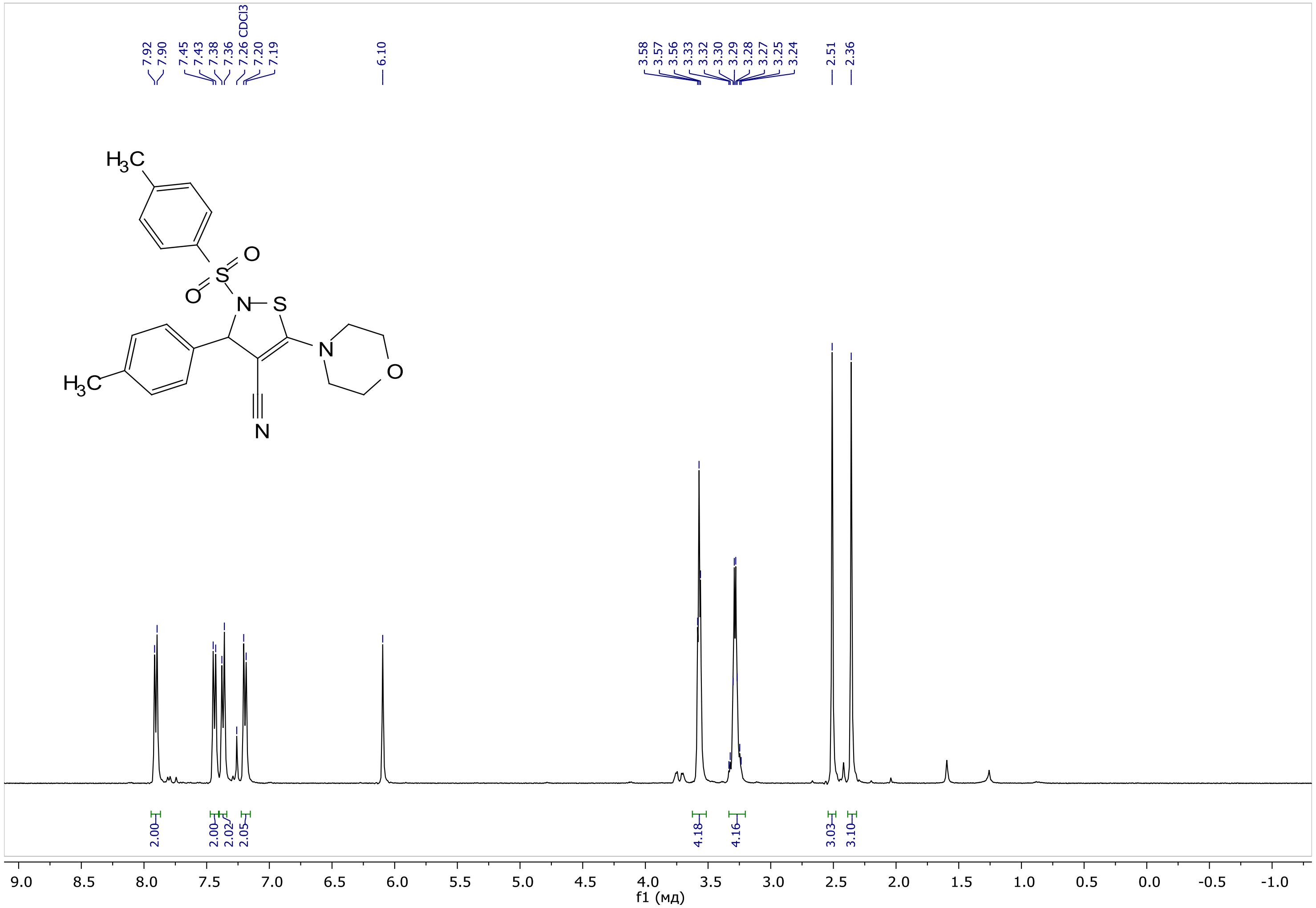

^{13}C NMR ($CDCl_3$) spectrum of **3la**

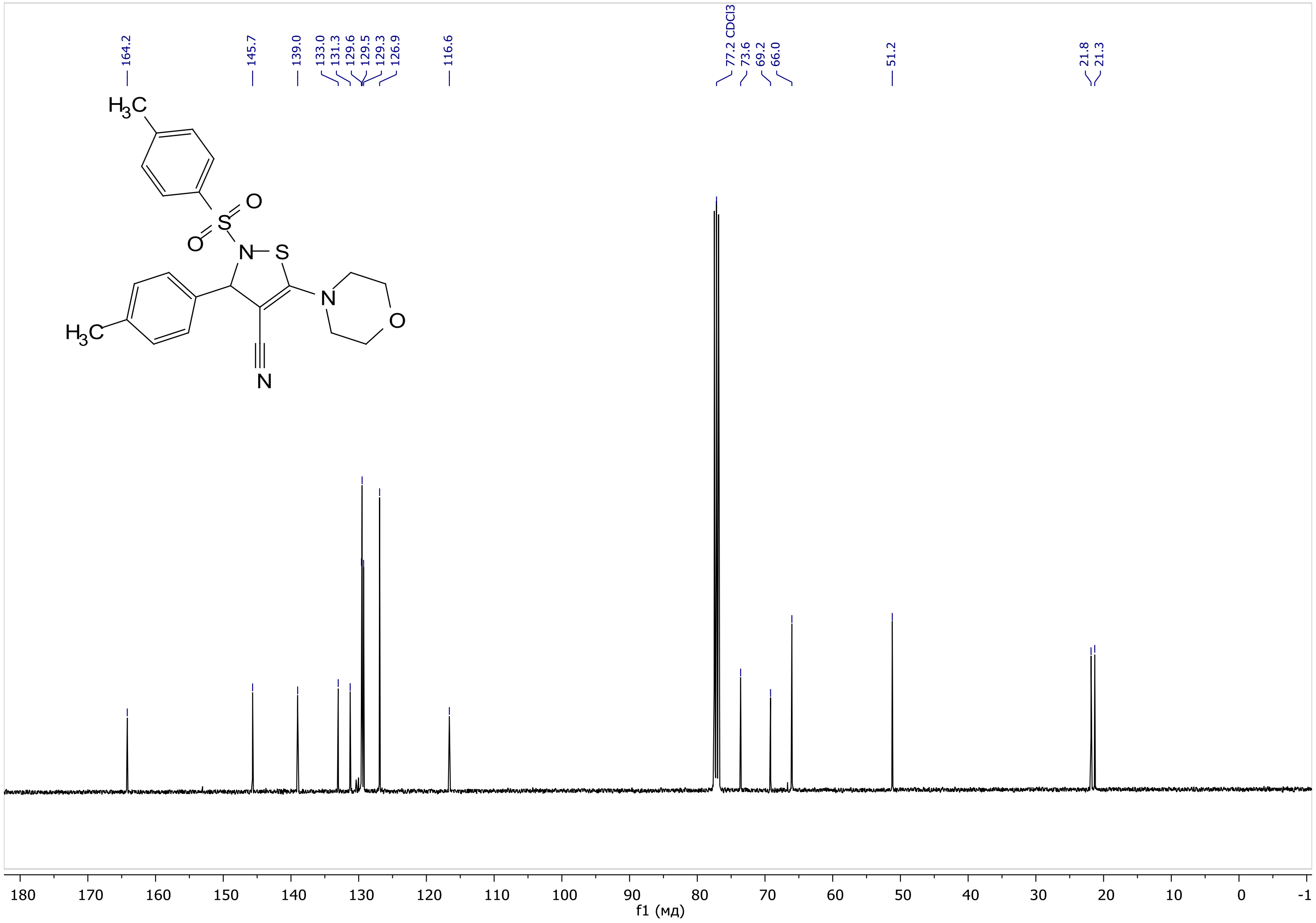

HRMS of **3la**

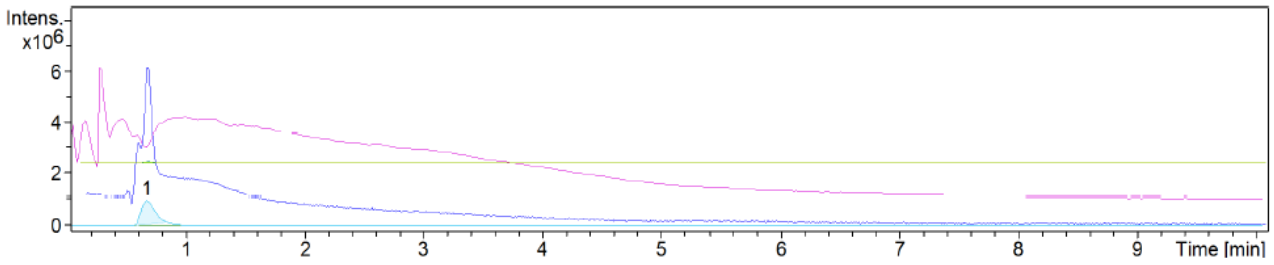

#	RT [min]	Area	Int. Type	I	S/N	Chromatogram	Max. m/z	FWHM [min]
1	0.7	6967170	Manual	950527	1870.0	EIC C21H20ClN3O3S2 [M+H, M+Na]+ (462.0707; 484.0527)±0.02 All MS	462.0712	

Cmpd 1, 0.7 min

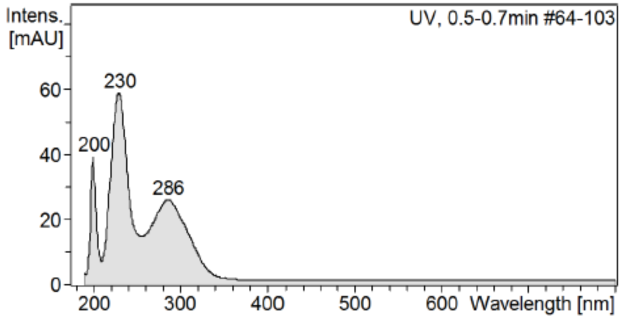

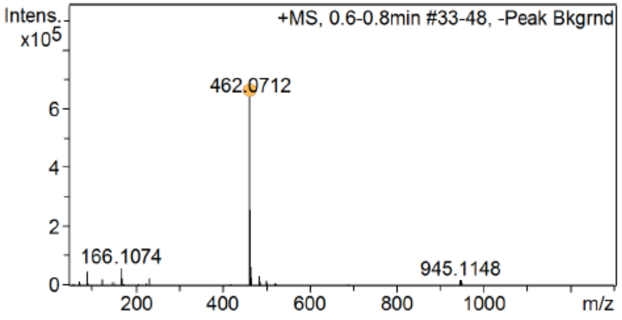

#	Wavelength	Intensity
0	200	39.3
1	230	58.8
2	286	26.2

#	m/z	Res.	S/N	I	I %	FWHM
1	91.0393	18339	23848024.0	44213	6.9	0.0050
2	166.1074	20910	30936734.0	57355	9.0	0.0079
3	169.0427	17541	12486836.0	23150	3.6	0.0096
4	231.0835	17801	12487251.0	23151	3.6	0.0130
5	462.0712	28467	344354048.0	638413	100.0	0.0162
6	463.0736	21407	80385752.0	149031	23.3	0.0216
7	464.0683	22912	138767184.0	257266	40.3	0.0203
8	465.0703	19740	32930016.0	61050	9.6	0.0236
9	466.0663	17751	12733924.0	23608	3.7	0.0263
10	484.0521	19481	16335288.0	30285	4.7	0.0248

^{1}H NMR ($CDCl_3$) spectrum of **3ma**

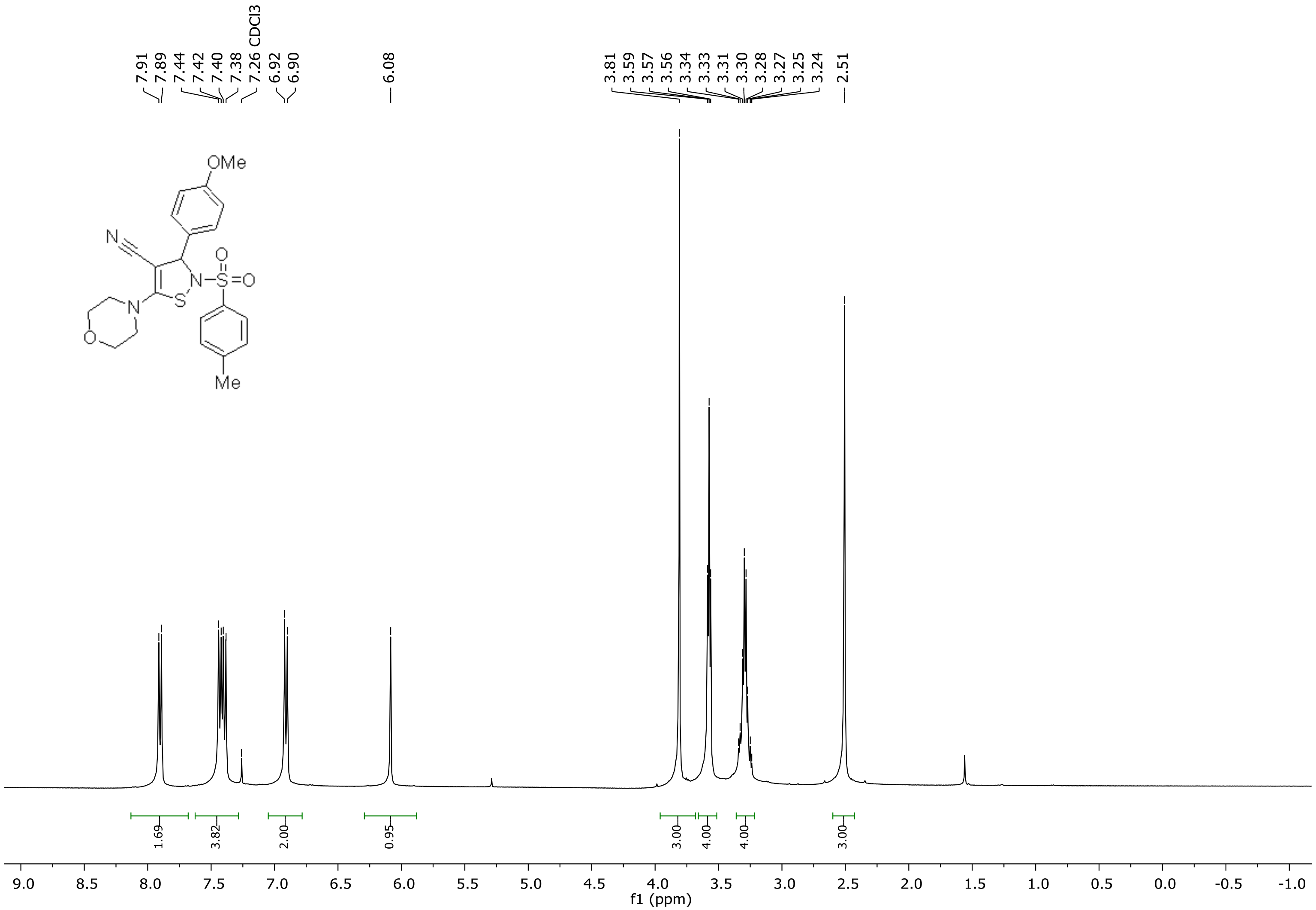

^{13}C NMR ($CDCl_3$) spectrum of **3ma**

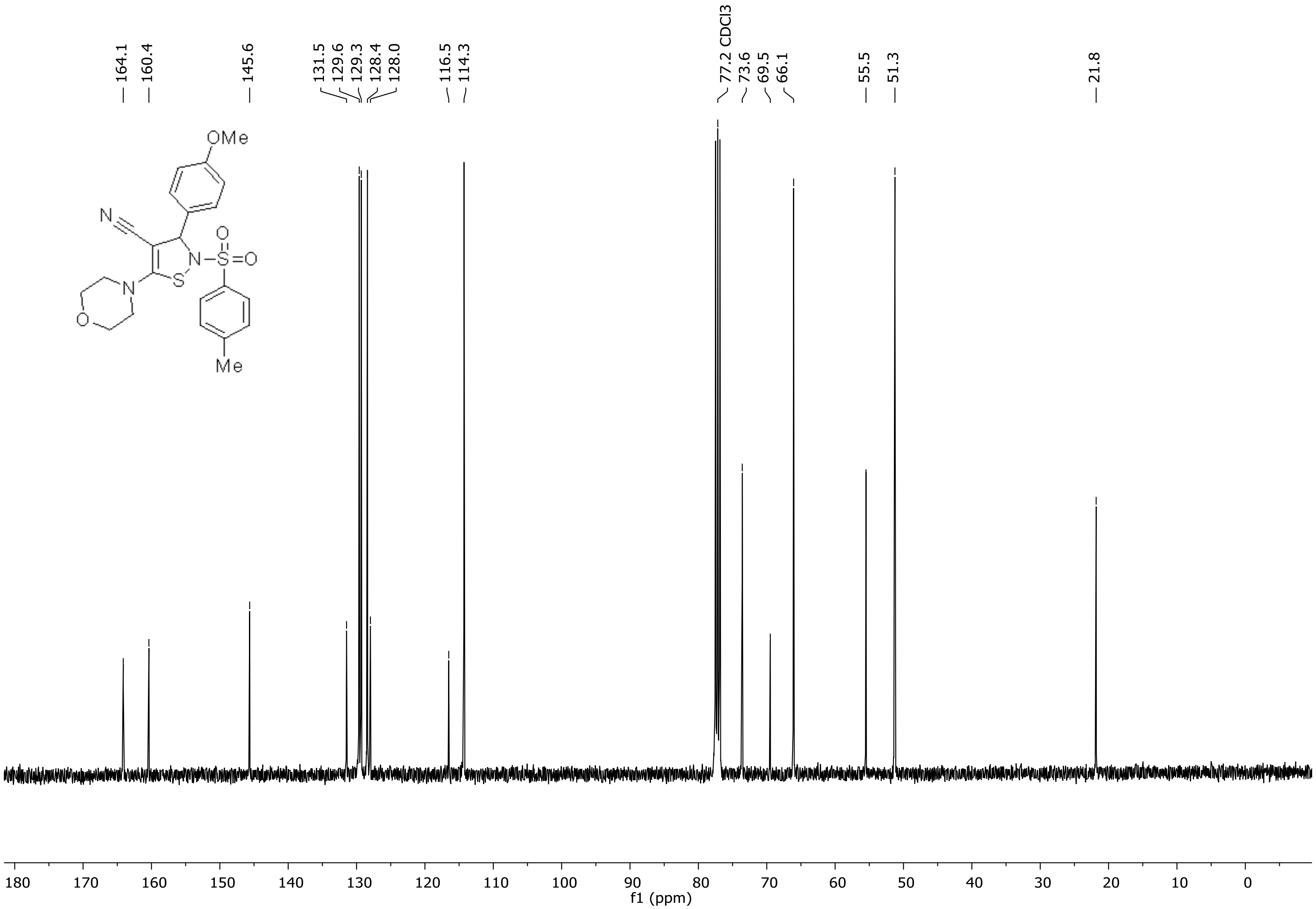

HRMS of **3ma**

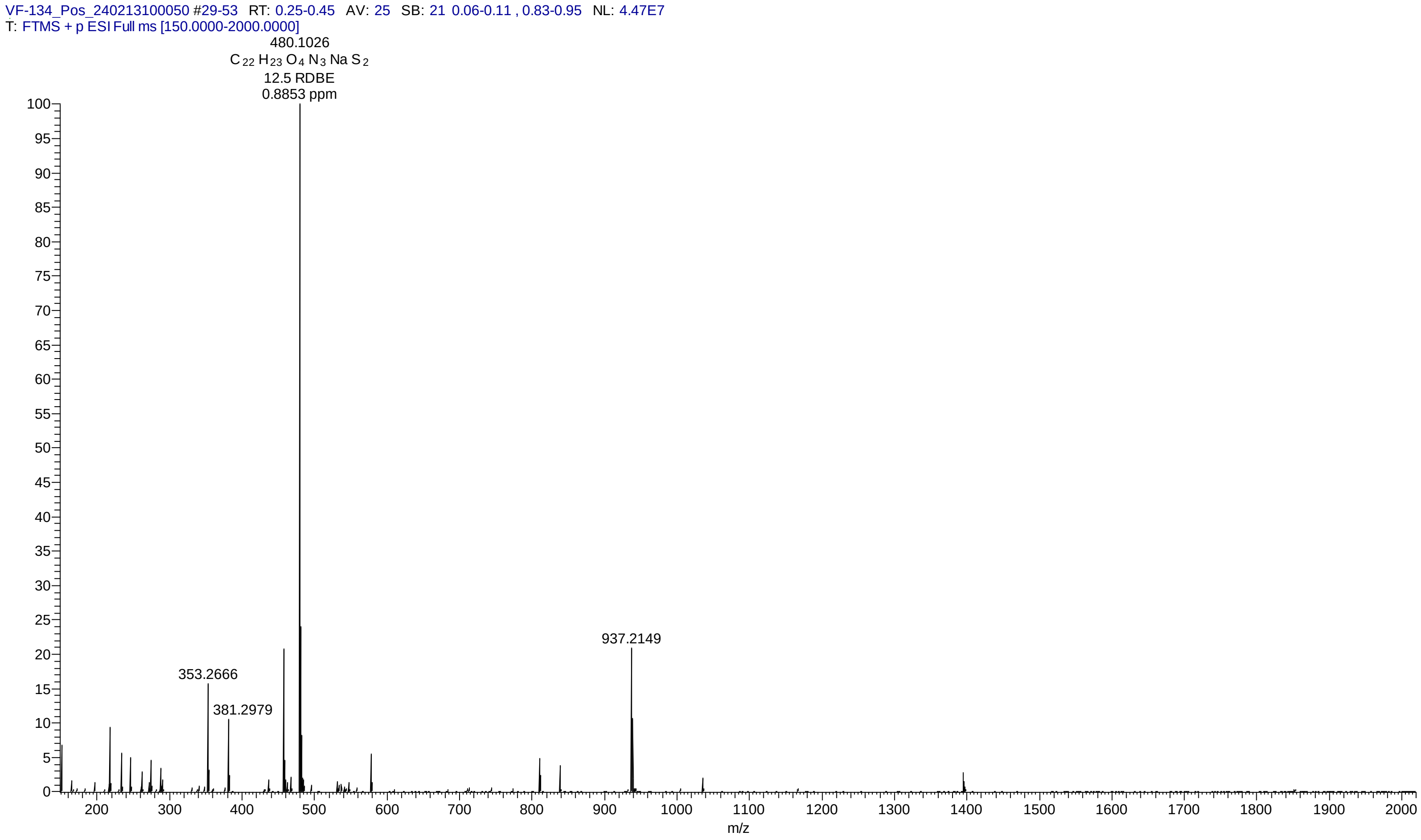

VF-134_Pos_240213100050 #29-53 RT: 0.25-0.45 AV: 25 SB: 21 0.06-0.11 , 0.83-0.95 NL: 4.47E7
T: FTMS + p ESI Full ms [150.0000-2000.0000]
480.1026
C 22 H 23 O 4 N 3 Na S 2
12.5 RDBE
0.8853 ppm
353.2666
381.2979
937.2149
m/z

^{1}H NMR ($CDCl_3$) spectrum of **3na**

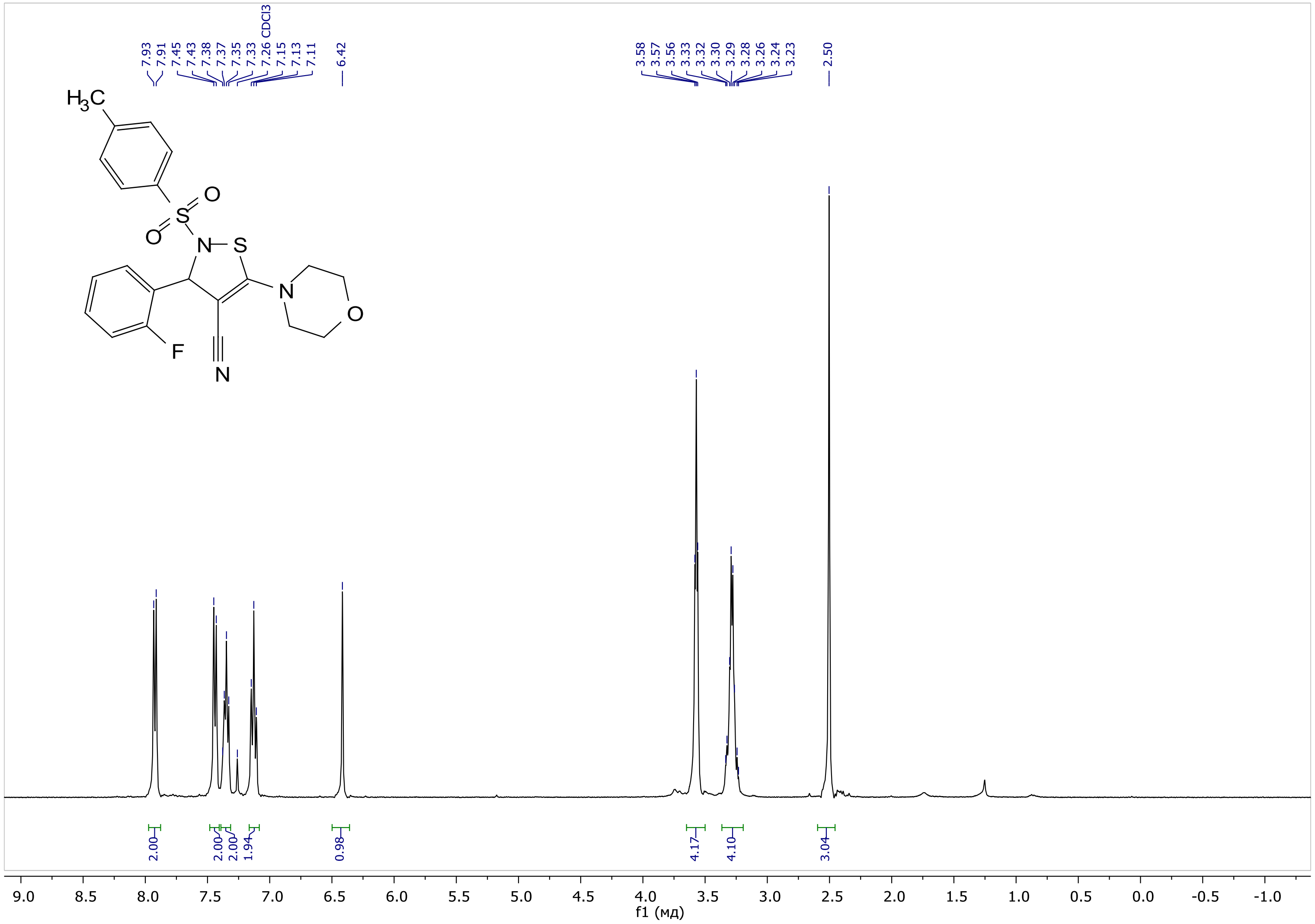

^{19}F NMR ($CDCl_3$) spectrum of **3na**

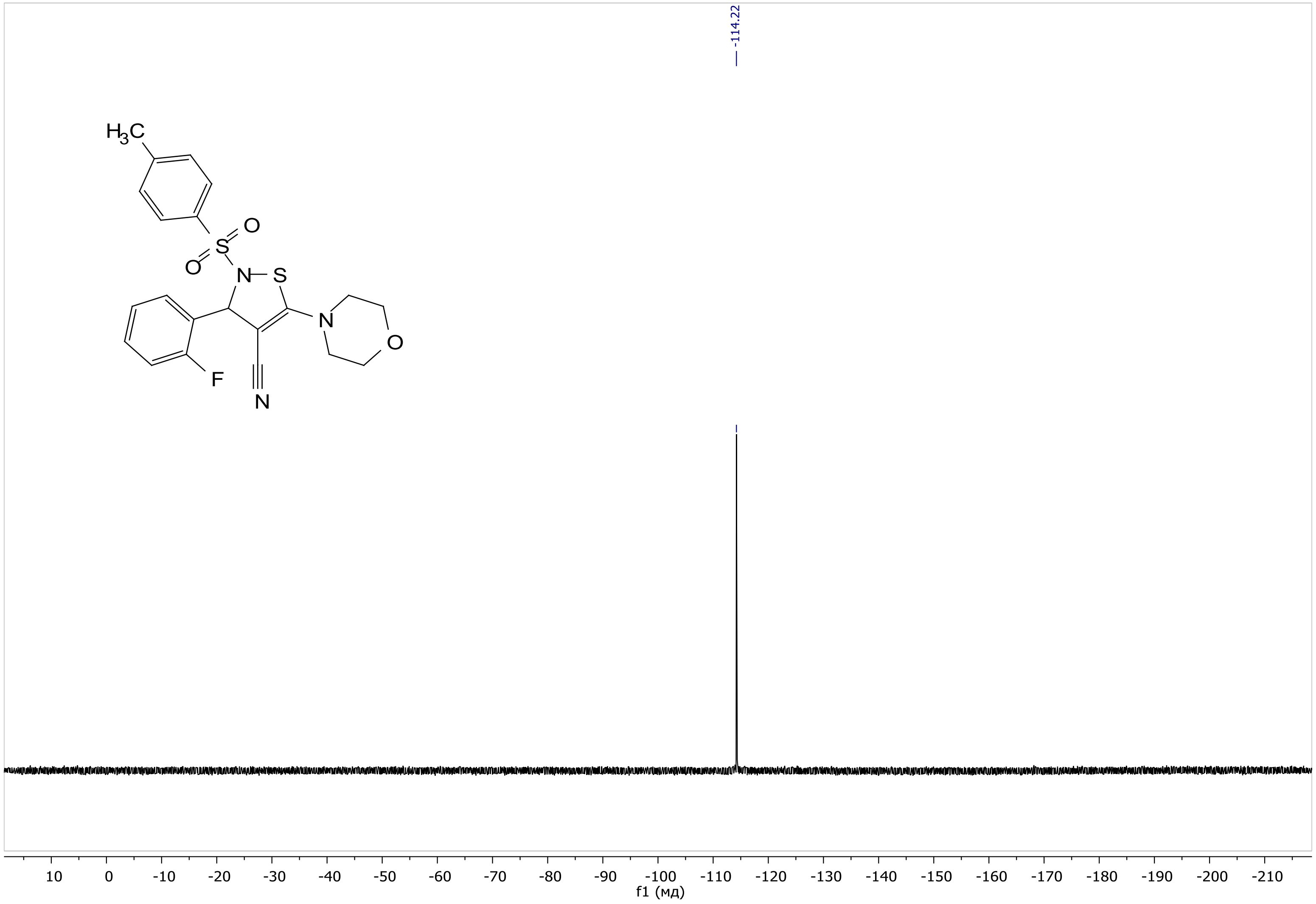

^{13}C NMR (DMSO-d_6) spectrum of **3na**

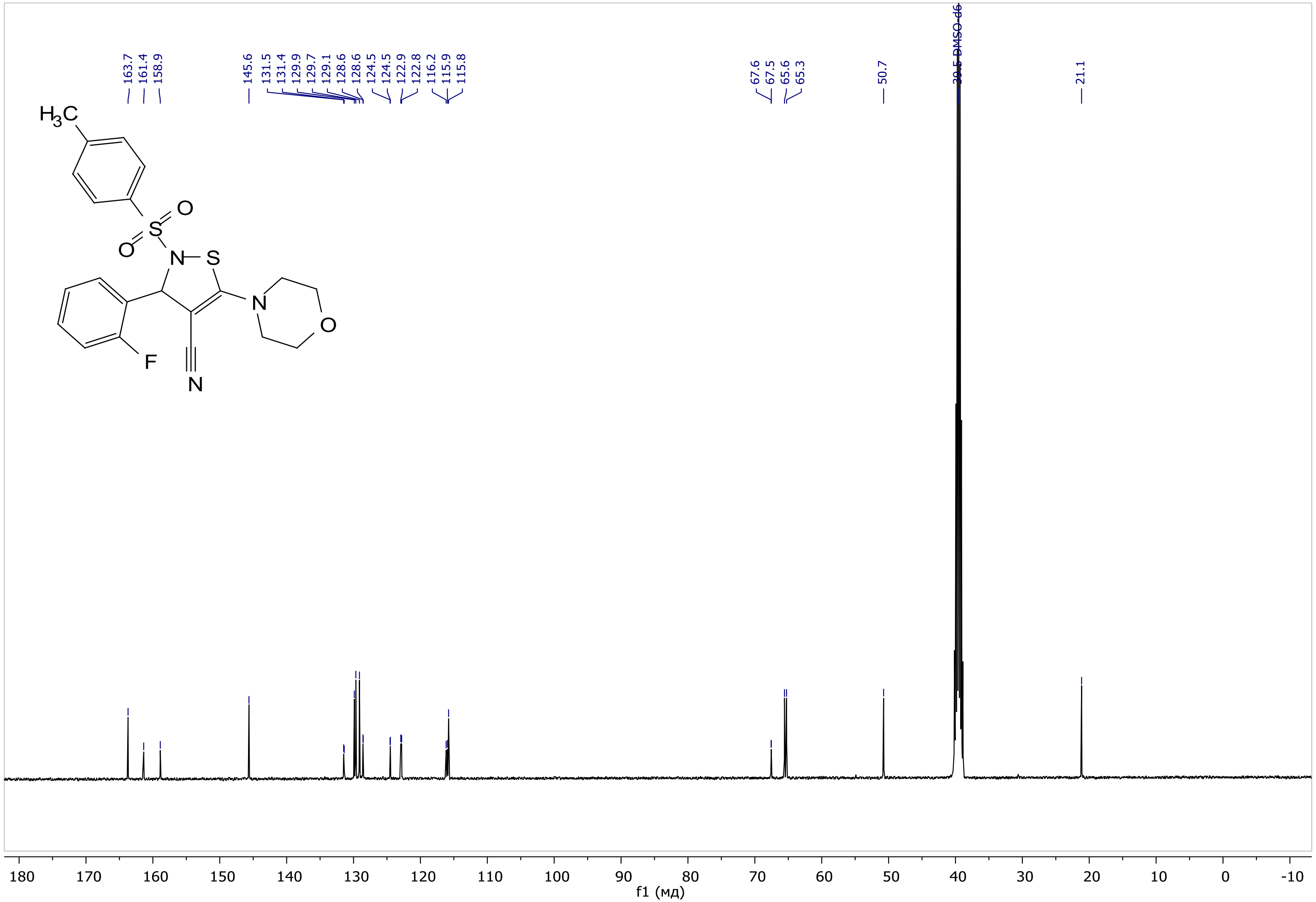

HRMS of **3na**

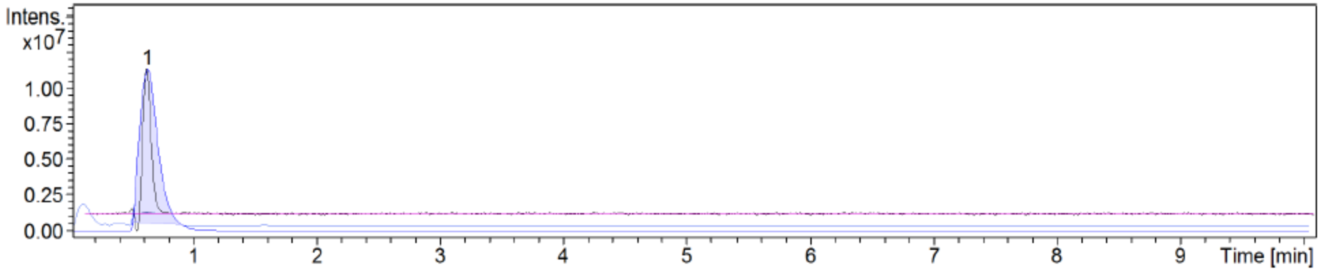

#	RT [min]	Area	Int. Type	I	S/N	Chromatogram	Max. m/z	FWHM [min]
1	0.6	108223808	Manual	11257631	31118.0	EIC 446.0987±0.02 +All MS	446.1007	

Cmpd 1, 0.6 min

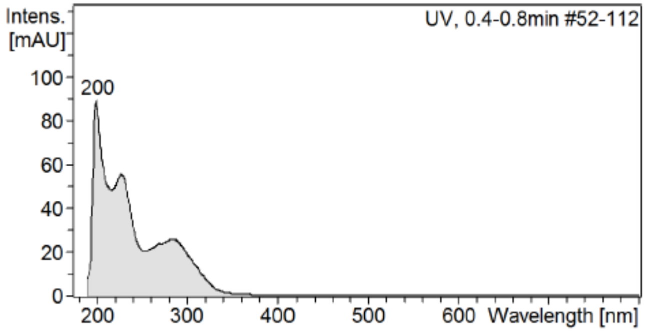

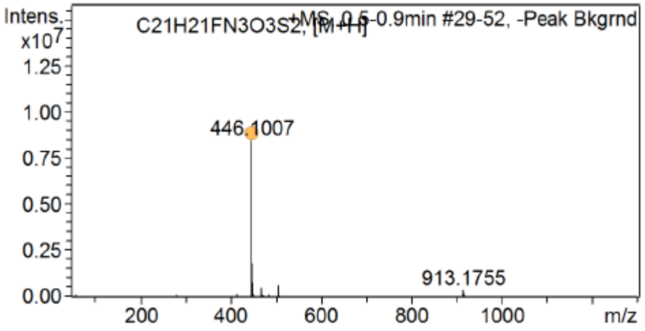

#	Wavelength	Intensity
0	200	89.1

#	m/z	Res.	S/N	I	I %	FWHM
1	414.1263	21333	19514274.0	146872	1.7	0.0194
2	446.1007	39592	1120244096.0	8431403	100.0	0.0113
3	447.1037	32163	238496928.0	1795023	21.3	0.0139
4	448.0992	24305	96361864.0	725258	8.6	0.0184
5	449.1003	19451	18586732.0	139891	1.7	0.0231
6	468.0823	25387	64444732.0	485037	5.8	0.0184
7	504.1536	27257	80141016.0	603173	7.2	0.0185
8	505.1559	21139	20727910.0	156007	1.9	0.0239
9	913.1755	30914	44036976.0	331440	3.9	0.0295
10	914.1780	26238	21774960.0	163887	1.9	0.0348

^{1}H NMR ($CDCl_3$) spectrum of **3oa**

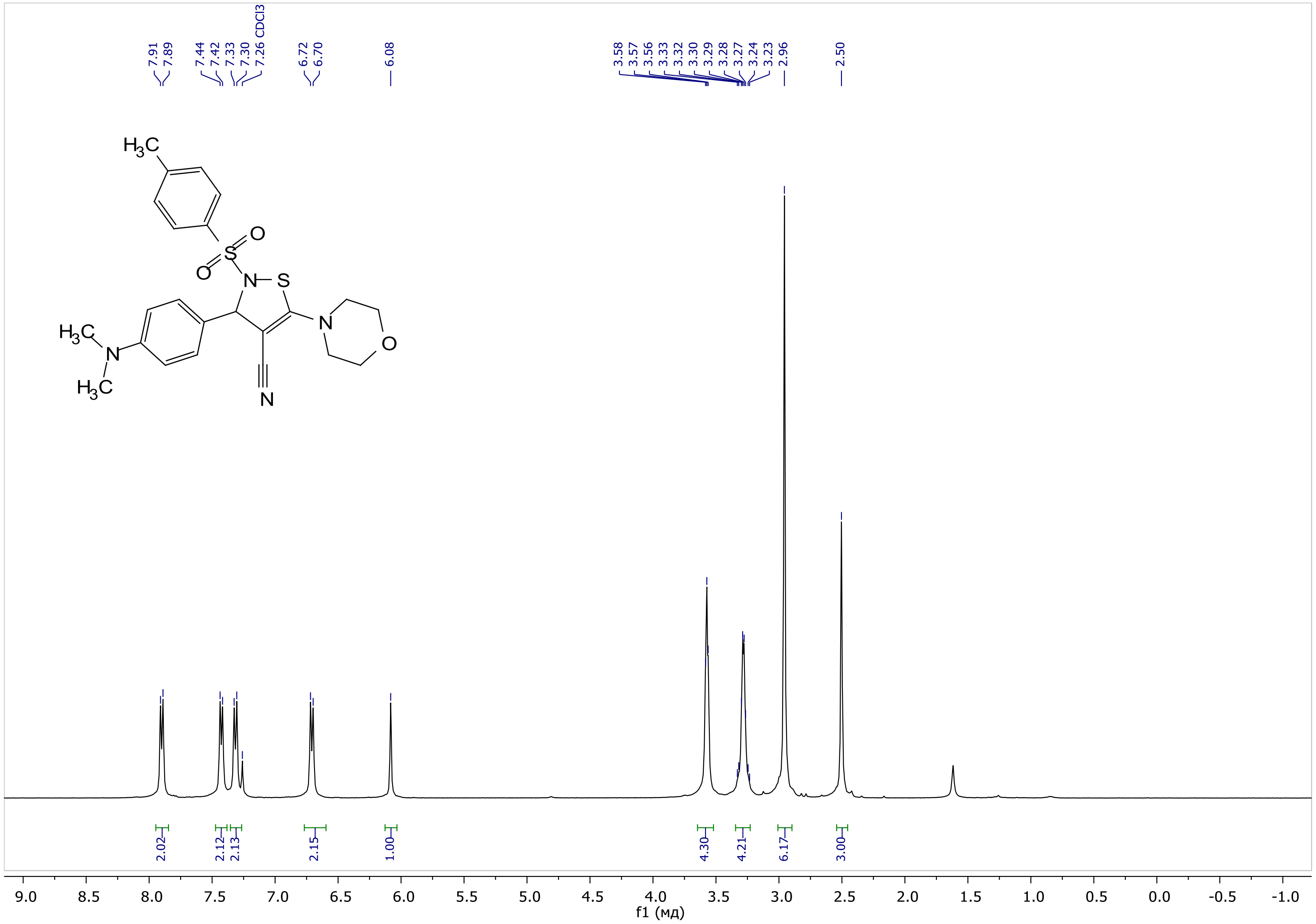

^{13}C NMR ($CDCl_3$) spectrum of **3oa**

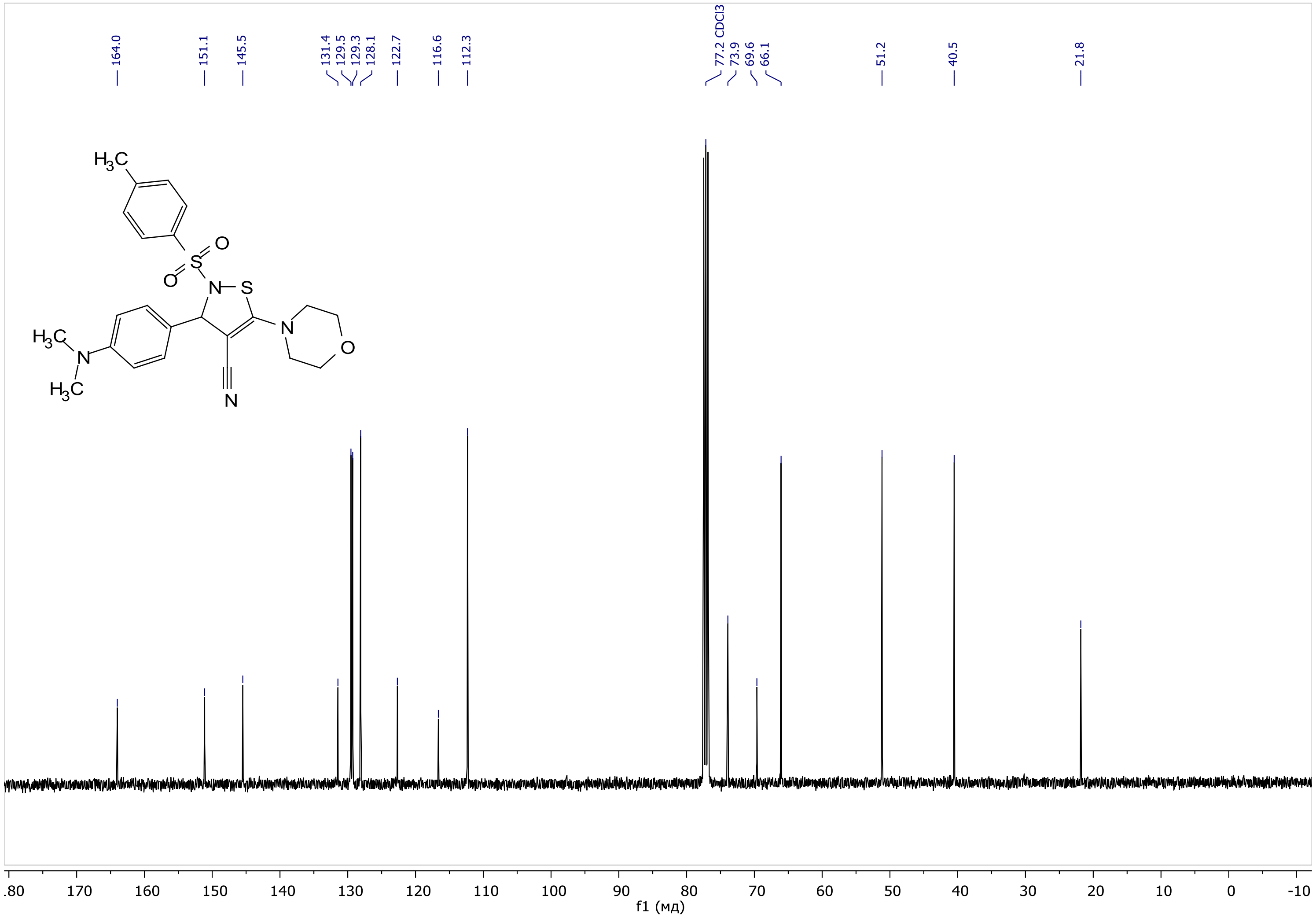

HRMS of **3oa**

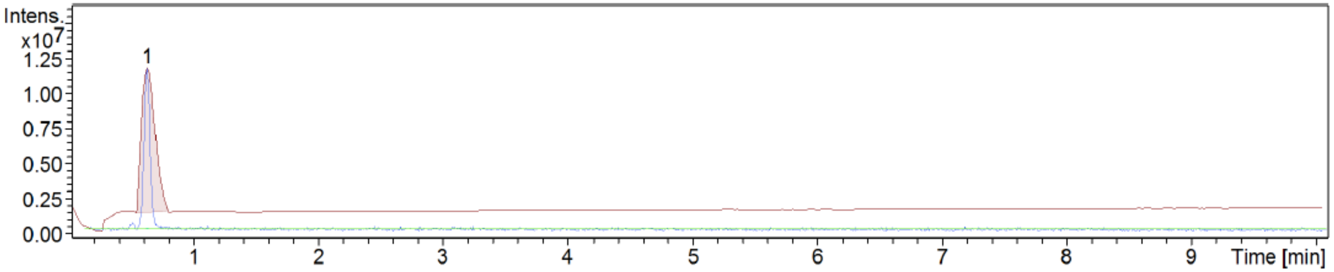

#	RT [min]	Area	Int. Type	I	S/N	Chromatogram	Max. m/z	FWHM [min]
1	0.6	77795384	Manual	11749728	508.0	BPC +All MS	471.1519	

Cmpd 1, 0.6 min

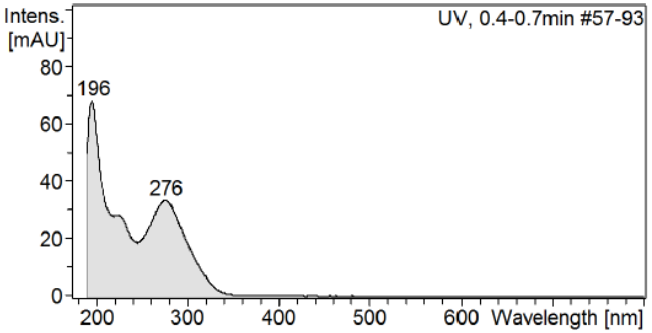

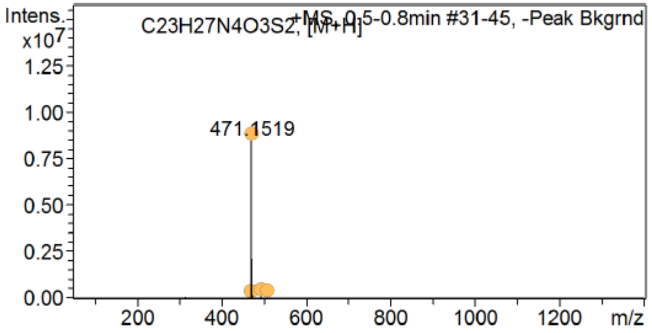

#	Wavelength	Intensity
0	196	67.7
1	276	33.3

#	m/z	Res.	S/N	I	I %	FWHM
1	315.1266	19351	3658764.5	72284	0.9	0.0163
2	471.1519	30538	425545344.0	8407209	100.0	0.0154
3	471.5521	17796	1992938.8	39373	0.5	0.0265
4	472.1545	34456	108452792.0	2142628	25.5	0.0137
5	473.1504	25053	40309732.0	796372	9.5	0.0189
6	474.1510	20093	8637641.0	170648	2.0	0.0236
7	475.1492	15902	1846111.5	36472	0.4	0.0299
8	493.1325	20663	4803625.5	94902	1.1	0.0239
9	509.1063	19020	1972855.4	38976	0.5	0.0268
10	551.0909	20404	1952696.3	38578	0.5	0.0270

^{1}H NMR ($CDCl_3$) spectrum of **3pa**

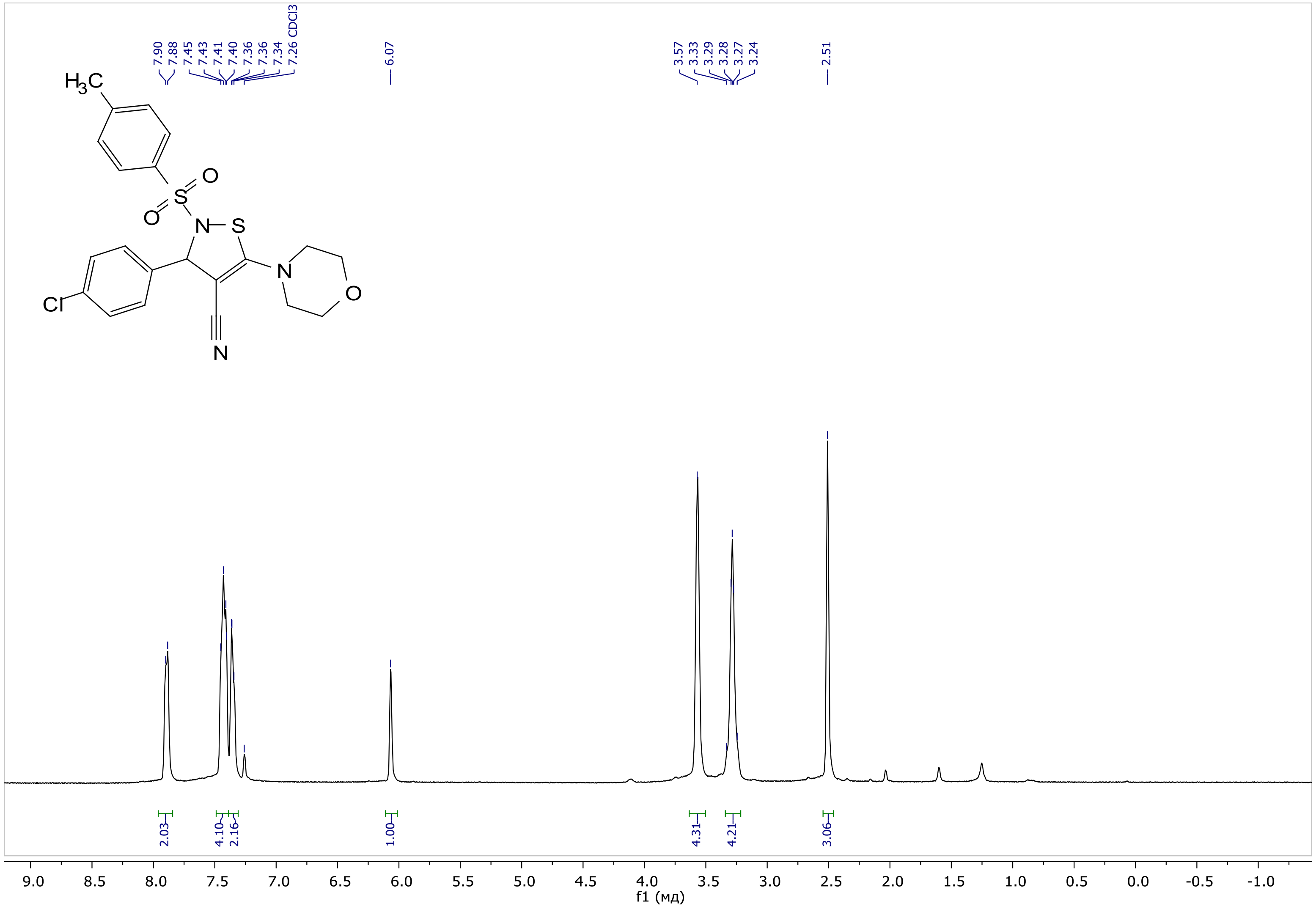

^{13}C NMR ($CDCl_3$) spectrum of **3pa**

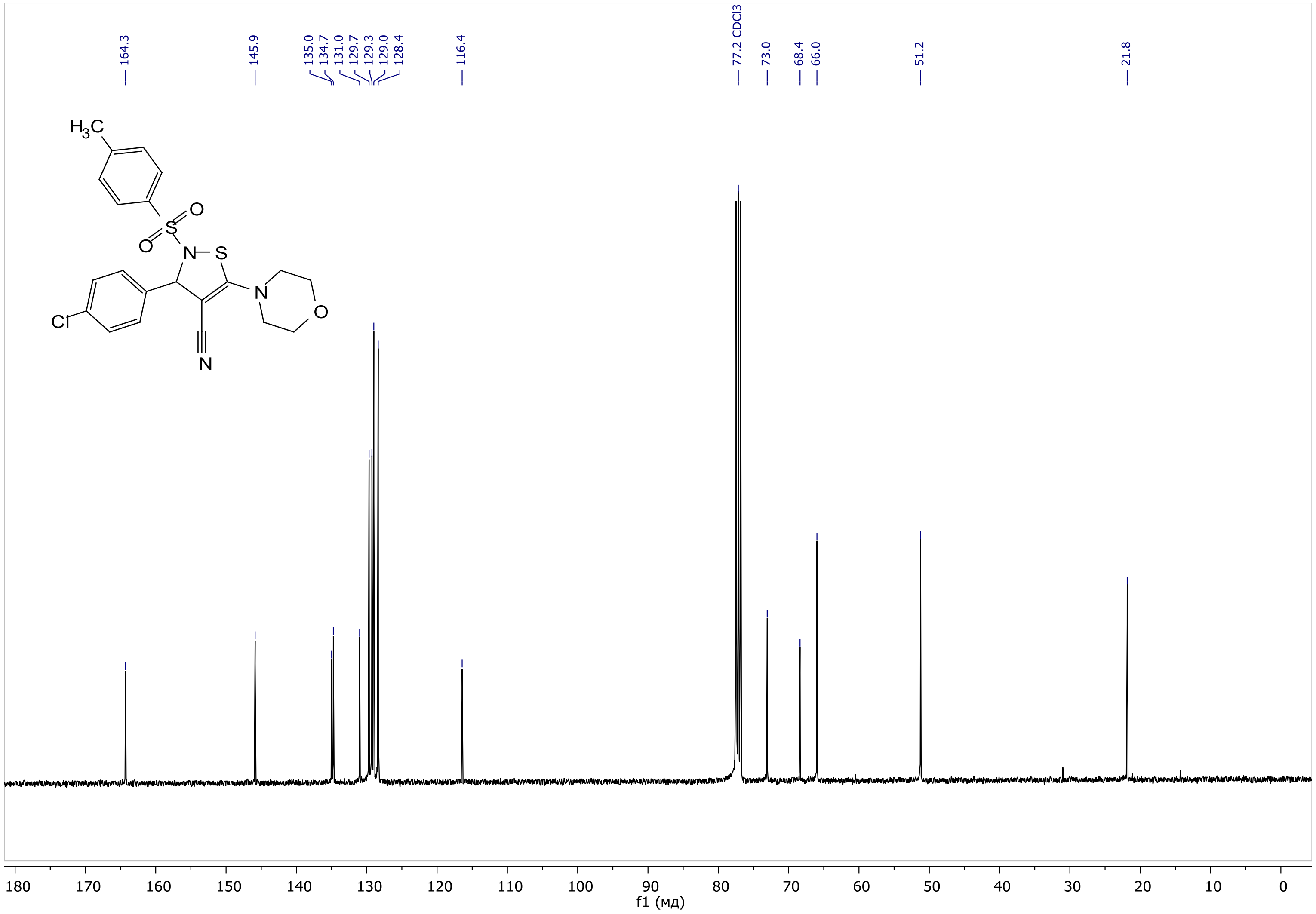

HRMS of **3pa**

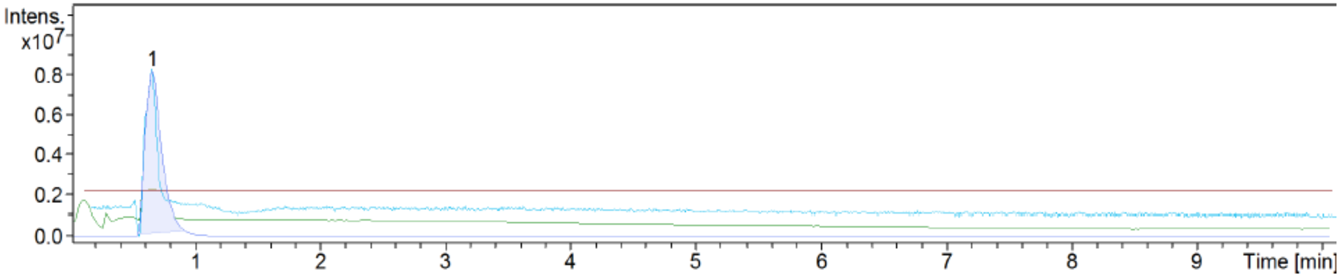

#	RT [min]	Area	Int. Type	I	S/N	Chromatogram	Max. m/z	FWHM [min]
1	0.7	72452992	Manual	8179648	40750.3	EIC 442.1233±0.02 +All MS	442.1257	

Cmpd 1, 0.7 min

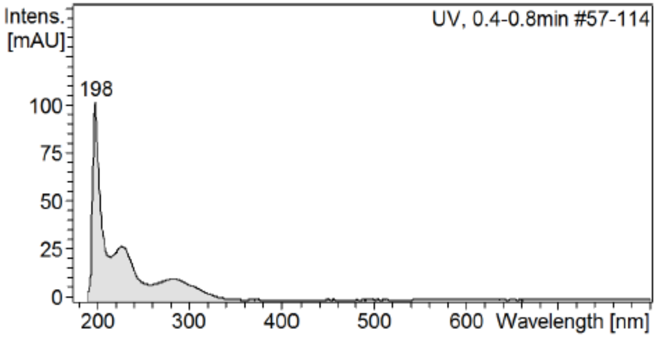

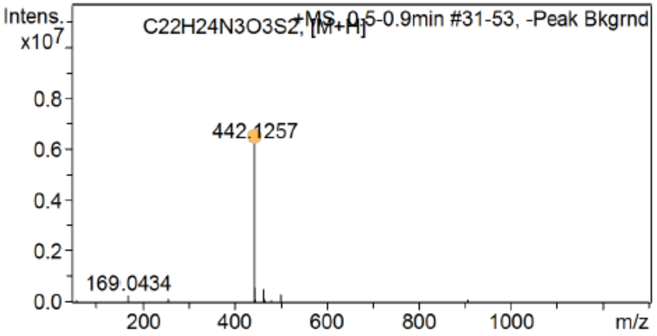

#	Wavelength	Intensity
0	198	101.4

#	m/z	Res.	S/N	I	I %	FWHM
1	169.0434	20831	31916674.0	259728	4.2	0.0081
2	257.1646	20920	14570147.0	118568	1.9	0.0123
3	442.1257	40945	763024832.0	6209270	100.0	0.0108
4	443.1288	31605	173096464.0	1408608	22.7	0.0140
5	444.1243	22423	65815572.0	535588	8.6	0.0198
6	445.1249	19097	13757497.0	111954	1.8	0.0233
7	464.1075	25271	61675796.0	501899	8.1	0.0184
8	465.1099	20233	15876154.0	129195	2.1	0.0230
9	500.1787	23353	36161592.0	294272	4.7	0.0214
10	905.2246	24564	12844662.0	104526	1.7	0.0369

^{1}H NMR ($CDCl_3$) spectrum of **3qa**

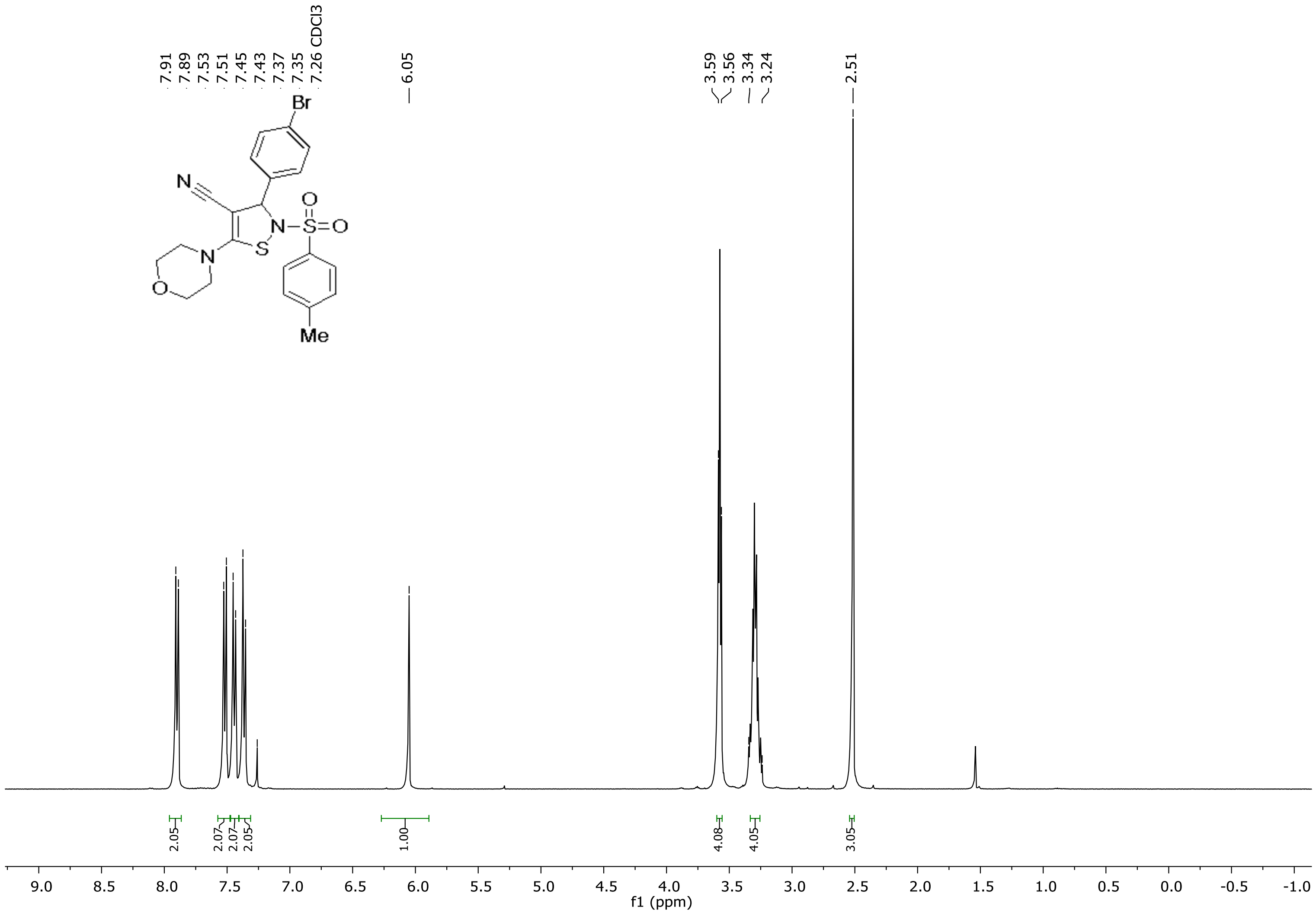

^{13}C NMR ($CDCl_3$) spectrum of **3qa**

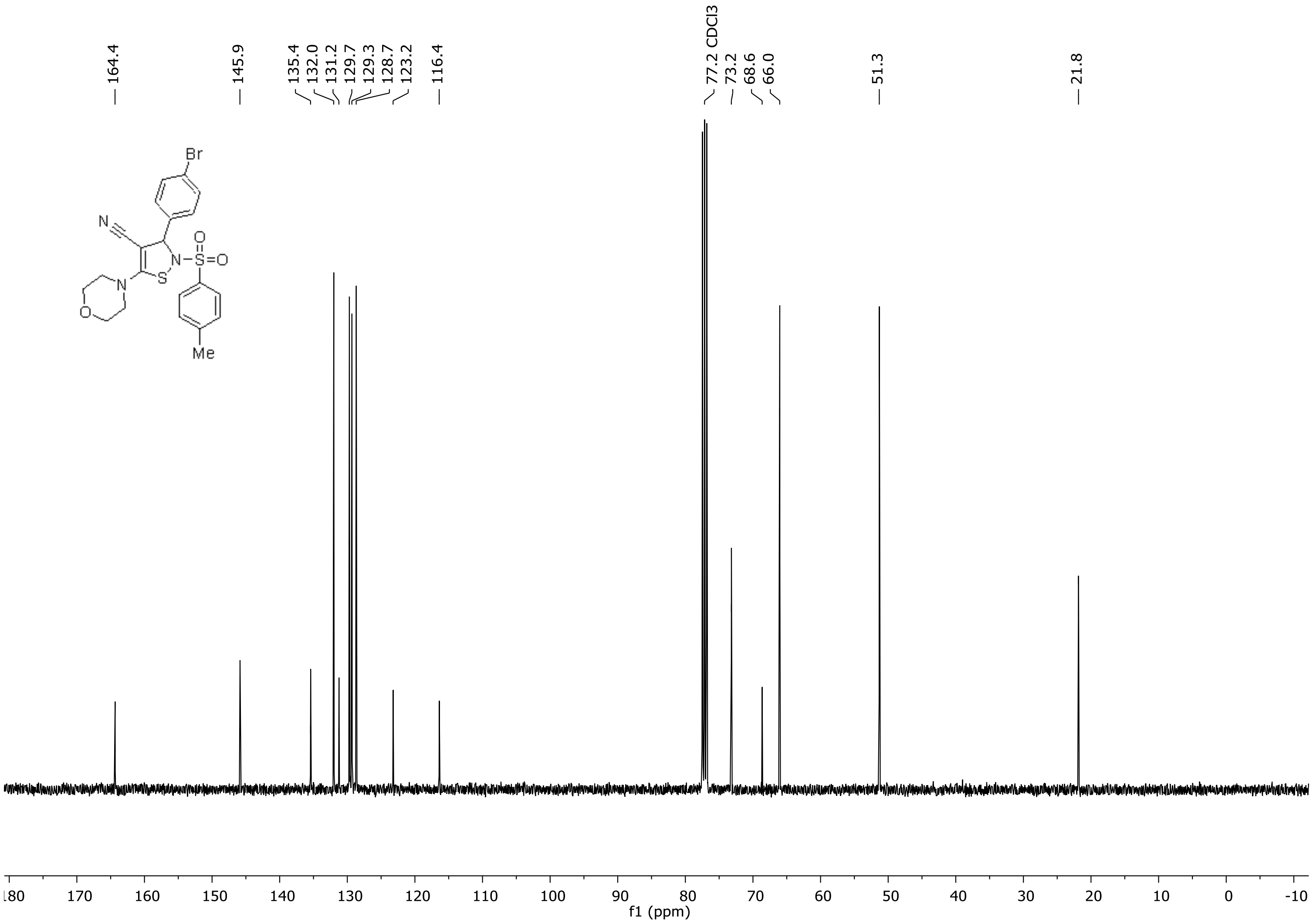

HRMS of **3qa**

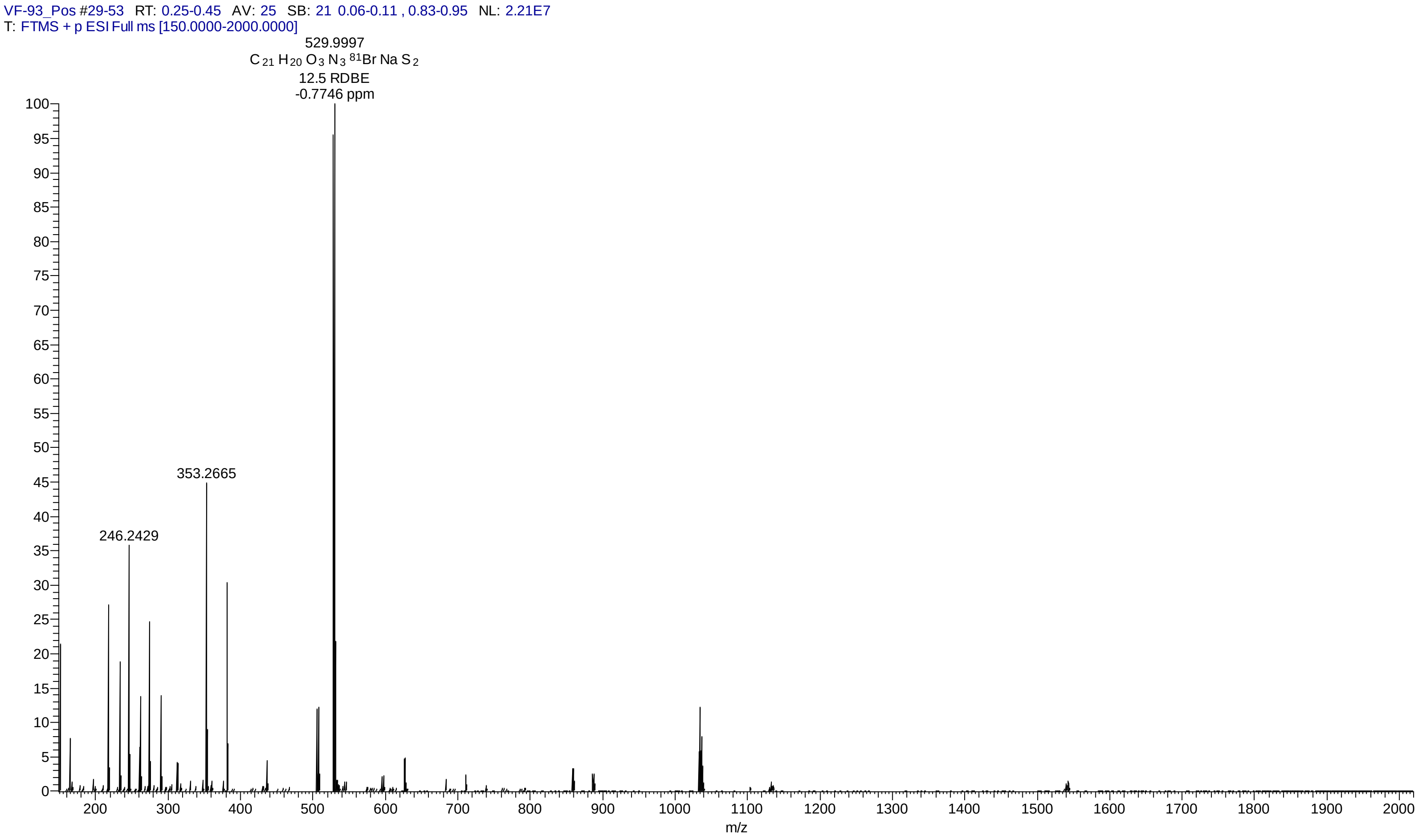

VF-93_Pos #29-53 RT: 0.25-0.45 AV: 25 SB: 21 0.06-0.11 , 0.83-0.95 NL: 2.21E7
T: FTMS + p ESI Full ms [150.0000-2000.0000]
529.9997
C 21 H 20 O 3 N 3 81Br Na S 2
12.5 RDBE
-0.7746 ppm
353.2665
246.2429
m/z

^{1}H NMR ($CDCl_3$) spectrum of **3ra**

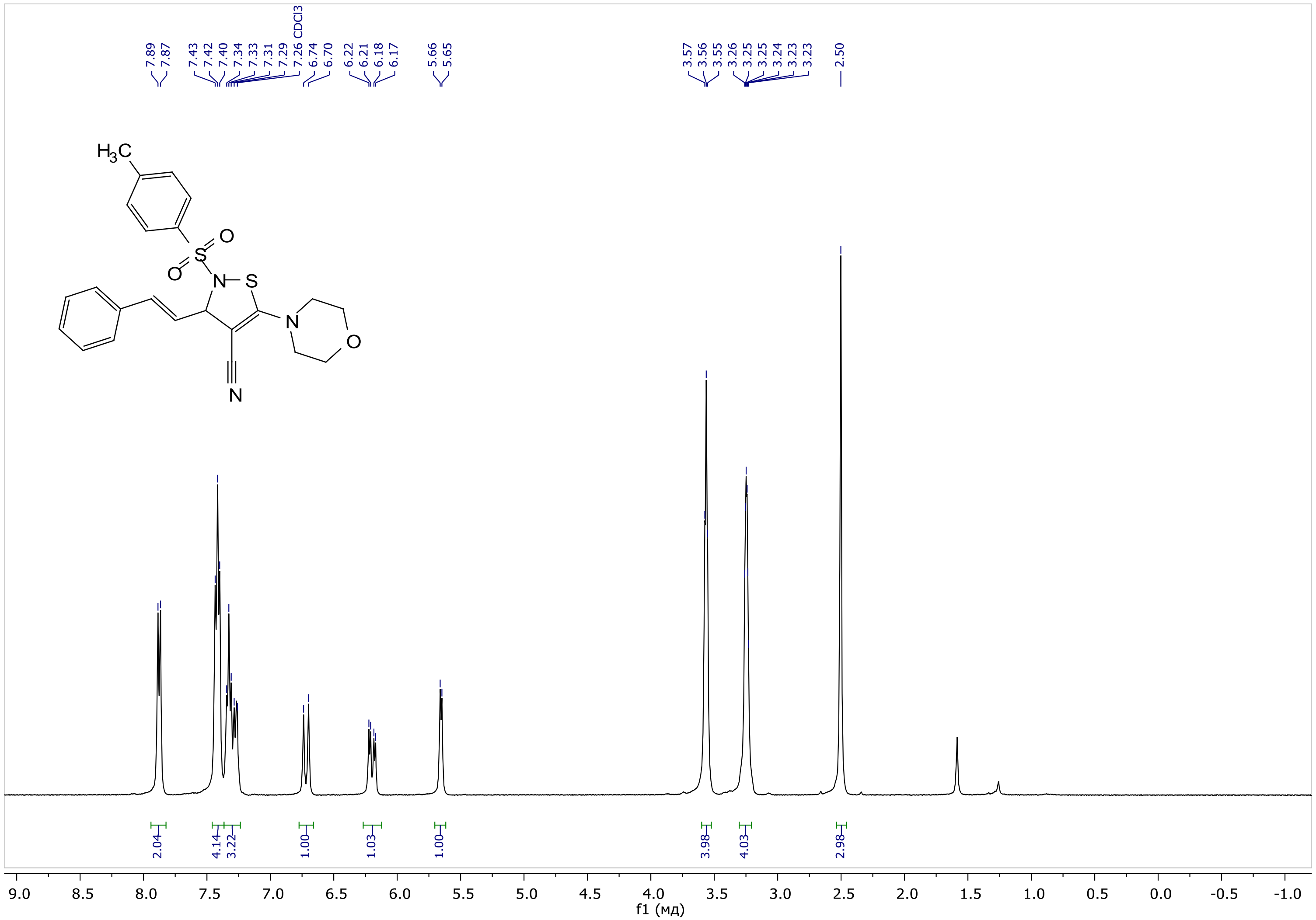

^{13}C NMR ($CDCl_3$) spectrum of **3ra**

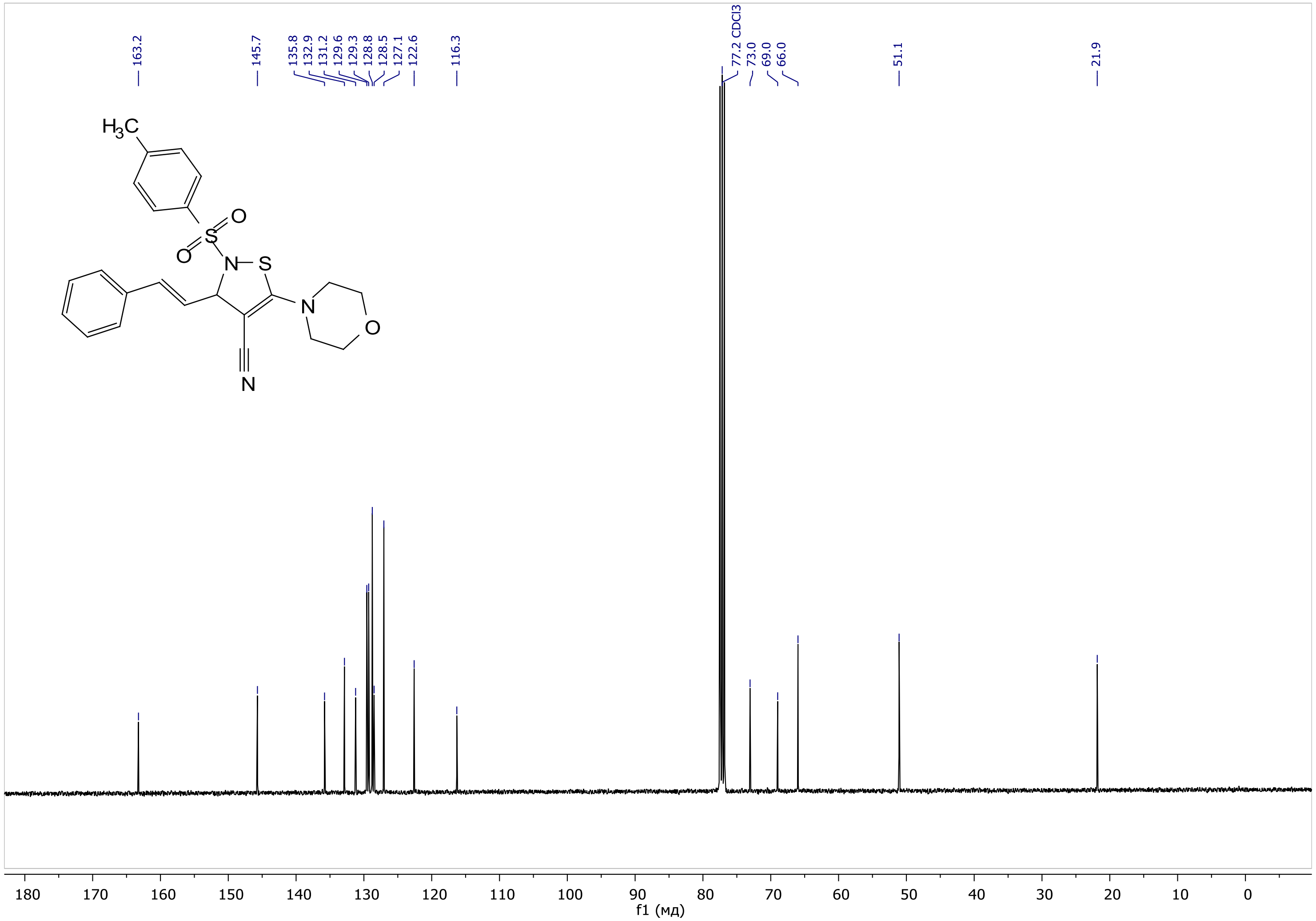

HRMS of **3ra**

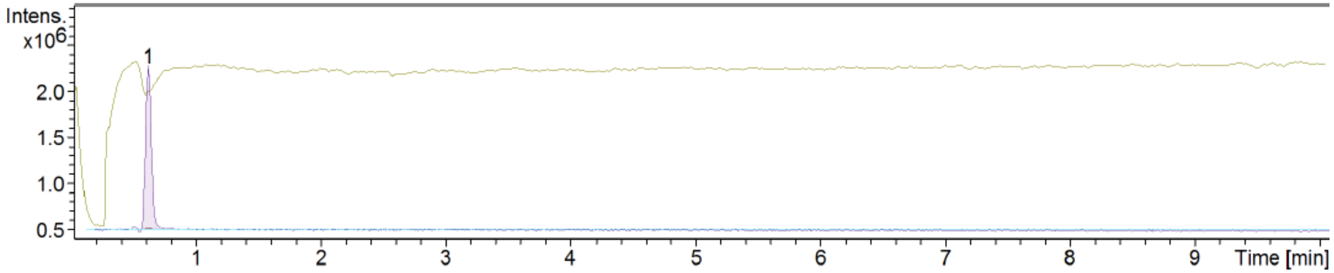

#	RT [min]	Area	Int. Type	I	S/N	Chromatogram	Max. m/z	FWHM [min]
1	0.6	194073	Manual	60477	148.0	UV Chromatogram, 190-800 nm	454.1255	

Cmpd 1, 0.6 min

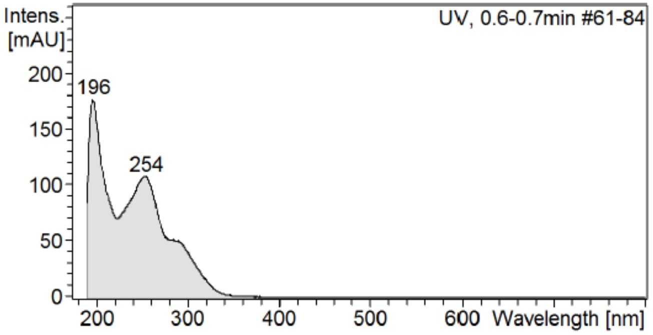

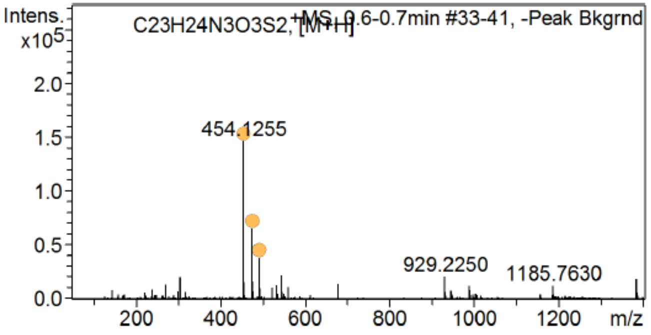

#	Wavelength	Intensity
0	196	175.8
1	254	107.3

#	m/z	Res.	S/N	I	I %	FWHM
1	305.3022	19665	4065745.3	20023	13.7	0.0155
2	454.1255	23848	29686678.0	146200	100.0	0.0190
3	455.1280	20462	7653192.0	37690	25.8	0.0222
4	476.1067	21291	13292856.0	65464	44.8	0.0224
5	477.1098	18720	3275426.3	16131	11.0	0.0255
6	492.0807	20734	7795246.5	38390	26.3	0.0237
7	544.0940	20268	4413087.0	21733	14.9	0.0268
8	929.2250	23177	4174060.3	20556	14.1	0.0401
9	1382.3414	23228	3737368.5	18406	12.6	0.0595
10	1383.3436	24091	3807002.0	18749	12.8	0.0574

^{1}H NMR (DMSO-d_6) spectrum of **3sa**

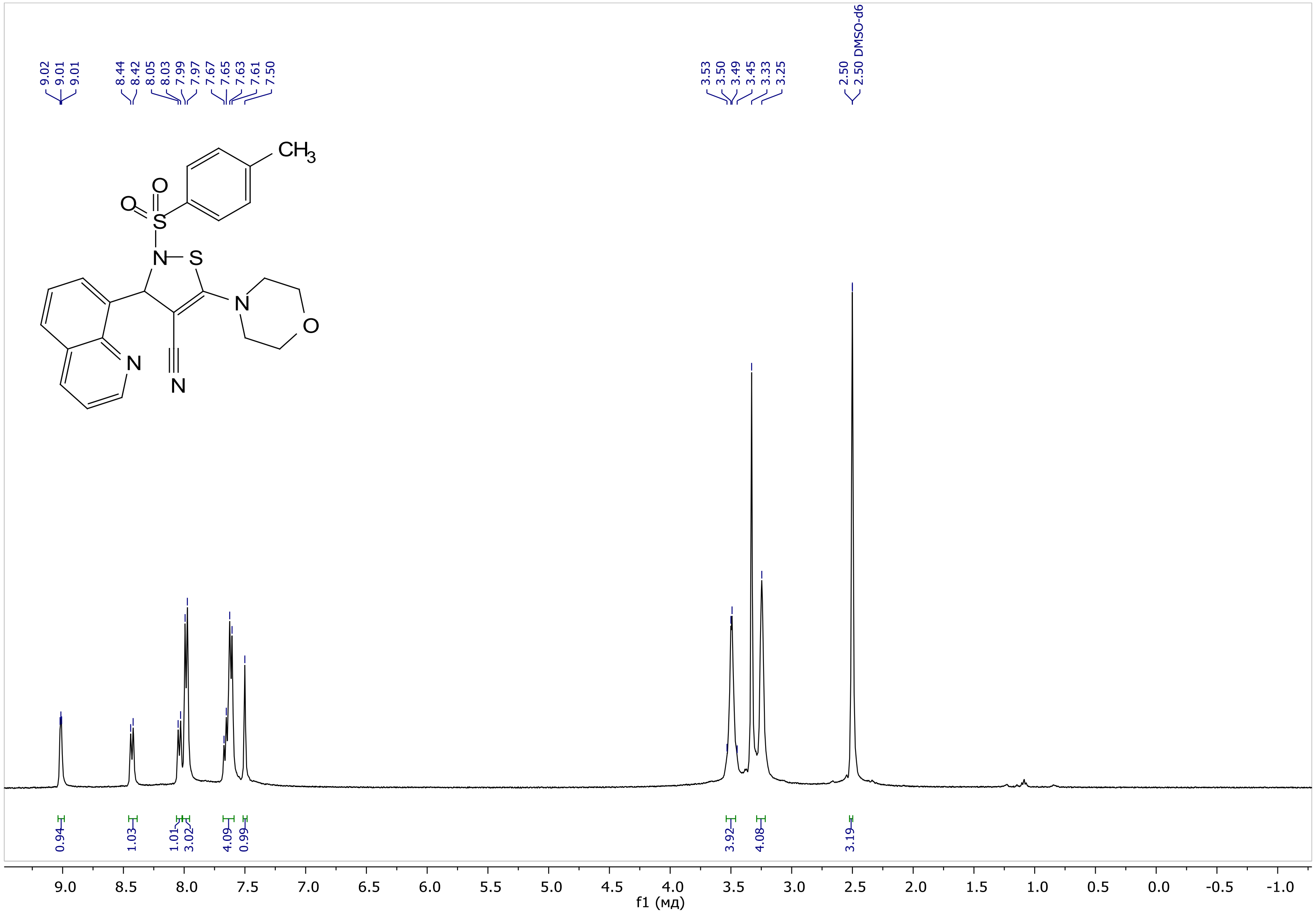

^{13}C NMR (DMSO-d_6) spectrum of **3sa**

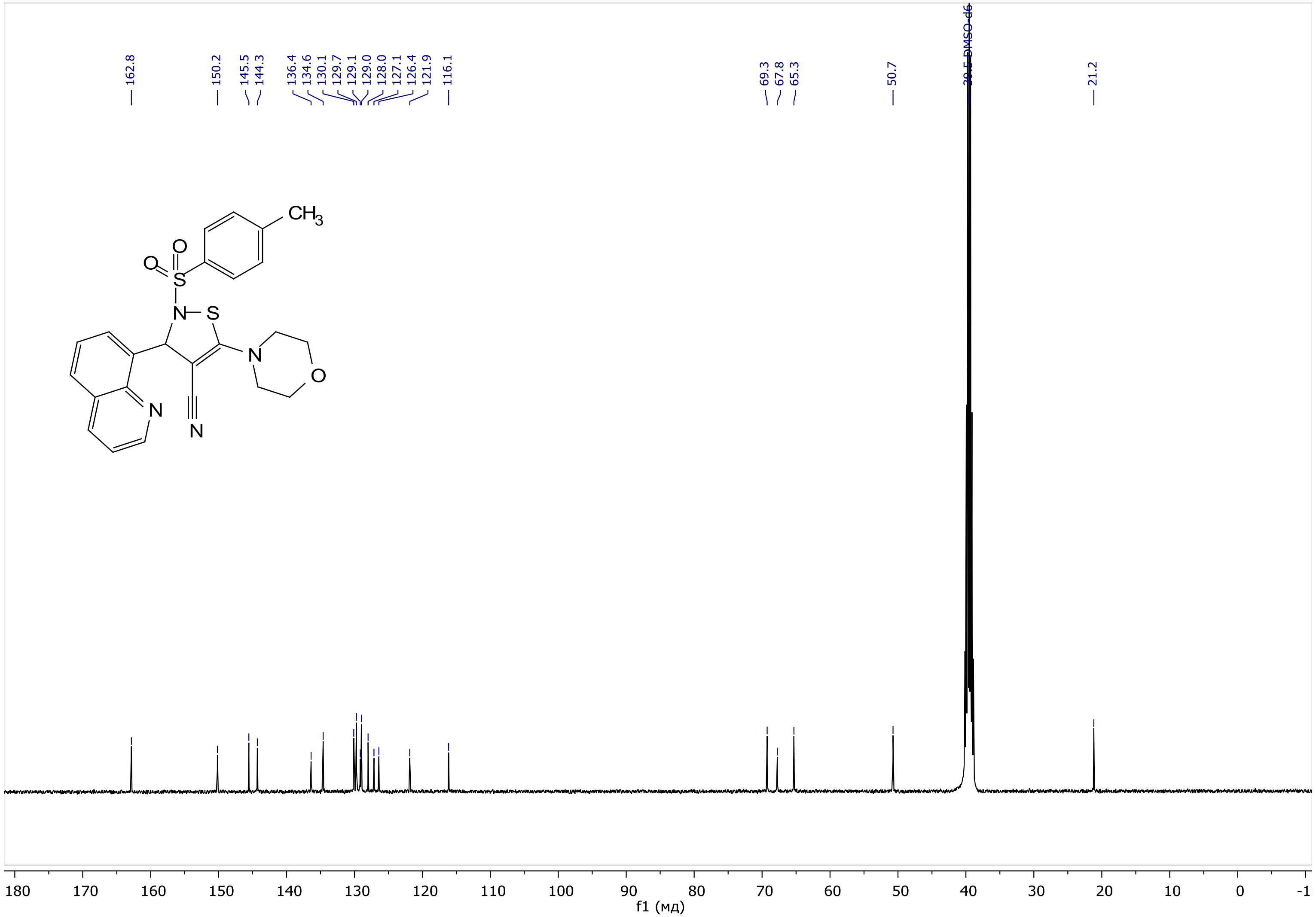

HRMS of **3sa**

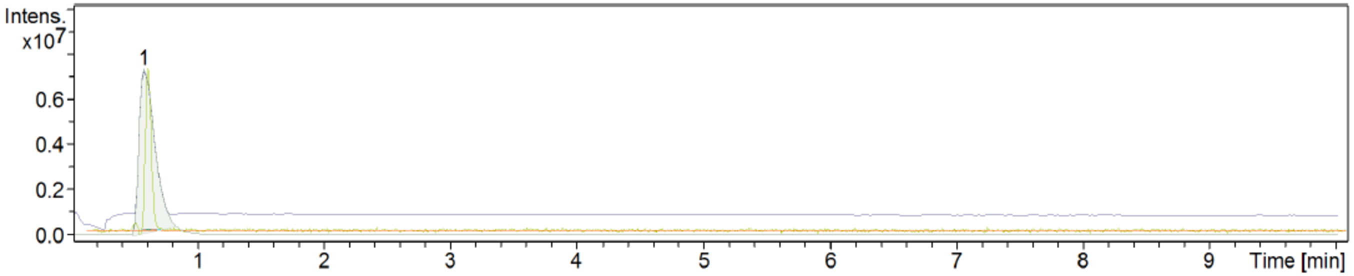

#	RT [min]	Area	Int. Type	I	S/N	Chromatogram	Max. m/z	FWHM [min]
1	0.6	63860744	Manual	7268343	3732.9	EIC 479.1205 +All MS	479.1205	

Cmpd 1, 0.6 min

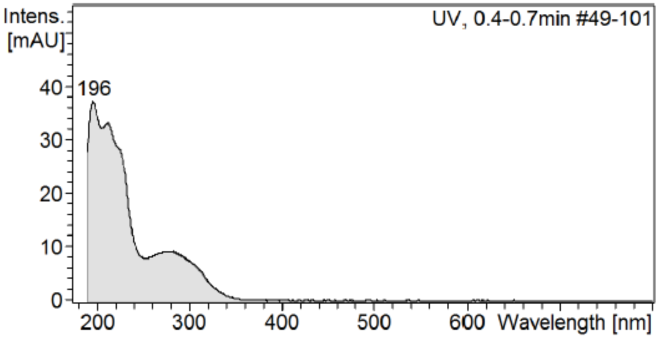

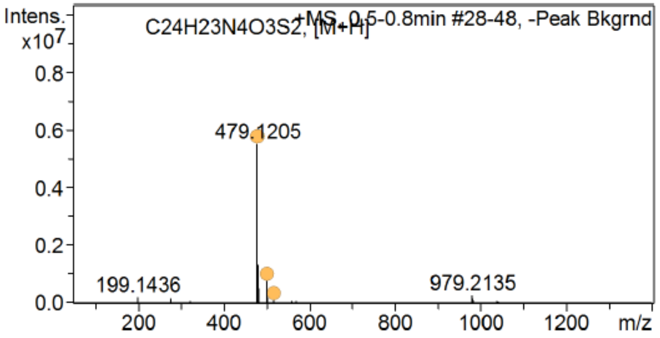

#	Wavelength	Intensity
0	196	37.2

#	m/z	Res.	S/N	I	I %	FWHM
1	199.1436	18758	8568632.0	203115	3.7	0.0106
2	277.1201	20394	6395646.5	151606	2.8	0.0136
3	479.1205	39483	231824176.0	5495283	100.0	0.0121
4	480.1233	30727	55933656.0	1325881	24.1	0.0156
5	481.1192	22353	21274806.0	504309	9.2	0.0215
6	482.1200	18624	4666859.0	110626	2.0	0.0259
7	501.1019	27211	31876052.0	755607	13.8	0.0184
8	502.1043	21451	8504620.0	201598	3.7	0.0234
9	979.2135	31310	10958886.0	259775	4.7	0.0313
10	980.2160	26529	6093268.0	144438	2.6	0.0369

^{1}H NMR ($CDCl_3$) spectrum of **3ta**

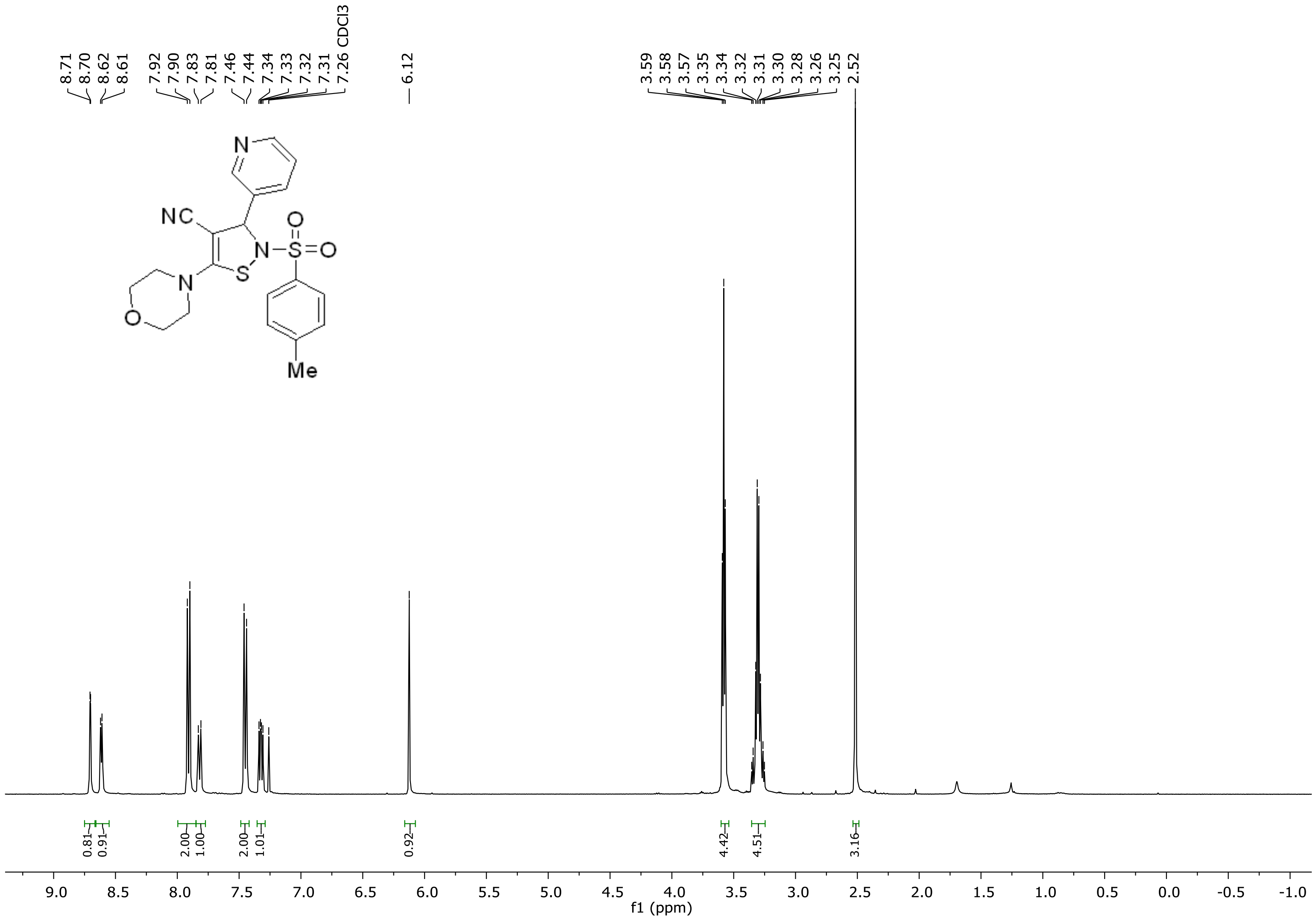

^{13}C NMR ($CDCl_3$) spectrum of **3ta**

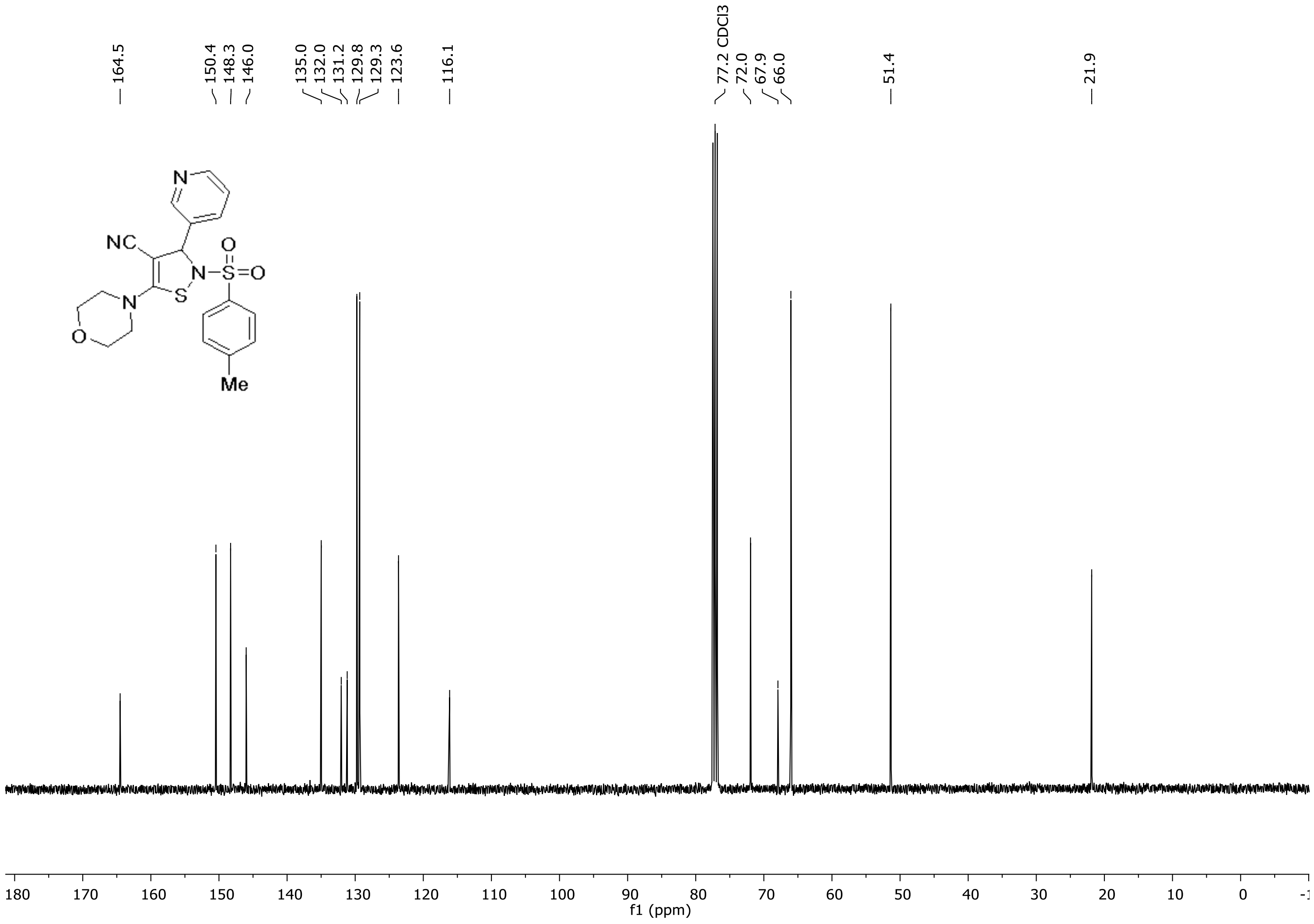

HRMS of **3ta**

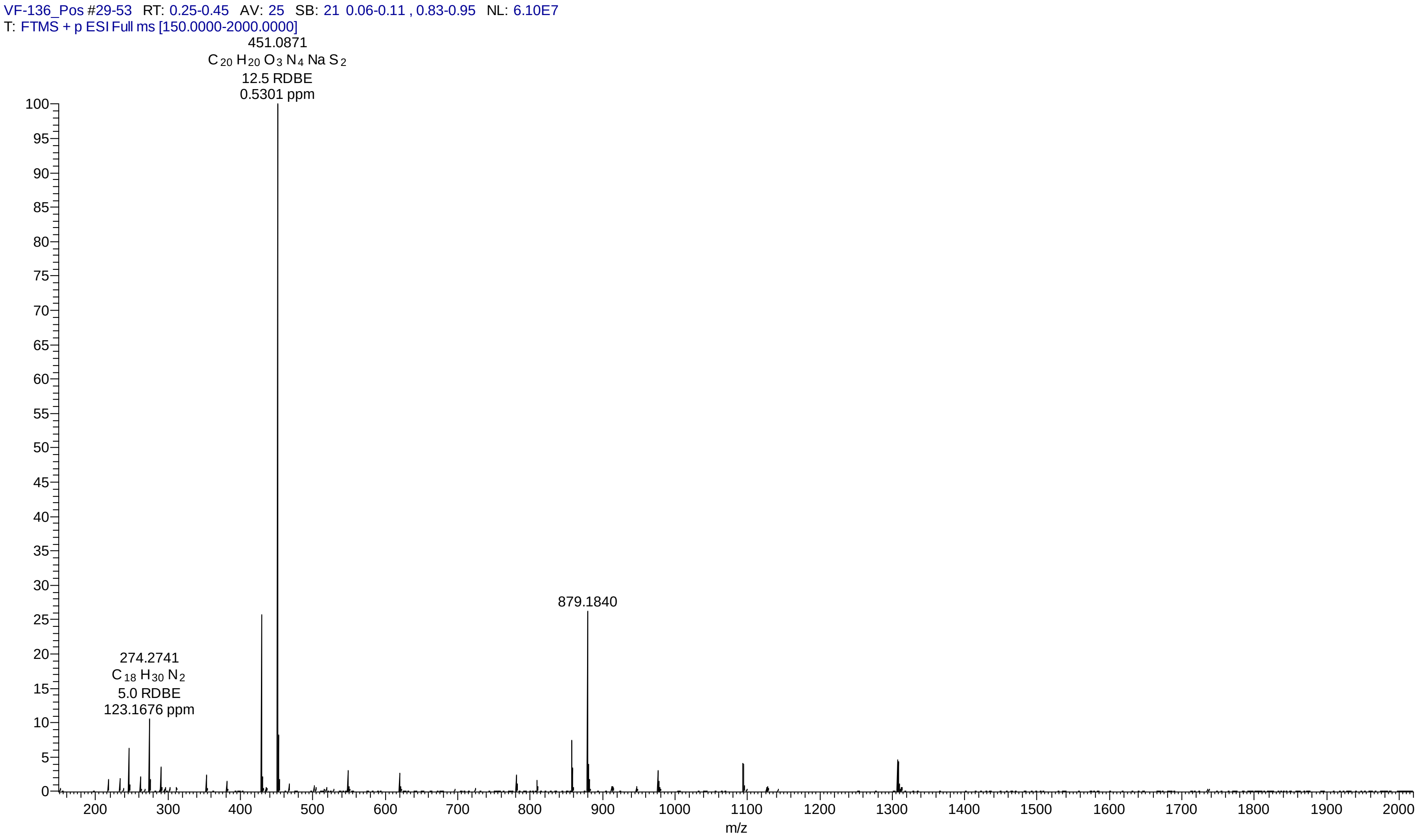

VF-136_Pos #29-53 RT: 0.25-0.45 AV: 25 SB: 21 0.06-0.11 , 0.83-0.95 NL: 6.10E7
T: FTMS + p ESI Full ms [150.0000-2000.0000]
451.0871
C 20 H 20 O 3 N 4 Na S 2
12.5 RDBE
0.5301 ppm
879.1840
274.2741
C 18 H 30 N 2
5.0 RDBE
123.1676 ppm
m/z

^{1}H NMR ($CDCl_3$) spectrum of **3ua**

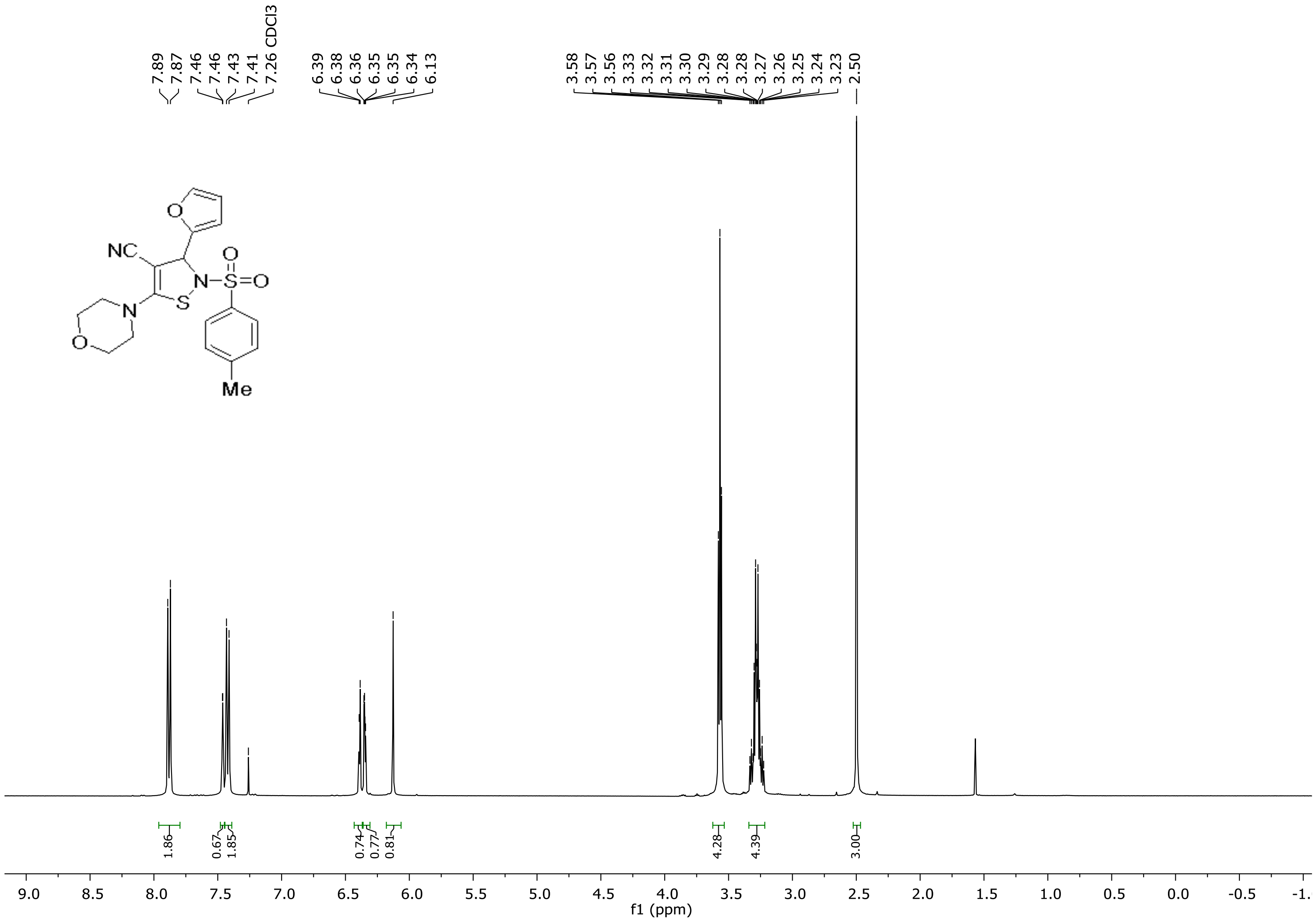

^{13}C NMR ($CDCl_3$) spectrum of **3ua**

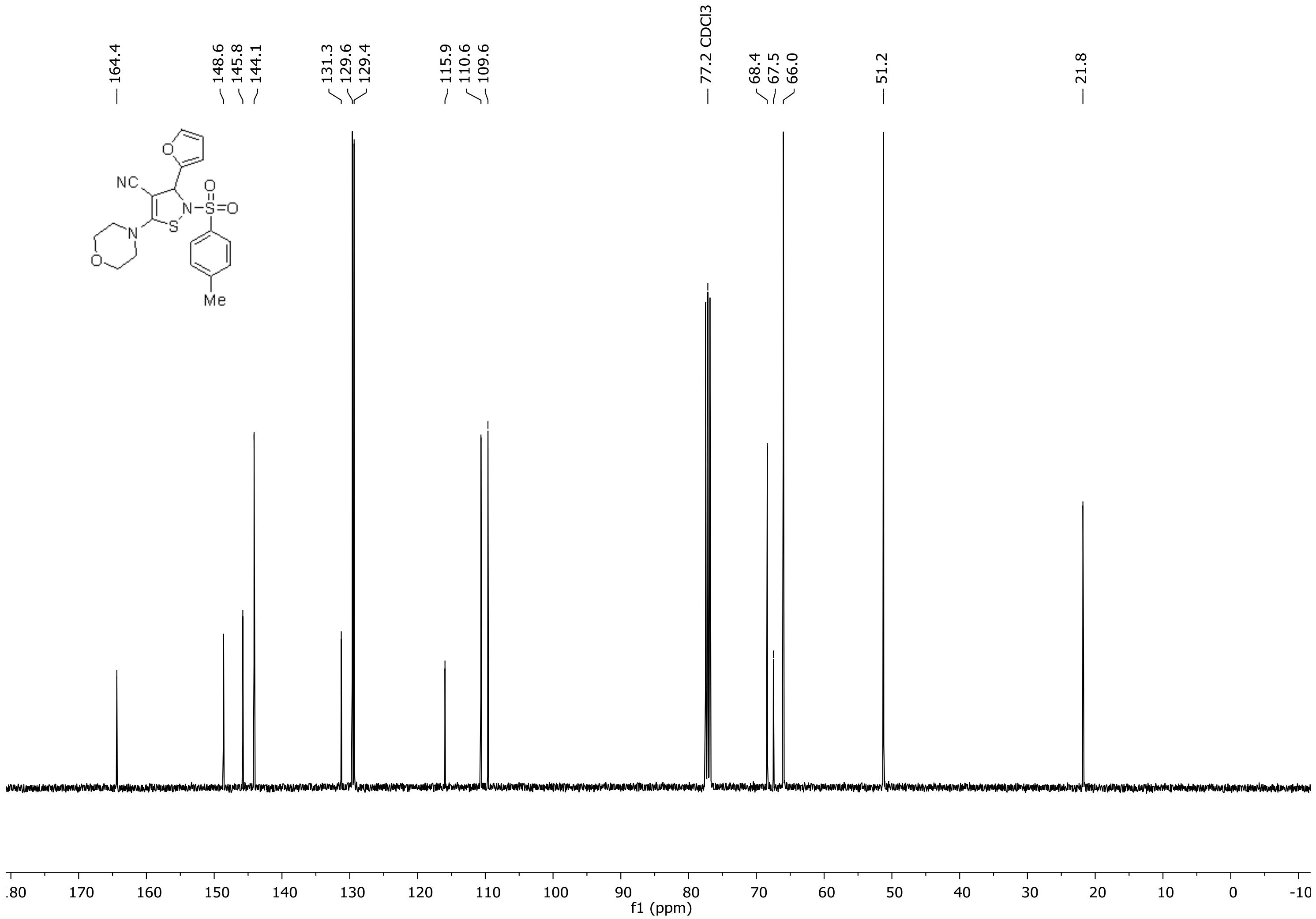

HRMS of **3ua**

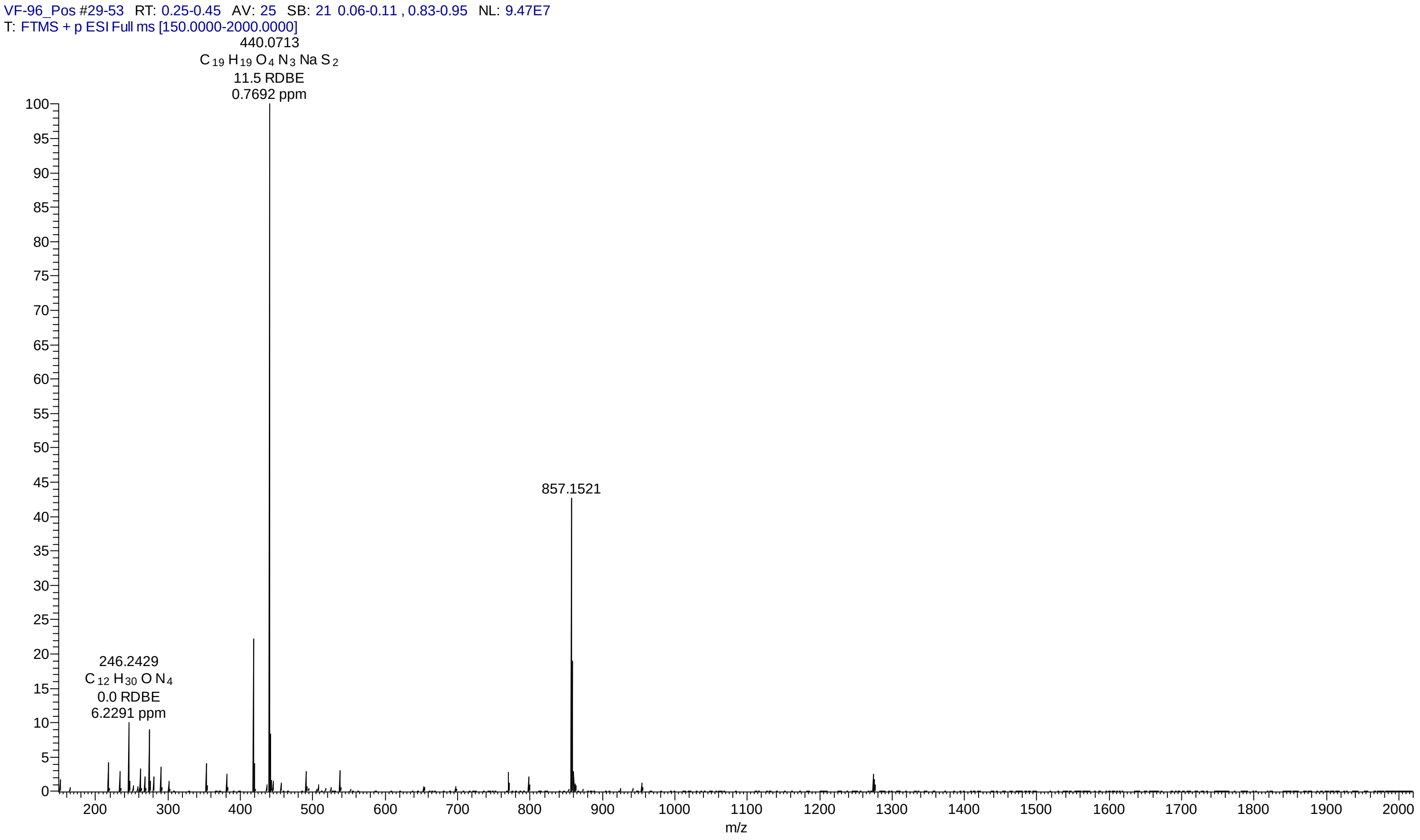

VF-96_Pos #29-53 RT: 0.25-0.45 AV: 25 SB: 21 0.06-0.11 , 0.83-0.95 NL: 9.47E7
T: FTMS + p ESI Full ms [150.0000-2000.0000]
440.0713
C 19 H 19 O 4 N 3 Na S 2
11.5 RDBE
0.7692 ppm
857.1521
246.2429
C 12 H 30 O N 4
0.0 RDBE
6.2291 ppm
m/z

^{1}H NMR ($CDCl_3$) spectrum of **3va**

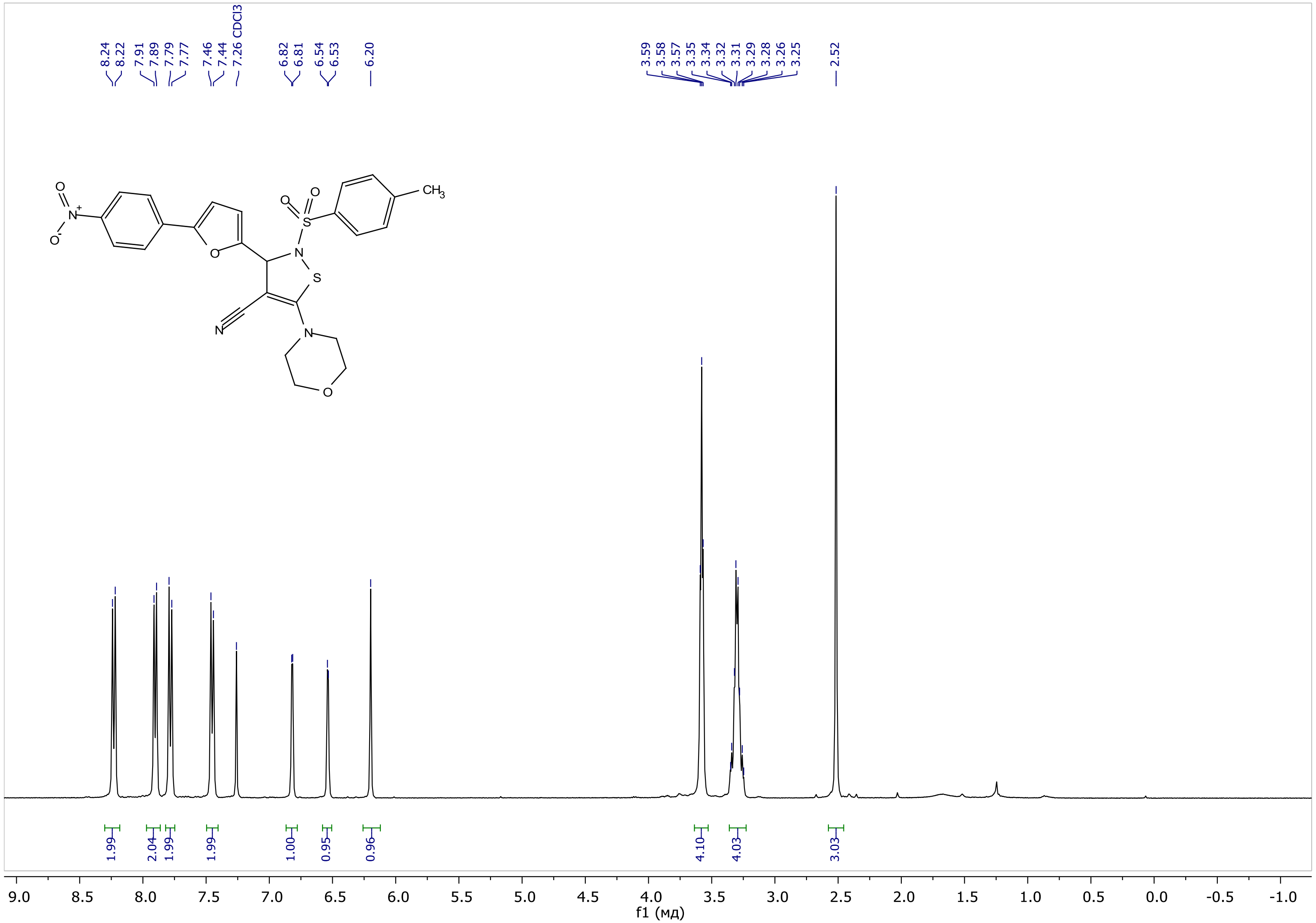

^{13}C NMR ($CDCl_3$) spectrum of **3va**

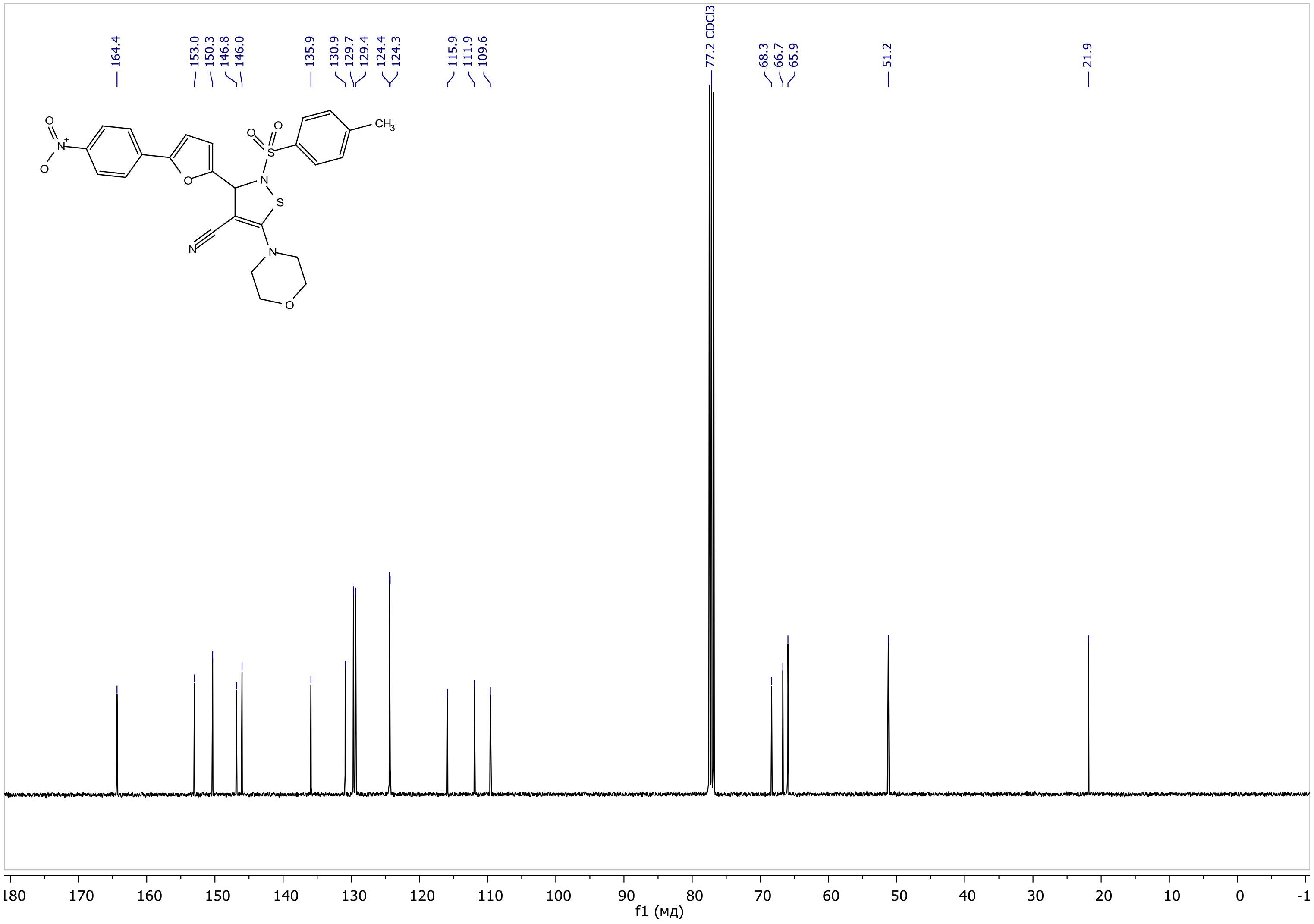

HRMS of **3va**

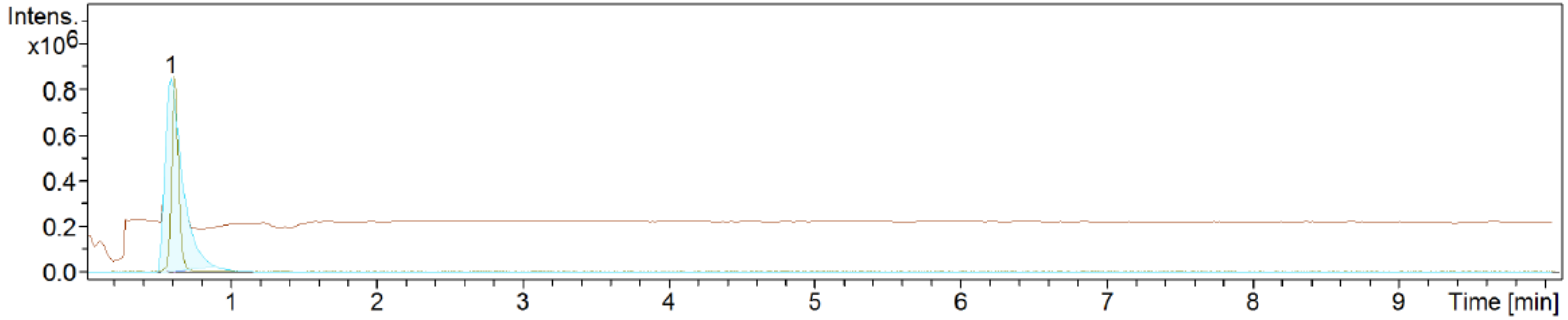

#	RT [min]	Area	Int. Type	I	S/N	Chromatogram	Max. m/z	FWHM [min]
1	0.6	6934571	Manual	841242	8952.5	EIC 539.1079±0.01 +All MS	539.1061	

Cmpd 1, 0.6 min

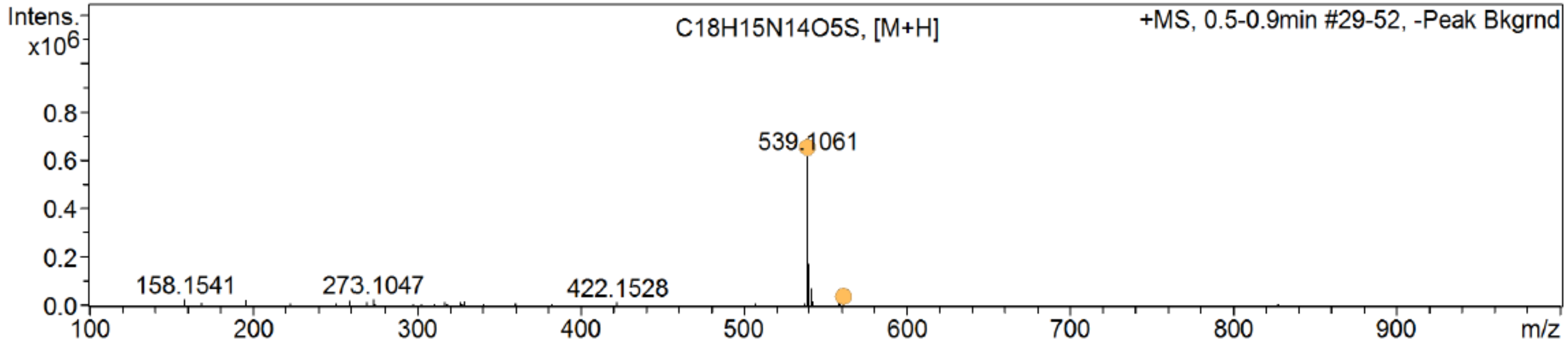

Meas. m/z	#	Ion Formula	m/z	err [ppm]	mSigma	# mSigma	Score	rdb	e¯ Conf	N-Rule
539.1061	1	C18H15N14O5S	539.1065	0.8	21.8	1	100.00	18.5	even	ok
561.0881	1	C18H14N14NaO5S	561.0885	0.6	66.1	1	100.00	18.5	even	ok

^{1}H NMR (DMSO-d_6) spectrum of **3wa**

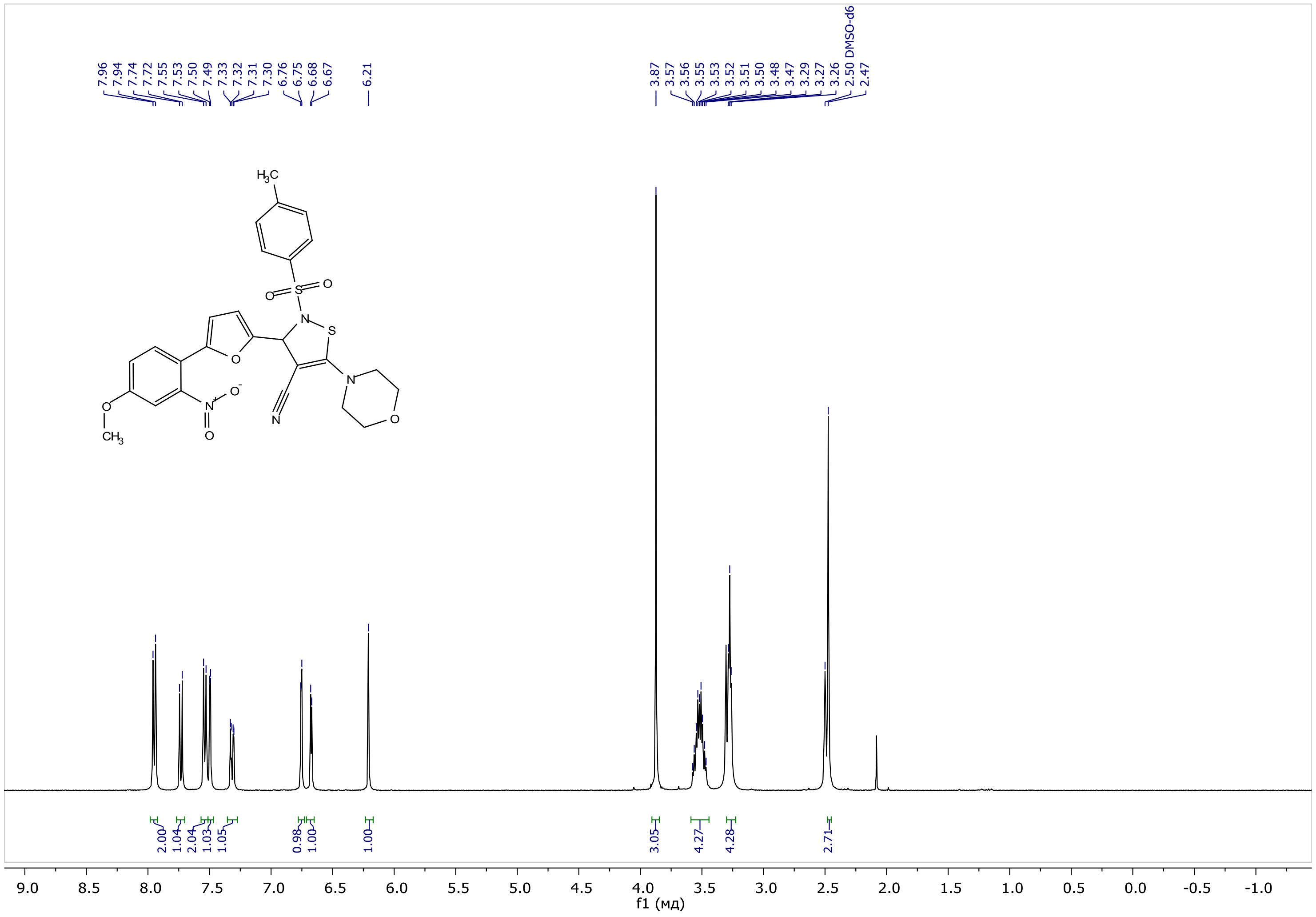

^{13}C NMR (DMSO-d_6) spectrum of **3wa**

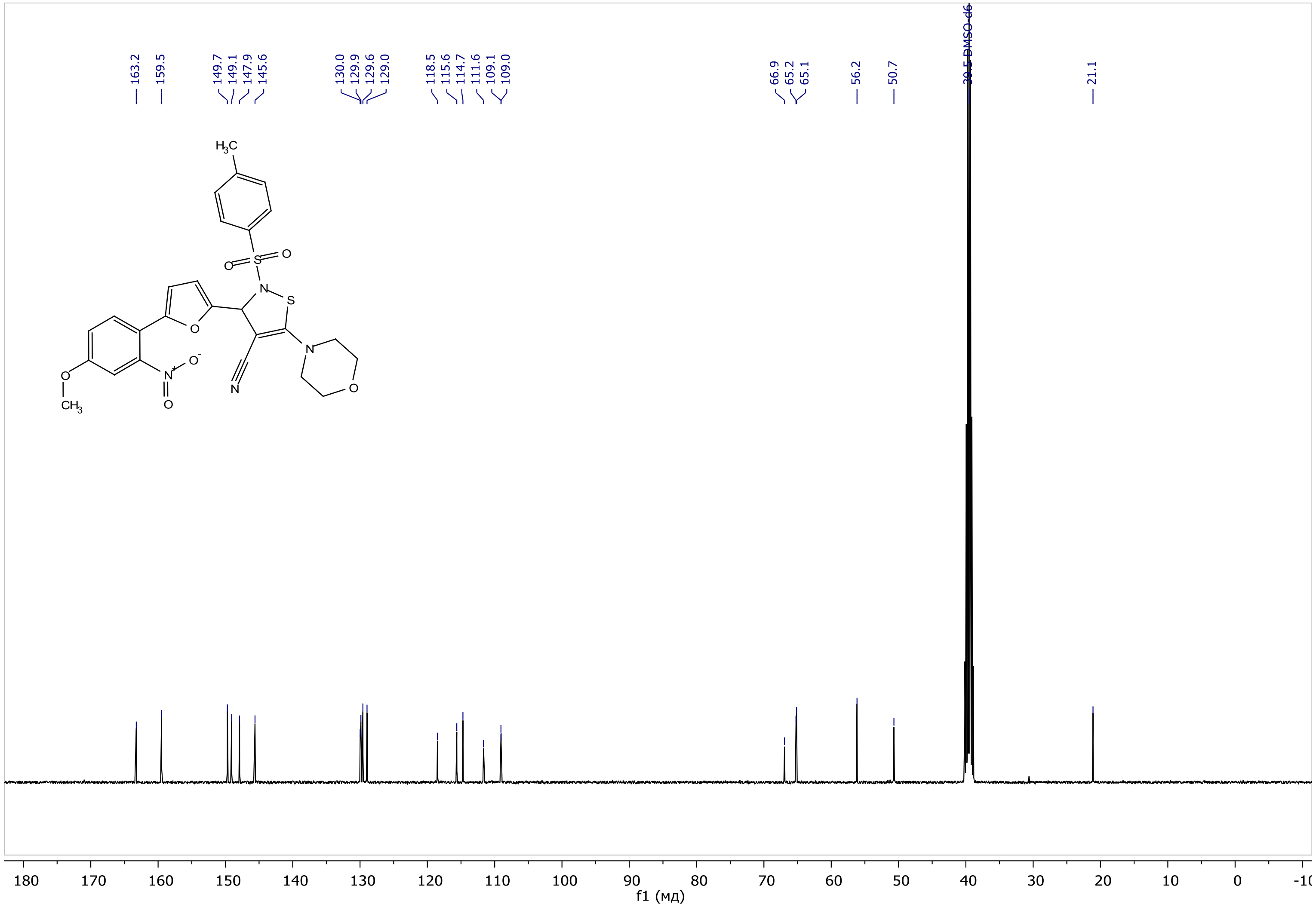

HRMS of **3wa**

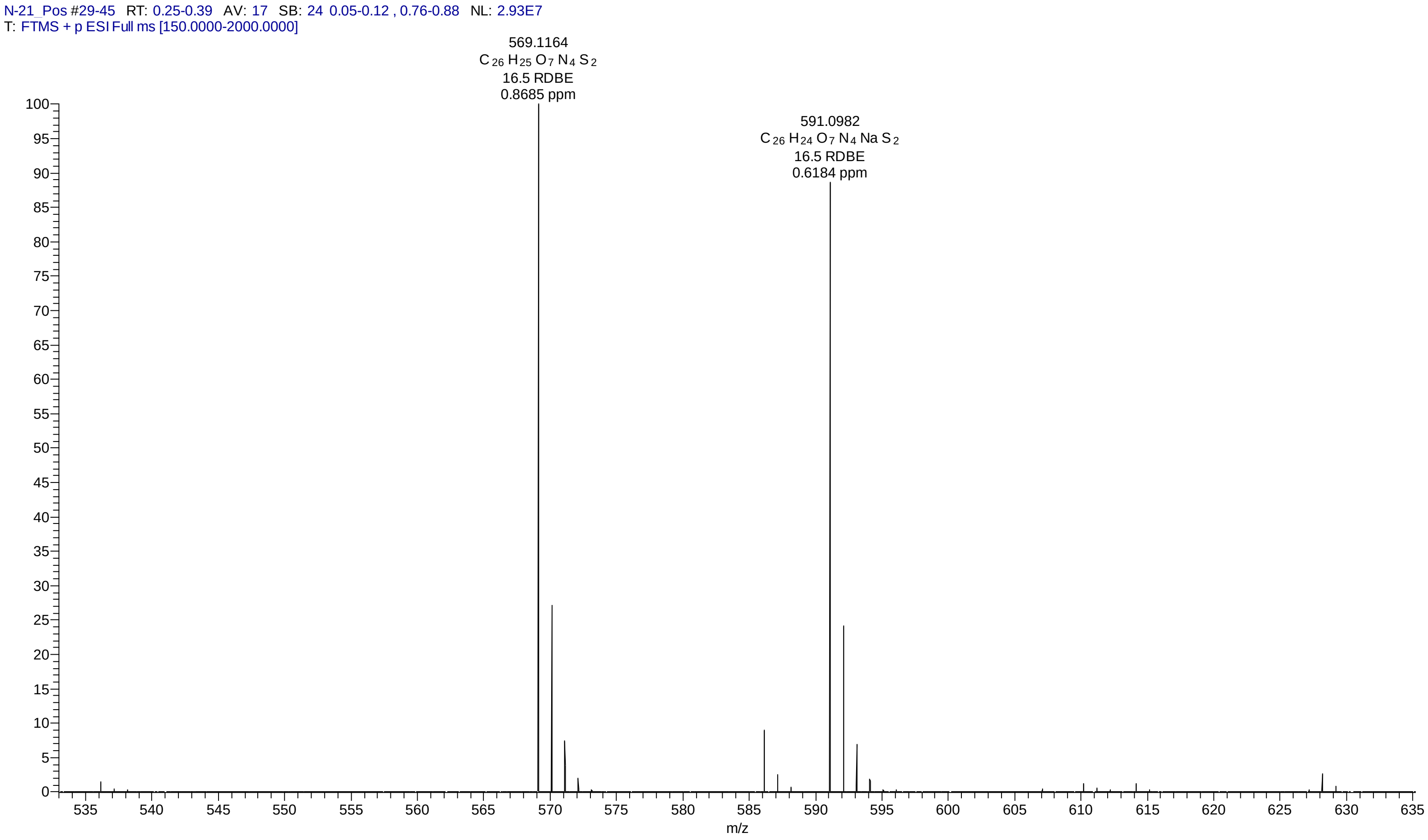

N-21_Pos #29-45 RT: 0.25-0.39 AV: 17 SB: 24 0.05-0.12 , 0.76-0.88 NL: 2.93E7
T: FTMS + p ESI Full ms [150.0000-2000.0000]
569.1164
C 26 H 25 O 7 N 4 S 2
16.5 RDBE
0.8685 ppm
591.0982
C 26 H 24 O 7 N 4 Na S 2
16.5 RDBE
0.6184 ppm
m/z

^{1}H NMR ($CDCl_3$) spectrum of **3xa**

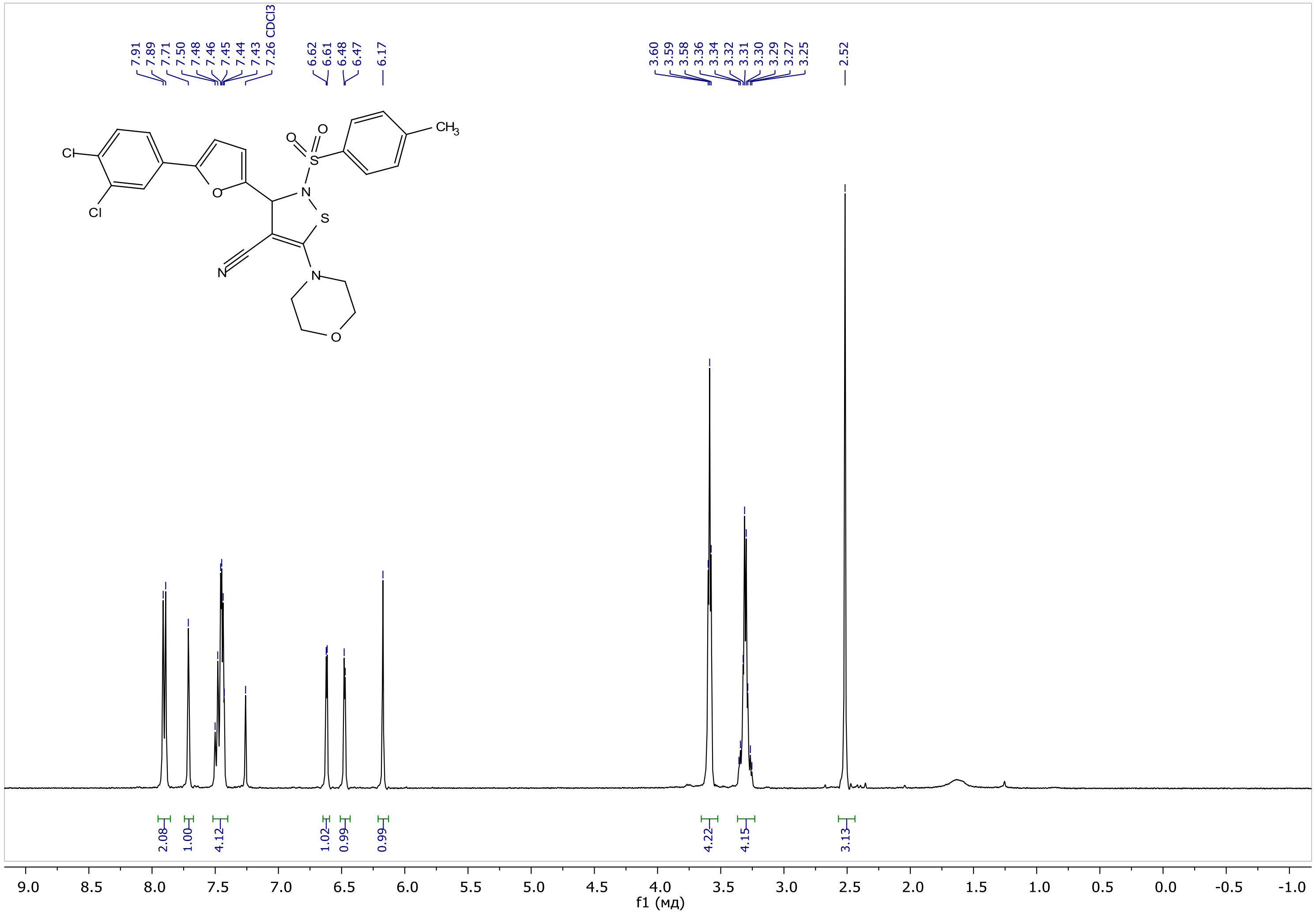

^{13}C NMR (CDCl$_3$) spectrum of **3xa**

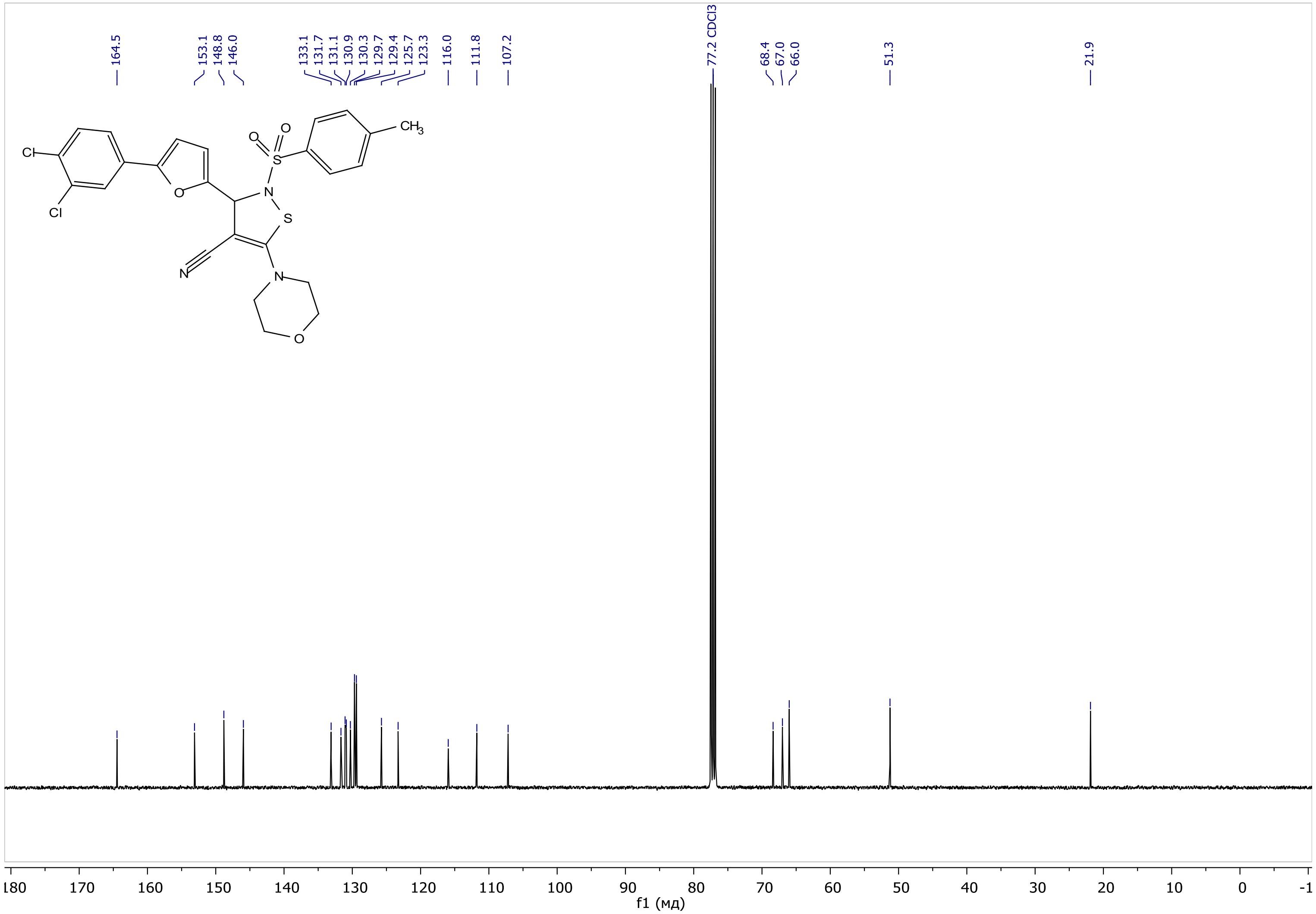

HRMS of **3xa**

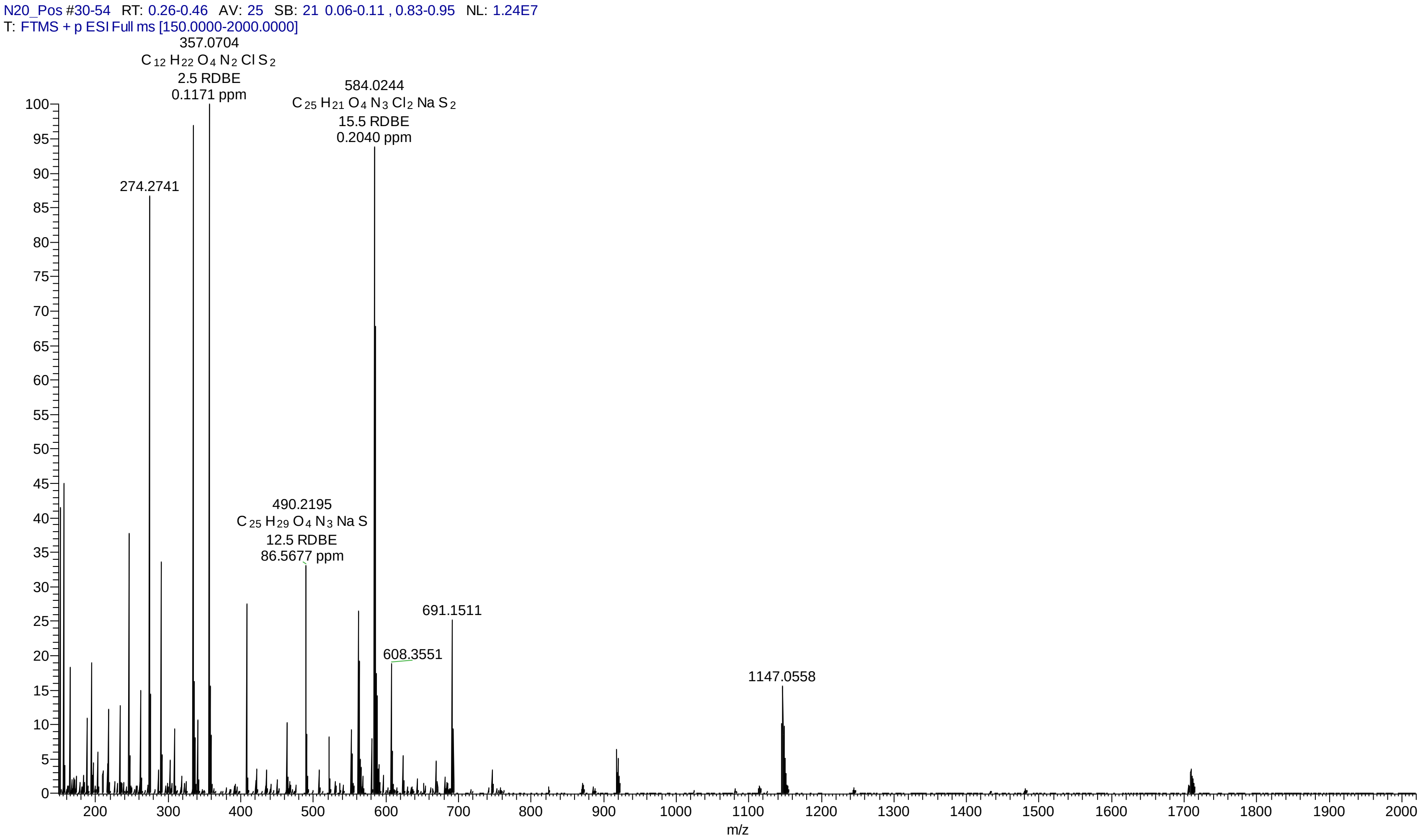

N20_Pos #30-54 RT: 0.26-0.46 AV: 25 SB: 21 0.06-0.11 , 0.83-0.95 NL: 1.24E7
T: FTMS + p ESI Full ms [150.0000-2000.0000]
357.0704
C 12 H 22 O 4 N 2 Cl S 2
2.5 RDBE
0.1171 ppm
584.0244
C 25 H 21 O 4 N 3 Cl 2 Na S 2
15.5 RDBE
0.2040 ppm
274.2741
490.2195
C 25 H 29 O 4 N 3 Na S
12.5 RDBE
86.5677 ppm
691.1511
608.3551
1147.0558
m/z

^{1}H NMR ($CDCl_3$) spectrum of **3ya**

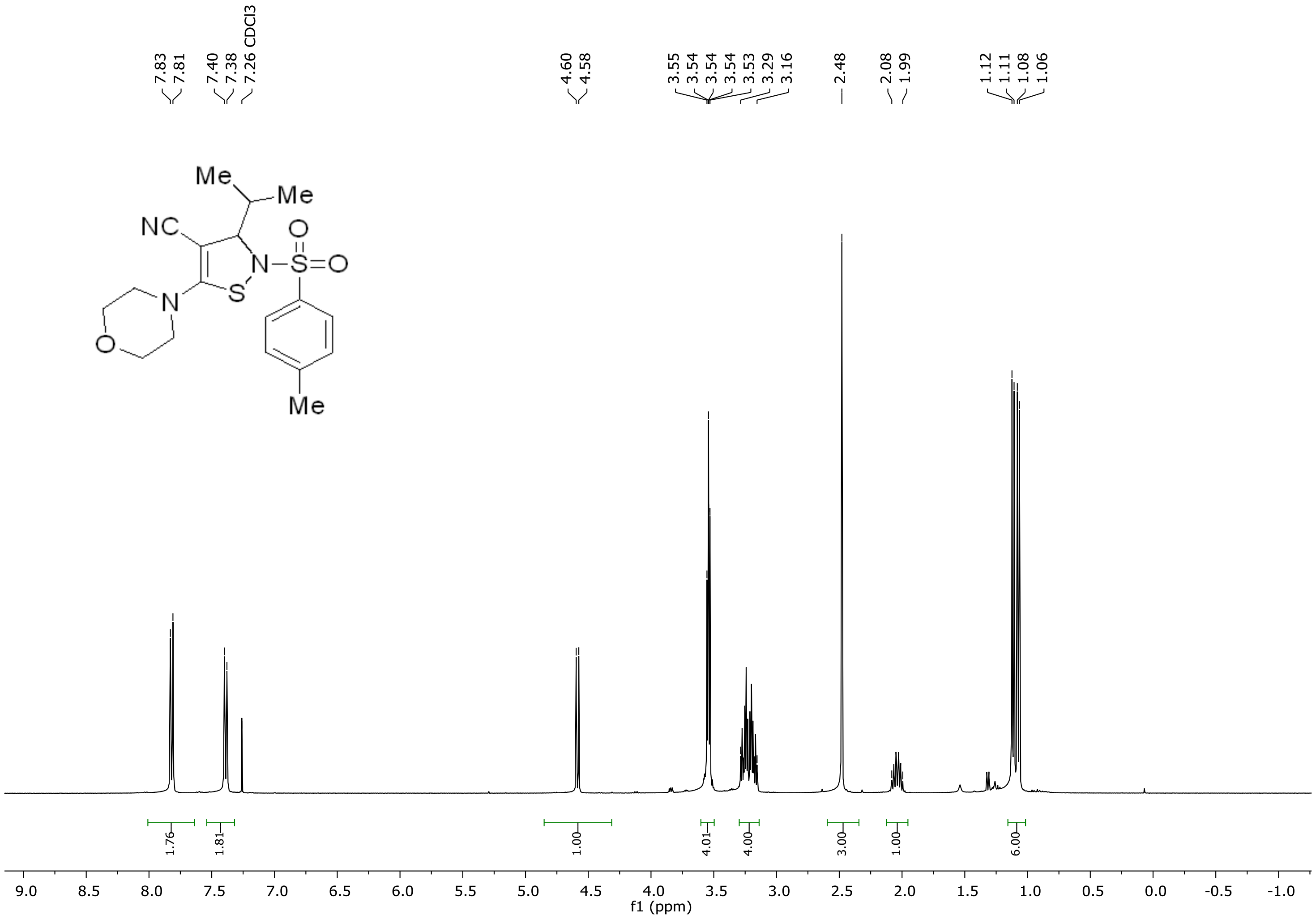

^{13}C NMR ($CDCl_3$) spectrum of **3ya**

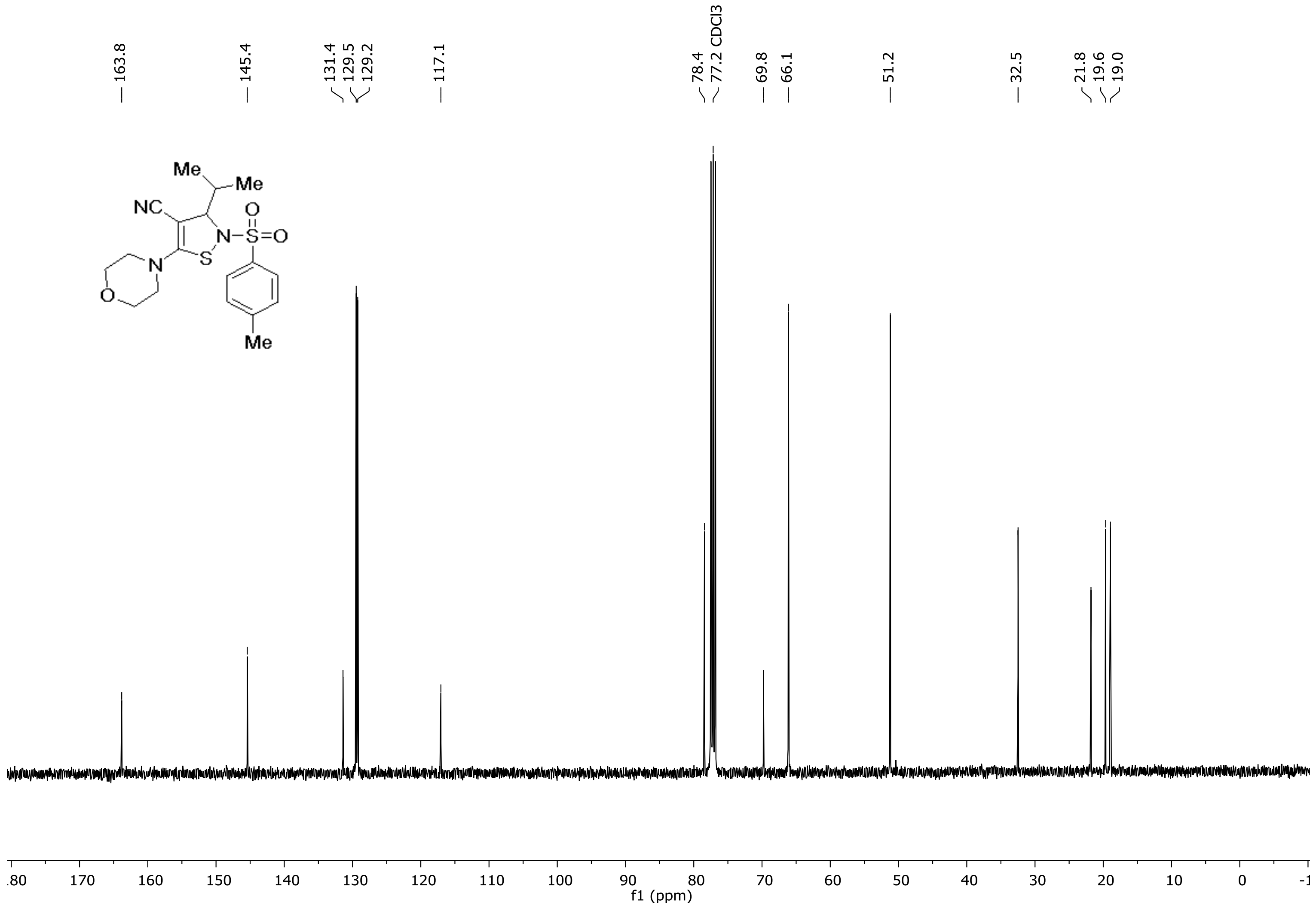

HRMS of **3ya**

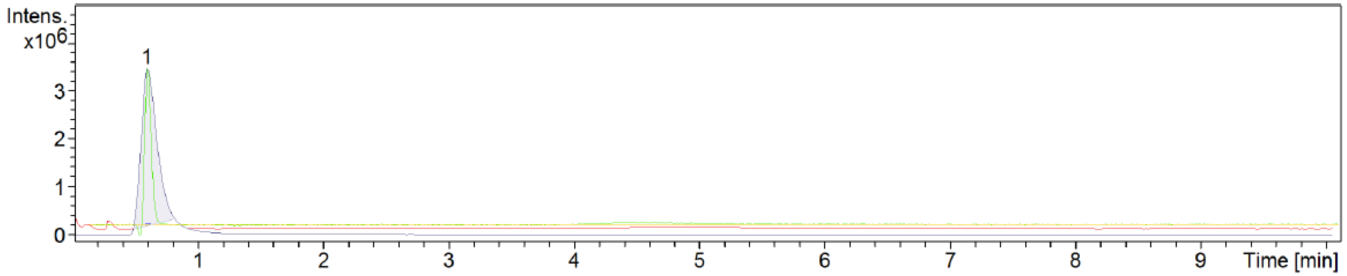

#	RT [min]	Area	Int. Type	I	S/N	Chromatogram	Max. m/z	FWHM [min]
1	0.6	28001498	Manual	3441882	10956.4	EIC 416.1075±0.02 +All MS	416.1075	

Cmpd 1, 0.6 min

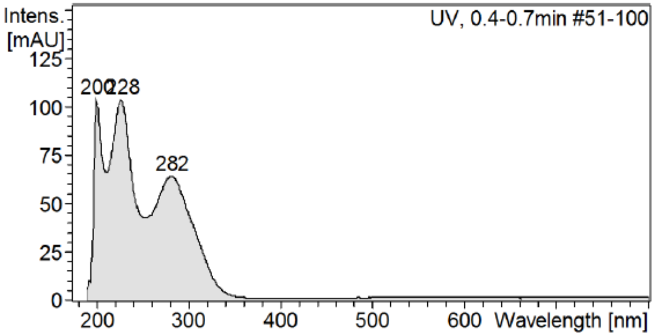

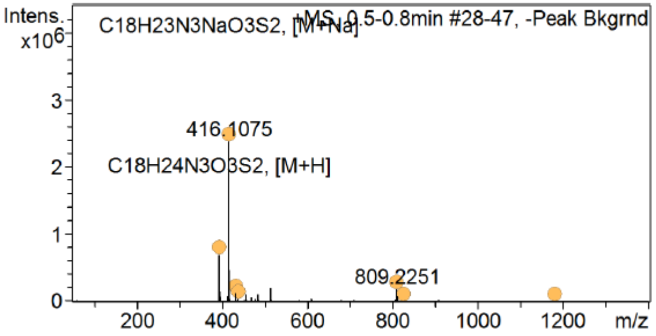

#	Wavelength	Intensity
0	200	103.4
1	228	103.4
2	282	64.3

#	m/z	Res.	S/N	I	I %	FWHM
1	394.1253	28045	533429088.0	696943	29.4	0.0141
2	395.1280	20363	114767552.0	149948	6.3	0.0194
3	416.1075	36261	1811445504.0	2366715	100.0	0.0115
4	417.1099	24977	351598240.0	459375	19.4	0.0167
5	418.1046	19490	162584656.0	212422	9.0	0.0215
6	432.0810	19893	95236416.0	124430	5.3	0.0217
7	456.0993	19770	76034040.0	99341	4.2	0.0231
8	484.0944	19878	81571528.0	106576	4.5	0.0244
9	514.1048	21448	153495024.0	200546	8.5	0.0240
10	809.2251	27488	137441984.0	179573	7.6	0.0294

^{1}H NMR ($CDCl_3$) spectrum of **3za**

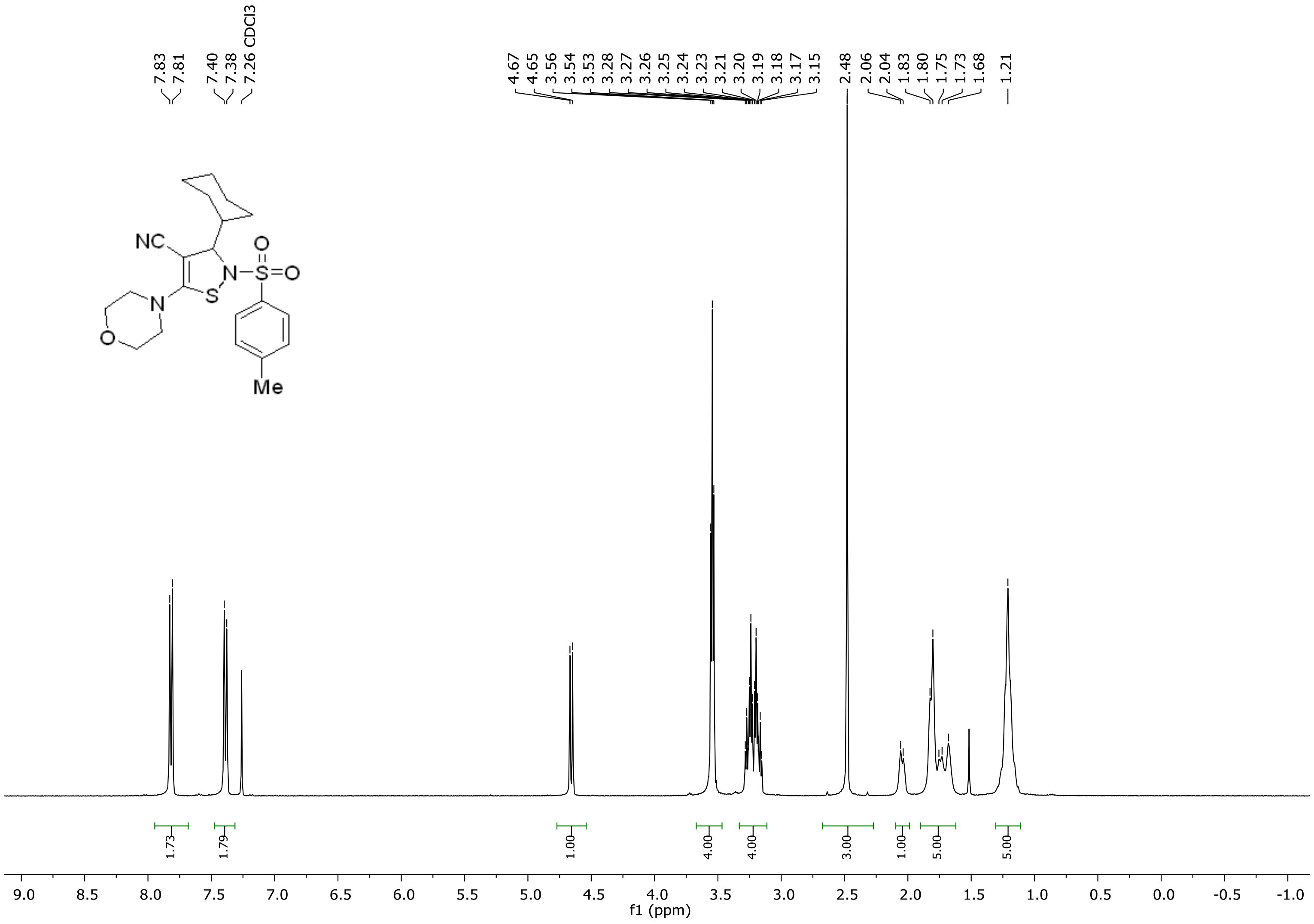

^{13}C NMR ($CDCl_3$) spectrum of **3za**

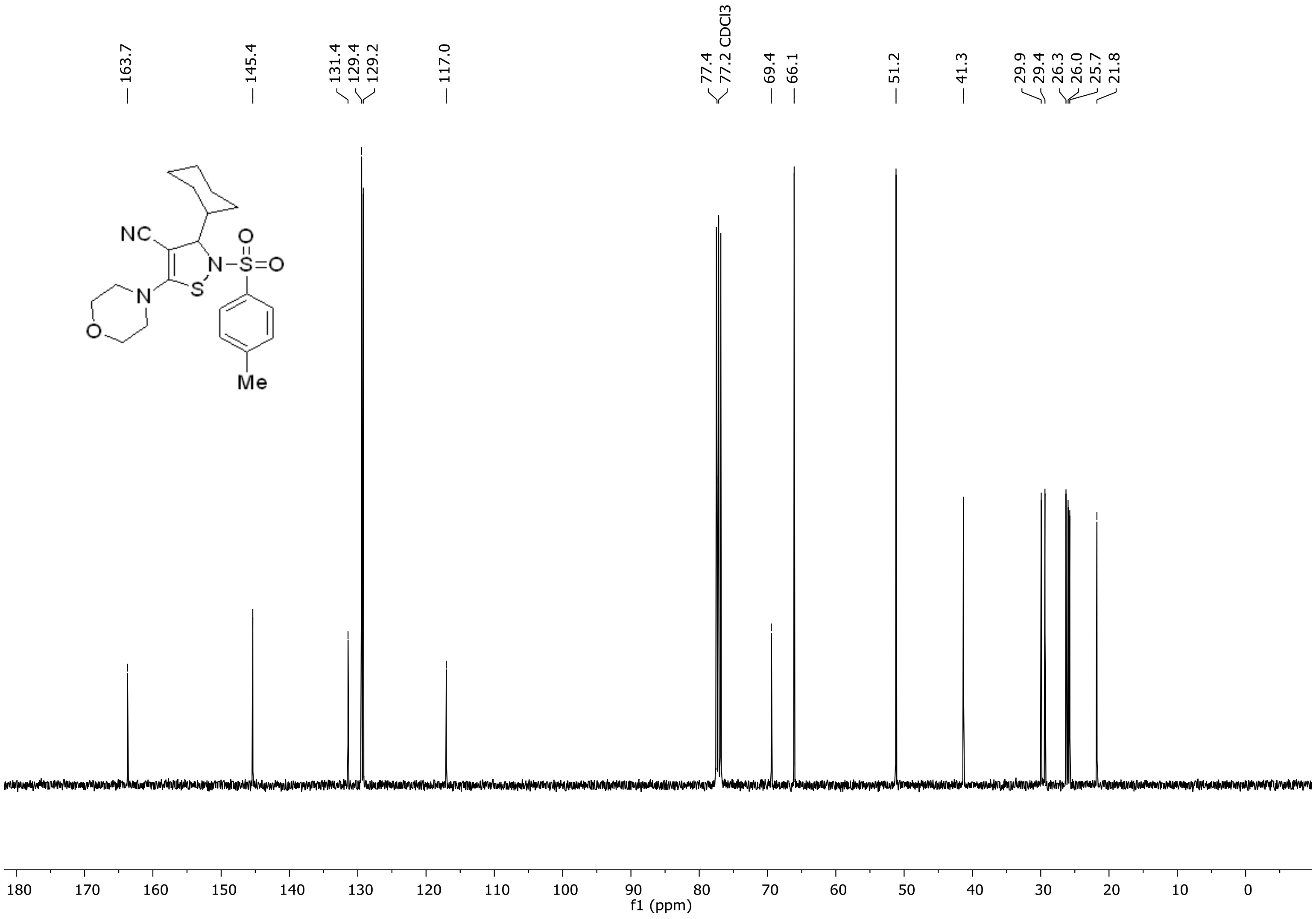

HRMS of **3za**

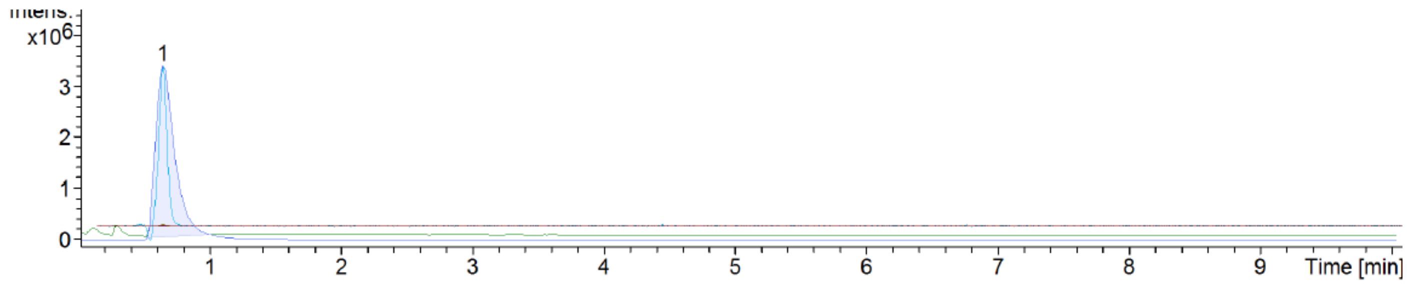

#	RT [min]	Area	Int. Type	I	S/N	Chromatogram	Max. m/z	FWHM [min]
1	0.6	33195916	Manual	3386411	17067.4	EIC 456.1390±0.02 +All MS	456.1390	

Cmpd 1, 0.6 min

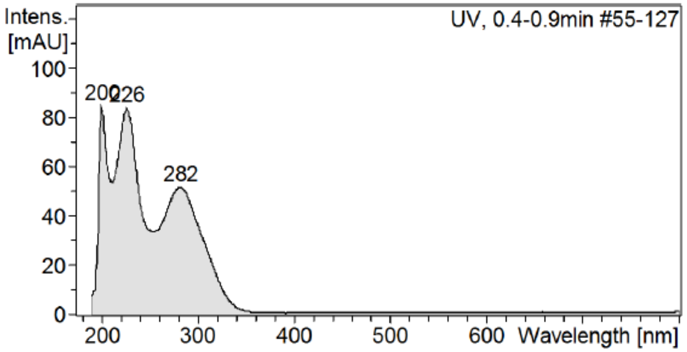

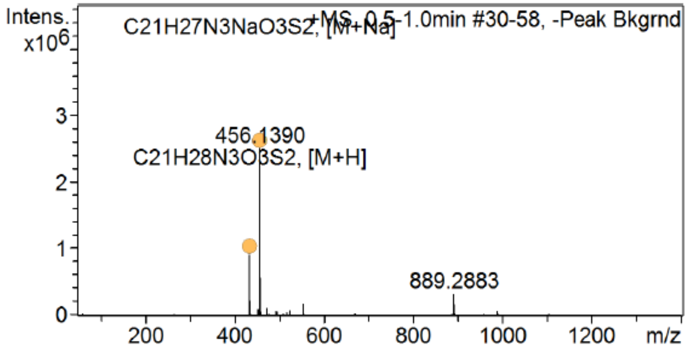

#	Wavelength	Intensity
0	200	84.4
1	226	83.6
2	282	51.9

#	m/z	Res.	S/N	I	I %	FWHM
1	434.1568	29792	34548200.0	920691	36.9	0.0146
2	435.1593	21144	8284613.0	220781	8.8	0.0206
3	436.1548	17322	3654905.5	97401	3.9	0.0252
4	456.1390	37563	93632856.0	2495267	100.0	0.0121
5	457.1416	26687	20877188.0	556366	22.3	0.0171
6	458.1370	19834	9099524.0	242498	9.7	0.0231
7	472.1124	19770	4194104.8	111771	4.5	0.0239
8	554.1365	20978	6182741.0	164767	6.6	0.0264
9	889.2883	32375	11791003.0	314224	12.6	0.0275
10	890.2909	25962	5869462.0	156418	6.3	0.0343
